# Rh(iii)-catalyzed heteroannular-selective heteroarylation of biaryls: facile access to heteroacenes with sulfur-embedded 5–7 ring topology

**DOI:** 10.1039/d5sc08209a

**Published:** 2026-01-03

**Authors:** Zhanhui He, Li Yang, Menghang Zhou, Yue Zhong, Zheng Liu, Ziao Zhang, Feiyang Xia, Xuezhe Deng, Shuang Yan, Cheng Xu, Cheng Zhang, Guodong Yin

**Affiliations:** a Hubei Key Laboratory of Pollutant Analysis and Reuse Technology, College of Chemistry and Chemical Engineering, Hubei Normal University Huangshi 435002 P. R. China zhengliu0827@hbnu.edu.cn gdyin@hbnu.edu.cn; b Medical Imaging Key Laboratory of Sichuan Province, North Sichuan Medical College Nanchong 637000 P. R. China; c Key Laboratory of Green Chemistry and Technology of Ministry of Education, College of Chemistry, Sichuan University 29 Wangjiang Road Chengdu 610064 P. R. China cheng.zhang@scu.edu.cn

## Abstract

Acenes with non-benzenoid 5–7 ring topology have drawn significant attention. Nevertheless, introducing sulfur atoms into 5/7-membered rings of acenes has not yet been achieved owing to synthetic challenges. Disclosed herein is a rhodium-catalyzed heteroannular-selective C–H/C–H oxidative cross-coupling reaction of 2-substituted biaryls with heteroarenes, which provides a convenient approach to heteroacenes with sulfur-embedded 5–7 ring topology. This protocol demonstrates excellent regioselectivity and broad functional group tolerance, enabling the efficient synthesis of a diverse library of thioether- and seleno-ether-substituted biheteroaryl compounds. Heteroacenes featuring sulfur-embedded 5–7 or 5–6 ring topologies can be switchably constructed through further intramolecular cyclization involving directing groups. Crystal structure analysis, along with studies on photophysical properties and aromaticity, indicates that heteroacenes with sulfur-embedded 5–7 ring topology have the potential as high-performance organic semiconductor materials.

## Abstract 2

The heteroacenes with sulfur-embedded 5–7 or 5–6 ring topologies are switchably obtained *via* heteroannular-selective oxidative cross-coupling of biaryls with heteroarenes, followed by directing group-involved intramolecular cyclization.

## Graphical abstract

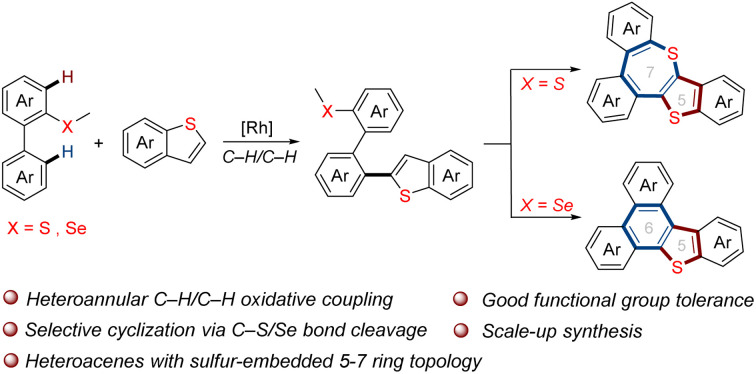

## Introduction

**Scheme 1:**
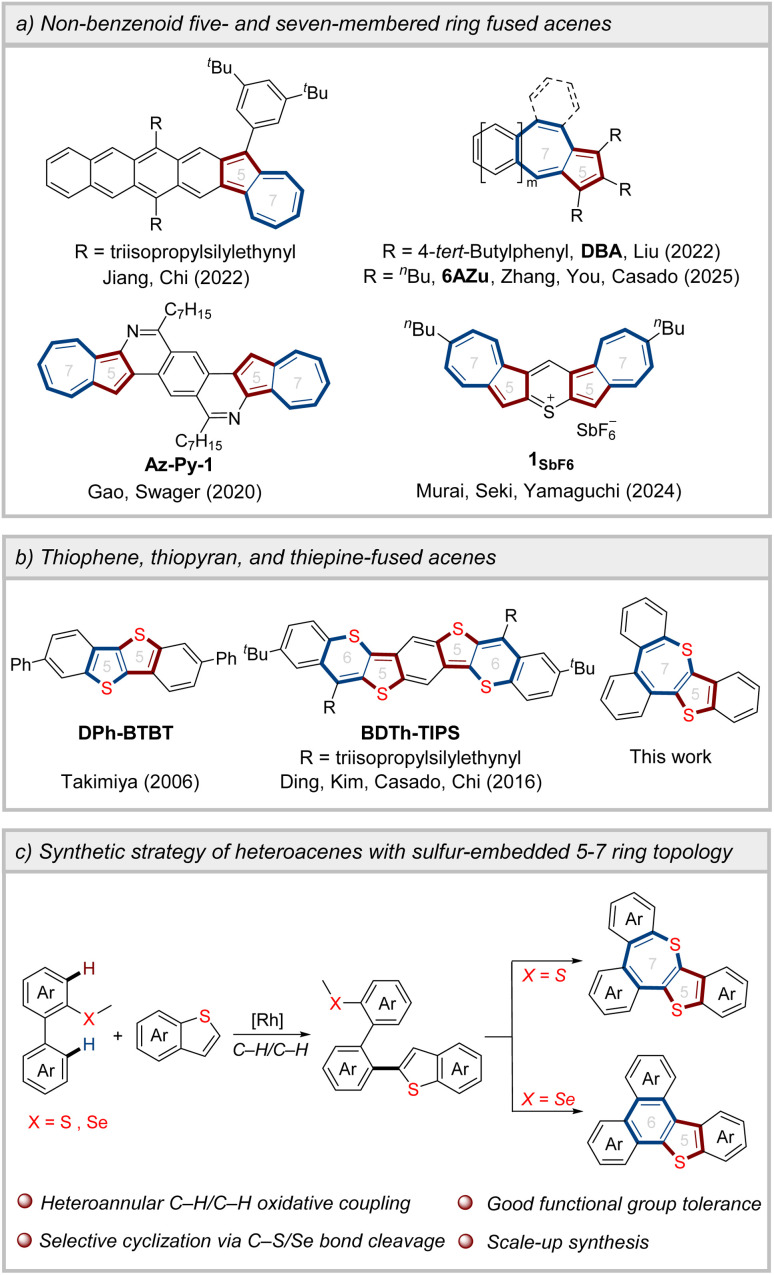
Design and synthetic strategy of heteroacenes with sulfur-embedded 5–7 ring topology.

Acenes, characterized by linearly arranged benzenoid units, have attracted significant attention in the field of organic field-effect transistors (OFETs) due to their remarkable charge transport performance.^1^ Nevertheless, larger acenes suffer from high sensitivity to oxidation by O_2_ and readily undergo dimerization *via* formal [4 + 4] cycloaddition reactions, severely limiting their applications as organic functional materials.^2^ The introduction of non-benzenoid five- and seven-membered ring arrays has emerged as a pivotal strategy to modulate the stability and optoelectronic properties of acenes (Scheme 1a).^3^ In 2022, Jiang, Chi, and co-workers reported azulene-fused acenes with a 6–5–7 ring topology, demonstrating enhanced chemical stability compared to corresponding acenes.^4^ Concurrently, acenes fused with azulene in a 6–7–5 ring configuration display anti-Kasha emission (even with double anti-Kasha bands) and function as hole-transporting semiconductors.^5^ The integration of 5–7 ring pairs into heteroacene frameworks has also been explored.^6^ For example, Gao, Swager *et al.* successfully synthesized *N*-doped heptacene incorporating azulene units, which demonstrated exceptional electronic transport properties.^7^ Additionally, sulfur-bridged azulene-based acene forms charge-segregated assembly in the crystalline state, achieving a high charge-carrier mobility of 1.7 cm^2^ V^−1^ s^−1^.^8^ Notably, the incorporation of diverse heteroatoms not only tunes the electronic properties and intermolecular interactions of acenes, but also significantly influences the optoelectronic performance of organic functional materials.^9^ To date, the development of novel heteroacenes through embedding heteroatoms into 5–7 ring pairs remains highly desirable.^10^

As an isoelectronic counterpart of benzene, thiophene exhibits good planarity and aromaticity. Thiophene-fused materials not only demonstrate enhanced antioxidative stability,^11^ but also enable the formation of intermolecular S⋯S, S⋯H, and S⋯π interactions.^12^ These noncovalent interactions facilitate efficient charge transport and may significantly influence molecular packing arrangements. Therefore, thiophene-fused acenes exhibit excellent OFET performance,^13^ and the maximum mobility of thin-film OFETs based on DPh-BTBT featuring five- and five-membered ring arrays reaches 2.0 cm^2^ V^−1^ s^−1^ (Scheme 1b).^14^ Despite the disruption of the aromaticity of planar six-membered thiopyran,^15^ thiophene- and thiopyran-fused polycyclic aromatics with five- and six-membered ring structures have drawn considerable attention.^16^ In contrast, thiepine-based skeletons incorporating a sulfur-containing seven-membered ring have rarely been reported, primarily owing to synthetic challenges.^17^

C–H activation has emerged as a powerful strategy for constructing π-conjugated structures,^18^ especially through C–H arylation/cyclization approaches, which significantly simplify synthetic pathways.^19^ We envisioned that the heteroannular-selective heteroarylation of biaryls, followed by directing group-participated intramolecular cyclization, could serve as an effective strategy for constructing sulfur-embedded heteroacenes (Scheme 1c). Nevertheless, achieving the C–H/C–H oxidative coupling reaction of biaryls remains a challenge due to poor regioselectivity, as chelation-assisted transition metal-catalyzed C–H bond activation predominantly occurs at the *ortho*-position of the directing groups.^20^ When employing a thioether as the directing group, the transition metal coordinates with the sulfur atom and activates the heteroannular C–H bond of the biaryl, leading to the formation of a six-membered cyclometalated intermediate.^21^ This intermediate has lower ring strain compared to the four-membered cyclometalated intermediate generated by activating the *ortho*-C–H bond, thus enabling regioselectivity.^22^ Herein, we present the heteroannular-selective heteroarylation of biaryls for constructing thiophene- and thiepine-fused polycyclic aromatics. Crystal structure analysis and DFT calculations indicate that heteroacenes with sulfur-embedded 5–7 ring topology have the potential as high-performance organic semiconductor materials.

## Results and discussion

The initial investigation into the oxidative heteroannular-selective cross-coupling reaction was carried out using (3′-(*tert*-butyl)-[1,1′-biphenyl]-2-yl)(methyl)sulfane (1a) and benzo[*b*]thiophene (2a) as model substrates under the catalytic system of [Cp*RhCl_2_]_2_/AgSbF_6_. To our delight, when Ag_2_O was used as an oxidant, and pivalic acid (PivOH) as an additive in tetrahydrofuran (THF), the desired product 3a was obtained in 35% yield (Table S1, entry 1). The solvent screening indicated that DCE could enhance the yield of 3a to 41% (Table S1). The catalysts of Pd(ii), Ir(iii), and Rh(iii) could all promote the coupling reaction. Notably, Cp*Rh(MeCN)_3_[SbF_6_]_2_ demonstrated superior catalytic performance (Table S2). Further optimization of the oxidant revealed that Ag_2_O was a more appropriate choice, and other oxidants, such as Ag_2_CO_3_, AgOAc, and AgF, exhibited lower efficiency than Ag_2_O (Table S3). Additionally, it was found that the addition of bases had an inhibitory influence on the reaction, while acid additives could promote the formation of 3a (Table S4). Attempts to decrease the amount of 2a were unsuccessful, as the heteroarene decomposed under the reaction conditions (Table S5, entries 7 and 8). Further reduction of the oxidant Ag_2_O loading to 3.0 equivalents resulted in a lower yield of 71% (Table S5, entry 9). Finally, 3a was synthesized with a yield of 76% by employing Cp*Rh(MeCN)_3_[SbF_6_]_2_ as a catalyst, Ag_2_O as an oxidant, and PivOH as an additive in DCE at 120 °C for 24 h (Table S5, entry 2).

**Scheme 2:**
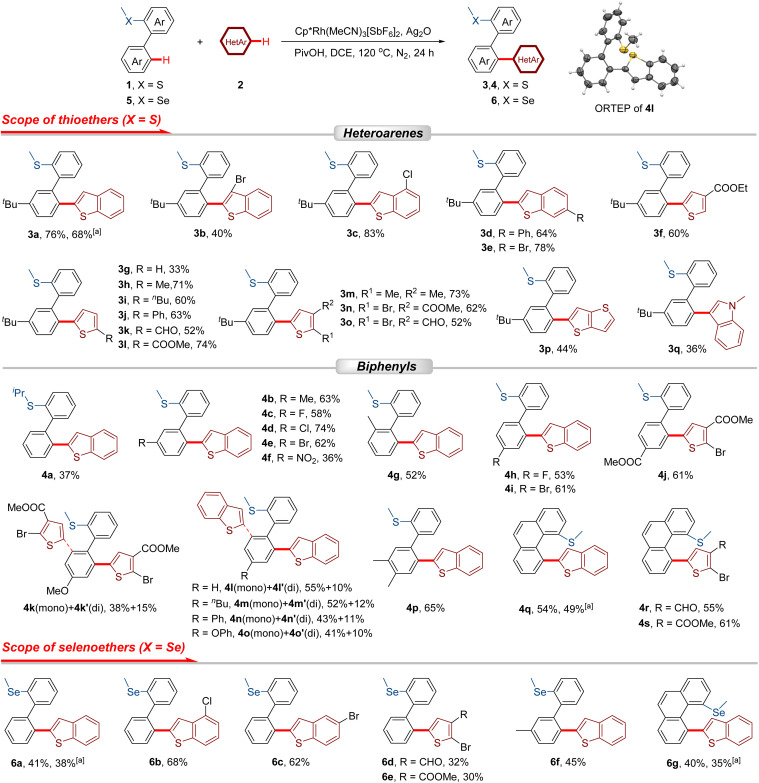
Scope of substrates. Reaction conditions: 1 or 5 (0.2 mmol), 2 (0.6 mmol), Cp*Rh(MeCN)_3_[SbF_6_]_2_ (3 mol%), Ag_2_O (0.7 mmol), PivOH (0.4 mmol) and DCE (1.0 mL) at 120 °C for 24 h under N_2_. Isolated yields. ^*a*^2.0 mmol scale.

With the optimized conditions established, a systematic exploration of the scope of heteroarenes (2) was conducted using 1a as the coupling reagent (Scheme 2). The 3-, 4-, and 6-substituted benzo[*b*]thiophenes gave rise to the coupling products with yields ranging from 40% to 83% (3b–3e). When thiophenes were employed as the heteroarylation reagent, the cross-coupling reaction proceeded smoothly, affording the corresponding products in 33–74% yields (3f–3o). This catalytic system exhibited excellent tolerance towards thiophenes bearing electron-donating groups (–Me, –^*n*^Bu, and –Ph) and electron-withdrawing groups (–CHO and –COOMe) at the C2 position (3h–3l, 52–74%). Furthermore, several disubstituted thiophenes were also identified as suitable substrates for this reaction (3m–3o, 52–73%). Additionally, thieno[3,2-*b*]thiophene (2p) and *N*-methylindole (2q) were demonstrated to be compatible with the catalytic system. Nevertheless, the yields of the resultant products 3p and 3q were relatively low, attaining values of 44% and 36%, respectively.

Subsequently, the substrate scope was extended to 2-thioether-substituted biphenyls. The steric hindrance caused by the enlarged directing group (–S^i^Pr) led to relatively low coupling efficiency (4a, 37%). Functional groups (–Me, –F, –Cl, –Br, and –NO_2_) substituted at the *meta*-position of [1,1′-biphenyl]-2-yl(methyl)sulfane can be well tolerated (4b–4f, 36–74%). The substrate with a methyl group at the *ortho*-position afforded heteroarylation product (4g) with a yield of 52%. The cross-coupling could also work well with *para*-substituted 2-thioether biphenyls (4h–4k, 4m–4o). For unsubstituted biaryls or those bearing *para*-electron-donating groups, both mono- and di-heteroarylated products were obtained (4k–4o, 4k′–4o′), whereas substrates featuring *para*-electron-withdrawing substituents exclusively afforded the mono-heteroarylated derivatives (4h–4j), with only trace amounts of the corresponding di-heteroarylated species observed. Notably, in the catalytic systems involving *ortho*- and *meta*-substituted biaryl substrates, no di-heteroarylated products were detected, which can be attributed to steric hindrance. The structure of product 4l was further confirmed by X-ray crystallography. (3′,4′-Dimethyl-[1,1′-biphenyl]-2-yl)(methyl)sulfane and methyl(phenanthren-4-yl)sulfane could undergo the oxidative coupling reactions in moderate yields (4p–4s, 54–65%).

As a member of the same group as the sulfur atom, the selenium atom could also serve as a directing group to facilitate the selective heteroannular C–H bond activation of biphenyls, thereby enriching the diversity of products. A series of selenoether-substituted biheteroaryls were successfully synthesized under the rhodium catalytic system (6a–6g, 30–68%).

The scale-up synthesis of compounds 3a, 4q, 6a, and 6g was successfully accomplished on a 2.0 mmol scale, paving the way for the further development and construction of heteroacenes (Scheme S4). In addition, the oxidant Ag_2_O could be regenerated through treatment with HNO_3_ and NaOH. When the regenerated Ag_2_O was reused as the oxidant, the performance of the oxidative coupling reaction remained unaffected (Scheme S5).

**Scheme 3:**
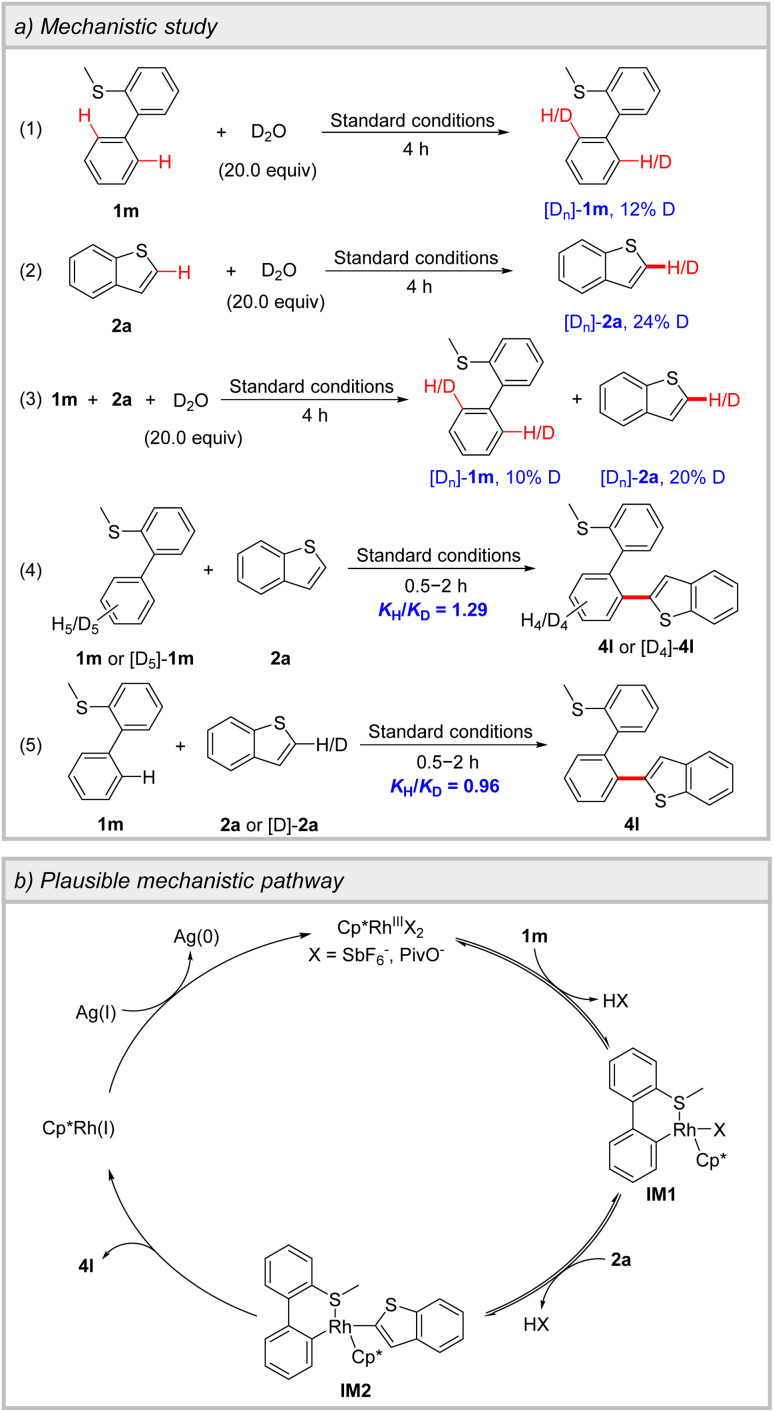
Mechanistic study and plausible mechanistic pathway.

To delve deeper into the heteroannular-selective oxidative cross-coupling, the H/D exchange experiments were performed (Scheme 3a). In the absence and presence of 2a, 12% and 10% deuterium rates of C2′–H of biphenyl in 1m were observed, respectively, indicating the reversible process of the heteroannular C–H metalation (Schemes S6 and S8). Furthermore, the reaction of 2a with D_2_O resulted in 24% and 20% deuterium rates of C2–H of 2a in the absence and presence of 1m, respectively, demonstrating that the cleavage of C2–H of 2a is reversible (Schemes S7 and S8). Kinetic isotope effect (KIE) experiments were conducted for 1m and 2a. A KIE value of 1.29 was observed in the parallel reactions of 1m and [D_5_]-1m with 2a, while a KIE value of 0.96 was detected in the parallel reactions between 2a and [D]-2a with 1m. These results suggest that the C–H cleavage of 1m and 2a may not be involved in the rate-determining step (Tables S6 and S7).

The plausible mechanistic pathway was proposed (Scheme 3b). The coordination of 1m to Rh(iii), followed by heteroannular C–H activation, leads to the formation of a six-membered cyclometalated complex IM1. Subsequently, the second C–H activation on 2a delivers intermediate IM2. The reductive elimination of IM2 gives product 4l and Rh(i), which is then oxidized by Ag(i) and re-enters the catalytic cycle.

**Scheme 4:**
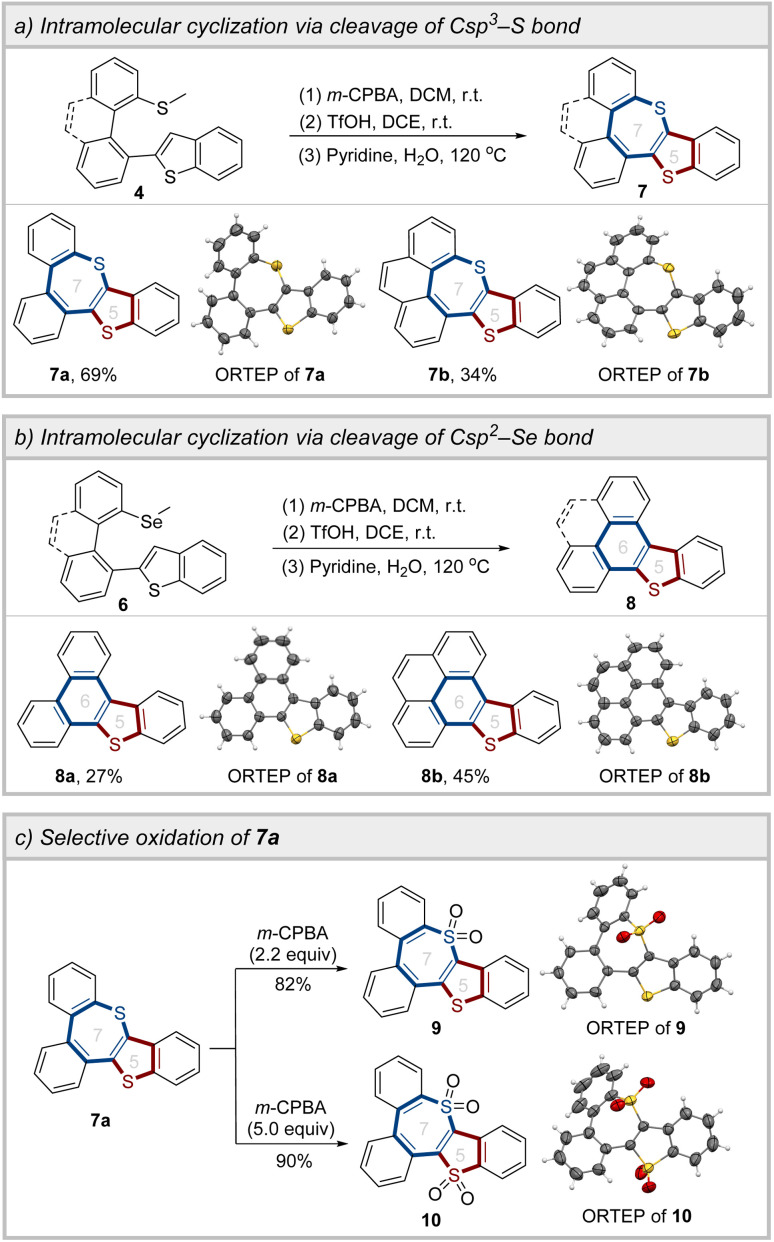
Synthetic applications.

Following the preparation of the coupling products, the intramolecular cyclization reaction was carried out to construct the sulfur-embedded polycyclic aromatics. Under the oxidation/cyclization reaction conditions, compounds 7a and 7b were successfully synthesized, with yields of 69% and 34%, respectively, *via* the cleavage of the C sp^3^–S bond (Scheme 4a). Intriguingly, under identical experimental conditions, the selenomethyl-substituted biheteroaryls (6a and 6g) underwent cleavage of the C sp^2^–Se bond, leading to the formation of the six-membered ring products 8a and 8b (Scheme 4b). Moreover, by adjusting the equivalents of the oxidant, it was feasible to achieve the selective oxidation of 7a, thereby obtaining compounds 9 and 10 (Scheme 4c).

**Fig. 1:**
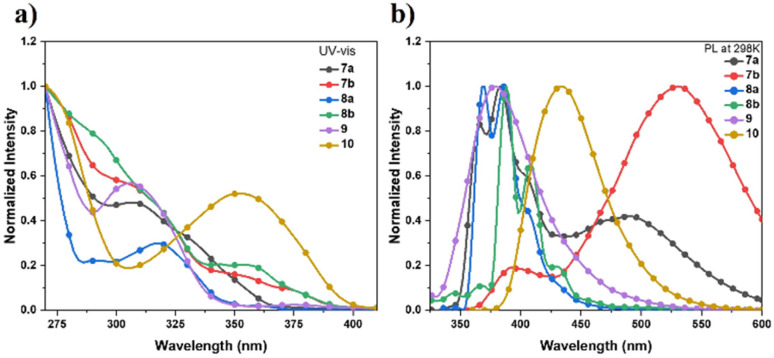
(a) Absorption spectra and (b) fluorescence emission spectra of 7a, 7b, 8a, 8b, 9, and 10 in CH_2_Cl_2_ (1.0 × 10^−5^ M).

The UV-vis absorption and fluorescence emission spectra of these sulfur-embedded polycyclic aromatics in diluted CH_2_Cl_2_ (1.0 × 10^−5^ mol L^−1^) were measured (Fig. 1, S1 and Table S8). The absorption maxima of compounds 7a, 7b, 8a, and 8b are observed at 306, 304, 318, and 354 nm, respectively. Compared with 7a, the oxidation of the sulfur atoms to sulfone groups in compound 10 results in a bathochromically shifted absorption peak from 306 nm to 354 nm. According to the time dependent density functional theory (TD-DFT) calculations (Tables S9–S14), the absorption maxima of 7a and 9 were assigned to the S_0_ → S_2_ excitation, the shoulder peak of 7b between 310–380 nm were ascribed to the transitions of S_0_ to S_*n*_ (*n* = 1–4). Compounds 7a and 7b exhibit photoluminescence peaks at 360, 384, 500 nm and 392, 532 nm, respectively. The bathochromic-shifted emission band of 7b can be attributed to the extension of π-conjugation. In contrast, compounds 8a, 8b, 9, and 10 display relatively blue-shifted emission bands.

**Fig. 2:**
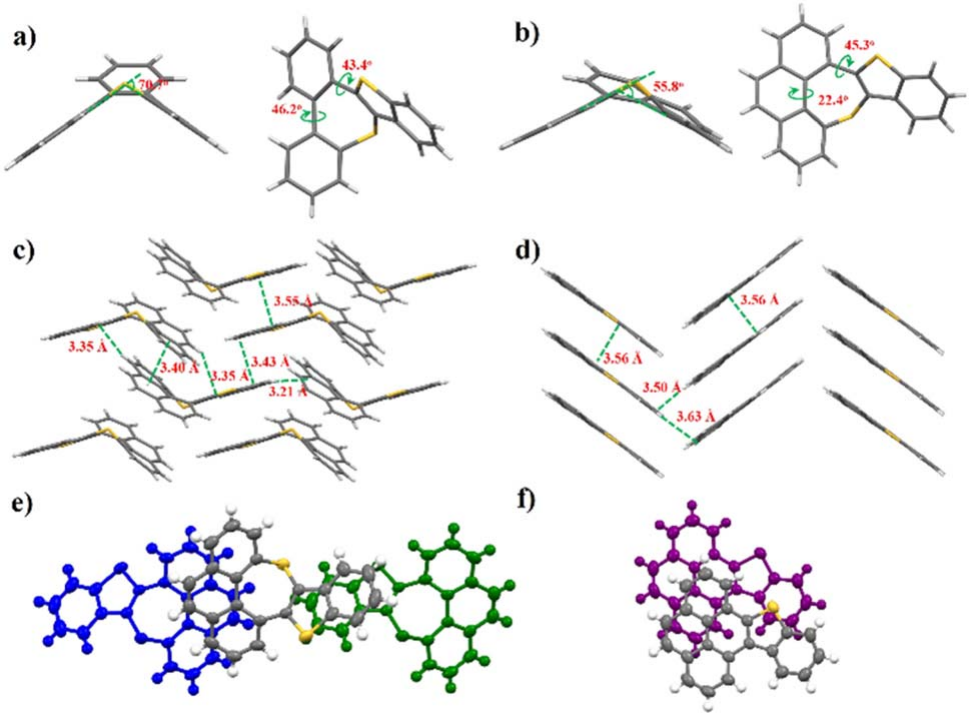
X-ray single-crystal structures of (a) 7a, and (b) 7b; crystal packings of (c) 7b, and (d) 8b; π-stacked molecules of (e) 7b, and (f) 8b.

Single crystals of these sulfur-embedded polycyclic aromatics were obtained *via* the solvent diffusion method with dichloromethane and hexane. The crystal structures and packing modes of these six single crystals are shown in Fig. 2 and S2–S8. Moreover, detailed crystallographic data are summarized in Tables S16–S21. Owing to the existence of the sulfur-containing seven-membered ring, compound 7a presents a V-shaped geometry rather than a plane one. A C–S–C bond angle of 70.7° is observed, and the dihedral angle between the benzene ring and the thiophene ring is measured to be 43.4°. The distance between the S atoms is 3.38 Å, indicating the existence of S⋯S interactions among molecules. In the π-extended 7b, the C–S–C bond angle decreases to 55.8°. Moreover, a twist angle of 8.6° is observed for the phenanthrene ring. 7b exhibits 2D “brick-wall” lamellar stacking arrangement, and the π–π stacking distances are 3.40 and 3.43 Å. Compounds 8a and 8b, featuring planar geometry, display herringbone packing. Notably, 8b exhibits a smaller π–π overlap than 7b, whose crystal packing mode is influenced by the seven-membered ring (Fig. 2e and f). As a common approach for regulating the intermolecular interactions, the formation of intermolecular C–H⋯O hydrogen bonds were accomplished by oxidizing the sulfur atoms to sulfone groups.

**Fig. 3:**
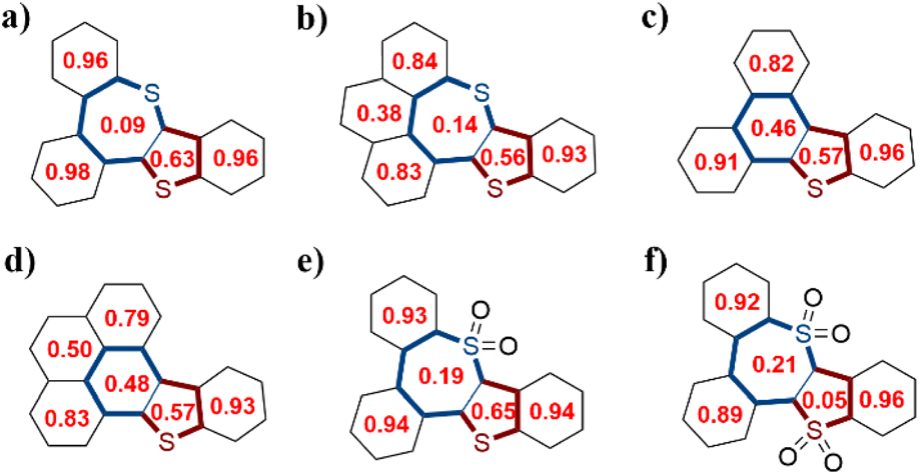
The calculated HOMA values of (a) 7a, (b) 7b, (c) 8a, (d) 8b, (e) 9, and (f) 10.

**Fig. 4:**
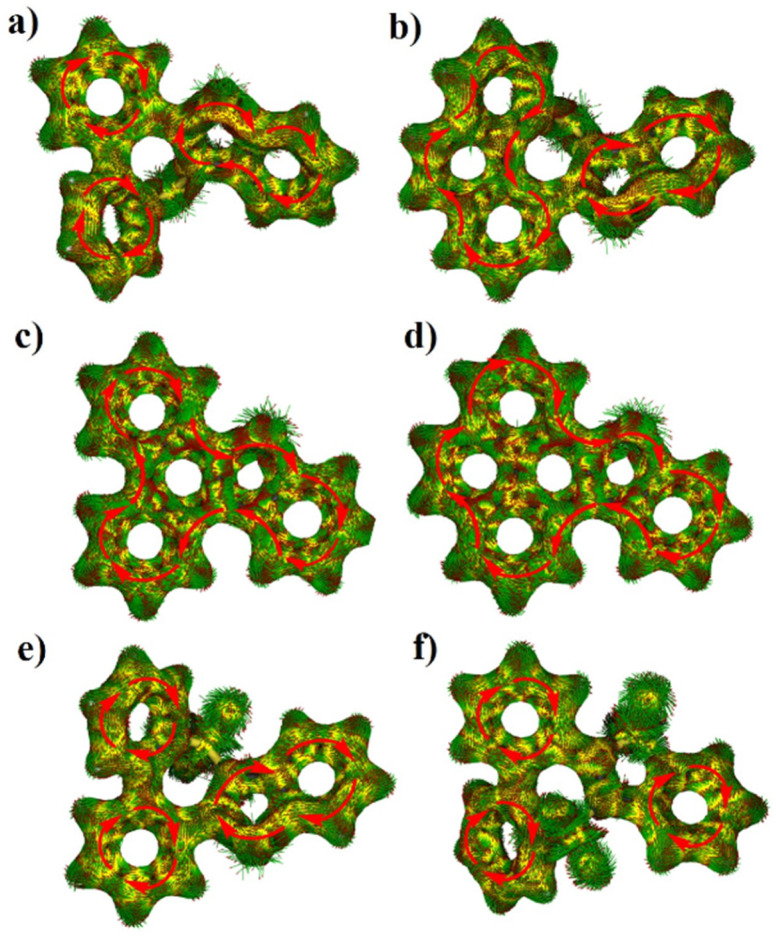
Calculated ACID plots of (a) 7a, (b) 7b, (c) 8a, (d) 8b, (e) 9, and (f) 10. The red arrows indicate the ring current flow.

To gain insights into the aromaticity, the Harmonic oscillator model of aromaticity (HOMA) values and anisotropy of the induced current density (ACID) were calculated (Fig. 3 and 4). The benzene rings and the five-membered sulfur-containing thiophene rings in compounds 7a, 7b, 8a, 8b, 9, and 10 display positive HOMA values, thereby indicating their aromatic properties. Meanwhile, the chalcogen-containing seven-membered rings in 7a and 7b possess relatively small positive HOMA values of 0.09 and 0.14, respectively, suggesting weak aromatic characteristics. Upon the oxidation of the sulfur atom within the seven-membered ring to a sulfone group, the aromaticity of thiepine is enhanced. Conversely, when the sulfur atom in the five-membered ring is oxidized to sulfone, the aromaticity of thiophene diminishes. ACID plots of 8a and 8b show a clockwise ring current along the periphery of the whole backbone, indicating the global aromatic characteristics, while weaker diatropic ring current were observed on sulfur-embedded seven-membered rings of 7a, 7b, 9, and 10.

The HOMOs and the LUMOs of these sulfur-embedded polycyclic aromatics are shown in Tables S22 and S23. Compounds 8a and 8b exhibit narrower bandgaps compared to 7a and 7b, respectively, consistent with the results of photophysical measurements. This can be attributed to the disruption of conjugation caused by the sulfur-containing seven-membered ring. The introduction of sulfone results in deeper LUMOs of −1.71 eV (9) and −2.62 eV (10). The electrostatic potential maps are presented in Fig. S9. For compounds 7a and 7b, the electron density is concentrated on the sulfur-containing seven-membered rings. In contrast, compounds 8a and 8b show a more delocalized electron distribution across the conjugated framework. When the sulfur atoms are oxidized to sulfone groups, the π-cores of compounds 9 and 10 exhibit electron deficiency. The electron density is predominantly concentrated on the oxygen atoms, suggesting that these compounds are more prone to form intermolecular C–H⋯O hydrogen bonds.

Hirshfeld surface analysis was performed to quantitatively analyze the intermolecular interactions (Fig. S10–S12). The S⋯S interaction (2.4%) in 7a demonstrates the influence of the introduction of the sulfur-containing seven-membered ring on intermolecular interactions. The ratio of π–π interactions (C⋯C) of 7b (15.1%) and 8b (14.2%) is higher than that of 7a (5.0%) and 8a (11.3%), which can be attributed to the extended π-plane. The oxidation of sulfur atoms led to O⋯H interactions of 9 (18.4%) and 10 (36.2%), which are consistent with the results from the crystal structure analysis.

## Conclusions

In summary, the incorporation of sulfur-containing 5–7 ring topology into acenes has been successfully achieved *via* heteroannular-selective C–H/C–H oxidative cross-coupling of 2-substituted biaryls with heteroarenes followed by directing group-involved intramolecular cyclization. A diverse library of thioether- and selenoether-substituted biheteroaryl compounds can be efficiently synthesized, owing to the excellent regioselectivity and high functional group tolerance of this protocol. Under oxidation/cyclization conditions, the cleavage of the C sp^3^–S bond of biheteroaryl compounds delivers thiophene- and thiepine-fused polycyclic aromatics 7, while the cleavage of the C sp^2^–Se bond generates thiophene and benzene rings fused products 8. Although the introduction of the seven-membered thiepine ring compromises molecular planarity and aromaticity, the crystal structure of 7b exhibits a “brick-wall” lamellar stacking arrangement, whereas compound 8a, which possesses a planar geometry, adopts a herringbone packing motif. The selective oxidation of sulfur atoms from 7a to 9 and 10, achieved by adjusting the equivalents of the oxidant, reduces the electron density of the molecular framework and enables the formation of intermolecular C–H⋯O hydrogen bonds. Crystal structure analysis and DFT calculations indicate that heteroacenes with sulfur-embedded 5–7 ring topology have the potential as high-performance organic semiconductor materials.

## Author contributions

Z. L., C. Z., and G. Y. conceived and supervised the project. Z. H. and L. Y. designed and carried out the experiments, analyzed the data, and wrote the manuscript. M. Z., Y. Z., Z. Z., F. X., and X. D. provided valuable assistance with the synthesis of compounds. S. Y. and C. X. contributed to experimental testing and analysis. All authors discussed the results and commented on the manuscript.

## Conflicts of interest

There are no conflicts to declare.

## Supplementary Material

SC-017-D5SC08209A-s001

SC-017-D5SC08209A-s002

## Data Availability

CCDC 2281727 (4l), 2433068 (7a), 2475673 (7b), 2433061 (8a), 2433067 (8b), 2433062 (9) and 2433063 (10) contain the supplementary crystallographic data for this paper.^23*a*–*g*^ The data supporting this article have been included as part of the supplementary information (SI). Supplementary information: further information, including general experimental information, optimization studies, detailed experimental procedures, compound characterization data and NMR spectra of new compounds. See DOI: https://doi.org/10.1039/d5sc08209a.
