# Supplementary material for: Rh(iii)-catalyzed heteroannular-selective heteroarylation of biaryls: facile access to heteroacenes with sulfur-embedded 5–7 ring topology
Source: Chem Sci. 2026 Jan 3;17(9):4594–600. doi: 10.1039/d5sc08209a (PMC12794288; doi:10.1039/d5sc08209a)

Supplementary Information (SI) for Chemical Science.
This journal is © The Royal Society of Chemistry 2026

Supporting Information

Rh(III)-Catalyzed heteroannular-selective heteroarylation of biaryls: facile access to heteroacenes with sulfur-embedded 5-7 ring topology

Zhanhui He[a], Li Yang[a], Menghang Zhou[a], Yue Zhong[a], Zheng Liu[a,b,*], Ziao Zhang[a], Feiyang Xia[a], Xuezhe Deng[a], Shuang Yan[b], Cheng Xu[a], Cheng Zhang[c,*], Guodong Yin[a,*]

[a]*Hubei Key Laboratory of Pollutant Analysis and Reuse Technology, College of Chemistry and Chemical Engineering, Hubei Normal University, Huangshi 435002, P.R. China*
[b]*Medical Imaging Key Laboratory of Sichuan Province, North Sichuan Medical College, Nanchong 637000, P.R. China*
[c]*Key Laboratory of Green Chemistry and Technology of Ministry of Education, College of Chemistry, Sichuan University, 29 Wangjiang Road, Chengdu 610064, P.R. China*

Table of Contents

I. General remarks……………………..………………..…………………………..………..1
II. Synthesis of thioether- and selenoether-substituted biaryls……..........................2
III. Optimization of heteroannular-selective heteroarylation of biaryls..…….……...5
IV. Rh(III)-Catalyzed heteroannular-selective heteroarylation of biaryls…….........8
V. Scale-up synthesis……………..……………..…………………….…..…..….29
VI. General procedure for the recovery experiment of Ag salt………...…...…......30
VII. Mechanistic study....……………….…………………..….……………....………..31
VIII. Synthetic applications………..………………..…………………......………..38
IX. Photophysical properties…...………………….. …….….………………....………..41
X. Single crystal X-ray structure and crystallographic data……..........………..47
XI. Theoretical calculations……………….….……………………………....………..56
XII. References……………………………………..……………………....………..60
XIII. Copies of ^{1}H and ^{13}C{^{1}H} NMR spectra…………………………….……..61

I. General remarks

NMR spectra were recorded on Bruker Avance III^{TM} 300 MHz NMR spectrometers. The ^{1}H NMR (300 MHz) chemical shifts were measured relative to $CDCl_3$ or DMSO-d_6 as the internal reference (DMSO-d_6: δ = 2.50 ppm; $CDCl_3$: δ = 7.26 ppm). The ^{13}C NMR (75 MHz) chemical shifts were given using $CDCl_3$ or DMSO-d_6 as the internal standard (DMSO-d_6: δ = 39.52 ppm; $CDCl_3$: δ = 77.16 ppm). High-resolution mass spectra (HRMS) were obtained with a Shimadzu LCMS-ITTOF (ESI). The X-ray crystal structure determination was performed using a Bruker SMART APEX CCD system. The UV-vis absorption data were determined on a METASH UV spectrometer, and the PL emission data were determined on a Shimadzu RF-6000 fluorescence spectrometer.

All reagents were obtained from commercial suppliers and used without further purification unless otherwise stated. Phenylboronic acid derivatives and 2-bromothioanisole were purchased from Shanghai Bide Pharmaceutical Technology Co., Ltd., $RhCl_3$ and 1,2,3,4,5-pentamethylcyclopentadiene were purchased from Shanxi Kaida Chemical Engineering (China) CO., Ltd., Cp*Rh(MeCN)$_3$[SbF$_6$]$_2$ was prepared according to literature methods,[1] *n*-Butyllithium, solvents and inorganic salt were purchased from Shanghai Energy Chemical Co., Ltd. Thioether- and selenoether-substituted biaryls were prepared according to the literatures.[2–5]

II. Synthesis of thioether- and selenoether-substituted biaryls

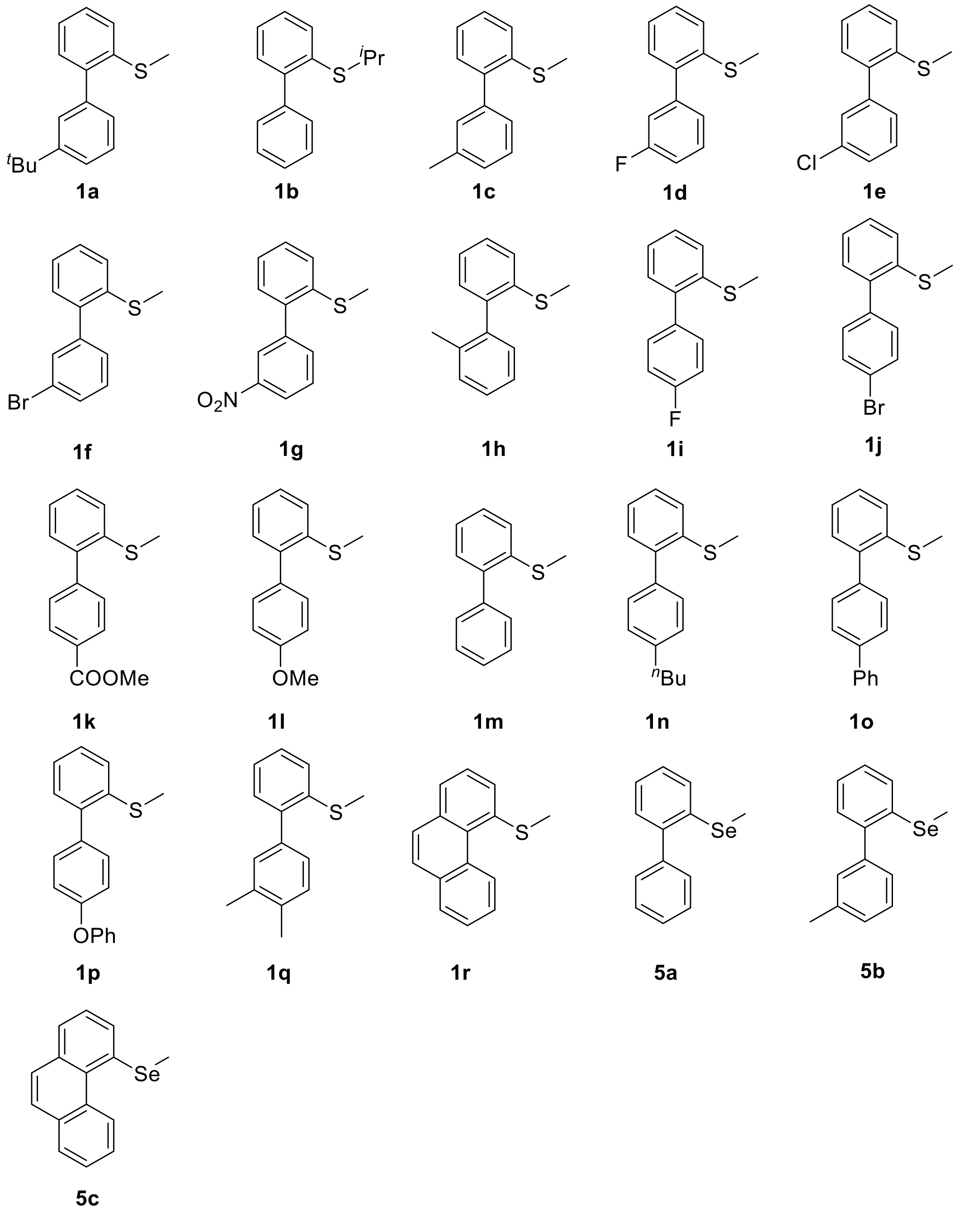

Scheme S1 List of thioether- and selenoether-substituted biaryls.

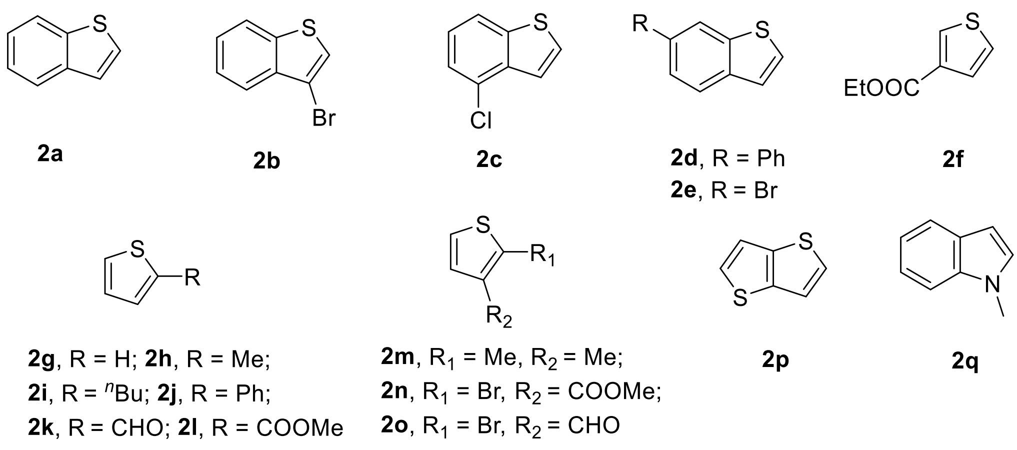

Scheme S2 List of heteroarenes.

The procedure for the synthesis of the thioether- and selenoether-substituted biaryls were prepared according to the literatures[2–5] except **1r** and **5c**.

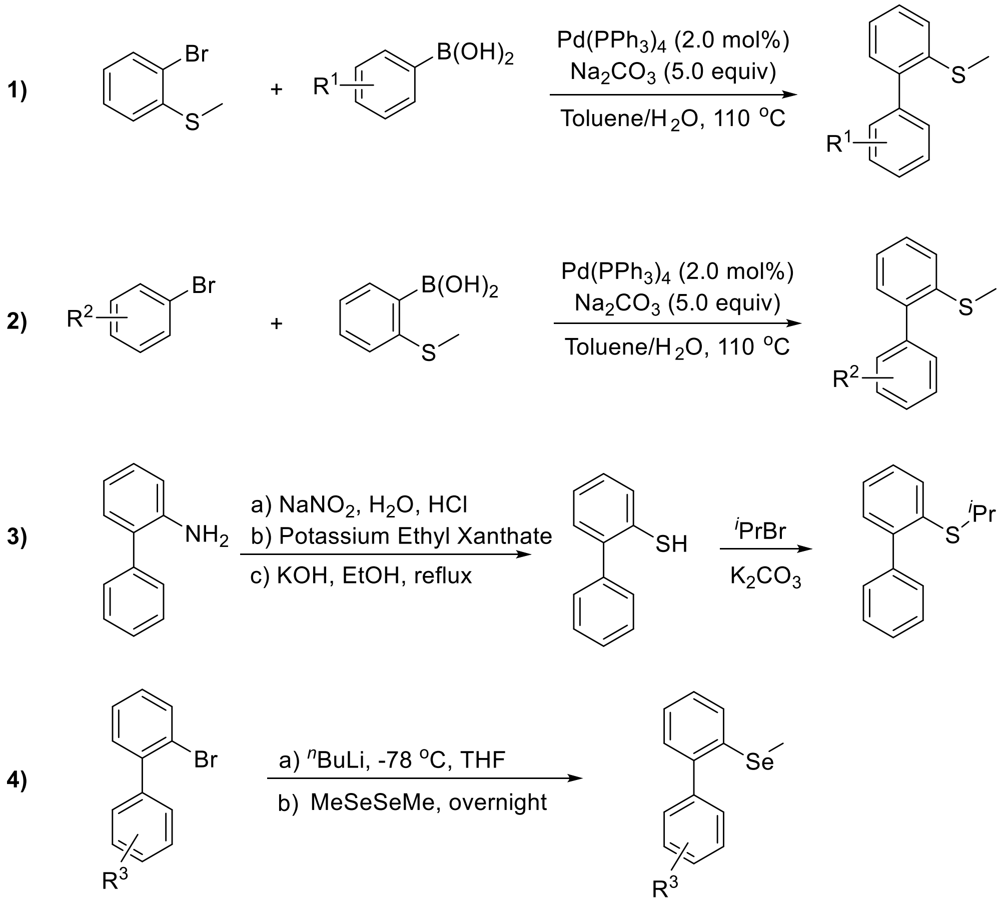

Scheme S3 The general procedure for synthesis of the thioether- and selenoether-substituted biaryls.

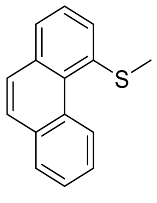

Methyl(phenanthren-4-yl)sulfane (1r)

To a 100 mL two-neck round-bottom flask was added 4-bromophenanthrene[5] (5.0 mmol, 1.0 equiv) and THF (40 mL). The solution was cooled to -78 °C and ^{n}BuLi (2.5 mol/L in THF, 5.5 mmol, 1.1 equiv) was added dropwise. After stirring for 0.5 hours at the temperature, dimethyl disulfide (5.5 mmol, 1.1 equiv) in THF (5 mL) was added. The mixture was stirred at room temperature overnight, and then saturated aqueous NH_4Cl solution was added. The organic layer was separated, washed with distilled water and aqueous $NaHCO_3$ solution, dried over Na_2SO_4, and concentrated in vacuo, the residue was purified by flash column chromatography to afford the desired product. Product obtained as a yellow liquid (0.84 g, 75% yield). ^{1}H NMR (300 MHz, $CDCl_3$): δ = 9.48 (d, J = 8.4 Hz, 1H), 7.69 (d, J = 7.5 Hz, 1H), 7.53-7.38 (m, 6H), 7.29 (t, J = 7.5 Hz, 1H), 2.35 (s, 3H) ppm; ^{13}C{^{1}H} NMR (75 MHz, $CDCl_3$): δ = 136.8, 134.0, 133.3, 130.5, 129.5, 128.4, 128.1, 127.7, 126.7, 126.6, 126.4, 125.9, 125.6, 18.8 ppm. HRMS (ESI$^+$): calcd for $C_{15}H_{13}S^+$: [M+H]$^+$, 225.0733, found: 225.0738.

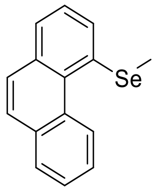

Methyl(phenanthren-4-yl)selane (5c)

To a 100 mL two-neck round-bottom flask was added 4-bromophenanthrene (5.0 mmol, 1.0 equiv) and THF (40 mL). The solution was cooled to -78 °C and ^{n}BuLi (2.5 mol/L in THF, 5.5 mmol, 1.1 equiv) was added dropwise. After stirring for 0.5 hours at the temperature, dimethyl diselenide (5.5 mmol, 1.1 equiv) in THF (5 mL) was added. The mixture was stirred at room temperature overnight, and then saturated aqueous NH_4Cl solution was added. The organic layer was separated, washed with distilled water and aqueous $NaHCO_3$ solution, dried over Na_2SO_4, and concentrated in vacuo, the residue was purified by flash column chromatography to afford the desired product. Product

obtained as a yellow liquid (1.03 g, 76% yield). ^{1}H NMR (300 MHz, $CDCl_3$): δ = 9.22 (d, J = 8.4 Hz, 1H), 7.95 (d, J = 8.7 Hz, 1H), 7.69-7.68 (m, 6H), 7.52 (t, J = 7.5 Hz, 1H), 2.46 (s, 3H) ppm; ^{13}C{^{1}H} NMR (75 MHz, $CDCl_3$): δ = 133.9, 133.3, 131.4, 130.9, 130.7, 129.2, 128.4, 127.6, 127.5, 127.4, 126.9, 126.6, 126.3, 125.4, 10.8 ppm. HRMS (ESI$^+$): calcd for $C_{15}H_{12}SeK^+$: [M+K]$^+$, 310.9736, found: 310.9741.

III. Optimization of heteroannular-selective heteroarylation of biaryls

A 25 mL Schlenk tube with a magnetic stir bar was charged with (3'-(*tert*-butyl)-[1,1'-biphenyl]-2-yl)(methyl)sulfane (**1a**, 0.2 mmol), benzo[*b*]thiophene (**2a**, 0.6 mmol), Catalyst, Oxidant, Additive and Solvent (1.0 mL) under N_2 atmosphere. The resulting mixture was stirred at 120 ºC for 24 h and then diluted with 10 mL of CH_2Cl_2. The solution was filtered through a celite pad and washed with 10-25 mL of CH_2Cl_2. The filtrate was concentrated under vacuum and the residue was purified by column chromatography on Silica gel column (Petroleum ether/CH_2Cl_2 = 10/1, v/v) to provide the desired product.

Table S1 The screening of solvent.

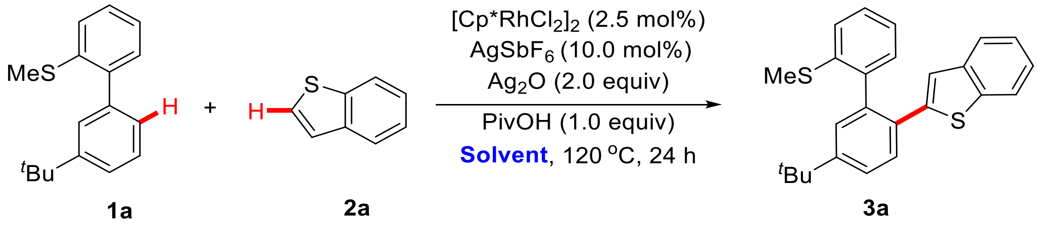

Entry	Solvent	Yield[a]	Entry	Solvent	Yield[a]
1	THF	35	6	DMF	Trace
2	1,4-dioxane	28	7	DMSO	N.D.
3	Toluene	Trace	8	TFE	26
4	PhCl	32	9	^{t}BuOH	32
**5**	**DCE**	**41**	10	TFE	26
6	CH_2Cl_2	28	12	HFIP	Trace

Reaction conditions: **1a** (0.2 mmol, 1.0 equiv), **2a** (0.6 mmol, 3.0 equiv), $[Cp^*RhCl_2]_2$ (2.5 mol%), $AgSbF_6$ (10.0 mol%), Ag_2O (0.4 mmol, 2.0 equiv) and PivOH (0.2 mmol,

1.0 equiv) in Solvent (1.0 mL) at 120 °C for 24 h under N_2 atmosphere. N.D.: not detected. [a]. Yield of isolated products.

Table S2 The screening of catalyst.

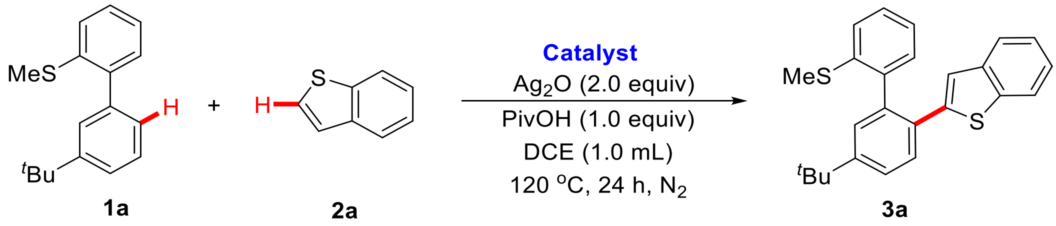

Entry	Catalyst	Yield[a]	Entry	Catalyst	Yield[a]
1	–	N.D.	8	$RhCl_3 \cdot 3H_2O$	23
2	CuI	N.D.	9	$[Cp^*RhCl_2]_2/AgSbF_6$	41
3	[Ru(*p*-cymene)$Cl_2]_2$	trace	10	$[Cp^*RhCl_2]_2/AgBF_4$	34
4	$Pd(OAc)_2$	27	11	$[Cp^*RhCl_2]_2$/AgOTf	34
5	$[Cp^*IrCl_2]_2/AgSbF_6$	18	15[b]	$Cp^*Rh(MeCN)_3[SbF_6]_2$	48
6	$[Rh(coe)_2Cl]_2$	31	**13[c]**	**$Cp^*Rh(MeCN)_3[SbF_6]_2$**	**59**
7	$[Cp^*RhCl_2]_2$	32	14[d]	$Cp^*Rh(MeCN)_3[SbF_6]_2$	40

Reaction conditions: **1a** (0.2 mmol, 1.0 equiv), **2a** (0.6 mmol, 3.0 equiv), Catalyst (5 mol%), Ag_2O (0.4 mmol, 2.0 equiv) and PivOH (0.2 mmol, 1.0 equiv) in DCE (1.0 mL) at 120 °C for 24 h under N_2 atmosphere. N.D.: not detected. [a]. Yield of isolated products. [b]. $Cp^*Rh(MeCN)_3[SbF_6]_2$ (5.0 mol%) was used, [c]. $Cp^*Rh(MeCN)_3[SbF_6]_2$ (3.0 mol%) was used, [d]. $Cp^*Rh(MeCN)_3[SbF_6]_2$ (1.0 mol%) was used.

Table S3 The screening of oxidant.

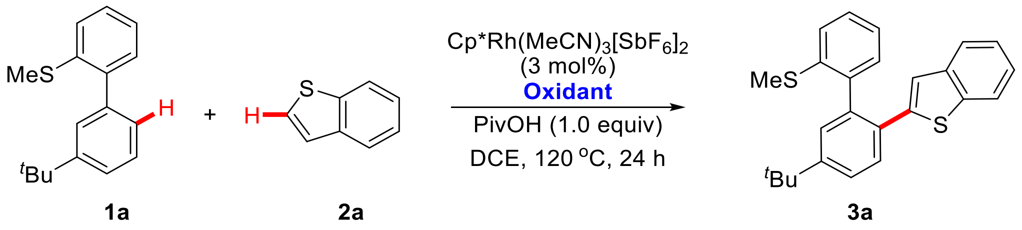

Entry	Oxidant	Yield[a]	Entry	Oxidant	Yield[a]
1	–	N.D.	7	Ag_2CO_3	51
2	$K_2S_2O_8$	N.D.	8	AgOAc	43
3	1,4-BQ	N.D.	9	AgOTFA	Trace
4	$PhI(OAc)_2$	Trace	10	AgF	48

5	$Cu(OAc)_2 \cdot H_2O$	Trace	11[b]	Ag_2O	62
6	Ag_2O	59	**12[c]**	**Ag_2O**	**68**

Reaction conditions: **1a** (0.2 mmol, 1.0 equiv), **2a** (0.6 mmol, 3.0 equiv), Cp*Rh(MeCN)$_3$[SbF$_6$]$_2$ (3.0 mol%), Oxidant (0.4 mmol, 2.0 equiv) and PivOH (0.2 mmol, 1.0 equiv) in DCE (1.0 mL) at 120 °C for 24 h under N_2 atmosphere. N.D.: not detected. [a]. Yield of isolated products. [b]. Ag_2O (0.5 mmol, 2.5 equiv) was used. [c]. Ag_2O (0.7 mmol, 3.5 equiv) was used.

Table S4 The screening of additive.

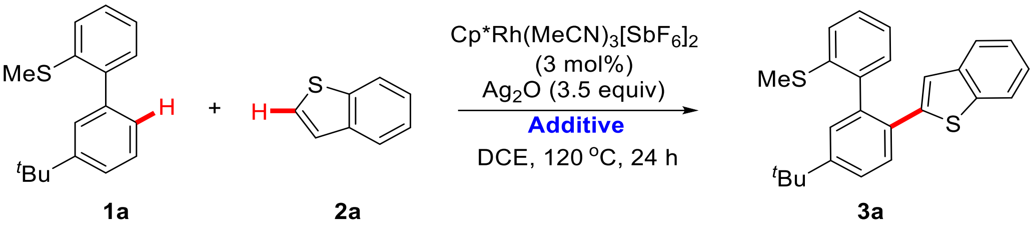

Entry	Additive	Yield[a]	Entry	Additive	Yield[a]
1	–	Trace	6	HOAc	42
2	K_2CO_3	N.D.	7	TfOH	38
3	NaOAc	29	8	MesCOOH	45
4	K_2HPO_4	N.D.	9	AdCOOH	46
5	DBU	N.D.	**10[b]**	**PivOH**	**76**

Reaction conditions: **1a** (0.2 mmol, 1.0 equiv), **2a** (0.6 mmol, 3.0 equiv), Cp*Rh(MeCN)$_3$[SbF$_6$]$_2$ (3.0 mol%), Ag_2O (0.7 mmol, 3.5 equiv) and Additive (0.2 mmol, 1.0 equiv) in DCE (1.0 mL) at 120 °C for 24 h under N_2 atmosphere. N.D.: not detected. [a]. Yield of isolated products. [b]. PivOH (2.0 equiv) was used.

Table S5 The screening of reaction time and temperature.

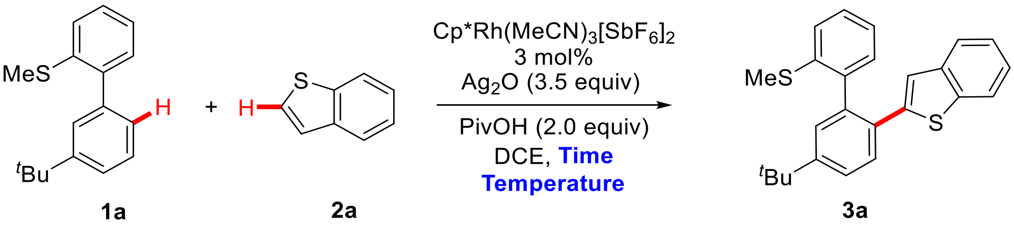

Entry	Temperature	Time	Yield[a]	Entry	Temperature	Time	Yield[a]
1	110	24	68	6	120	30	73
**2**	**120**	**24**	**76**	7[b]	120	24	69
3	130	24	68	8[c]	120	24	59

4	140	24	56	9[d]	120	24	71
5	120	18	62	10[e]	120	24	64

Reaction conditions: **1a** (0.2 mmol, 1.0 equiv), **2a** (0.6 mmol, 3.0 equiv), $Cp^*Rh(MeCN)_3[SbF_6]_2$ (3.0 mol%), Ag_2O (0.7 mmol, 3.5 equiv) and PivOH (0.4 mmol, 2.0 equiv) in DCE (1.0 mL) under N_2 atmosphere. [a]. Yield of isolated products. [b]. **2a** (0.5 mmol, 2.5 equiv) was used. [c]. **2a** (0.4 mmol, 2.0 equiv) was used. [d]. Ag_2O (0.6 mmol, 3.0 equiv) was used. [e]. Ag_2O (0.5 mmol, 2.5 equiv) was used.

IV. Rh(III)-Catalyzed heteroannular-selective heteroarylation of biaryls

A 25 mL Schlenk tube with a magnetic stir bar was charged with (3'-(*tert*-butyl)-[1,1'-biphenyl]-2-yl)(methyl)sulfane **1a** (0.2 mmol, 1.0 equiv), heteroarenes **2** (0.6 mmol, 3.0 equiv), $(Cp^*Rh(MeCN)_3[SbF_6]_2$ (5.3 mg, 3 mol%), Ag_2O (162.2 mg, 0.7 mmol), PivOH (40.8 mg, 0.4 mmol) and DCE (1.0 mL) under N_2 atmosphere. The resulting mixture was stirred at 120 ºC for 24 h and then diluted with 10 mL of CH_2Cl_2. The solution was filtered through a celite pad and washed with 10-25 mL of CH_2Cl_2. The filtrate was concentrated under vacuum and the residue was purified by column chromatography on silica gel to provide the desired product **3**, **4** or **6**.

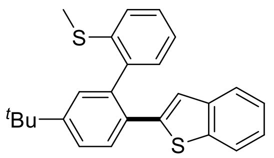

2-(5-(*tert*-Butyl)-2'-(methylthio)-[1,1'-biphenyl]-2-yl)benzo[*b*]thiophene (3a)

Purification by column chromatography on silica gel (Petroleum ether/CH_2Cl_2 = 15/1, v/v) afforded the desired product **3a** as a yellow solid (59.1 mg, 76%). ^1H NMR (300 MHz, $CDCl_3$): δ = 7.70-7.65 (m, 2H), 7.58-7.55 (m, 1H), 7.48 (dd, J_1 = 8.1 Hz, J_2 = 2.1 Hz, 1H), 7.36-7.31 (m, 2H), 7.23-7.29 (m, 5H), 6.91 (s, 1H), 2.31 (s, 3H), 1.38 (s, 9H) ppm; $^{13}C\{^1H\}$ NMR (75 MHz, $CDCl_3$): δ = 151.0, 143.5, 140.4, 140.3, 140.2, 138.7, 138.5, 130.8, 130.7, 129.9, 128.7, 128.4, 125.3, 125.2, 124.7, 124.0, 123.8, 123.5, 122.5, 122.0, 34.9, 31.4, 16.0 ppm. HRMS (ESI^+): calcd for $C_{25}H_{25}S_2^+$: $[M+H]^+$ 389.1393, found: 389.1392.

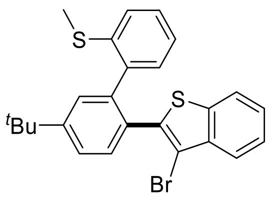

3-Bromo-2-(5-(*tert*-butyl)-2'-(methylthio)-[1,1'-biphenyl]-2-yl)benzo[*b*]thiophene (3b)

Purification by column chromatography on silica gel (Petroleum ether/CH_2Cl_2 = 10/1, v/v) afforded the desired product **3b** as a yellow solid (37.4 mg, 40%). ^{1}H NMR (300 MHz, $CDCl_3$): δ = 7.77 (d, J= 8.1 Hz, 1H), 7.63-7.56 (m, 2H), 7.52-7.48 (m, 2H), 7.40 (d, J= 8.1 Hz, 1H), 7.34 (d, J= 13.8 Hz, 1H), 7.29-7.26 (m, 1H), 7.21-7.19 (m, 2H), 7.08 (d, J= 6.9 Hz, 1H), 7.02-6.95 (m, 1H), 2.37 (s, 3H), 1.40 (s, 9H) ppm; ^{13}C{^{1}H} NMR (75 MHz, $CDCl_3$): δ = 151.8, 140.1, 139.8, 138.9, 138.6, 138.4, 138.3, 131.8, 130.6, 129.2, 128.6, 128.1, 127.9, 125.3, 125.0, 124.8, 124.4, 123.4, 122.2, 107.3, 35.0, 31.4, 16.3 ppm. HRMS (ESI$^+$): calcd for $C_{25}H_{24}{}^{79}BrS_2{}^+$: [M+H]$^+$, 467.0498, found: 467.0500; HRMS (ESI$^+$): calcd for $C_{25}H_{24}{}^{81}BrS_2{}^+$: [M+H]$^+$, 469.0477, found:469.0471.

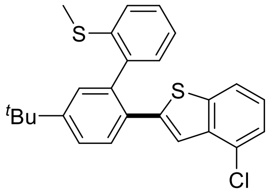

2-(5-(*tert*-Butyl)-2'-(methylthio)-[1,1'-biphenyl]-2-yl)-4-chlorobenzo[*b*]thiophene (3c)

Purification by column chromatography on silica gel (Petroleum ether/CH_2Cl_2 = 10/1, v/v) afforded the desired product **3c** as a yellow solid (70.4 mg, 83%). ^{1}H NMR (300 MHz, $CDCl_3$): δ = 7.68 (d, J= 8.1 Hz, 1H), 7.54-7.47 (m, 2H), 7.37-7.33 (m, 2H), 7.22 (d, J = 6.9 Hz, 2H), 7.15-7.07 (m, 4H), 2.30 (s, 3H), 1.38 (s, 9H) ppm; ^{13}C{^{1}H} NMR (75 MHz, $CDCl_3$): δ = 151.5, 144.5, 141.4, 140.0, 138.8, 138.6, 138.3, 130.6, 130.3, 129.8, 128.64, 128.56, 128.3, 125.4, 125.2, 124.8, 124.3, 124.0, 120.7, 120.5, 34.9, 31.4, 15.9 ppm. HRMS (ESI$^+$): calcd for $C_{25}H_{24}{}^{35}ClS_2{}^+$: [M+H]$^+$, 423.1003, found: 423.1001 ppm; HRMS (ESI$^+$): calcd for $C_{25}H_{24}{}^{37}ClS_2{}^+$: [M+H]$^+$, 425.0973, found: 425.0976.

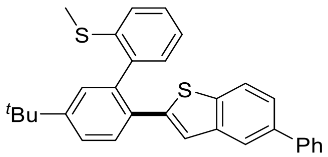

2-(5-(*tert*-Butyl)-2'-(methylthio)-[1,1'-biphenyl]-2-yl)-5-phenylbenzo[*b*]thiophene (3d)

Purification by column chromatography on silica gel (Petroleum ether/CH_2Cl_2 = 10/1, v/v) afforded the desired product **3d** as a yellow solid (59.4 mg, 64%). ^{1}H NMR (300 MHz, $CDCl_3$): δ = 7.77-7.67 (m, 3H), 7.59 (d, J = 7.5 Hz, 2H), 7.51-7.42 (m, 4H), 7.40-7.37 (m, 2H), 7.34-7.32 (m, 2H), 7.17-7.10 (m, 2H), 6.93 (s, 1H), 2.31 (s, 3H), 1.38 (s, 9H) ppm; ^{13}C{^{1}H} NMR (75 MHz, $CDCl_3$): δ = 151.1, 144.2, 141.5, 140.7, 140.2, 139.3, 138.7, 138.5, 137.5, 130.7, 130.6, 129.9, 128.9, 128.7, 128.4, 127.4, 127.1, 125.3, 125.2, 124.7, 124.30, 124.25, 123.7, 123.5, 122.7, 122.3, 121.9, 34.9, 31.4, 15.9 ppm. HRMS (ESI$^+$): calcd for $C_{31}H_{29}S_2^+$: [M+H]$^+$, 465.1706, found: 465.1704.

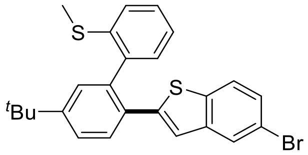

5-Bromo-2-(5-(*tert*-butyl)-2'-(methylthio)-[1,1'-biphenyl]-2-yl)benzo[*b*]thiophene (3e)

Purification by column chromatography on silica gel (Petroleum ether/CH_2Cl_2 = 10/1, v/v) afforded the desired product **3e** as a yellow solid (72.7 mg, 78%). ^{1}H NMR (300 MHz, $CDCl_3$): δ = 7.67 (d, J = 1.8 Hz, 1H), 7.63 (d, J = 8.1 Hz, 1H), 7.52-7.46 (m, 2H), 7.35-7.31 (m, 2H), 7.27 (dd, J_1 = 8.7 Hz, J_2 = 2.1 Hz, 1H), 7.22 (d, J = 7.8 Hz 1H), 7.13-7.09 (m, 2H), 6.80 (s, 1H), 2.28 (s, 3H), 1.37 (s, 9H) ppm; ^{13}C{^{1}H} NMR (75 MHz, $CDCl_3$): δ = 151.5, 145.4, 141.7, 140.1, 138.8, 138.7, 138.6, 130.6, 130.3, 129.9, 128.7, 128.5, 126.8, 126.0, 125.4, 125.1, 124.7, 123.4, 121.6, 118.0, 34.9, 31.4, 15.9 ppm. HRMS (ESI$^+$): calcd for $C_{25}H_{24}{}^{79}BrS_2^+$: [M+H]$^+$, 467.0498, found: 467.0500 ppm; HRMS (ESI$^+$): calcd for $C_{25}H_{24}{}^{81}BrS_2^+$: [M+H]$^+$, 469.0477, found: 469.0471.

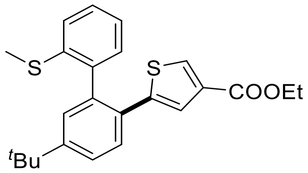

Ethyl 5-(5-(*tert*-butyl)-2'-(methylthio)-[1,1'-biphenyl]-2-yl)thiophene-3-carboxylate (3f)

Purification by column chromatography on silica gel (Petroleum ether/CH_2Cl_2 = 2/1, v/v) afforded the desired product **3f** as a yellow solid (49.3 mg, 60%). ^{1}H NMR (300 MHz, $CDCl_3$): δ = 7.83 (d, J = 1.5 Hz, 1H), 7.58 (d, J = 8.4 Hz, 1H), 7.45 (dd, J_1 = 2.1 Hz, J_2 = 8.1 Hz, 1H), 7.36 (d, J = 6.0 Hz, 1H), 7.31 (d, J = 2.1 Hz, 1H), 7.27 (d, J = 1.2 Hz, 1H), 7.23 (d, J = 7.8 Hz, 1H), 7.17-7.09 (m, 2H), 4.25 (q, J = 7.2 Hz, 2H), 2.31 (s, 3H), 1.36 (s, 9H), 1.32 (t, J = 7.2 Hz, 3H) ppm; ^{13}C{^{1}H} NMR (75 MHz, $CDCl_3$): δ = 163.0, 151.0, 143.7, 140.0, 139.0, 138.1, 133.1, 132.5, 130.7, 130.0, 129.1, 128.6, 128.4, 126.2, 125.4, 125.1, 124.7, 60.6, 34.8, 31.4, 15.9, 14.5 ppm. HRMS (ESI$^+$): calcd for $C_{24}H_{27}O_2S_2^+$: [M+Na]$^+$, 433.1267, found: 433.1266.

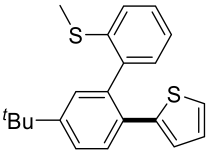

2-(5-(*tert*-Butyl)-2'-(methylthio)-[1,1'-biphenyl]-2-yl)thiophene (3g)

Purification by column chromatography on silica gel (Petroleum ether/CH_2Cl_2 = 10/1, v/v) afforded the desired product **3g** as a yellow solid (22.3 mg, 33%). ^{1}H NMR (300 MHz, $CDCl_3$): δ = 7.58 (d, J = 8.4 Hz, 1H), 7.44 (dd, J_1 = 8.1 Hz, J_2 = 2.1 Hz, 1H), 7.36-7.30 (m, 2H), 7.25 (d, J = 6.0 Hz, 1H), 7.16-7.09 (m, 3H), 6.83-6.81 (m, 1H), 6.72-6.71 (m, 1H), 2.29 (s, 3H), 1.36 (s, 9H) ppm; ^{13}C{^{1}H} NMR (75 MHz, $CDCl_3$): δ = 150.3, 143.1, 140.9, 138.8, 138.1, 130.9, 130.7, 129.4, 128.5, 128.2, 126.8, 125.9, 125.5, 125.2, 124.7, 34.8, 31.4, 16.0 ppm. HRMS (ESI$^+$): calcd for $C_{21}H_{23}S_2^+$: [M+H]$^+$, 339.1236, found: 339.1239.

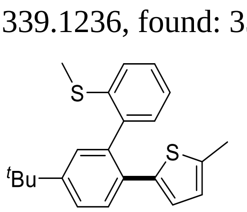

2-(5-(*tert*-Butyl)-2'-(methylthio)-[1,1'-biphenyl]-2-yl)-5-methylthiophene (3h)

Purification by column chromatography on silica gel (Petroleum ether/CH_2Cl_2 = 10/1, v/v) afforded the desired product **3h** as a yellow solid (49.9 mg, 71%). ^{1}H NMR (300 MHz, $CDCl_3$): δ = 7.53 (d, J = 8.1 Hz, 1H), 7.41 (dd, J_1 = 8.1 Hz, J_2 = 2.1 Hz, 1H), 7.35-7.31 (m, 1H), 7.28-7.23 (m, 2H), 7.12 (d, J = 3.6, 2H), 6.46 (s, 2H), 2.36 (s, 3H),

2.31 (s, 3H), 1.34 (s, 9H) ppm; $^{13}C\{^1H\}$ NMR (75 MHz, $CDCl_3$): δ = 149.8, 140.9, 140.6, 139.6, 138.7, 137.6, 131.1, 130.6, 129.0, 128.5, 128.1, 125.8, 125.3, 125.1, 124.7, 34.7, 31.4, 16.0, 15.4 ppm. HRMS (ESI^+): calcd for $C_{22}H_{25}S_2^+$: $[M+H]^+$, 353.1393, found: 353.1391.

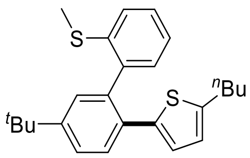

2-Butyl-5-(5-(*tert*-butyl)-2'-(methylthio)-[1,1'-biphenyl]-2-yl)thiophene (3i)

Purification by column chromatography on silica gel (Petroleum ether/CH_2Cl_2 = 10/1, v/v) afforded the desired product **3i** as a yellow solid (47.3 mg, 60%). ^1H NMR (300 MHz, $CDCl_3$): δ = 7.55 (d, J = 8.4 Hz, 1H), 7.41 (dd, J_1 = 8.4 Hz, J_2 = 2.1 Hz, 1H), 7.34-7.30 (m, 1H), 7.28 (d, J = 2.1 Hz, 1H), 7.25 (d, J = 6.9 Hz, 1H), 7.12 (d, J = 3.9 Hz, 2H), 6.46 (q, J = 3.6 Hz, 2H), 2.68 (t, J = 7.5 Hz, 2H), 2.29 (s, 3H), 1.54 (q, J = 7.8 Hz, 2H), 1.35 (s, 9H), 1.30 (t, J = 7.5 Hz, 2H), 0.89 (t, J = 7.5 Hz, 3H) ppm; $^{13}C\{^1H\}$ NMR (75 MHz, $CDCl_3$): δ = 149.7, 145.6, 141.0, 140.3, 138.7, 137.6, 131.1, 130.6, 129.0, 128.4, 128.1, 125.5, 125.3, 125.1, 124.7, 124.0, 34.7, 33.7, 31.4, 29.8, 22.2, 16.0, 14.0 ppm. HRMS (ESI^+): calcd for $C_{25}H_{31}S_2^+$: $[M+H]^+$, 395.1862, found: 395.1861.

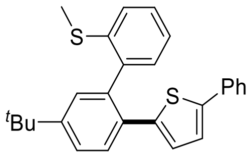

2-(5-(*tert*-Butyl)-2'-(methylthio)-[1,1'-biphenyl]-2-yl)-5-phenylthiophene (3j)

Purification by column chromatography on silica gel (Petroleum ether/CH_2Cl_2 = 10/1, v/v) afforded the desired product **3j** as a yellow solid (52.2 mg, 63%). ^1H NMR (300 MHz, $CDCl_3$): δ = 7.60 (d, J = 8.1 Hz, 1H), 7.47-7.41 (m, 3H), 7.36-7.29 (m, 3H), 7.26-7.18 (m, 3H), 7.16-7.13 (m, 2H), 7.01 (d, J = 3.6 Hz, 1H), 6.60 (d, J = 3.9 Hz, 1H), 2.28 (s, 3H), 1.35 (s, 9H) ppm; $^{13}C\{^1H\}$ NMR (75 MHz, $CDCl_3$): δ = 150.4, 143.5, 142.5, 140.6, 138.7, 137.8, 134.6, 130.7, 130.6, 129.0, 128.9, 128.6, 128.3, 127.3, 126.9, 125.6, 125.3, 124.7, 123.1, 34.8, 31.4, 15.9 ppm. HRMS (ESI^+): calcd for $C_{27}H_{27}S_2^+$: $[M+H]^+$, 415.1549, found: 415.1550.

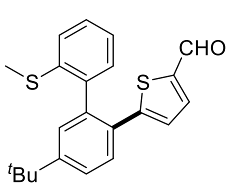

5-(5-(*tert*-Butyl)-2'-(methylthio)-[1,1'-biphenyl]-2-yl)thiophene-2-carbaldehyde (3k)

Purification by column chromatography on silica gel (Petroleum ether/CH_2Cl_2 = 2/1, v/v) afforded the desired product **3k** as a yellow solid (38.1 mg, 52%). ^1H NMR (300 MHz, $CDCl_3$): δ = 9.75 (s, 1H), 7.60 (d, J = 8.4 Hz, 1H), 7.50-7.47 (m, 2H), 7.38-7.33 (m, 2H), 7.23 (d, J = 7.2, 1H), 7.17-7.09 (m, 2H), 6.78 (d, J = 3.9 Hz, 1H) 2.31 (s, 3H), 1.37 (s, 9H) ppm; $^{13}C\{^1H\}$ NMR (75 MHz, $CDCl_3$): δ = 183.0, 153.7, 152.3, 142.6, 139.6, 138.6, 138.5, 136.5, 130.5, 129.7, 129.5, 128.8, 128.7, 127.1, 125.5, 125.1, 124.8, 35.0, 31.3, 15.7 ppm. HRMS (ESI$^+$): calcd for $C_{22}H_{23}OS_2^+$: [M+H]$^+$, 367.1185, found: 367.1182.

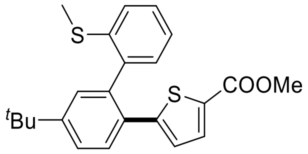

Methyl 5-(5-(*tert*-butyl)-2'-(methylthio)-[1,1'-biphenyl]-2-yl)thiophene-2-carboxylate (3l)

Purification by column chromatography on silica gel (Petroleum ether/CH_2Cl_2 = 1/1, v/v) afforded the desired product **3l** as a yellow solid (58.7 mg, 74%). ^1H NMR (300 MHz, $CDCl_3$): δ = 7.49 (d, J = 8.1 Hz, 1H), 7.42-7.36 (m, 2H), 7.29-7.23 (m, 2H), 7.16-7.13 (m, 1H), 7.08-7.01 (m, 2H), 6.54 (d, J = 3.9 Hz, 1H), 3.73 (s, 3H), 2.22 (s, 3H), 1.28 (s, 9H) ppm; $^{13}C\{^1H\}$ NMR (75 MHz, $CDCl_3$): δ = 162.9, 151.6, 150.5, 139.8, 138.5, 138.3, 133.4, 132.1, 130.4, 129.9, 129.5, 128.7, 128.5, 126.7, 125.4, 125.1, 124.7, 52.1, 34.9, 31.3, 15.8 ppm. HRMS (ESI$^+$): calcd for $C_{23}H_{25}O_2S_2^+$: [M+H]$^+$, 397.1291, found: 397.1280.

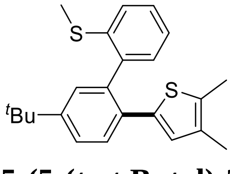

5-(5-(*tert*-Butyl)-2'-(methylthio)-[1,1'-biphenyl]-2-yl)-2,3-dimethylthiophene (3m)

Purification by column chromatography on silica gel (Petroleum ether/CH_2Cl_2 = 10/1, v/v) afforded the desired product **3m** as a yellow solid (53.5 mg, 73%). ^{1}H NMR (300 MHz, $CDCl_3$): δ = 7.45 (d, J = 8.1 Hz, 1H), 7.33-7.30 (m, 1H), 7.24-7.22 (m, 1H), 7.18-7.15 (m, 2H), 7.05 (d, J = 4.5 Hz, 2H), 6.32 (s, 1H), 2.22 (s, 3H), 2.11 (s, 3H), 1.88 (s, 3H), 1.27 (s, 9H) ppm; $^{13}C\{^1H\}$ NMR (75 MHz, $CDCl_3$): δ = 149.5, 140.9, 138.7, 137.8, 137.4, 132.9, 132.8, 131.0, 130.6, 128.8, 128.7, 128.4, 128.1, 125.3, 125.1, 124.7, 34.7, 31.4, 16.0, 14.3, 12.5 ppm. HRMS (ESI$^+$): calcd for $C_{23}H_{27}S_2^+$: [M+H]$^+$, 367.1549, found: 367.1549.

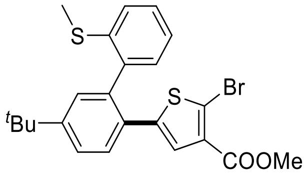

Methyl 2-bromo-5-(5-(*tert*-butyl)-2'-(methylthio)-[1,1'-biphenyl]-2-yl)thiophene-3-carboxylate (3n)

Purification by column chromatography on silica gel (Petroleum ether/CH_2Cl_2 = 1/1, v/v) afforded the desired product **3n** as a yellow solid (59.7mg, 62%). ^{1}H NMR (300 MHz, $CDCl_3$): δ = 7.56-7.52 (m, 1H), 7.47-7.43 (m, 1H), 7.39-7.36 (m, 1H), 7.29 (d, J = 1.8 Hz, 1H), 7.25-7.23 (m, 1H), 7.19-7.09 (m, 3H), 3.82 (s, 3H), 2.31 (s, 3H), 1.35 (s, 9H) ppm; $^{13}C\{^1H\}$ NMR (75 MHz, $CDCl_3$): δ = 162.6, 151.5, 143.2, 139.3, 139.0, 138.0, 130.7, 130.3, 129.3, 128.9, 128.6, 128.5, 127.0, 125.5, 125.1, 124.8, 119.5, 51.9, 34.9, 31.4, 15.7 ppm. HRMS (ESI$^+$): calcd for $C_{23}H_{24}{}^{79}BrO_2S_2^+$: [M+H]$^+$, 475.0396, found: 475.0417; HRMS (ESI$^+$): calcd for $C_{23}H_{24}{}^{81}BrO_2S_2^+$: [M+H]$^+$, 477.0376, found: 477.0285.

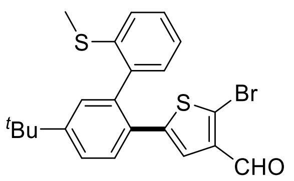

2-Bromo-5-(5-(*tert*-butyl)-2'-(methylthio)-[1,1'-biphenyl]-2-yl)thiophene-3-carbaldehyde (3o)

Purification by column chromatography on silica gel (Petroleum ether/CH_2Cl_2 = 5/1, v/v) afforded the desired product **3o** as a yellow solid (46.0 mg, 52%). ^{1}H NMR (300 MHz, $CDCl_3$): δ = 9.79 (s, 1H), 7.55-7.48 (m, 1H), 7.47-7.42 (m, 1H), 7.40-7.36 (m,

1H), 7.30 (d, J= 1.5 Hz, 1H), 7.25-7.23 (m, 1H), 7.19-7.09 (m, 3H), 2.33 (s, 3H), 1.36 (s, 9H) ppm; $^{13}C\{^{1}H\}$ NMR (75 MHz, $CDCl_3$): δ = 184.9, 151.9, 144.5, 139.0, 138.03, 137.99, 130.6, 129.03, 129.00, 128.7, 128.5, 127.0, 126.4, 125.6, 125.0, 124.8, 123.5, 34.9, 31.3, 15.7 ppm. HRMS (ESI$^+$): calcd for $C_{22}H_{22}{}^{79}BrOS_2{}^+$: $[M+Na]^+$, 467.0110, found: 467.0105; HRMS (ESI$^+$): calcd for $C_{22}H_{22}{}^{81}BrOS_2{}^+$: $[M+Na]^+$, 469.0089, found: 469.0090.

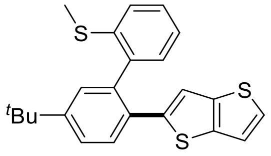

2-(5-(*tert*-Butyl)-2'-(methylthio)-[1,1'-biphenyl]-2-yl)thieno[3,2-*b*]thiophene (3p)

Purification by column chromatography on silica gel (Petroleum ether/CH_2Cl_2 = 15/1, v/v) afforded the desired product **3p** as a yellow solid (34.6 mg, 44%). ^1H NMR (300 MHz, $CDCl_3$): δ = 7.52 (d, J = 8.4 Hz, 1H), 7.38 (dd, J_1 = 8.1 Hz, J_2 = 2.1 Hz, 1H), 7.25-7.22 (m, 2H), 7.17-7.13 (m, 2H), 7.06-7.01 (m, 3H), 6.77 (s, 1H), 2.23 (s, 3H), 1.28 (s, 9H) ppm; $^{13}C\{^{1}H\}$ NMR (75 MHz, $CDCl_3$): δ = 150.7, 145.2, 140.3, 139.3, 139.2, 138.7, 138.0, 131.1, 130.6, 129.5, 128.7, 128.4, 126.2, 125.3, 125.1, 124.7, 119.6, 118.1, 34.8, 31.4, 15.9 ppm. HRMS (ESI$^+$): calcd for $C_{23}H_{23}S_3{}^+$: $[M+H]^+$, 395.0957, found: 395.0959.

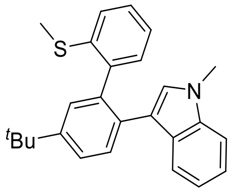

3-(5-(*tert*-Butyl)-2'-(methylthio)-[1,1'-biphenyl]-2-yl)-1-methyl-1*H*-indole (3q)

Purification by column chromatography on silica gel (Petroleum ether/CH_2Cl_2 = 10/1, v/v) afforded the desired product **3q** as a yellow solid (27.7 mg, 36%). ^1H NMR (300 MHz, $CDCl_3$): δ = 7.71 (d, J= 7.8 Hz, 1H), 7.60 (d, J= 8.1 Hz, 1H), 7.41 (dd, J_1 = 2.1 Hz, J_2 = 8.1 Hz, 1H), 7.33 (d, J= 2.4 Hz, 1H), 7.18-7.11 (m, 4H), 7.04-6.00 (m, 1H), 6.97-6.87 (m, 2H), 6.32 (s, 1H), 3.50 (s, 3H), 2.21 (s, 3H), 1.32 (s, 9H) ppm; $^{13}C\{^{1}H\}$ NMR (75 MHz, $CDCl_3$): δ = 147.4, 140.6, 137.5, 137.3, 135.7, 130.2, 129.7, 129.0, 127.5, 127.4, 126.6, 126.5, 123.9, 123.8, 123.3, 120.4, 119.2, 118.4, 113.7, 108.1, 33.7,

31.8, 30.5, 15.0 ppm. HRMS (ESI+): calcd for $C_{26}H_{28}NS^+$: $[M+H]^+$, 386.1937, found: 386.1934.

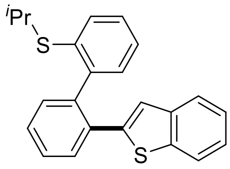

2-(2'-(Isopropylthio)-[1,1'-biphenyl]-2-yl)benzo[*b*]thiophene (4a)

Purification by column chromatography on silica gel (Petroleum ether/CH_2Cl_2 = 10/1, v/v) afforded the desired product **4a** as a yellow solid (26.7 mg, 37%). ^{1}H NMR (300 MHz, $CDCl_3$): δ = 7.72-7.67 (m, 2H), 7.57-7.54 (m, 1H), 7.45-7.38 (m, 2H), 7.37-7.29 (m, 3H), 7.25-7.19 (m, 2H), 7.17-7.10 (m, 2H), 6.91 (s, 1H), 3.31 (q, J = 6.6 Hz, 1H), 1.19 (d, J = 6.6 Hz, 3H), 1.08 (d, d, J = 6.6 Hz, 3H) ppm; ^{13}C{^{1}H} NMR (75 MHz, $CDCl_3$): δ = 143.5, 141.8, 140.4, 140.1, 139.6, 136.5, 133.8, 131.4, 131.0, 130.2, 129.1, 128.2, 128.2, 127.8, 125.6, 124.1, 123.9, 123.5, 123.1, 122.0, 36.8, 23.0, 22.7 ppm. HRMS (ESI+): calcd for $C_{23}H_{21}S_2^+$: $[M+H]^+$, 361.1080, found: 361.1083.

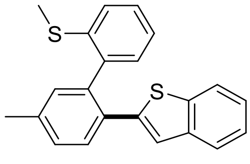

2-(5-Methyl-2'-(methylthio)-[1,1'-biphenyl]-2-yl)benzo[*b*]thiophene (4b)

Purification by column chromatography on silica gel (Petroleum ether/CH_2Cl_2 = 15/1, v/v) afforded the desired product **4b** as a yellow solid (43.6 mg, 63%). ^{1}H NMR (300 MHz, $CDCl_3$): δ = 7.71-7.62 (m, 2H), 7.60-7.57 (m, 1H), 7.37-7.35 (m, 1H), 7.32-7.28 (m, 2H), 7.25-7.21 (m, 3H), 7.16 (s, 2H), 6.94-6.93 (m, 1H), 2.44 (s, 3H), 2.31 (s, 3H) ppm; ^{13}C{^{1}H} NMR (75 MHz, $CDCl_3$): δ = 143.4, 140.3, 140.1, 140.0, 138.8, 138.5, 138.0, 131.9, 131.0, 130.5, 130.1, 129.2, 128.4, 125.0, 124.7, 124.0, 123.8, 123.5, 122.5, 122.0, 21.3, 15.9 ppm. HRMS (ESI+): calcd for $C_{22}H_{19}S_2^+$: $[M+H]^+$, 347.0923, found: 347.0924.

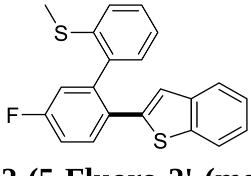

2-(5-Fluoro-2'-(methylthio)-[1,1'-biphenyl]-2-yl)benzo[*b*]thiophene (4c)

Purification by column chromatography on silica gel (Petroleum ether/CH_2Cl_2 = 10/1, v/v) afforded the desired product **4c** as a yellow solid (40.6 mg, 58%). ^{1}H NMR (300 MHz, $CDCl_3$): δ = 7.70-7.66 (m, 2H), 7.58 (d, J = 8.41 Hz, 1H), 7.39-7.32 (m, 1H), 7.24-7.13 (m, 6H), 7.10-7.06 (m, 1H), 6.92 (s, 1H), 2.31 (s, 3H) ppm; ^{13}C{^{1}H} NMR (75 MHz, $CDCl_3$): δ = 162.2 (d, J_{CF} = 248.25 Hz), 142.2, 140.4, 140.0, 138.8, 138.4, 132.1 (d, J_{CF} = 8.25 Hz), 130.4, 130.2 (d, J_{CF} = 3 Hz), 128.9, 128.6, 125.4, 124.9, 124.1 (d, J_{CF} = 7.5 Hz), 123.6, 123.0, 122.03, 121.95, 118.2 (d, J_{CF} = 21 Hz), 115.4 (d, J_{CF} = 8.25 Hz), 15.9 ppm. HRMS (ESI$^+$): calcd for $C_{21}H_{16}FS_2^+$: [M+H]$^+$, 351.0672, found: 351.0677.

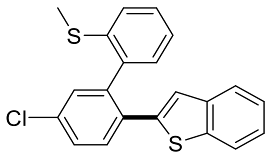

2-(5-Chloro-2'-(methylthio)-[1,1'-biphenyl]-2-yl)benzo[*b*]thiophene (4d)

Purification by column chromatography on silica gel (Petroleum ether/CH_2Cl_2 = 10/1, v/v) afforded the desired product **4d** as a yellow solid (54.2 mg, 74%). ^{1}H NMR (300 MHz, $CDCl_3$): δ = 7.68-7.62 (m, 2H), 7.58-7.55 (m, 1H), 7.41 (dd, J_1= 8.4 Hz, J_2 = 2.1 Hz, 1H), 7.36-7.31(m, 2H), 7.24-7.18 (m, 3H), 7.12 (d, J = 4.2 Hz, 2H), 6.94 (s, 1H), 2.28 (s, 3H) ppm;^{13}C{^{1}H} NMR (75 MHz, $CDCl_3$): δ = 142.0, 140.4, 140.3, 139.9, 138.4, 133.8, 132.5, 131.4, 131.2, 130.4, 128.9, 128.4, 125.3, 124.9, 124.2, 123.7, 123.2, 122.0, 15.8 ppm. HRMS (ESI$^+$): calcd for $C_{21}H_{16}{}^{35}ClS_2^+$: [M+H]$^+$, 367.0377, found: 367.0377 ppm; HRMS (ESI$^+$): calcd for $C_{21}H_{16}{}^{37}ClS_2^+$: [M+H]$^+$, 369.0347, found: 369.0293.

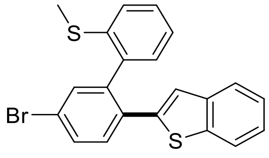

2-(5-Bromo-2'-(methylthio)-[1,1'-biphenyl]-2-yl)benzo[*b*]thiophene (4e)

Purification by column chromatography on silica gel (Petroleum ether/CH_2Cl_2 = 10/1, v/v) afforded the desired product **4e** as a yellow solid (50.9 mg, 62%). ^{1}H NMR (300 MHz, $CDCl_3$): δ = 7.68-7.65 (m, 1H), 7.58-7.56 (m, 3H), 7.48 (s, 1H), 7.35-7.31 (m, 1H), 7.24-7.18 (m, 3H), 7.13 (d, J = 4.5 Hz, 2H), 6.94 (s, 1H), 2.28 (s, 3H) ppm; ^{13}C{^{1}H}

NMR (75 MHz, $CDCl_3$): δ = 148.4, 142.0, 140.7, 140.4, 139.9, 138.5, 134.1, 132.9, 131.6, 131.4, 130.4, 128.9, 125.3, 124.9, 124.24, 124.22, 123.7, 123.2, 122.0, 121.9, 15.9 ppm. HRMS (ESI^+): calcd for $C_{21}H_{16}{}^{79}BrS_2{}^+$: $[M+H]^+$, 410.9872, found: 410.9851 ppm; HRMS (ESI^+): calcd for $C_{21}H_{16}{}^{81}BrS_2{}^+$: $[M+H]^+$, 412.9851, found: 412.9850.

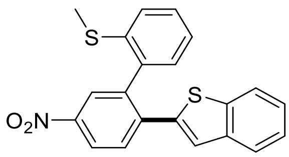

2-(2'-(Methylthio)-5-nitro-[1,1'-biphenyl]-2-yl)benzo[*b*]thiophene (4f)

Purification by column chromatography on silica gel (Petroleum ether/CH_2Cl_2 = 2/1, v/v) afforded the desired product **4f** as a yellow solid (27.1 mg, 36%). ^1H NMR (300 MHz, $CDCl_3$): δ = 8.29-8.20 (m, 2H), 7.87 (d, J = 8.7 Hz, 1H), 7.68-7.61 (m, 3H), 7.40-7.38 (m, 2H), 7.24-7.17 (m, 3H), 7.09 (s, 1H), 2.29 (s, 3H) ppm; $^{13}C\{^1H\}$ NMR (75 MHz, $CDCl_3$): δ = 146.9, 140.9, 140.6, 140.5, 140.0, 139.6, 138.5, 137.7, 130.8, 130.4, 129.5, 126.8, 125.8, 125.2, 125.1, 124.6, 124.2, 123.2, 122.1, 16.0 ppm. HRMS (ESI^+): calcd for $C_{21}H_{16}NO_2S_2{}^+$: $[M+Na]^+$, 400.0437, found: 400.0436.

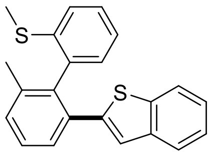

2-(6-Methyl-2'-(methylthio)-[1,1'-biphenyl]-2-yl)benzo[*b*]thiophene (4g)

Purification by column chromatography on silica gel (Petroleum ether/CH_2Cl_2 = 15/1, v/v) afforded the desired product **4g** as a yellow solid (36.0 mg, 52%). ^1H NMR (300 MHz, $CDCl_3$): δ = 7.68 (d, J = 8.4 Hz, 1H), 7.58 (d, J = 6.9 Hz, 2H), 7.41-7.31 (m, 3H), 7.25-7.18 (m, 3H), 7.16-7.05 (m, 2H), 6.95 (s, 1H), 2.35 (s, 3H), 2.11 (s, 3H) ppm; $^{13}C\{^1H\}$ NMR (75 MHz, $CDCl_3$): δ = 143.7, 140.5, 140.0, 138.9, 138.5, 138.3, 137.9, 134.1, 130.3, 130.1, 128.4, 128.2, 128.1, 124.9, 124.5, 124.0, 123.8, 123.5, 123.0, 121.9, 20.6, 15.3 ppm. HRMS (ESI^+): calcd for $C_{22}H_{19}S_2{}^+$: $[M+H]^+$, 347.0923, found: 347.0922.

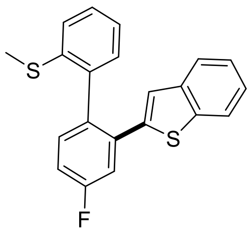

2-(4-Fluoro-2'-(methylthio)-[1,1'-biphenyl]-2-yl)benzo[*b*]thiophene (4h)

Purification by column chromatography on silica gel (Petroleum ether/CH_2Cl_2 = 10/1, v/v) afforded the desired product **4h** as a yellow solid (37.1 mg, 53%). ^{1}H NMR (300 MHz, $CDCl_3$): δ = 7.70-7.67 (m, 1H), 7.60-7.57 (m, 1H), 7.43 (dd, J_1 = 9.9 Hz, J_2 = 2.7 Hz, 1H), 7.35-7.31 (m, 1H), 7.29-7.20 (m, 4H), 7.13-7.08 (m, 3H), 6.98 (s, 1H), 2.30 (s, 3H) ppm; ^{13}C{^{1}H} NMR (75 MHz, $CDCl_3$): δ = 162.4 (d, J_{CF} = 245.25 Hz), 141.9 (d, J_{CF} = 2.25 Hz), 140.5, 139.8, 138.8, 135.8 (d, J_{CF} = 8.25 Hz), 134.9 (d, J_{CF} = 3.75 Hz), 133.0 (d, J_{CF} = 8.25 Hz), 130.8, 128.7, 125.1, 124.9, 124.4, 124.3, 123.8, 123.5, 122.1, 116.8 (d, J_{CF} = 22.5 Hz), 115.0 (d, J_{CF} = 21 Hz), 15.8 ppm. HRMS (ESI$^+$): calcd for $C_{21}H_{16}FS_2^+$: [M+H]$^+$, 351.0672, found: 351.0678.

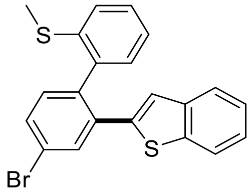

2-(4-Bromo-2'-(methylthio)-[1,1'-biphenyl]-2-yl)benzo[*b*]thiophene (4i)

Purification by column chromatography on silica gel (Petroleum ether/CH_2Cl_2 = 2/1, v/v) afforded the desired product **4i** as a yellow solid (50.1 mg, 61%). ^{1}H NMR (300 MHz, $CDCl_3$): δ = 7.86 (d, J = 2.1 Hz, 1H), 7.67-7.64 (m, 1H), 7.59-7.56 (m, 1H), 7.51 (dd, J_1 = 8.4 Hz, J_2 = 2.1 Hz, 1H), 7.32-7.29 (m, 1H), 7.24-7.19 (m, 3H), 7.17 (d, J = 3.0 Hz, 1H), 7.09 (d, J = 3.3 Hz, 2H), 6.99 (s, 1H), 2.27 (s, 3H); ^{13}C{^{1}H} NMR (75 MHz, $CDCl_3$): δ = 141.5, 140.5, 139.7, 138.5, 137.8, 135.9, 132.8, 130.9, 130.4, 128.8, 125.2, 124.8, 124.3, 124.2, 123.8, 123.6, 122.2, 122.0, 15.8 ppm. HRMS (ESI$^+$): calcd for $C_{21}H_{16}{}^{79}BrS_2^+$: [M+H]$^+$, 410.9872, found: 410.9889 ppm; HRMS (ESI$^+$): calcd for $C_{21}H_{16}{}^{81}BrS_2^+$: [M+H]$^+$, 412.9851, found: 412.9851.

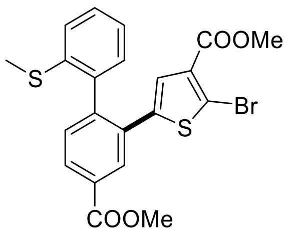

Methyl 2-bromo-5-(4-(methoxycarbonyl)-2'-(methylthio)-[1,1'-biphenyl]-2-yl)thiophene-3-carboxylate (4j)

Purification by column chromatography on silica gel (Petroleum ether/CH_2Cl_2 = 1/1, v/v) afforded the desired product **4j** as a yellow solid (58.1 mg, 61%). ^{1}H NMR (300 MHz, $CDCl_3$): δ = 8.28 (d, J = 1.8 Hz, 1H), 8.05 (dd, J_1 = 7.8 Hz, J_2 = 1.8 Hz, 1H), 7.42-7.32 (m, 3H), 7.26-7.16 (m, 2H), 7.08 (dd, J_1 = 7.5 Hz, J_2 = 1.5 Hz, 1H), 3.97 (s, 3H), 3.84 (s, 3H), 2.33 (s, 3H) ppm; ^{13}C{^{1}H} NMR (75 MHz, $CDCl_3$): δ = 166.5, 162.5, 142.9, 141.8, 138.5, 138.0, 132.7, 131.6, 130.4, 130.2, 130.1, 129.5, 129.2, 128.9, 128.1, 125.4, 125.1, 120.6, 52.5, 52.0, 15.7 ppm. HRMS (ESI$^+$): calcd for $C_{21}H_{18}{}^{79}BrO_4S_2{}^+$: [M+Na]$^+$, 498.9644, found: 498.9641; HRMS (ESI$^+$): calcd for $C_{21}H_{18}{}^{81}BrO_4S_2{}^+$: [M+Na]$^+$, 500.9624, found: 500.9622.

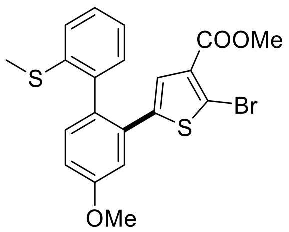

Methyl 2-bromo-5-(4-methoxy-2'-(methylthio)-[1,1'-biphenyl]-2-yl)thiophene-3-carboxylate (4k)

Purification by column chromatography on silica gel (Petroleum ether/CH_2Cl_2 = 1/1, v/v) afforded the desired product **4k** as a yellow solid (33.9 mg, 38%). ^{1}H NMR (300 MHz, $CDCl_3$): δ = 7.37 (m, 1H), 7.22-7.18 (m, 3H), 7.14-7.06 (m, 3H), 6.96 (dd, J_1 = 7.2 Hz, J_2 = 2.7 Hz, 1H), 3.89 (s, 3H), 3.82 (s, 3H), 2.32 (s, 3H) ppm; ^{13}C{^{1}H} NMR (75 MHz, $CDCl_3$): δ = 162.6, 159.5, 142.9, 139.4, 138.4, 135.5, 133.3, 132.4, 131.0, 130.3, 128.9, 127.5, 126.0 124.8, 120.1, 114.1, 55.6, 52.0, 15.6 ppm. HRMS (ESI$^+$): calcd for $C_{20}H_{18}{}^{79}BrO_3S_2{}^+$: [M+Na]$^+$, 470.9695, found: 470.9695; HRMS (ESI$^+$): calcd for $C_{20}H_{18}{}^{81}BrO_3S_2{}^+$: [M+Na]$^+$, 472.9675, found:472.9672.

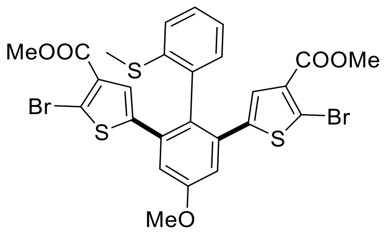

Dimethyl 5,5'-(4-methoxy-2'-(methylthio)-[1,1'-biphenyl]-2,6-diyl)bis(2-bromothiophene-3-carboxylate) (4k')

Purification by column chromatography on silica gel (Petroleum ether/CH_2Cl_2 = 1/2, v/v) afforded the desired product **4k'** as a yellow solid (20.0 mg, 15%). ^{1}H NMR (300 MHz, $CDCl_3$): δ = 7.41-7.35 (m, 1H), 7.22 (s, 1H), 7.15-7.08 (m, 4H), 6.99 (dd, J_1 = 7.5 Hz, J_2 = 1.5 Hz, 2H), 3.92 (s, 3H), 3.82 (s, Hz, 6H), 2.31 (s, 3H) ppm; $^{13}C\{^1H\}$ NMR (75 MHz, $CDCl_3$): δ = 162.5, 159.3, 142.4, 141.1, 135.3, 135.0, 132.2, 130.1, 129.9, 129.3, 129.1, 128.3, 127.9, 125.0, 124.8, 120.6, 115.2, 55.8, 52.0, 15.3 ppm. HRMS (ESI$^+$): calcd for $C_{26}H_{21}{}^{79}Br_2O_5S_3{}^+$: [M+Na]$^+$, 688.8737, found: 688.8737; HRMS (ESI$^+$): calcd for $C_{26}H_{21}{}^{81}Br_2O_5S_3{}^+$: [M+Na]$^+$, 690.8712, found:690.8690.

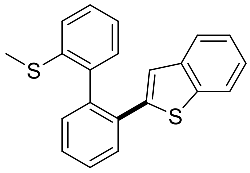

2-(2'-(Methylthio)-[1,1'-biphenyl]-2-yl)benzo[*b*]thiophene (4l)

Purification by column chromatography on silica gel (Petroleum ether/CH_2Cl_2 = 15/1, v/v) afforded the desired product **4k** as a yellow solid (36.5 mg, 55%). ^{1}H NMR (300 MHz, $CDCl_3$): δ = 7.75-7.68 (m, 2H), 7.61-7.57 (m, 1H), 7.50-7.40 (m, 2H), 7.35-7.31 (m, 2H), 7.26-7.21(m, 3H), 7.14-7.13 (m, 2H), 6.96 (s, 1H), 2.31 (s, 3H) ppm; $^{13}C\{^1H\}$ NMR (75 MHz, $CDCl_3$): δ = 143.3, 140.4, 140.0, 139.8, 138.9, 138.5, 133.8, 131.3, 130.5, 130.3, 128.5, 128.4, 128.0, 125.1, 124.8, 124.1, 124.0, 123.6, 123.0, 122.0, 15.9 ppm. HRMS (ESI$^+$): calcd for $C_{21}H_{17}S_2{}^+$: [M+H]$^+$, 333.0767, found: 333.0769.

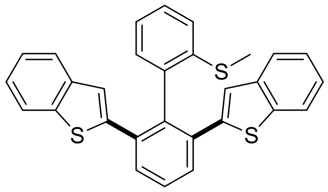

2,2'-(2'-(Methylthio)-[1,1'-biphenyl]-2,6-diyl)bis(benzo[*b*]thiophene) (4l')

Purification by column chromatography on silica gel (Petroleum ether/CH_2Cl_2 = 10/1, v/v) afforded the desired product **4k'** as a yellow solid (9.2 mg, 10%). ^{1}H NMR (300 MHz, $CDCl_3$): δ = 7.72 (s, 1H), 7.69 (s, 2H), 7.66 (d, J = 2.1 Hz, 1H), 7.59 (d, J = 2.1 Hz, 1H), 7.58-7.56 (m, 1H), 7.54-7.51 (m, 1H), 7.24-7.20 (m, 5H), 7.11 (t, J = 7.5 Hz, 2H), 7.03 (dd, J_1 = 7.2 Hz, J_2 = 1.2 Hz, 1H), 6.95 (s, 2H), 2.21 (s, 3H) ppm; ^{13}C{^{1}H} NMR (75 MHz, $CDCl_3$): δ = 143.1, 140.6, 139.9, 137.7, 137.5, 135.4, 131.6, 130.8, 128.9, 128.4, 125.4, 124.8, 124.1, 124.0, 123.7, 123.6, 122.0, 15.9 ppm. HRMS (ESI$^+$): calcd for $C_{29}H_{21}S_3^+$: [M+H]$^+$, 465.0800, found: 465.0800.

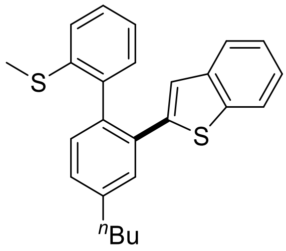

2-(4-Butyl-2'-(methylthio)-[1,1'-biphenyl]-2-yl)benzo[*b*]thiophene (4m)

Purification by column chromatography on silica gel (Petroleum ether/CH_2Cl_2 = 10/1, v/v) afforded the desired product **4m** as a yellow solid (40.6 mg, 52%). ^{1}H NMR (300 MHz, $CDCl_3$): δ = 7.69-7.66 (m, 1H), 7.58-7.54 (m, 2H), 7.34-7.28 (m, 1H), 7.26-7.17 (m, 5H), 7.16-7.07 (m, 2H), 6.95 (s, 1H), 2.72 (t, J = 7.8 Hz, 2H), 2.29 (s, 3H), 1.75-1.65 (m, 2H), 1.50-1.37 (m, 2H), 0.97 (t, J = 7.5 Hz, 3H) ppm; ^{13}C{^{1}H} NMR (75 MHz, $CDCl_3$): δ = 143.6, 143.1, 140.3, 140.1, 139.8, 138.7, 136.3, 133.5, 131.2, 130.7, 130.3, 128.3, 128.2, 124.9, 124.7, 124.0, 123.9, 123.5, 122.8, 122.0, 35.6, 33.6, 22.7, 15.8, 14.2 ppm. HRMS (ESI$^+$): calcd for $C_{25}H_{25}S_2^+$: [M+H]$^+$, 389.1393, found: 389.1395.

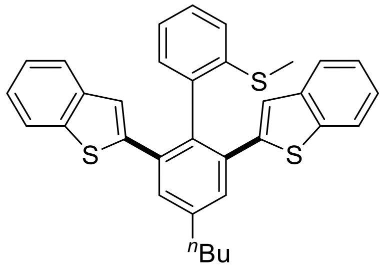

2,2'-(4-Butyl-2'-(methylthio)-[1,1'-biphenyl]-2,6-diyl)bis(benzo[*b*]thiophene) (4m')

Purification by column chromatography on silica gel (Petroleum ether/CH_2Cl_2 = 5/1, v/v) afforded the desired product **4m'** as a yellow solid (12.8 mg, 12%). ^{1}H NMR (300

MHz, $CDCl_3$): δ = 7.69-7.66 (m, 2H), 7.59-7.56 (m, 2H), 7.54 (s, 2H), 7.24-7.17 (m, 5H), 7.14-7.07 (m, 2H), 7.03-6.98 (m, 1H), 6.95 (s, 2H), 2.75 (t, J = 7.8 Hz, 2H), 2.21 (s, 3H), 1.79-1.69 (m, 2H), 1.51-1.45 (m, 2H), 0.98 (t, J = 7.2 Hz, 3H) ppm; $^{13}C\{^1H\}$ NMR (75 MHz, $CDCl_3$): δ = 143.4, 143.1, 140.5, 140.0, 139.9, 137.6, 135.1, 135.0, 131.8, 130.9, 128.8, 125.3, 124.8, 124.0, 123.9, 123.6, 123.5, 122.0, 35.5, 33.5, 22.7, 15.8, 14.2 ppm. HRMS (ESI^+): calcd for $C_{33}H_{29}S_3^+$: $[M+H]^+$, 521.1426, found: 521.1427.

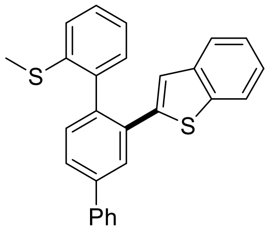

2-(2-(Methylthio)-[1,1':4',1''-terphenyl]-2'-yl)benzo[*b*]thiophene (4n)

Purification by column chromatography on silica gel (Petroleum ether/CH_2Cl_2 = 10/1, v/v) afforded the desired product **4n** as a yellow solid (35.2 mg, 43%).^{1}H NMR (300 MHz, $CDCl_3$): δ = 7.95 (d, J = 1.8 Hz, 1H), 7.70-7.68 (m, 3H), 7.65-7.56 (m, 2H), 7.46 (t, J = 6.9 Hz, 2H), 7.40 (d, J = 7.8 Hz, 1H), 7.37-7.29 (m, 2H), 7.25-7.15 (m, 4H), 7.14-7.08 (m, 1H), 7.02 (s, 1H), 2.29 (s, 3H) ppm; $^{13}C\{^1H\}$ NMR (75 MHz, $CDCl_3$): δ = 143.2, 141.2, 140.4, 140.0, 139.5, 138.6, 137.9, 134.3, 131.8, 130.6, 129.00, 128.96, 128.5, 127.7, 127.3, 126.7, 125.1, 124.7, 124.11, 124.05, 123.6, 123.1, 122.0, 15.8 ppm. HRMS (ESI^+): calcd for $C_{27}H_{22}S_2^+$: $[M+H]^+$, 409.1080, found: 409.1082.

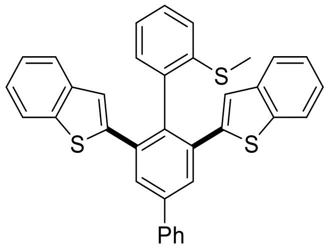

2,2'-(2-(Methylthio)-[1,1':4',1''-terphenyl]-2',6'-diyl)bis(benzo[b]thiophene) (4n')

Purification by column chromatography on silica gel (Petroleum ether/CH_2Cl_2 = 5/1, v/v) afforded the desired product **4n'** as a yellow solid (12.0 mg, 11%).^{1}H NMR (300 MHz, $CDCl_3$): δ = 7.95 (s, 2H), 7.77-7.69 (m, 4H), 7.63-7.60 (m, 2H), 7.51 (t, J = 6.9 Hz, 2H), 7.42 (t, J = 7.5 Hz, 2H), 7.28 (dd, J = 7.2, 1.8 Hz, 2H), 7.25-7.23 (m, 2H), 7.19-7.12 (m, 2H), 7.07 (dd, J = 7.5, 1.5 Hz, 1H), 7.03 (s, 2H), 2.25 (s, 3H) ppm. $^{13}C\{^1H\}$

NMR (75 MHz, $CDCl_3$): δ = 143.1, 141.3, 140.6, 140.0, 139.92, 139.86, 137.3, 136.6, 135.9, 131.7, 129.5, 129.1, 129.0, 128.0, 127.4, 125.4, 124.9, 124.14, 124.10, 123.8, 123.7, 122.02, 15.9 ppm. HRMS (ESI$^+$): calcd for $C_{35}H_{25}S_3^+$: [M+H]$^+$, 541.1113, found: 541.1112.

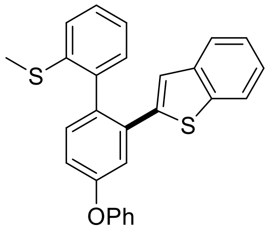

2-(2'-(Methylthio)-4-phenoxy-[1,1'-biphenyl]-2-yl)benzo[*b*]thiophene (4o)

Purification by column chromatography on silica gel (Petroleum ether/CH_2Cl_2 = 10/1, v/v) afforded the desired product **4o** as a yellow solid (34.5 mg, 41%).^{1}H NMR (300 MHz, $CDCl_3$): δ = 7.63-7.61 (m, 1H), 7.54-7.51 (m, 1H), 7.38-7.32 (m, 3H), 7.29-7.23 (m, 2H), 7.20-7.16 (m, 3H), 7.14-7.08 (m, 5H), 7.00 (dd, J = 8.4, 2.4 Hz, 1H), 6.92 (s, 1H), 2.27 (s, 3H) ppm. ^{13}C{^{1}H} NMR (75 MHz, $CDCl_3$): δ = 157.3, 156.7, 142.6, 140.4, 139.9, 139.2, 138.9, 135.4, 133.7, 132.7, 130.8, 130.0, 128.5, 124.9, 124.7, 124.13, 124.11, 123.9, 123.7, 123.2, 122.0, 119.8, 119.5, 117.8, 15.7 ppm. HRMS (ESI$^+$): calcd for $C_{27}H_{21}OS_2^+$: [M+H]$^+$, 425.1029, found: 425.1030.

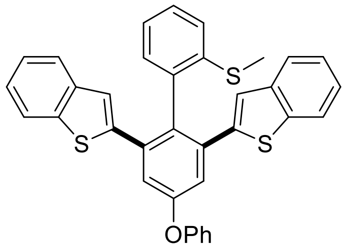

2,2'-(2'-(Methylthio)-4-phenoxy-[1,1'-biphenyl]-2,6-diyl)bis(benzo[*b*]thiophene) (4o')

Purification by column chromatography on silica gel (Petroleum ether/CH_2Cl_2 = 5/1, v/v) afforded the desired product **4o'** as a yellow solid (11.5 mg, 10%).^{1}H NMR (300 MHz, $CDCl_3$): δ = 7.69-7.66 (m, 2H), 7.60-7.57 (m, 2H), 7.45-7.39 (m, 2H), 7.37 (s, 2H), 7.28 (dd, J = 7.8, 1.2 Hz, 1H), 7.25-7.16 (m, 7H), 7.14-7.10 (m, 2H), 7.07-7.01 (m, 1H), 6.96 (s, 2H), 2.25 (s, 3H) ppm. ^{13}C{^{1}H} NMR (75 MHz, $CDCl_3$): δ = 157.0, 156.4, 142.5, 140.6, 140.3, 139.7, 137.0, 136.9, 132.4, 132.0, 130.1, 129.0, 125.2, 124.8,

124.2, 124.1, 123.8, 123.7, 122.0, 120.1, 119.7, 15.8 ppm. HRMS (ESI$^+$): calcd for $C_{35}H_{25}OS_3^+$: [M+H]$^+$, 557.1063, found: 557.1066.

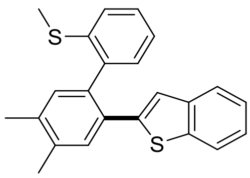

2-(4,5-Dimethyl-2'-(methylthio)-[1,1'-biphenyl]-2-yl)benzo[*b*]thiophene (4p)

Purification by column chromatography on silica gel (Petroleum ether/CH_2Cl_2 = 10/1, v/v) afforded the desired product **4p** as a yellow solid (46.8 mg, 65%). ^{1}H NMR (300 MHz, $CDCl_3$): δ = 7.68 (d, J = 6.6 Hz, 1H), 7.58-7.52 (m, 2H), 7.35-7.30 (m, 1H), 7.24-7.19 (m, 3H), 7.13 (d, J = 10.2 Hz, 3H), 6.92 (s, 1H), 2.36 (s, 3H), 2.32 (s, 3H), 2.28 (s, 3H) ppm; ^{13}C{^{1}H} NMR (75 MHz, $CDCl_3$) δ = 143.6, 140.4, 140.2, 139.9, 138.7, 136.9, 136.4, 132.5, 131.4, 131.2, 130.7, 128.4, 127.2, 124.9, 124.7, 124.1, 123.9, 123.5, 122.4, 122.1, 19.9, 19.8, 15.9 ppm. HRMS (ESI$^+$): calcd for $C_{23}H_{21}S_2^+$: [M+H]$^+$, 361.1080, found: 361.1075.

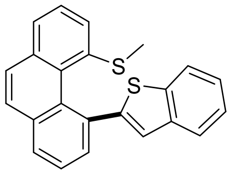

2-(5-(Methylthio)phenanthren-4-yl)benzo[*b*]thiophene (4q)

Purification by column chromatography on silica gel (Petroleum ether/CH_2Cl_2 = 20/1, v/v) afforded the desired product **4q** as a yellow solid (38.5 mg, 54%). ^{1}H NMR (300 MHz, $CDCl_3$): δ = 7.83-7.75 (m, 3H), 7.69 (d, J = 7.5 Hz, 1H), 7.64-7.59 (m, 3H), 7.52-7.47 (m, 2H), 7.38 (d, J = 7.5 Hz, 1H), 7.26-7.24 (m, 2H), 6.85 (s, 1H), 2.00 (s, 3H) ppm; ^{13}C{^{1}H} NMR (75 MHz, $CDCl_3$): δ = 146.2, 140.2, 140.0, 139.6, 134.9, 134.8, 134.0, 130.9, 129.5, 128.4, 127.4, 127.2, 127.1, 127.0, 125.5, 124.3, 124.2, 123.7, 122.29, 122.25, 21.4 ppm. HRMS (ESI$^+$): calcd for $C_{23}H_{17}S_2^+$: [M+H]$^+$, 357.0767, found: 357.0763.

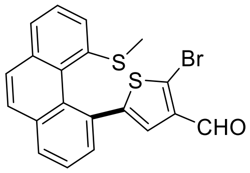

2-Bromo-5-(5-(methylthio)phenanthren-4-yl)thiophene-3-carbaldehyde (4r)

Purification by column chromatography on silica gel (Petroleum ether/CH_2Cl_2 = 1/1, v/v) afforded the desired product **4r** as a yellow solid (45.4 mg, 55%). ^{1}H NMR (300 MHz, $CDCl_3$): δ = 9.83 (s, 1H), 7.79 (dd, J_1 = 6.3 Hz, J_2 = 3.0 Hz, 1H), 7.69 (dd, J_1 = 7.5 Hz, J_2 = 0.9 Hz, 1H), 7.63-7.59 (m, 3H), 7.53 (t, J = 7.5 Hz, 1H), 7.43 (dd, J_1 = 7.5 Hz, J_2 = 1.5 Hz, 1H), 7.02 (s, 1H), 2.11 (s, 3H) ppm; ^{13}C{^{1}H} NMR (75 MHz, $CDCl_3$): δ = 184.8, 147.8, 139.1, 138.4, 134.8, 134.0, 132.8, 130.0 128.9, 128.4, 127.8, 127.7, 127.6, 127.1, 127.0, 126.8, 125.8, 124.4, 123.3, 21.1 ppm. HRMS (ESI$^+$): calcd for $C_{20}H_{14}{}^{79}BrOS_2^+$: [M+H]$^+$, 412.9664, found: 412.9642; HRMS (ESI$^+$): calcd for $C_{20}H_{14}{}^{81}BrOS_2^+$: [M+H]$^+$, 414.9644, found: 414.9655.

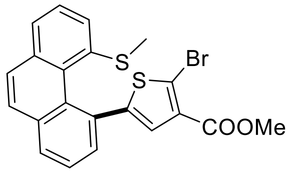

Methyl 2-bromo-5-(5-(methylthio)phenanthren-4-yl)thiophene-3-carboxylate (4s)

Purification by column chromatography on silica gel (Petroleum ether/CH_2Cl_2 = 5/1, v/v) afforded the desired product **4s** as a yellow solid (54.1 mg, 61%). ^{1}H NMR (300 MHz, $CDCl_3$): δ = 7.66 (dd, J_1 = 6.3 Hz, J_2 = 2.7 Hz, 1H), 7.69-7.66 (m, 1H), 7.62-7.59 (m, 4H), 7.52-7.50 (m, 1H), 7.43 (dd, J_1 = 7.5Hz, J_2 = 0.9 Hz, 1H), 7.05 (s, 1H), 3.79 (s, 3H), 2.12 (s, 3H) ppm; ^{13}C{^{1}H} NMR (75 MHz, $CDCl_3$) δ: = 162.6, 146.5, 139.4, 134.7, 134.0, 133.1, 130.6, 130.0, 128.8, 128.1, 127.6, 127.44, 127.39, 127.1, 127.0, 126.8, 126.7, 125.5, 118.8, 51.9, 21.0 ppm. HRMS (ESI$^+$): calcd for $C_{21}H_{16}{}^{79}BrO_2S_2^+$: [M+H]$^+$, 442.9770, found: 442.9742 ppm; HRMS (ESI$^+$): calcd for $C_{21}H_{16}{}^{81}BrO_2S_2^+$: [M+H]$^+$, 444.9750, found: 444.9747.

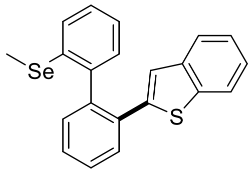

2-(2'-(Methylselanyl)-[1,1'-biphenyl]-2-yl)benzo[*b*]thiophene (6a)

Purification by column chromatography on silica gel (Petroleum ether/CH_2Cl_2 = 20/1, v/v) afforded the desired product **6a** as a yellow solid (31.2 mg, 41%). ^{1}H NMR (300

MHz, $CDCl_3$): δ = 7.73-7.67 (m, 2H), 7.59-7.56 (m, 1H), 7.49-7.46 (m, 1H), 7.44-7.43 (m, 1H), 7.41-7.38 (m, 1H), 7.33 (d, J = 6.6 Hz, 2H), 7.30-7.27 (m, 1H), 7.24-7.23 (m, 1H), 7.22-7.20 (m, 1H), 7.15 (d, J = 3.9 Hz, 1H), 6.95 (s, 1H) 2.15 (s, 3H) ppm; $^{13}C\{^1H\}$ NMR (75 MHz, $CDCl_3$): δ = 143.1, 141.9, 140.4, 140.03, 139.98, 133.7, 133.5, 131.2, 130.5, 130.3, 128.54, 128.48, 128.4, 128.0, 125.7, 124.1, 123.6, 123.2, 122.6, 122.0, 6.9 ppm. HRMS (ESI$^+$): calcd for $C_{21}H_{17}SSe^+$: $[M+H]^+$, 381.0211, found: 381.0211.

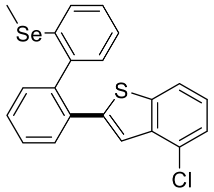

4-Chloro-2-(2'-(methylselanyl)-[1,1'-biphenyl]-2-yl)benzo[*b*]thiophene (6b)

Purification by column chromatography on silica gel (Petroleum ether/CH_2Cl_2 = 20/1, v/v) afforded the desired product **6b** as a yellow solid (55.9 mg, 68%). ^1H NMR (300 MHz, $CDCl_3$): δ = 7.76 (dd, J_1 = 1.8 Hz, J_2 = 7.5 Hz, 1H), 7.56 (d, J_1 = 7.2 Hz, 1H), 7.50-7.42 (m, 3H), 7.38-7.31 (m, 3H), 7.24-7.22 (m, 1H), 7.19-7.14 (m, 3H), 2.18 (s, 3H) ppm; $^{13}C\{^1H\}$ NMR (75 MHz, $CDCl_3$): δ = 144.2, 141.7, 141.5, 140.1, 138.2, 133.7, 133.1, 131.2, 130.5, 130.2, 129.2, 128.7, 128.59, 128.55, 128.5, 128.4, 125.8, 124.7, 124.1, 121.4, 120.6, 7.0 ppm. HRMS (ESI$^+$): calcd for $C_{21}H_{16}{}^{35}ClSSe^+$: $[M+H]^+$, 414.9821, found: 414.9820; HRMS (ESI$^+$): calcd for $C_{21}H_{16}{}^{37}ClSSe\ ^+$: $[M+H]^+$, 416.9792, found: 416.9795.

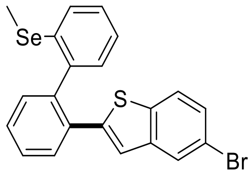

5-Bromo-2-(2'-(methylselanyl)-[1,1'-biphenyl]-2-yl)benzo[*b*]thiophene (6c)

Purification by column chromatography on silica gel (Petroleum ether/CH_2Cl_2 = 20/1, v/v) afforded the desired product **6c** as a yellow solid (56.8 mg, 62%). ^1H NMR (300 MHz, $CDCl_3$): δ = 7.56-7.54 (m, 2H), 7.40-7.33 (m, 2H), 7.31-7.28 (m, 1H), 7.21-7.14 (m, 4H), 7.05-6.98 (m, 2H), 6.68 (s, 1H), 2.01 (s, 3H) ppm; $^{13}C\{^1H\}$ NMR (75 MHz, $CDCl_3$): δ = 145.0, 141.6, 140.0, 138.9, 133.5, 133.1, 131.2, 130.4, 130.3, 128.7, 128.6, 128.4, 128.3, 127.0, 126.1, 125.7, 123.4, 122.3, 118.1, 6.9 ppm. HRMS (ESI$^+$): calcd

for $C_{21}H_{16}{}^{79}BrSSe^+$: $[M+H]^+$, 458.9316, found: 458.9309; HRMS (ESI$^+$): calcd for $C_{21}H_{16}{}^{81}BrSSe^+$: $[M+H]^+$, 460.9296, found: 460.9295.

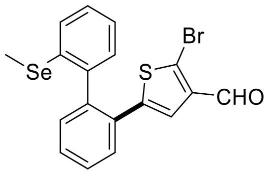

2-Bromo-5-(2'-(methylselanyl)-[1,1'-biphenyl]-2-yl)thiophene-3-carbaldehyde (6d)

Purification by column chromatography on silica gel (Petroleum ether/CH_2Cl_2 = 2/1, v/v) afforded the desired product **6d** as a yellow solid (27.9 mg, 32%). ^1H NMR (300 MHz, DMSO-d_6): δ = 9.71 (s, 1H), 7.76 (d, J = 6.6 Hz, 1H), 7.51-7.48 (m, 2H), 7.42-7.38 (m, 2H), 7.26-7.24 (m, 3H), 7.11 (d, J = 7.5 Hz, 1H), 2.18 (s, 3H) ppm; $^{13}C\{^1H\}$ NMR (75 MHz, DMSO-d_6): δ = 184.7, 143.4, 139.9, 138.7, 137.6, 133.6, 131.0, 130.8, 130.3, 129.3, 128.9, 128.8, 127.9, 125.7, 124.8, 124.1, 6.1 ppm. HRMS (ESI$^+$): calcd for $C_{18}H_{14}{}^{79}BrOSSe^+$: $[M+Na]^+$, 458.8928, found: 458.8936; HRMS (ESI$^+$): calcd for $C_{18}H_{14}{}^{81}BrOSSe^+$: $[M+Na]^+$, 460.8908, found: 460.8905.

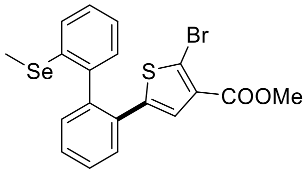

Methyl 2-bromo-5-(2'-(methylselanyl)-[1,1'-biphenyl]-2-yl)thiophene-3-carboxylate (6e)

Purification by column chromatography on silica gel (Petroleum ether/CH_2Cl_2 = 5/1, v/v) afforded the desired product **6e** as a yellow solid (27.9 mg, 30%). ^1H NMR (300 MHz, $CDCl_3$): δ = 7.54-7.51 (m, 1H), 7.40-7.30 (m, 3H), 7.28 (s, 1H), 7.22-7.18 (m, 1H), 7.15-7.11 (m, 2H), 7.02 (d, J = 7.5 Hz, 1H), 3.75 (s, 3H), 2.10 (s, 3H) ppm; $^{13}C\{^1H\}$ NMR (75 MHz, $CDCl_3$): δ = 162.6, 142.8, 140.9, 139.3, 133.9, 132.1, 131.1, 130.5, 130.3, 129.1, 129.0, 128.7, 128.4, 127.6, 125.8, 100.1, 51.9, 6.8 ppm. HRMS (ESI$^+$): calcd for $C_{19}H_{16}{}^{79}BrO_2SSe^+$: $[M+H]^+$, 466.9215, found: 466.9205; HRMS (ESI$^+$): calcd for $C_{19}H_{16}{}^{81}BrO_2SSe^+$: $[M+H]^+$, 468.9194, found: 468.9198.

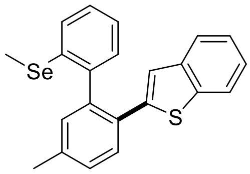

2-(5-Methyl-2'-(methylselanyl)-[1,1'-biphenyl]-2-yl)benzo[*b*]thiophene (6f)

Purification by column chromatography on silica gel (Petroleum ether/CH_2Cl_2 = 20/1, v/v) afforded the desired product **6f** as a yellow solid (35.4 mg, 45%). ^{1}H NMR (300 MHz, $CDCl_3$): δ = 7.43 (d, J = 7.2 Hz, 1H), 7.36 (d, J = 7.8 Hz, 1H), 7.33-7.30 (m, 1H), 7.06-7.00 (m, 3H), 7.01-6.94 (m, 2H), 6.91-6.90 (m, 3H), 6.66 (s, 1H), 2.17 (s, 3H), 1.90 (s, 3H) ppm; ^{13}C{^{1}H} NMR (75 MHz, $CDCl_3$): δ = 143.2, 141.9, 140.3, 140.1, 139.7, 138.0, 133.5, 131.8, 130.8, 130.5, 130.2, 129.3, 128.5, 128.2, 125.6, 124.0, 123.9, 123.5, 122.7, 122.0, 21.3, 6.9 ppm. HRMS (ESI$^+$): calcd for $C_{22}H_{19}SSe^+$: [M+H]$^+$, 395.0368, found: 395.0365.

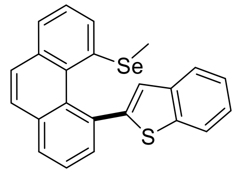

2-(5-(Methylselanyl)phenanthren-4-yl)benzo[*b*]thiophene (6g)

Purification by column chromatography on silica gel (Petroleum ether/CH_2Cl_2 = 20/1, v/v) afforded the desired product **6g** as a yellow solid (32.2 mg, 40%). ^{1}H NMR (300 MHz, $CDCl_3$): δ = 7.84 (dd, J_1 = 7.2 Hz, J_2 = 0.9 Hz,1H), 7.83-7.72 (m, 3H), 7.64-7.60 (m, 3H), 7.55-7.51 (m, 2H), 7.45 (t, J = 7.5 Hz, 1H), 7.27-7.24 (m, 2H), 6.97 (s, 1H), 1.83 (s, 3H) ppm; ^{13}C{^{1}H} NMR (75 MHz, $CDCl_3$) δ = 145.1, 140.0, 139.8, 135.3, 134.8, 134.6, 133.8, 132.7, 132.3, 131.1, 129.6, 127.5, 127.31, 127.3, 127.2, 127.0, 126.3, 124.6, 124.4, 123.9, 123.8, 122.2, 12.8 ppm. HRMS (ESI$^+$): calcd for $C_{23}H_{17}SSe$ $^+$: [M+H]$^+$, 405.0211, found: 405.0208.

V. Scale-up synthesis

A 100 mL Schlenk tube with a magnetic stir bar was charged with thioether- or selenoether-substituted biarlys (**1** or **5**, 2.0 mmol, 1.0 equiv), benzo[*b*]thiophene **2a** (6.0

mmol, 3.0 equiv), $(Cp^*Rh(MeCN)_3[SbF_6]_2$ (3.0 mol%), Ag_2O (7.0 mmol, 3.5 equiv), PivOH (4.0 mmol, 2.0 equiv) and DCE (10.0 mL) under N_2 atmosphere. The resulting mixture was stirred at 120 ºC for 24 h and then diluted with 20 mL of CH_2Cl_2. The solution was filtered through a celite pad and washed with 25-50 mL of CH_2Cl_2. The filtrate was concentrated under vacuum and the residue was purified by column chromatography on silica gel column to provide the desired product.

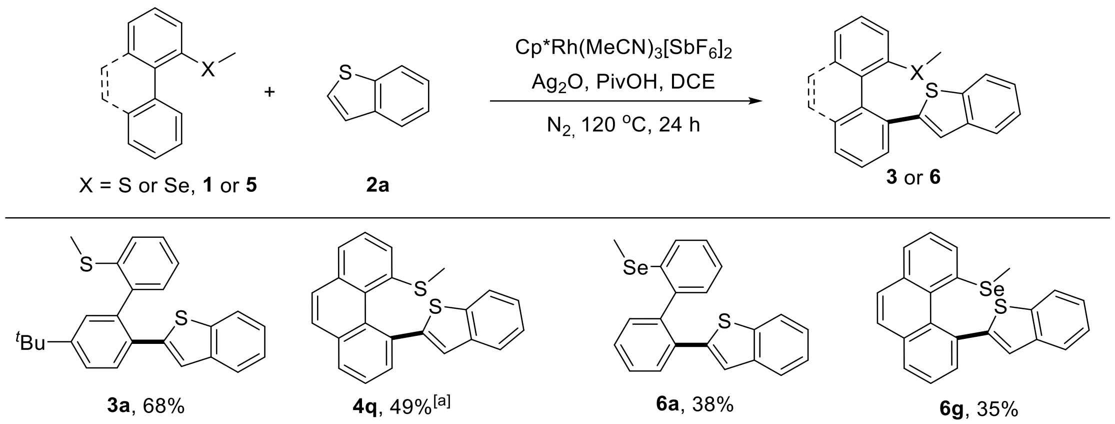

Reaction conditions: **1** or **5** (2.0 mmol, 1.0 equiv), **2a** (6.0 mmol, 3.0 equiv), $Cp^*Rh(MeCN)_3[SbF_6]_2$ (3.0 mol%), Ag_2O (3.5 equiv) and PivOH (2.0 equiv) in DCE (10.0 mL) at 120 °C for 24 h under N_2 atmosphere. [a]. PivOH (1.0 equiv).

Scheme S4. Scale-up synthesis.

VI. General procedure for the recovery experiment of Ag salt

Two 100 mL Schlenk tube with a magnetic stir bar were charged with (3'-(*tert*-butyl)-[1,1'-biphenyl]-2-yl)(methyl)sulfane (**1a**, 2.0 mmol, 1.0 equiv), benzo[*b*]thiophene (**2a**, 6.0 mmol, 3.0 equiv), $(Cp^*Rh(MeCN)_3[SbF_6]_2$ (53 mg, 3 mol%), Ag_2O (1.62 g, 7.0 mmol), PivOH (408 mg, 4.0 mmol) and DCE (10.0 mL) under an N_2 atmosphere at 120 ºC for 24 h and then diluted with 50 mL of CH_2Cl_2.The solution was filtered through a celite pad and washed with 50−60 mL of CH_2Cl_2.The celite pad with the silver residue was dissolved in 100 mL of HNO_3 (2 mol/L). After being stirred for 4 h at room temperature, the 120 mL of NaOH (2 mol/L) was then added. The solution was stirred at room temperature for 2 h. The suspension was filtered, and the solid residue was washed with water (3 × 20 mL) to afford Ag_2O (2.6 g) as a black powder. Since these two reaction tubes were processed simultaneously, the initial mass of Ag_2O added was

3.24 g (1.62 g×2). Subsequently, the recovery rate of Ag_2O was determined to be 80% (2.6 g/3.24 g).

A 25 mL Schlenk tube with a magnetic stir bar was charged with (3'-(*tert*-butyl)-[1,1'-biphenyl]-2-yl)(methyl)sulfane (**1a**, 0.2 mmol, 1.0 equiv), benzo[*b*]thiophene (**2a**, 0.6 mmol, 3.0 equiv), $(Cp^*Rh(MeCN)_3[SbF_6]_2$ (5.3 mg, 3 mol%), regenerated Ag_2O (162.2 mg, 0.7 mmol), PivOH (40.8 mg, 0.4 mmol) and DCE (1.0 mL) under N_2 atmosphere at 120 ºC for 24 h. and then diluted with 10 mL of CH_2Cl_2. The solution was filtered through a celite pad and washed with 10-25 mL of CH_2Cl_2. The filtrate was concentrated under vacuum and the residue was purified by column chromatography on Silica gel column (Petroleum ether/CH_2Cl_2 = 15/1, v/v) to provide **3a** in 73% yield.

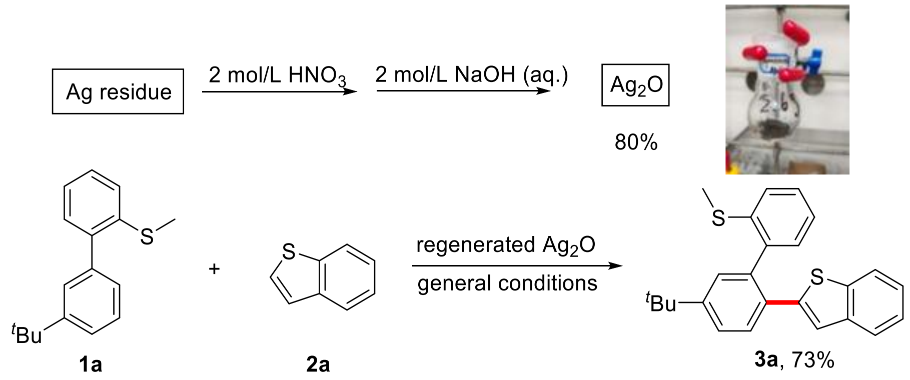

Scheme S5. Recovery experiment of Ag salt.

VII. Mechanistic study

(i) H/D exchange experiment.

A Schlenk tube with a magnetic stir bar was charged with $Cp^*Rh(MeCN)_3[SbF_6]_2$ (5.3 mg, 3 mol%), Ag_2O (162.2 mg, 0.7 mmol), PivOH (40.8 mg, 0.4 mmol), [1,1'-biphenyl]-2-yl(methyl)sulfane **1m** (40.0 mg, 0.2 mmol), D_2O (72.4 μL, 20.0 equiv) and DCE (1.0 mL) under an N_2 atmosphere. The resulting solution was stirred at 120 °C for 4 h. After being cooled to room temperature, the mixture was diluted with 3 mL of CH_2Cl_2. The mixture was filtered through a celite pad and washed with 10-20 mL of CH_2Cl_2. The filtrate was concentrated and the residue was purified by column chromatography on silica gel (Petroleum ether/CH_2Cl_2 = 20/1, v/v) to provide the

desired product. The deuterated ratio was calculated from ^{1}H NMR analysis. The ^{1}H NMR analysis showed that 12% hydrogen at the C2' position of [1,1'-biphenyl]-2-yl(methyl)sulfane **1m** was deuterated.

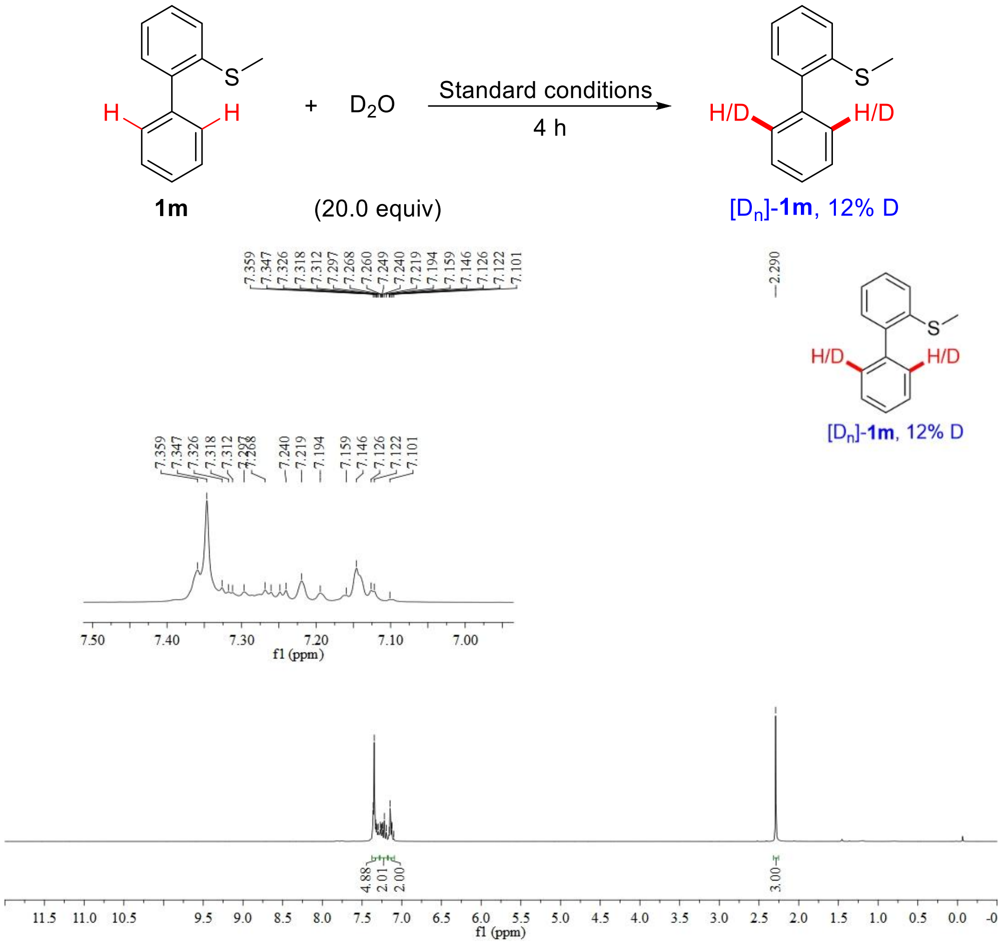

Scheme S6. H/D exchange experiment of the [1,1'-biphenyl]-2-yl(methyl)sulfane.

A Schlenk tube with a magnetic stir bar was charged with $Cp^*Rh(MeCN)_3[SbF_6]_2$ (5.3 mg, 3 mol%), Ag_2O (162.2 mg, 0.7 mmol), PivOH (40.8 mg, 0.4 mmol), benzo[*b*]thiophene **2a** (80.6 mg, 0.6 mmol， 1.0 equiv), D_2O (217.2 μL, 20.0 equiv) and DCE (1.0 mL) under an N_2 atmosphere. The resulting solution was stirred at 120 °C for 4 h. After being cooled to room temperature, the mixture was diluted with 3 mL of CH_2Cl_2. The mixture was filtered through a celite pad and washed with 10-20 mL of CH_2Cl_2. The filtrate was concentrated and the residue was purified by column chromatography on silica gel (Petroleum ether) to provide the desired product. The deuterated ratio was calculated from ^{1}H NMR analysis. The ^{1}H NMR analysis showed that 24% hydrogen at the C2 position of benzo[*b*]thiophene **2a** was deuterated.

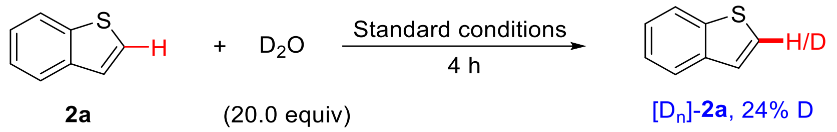

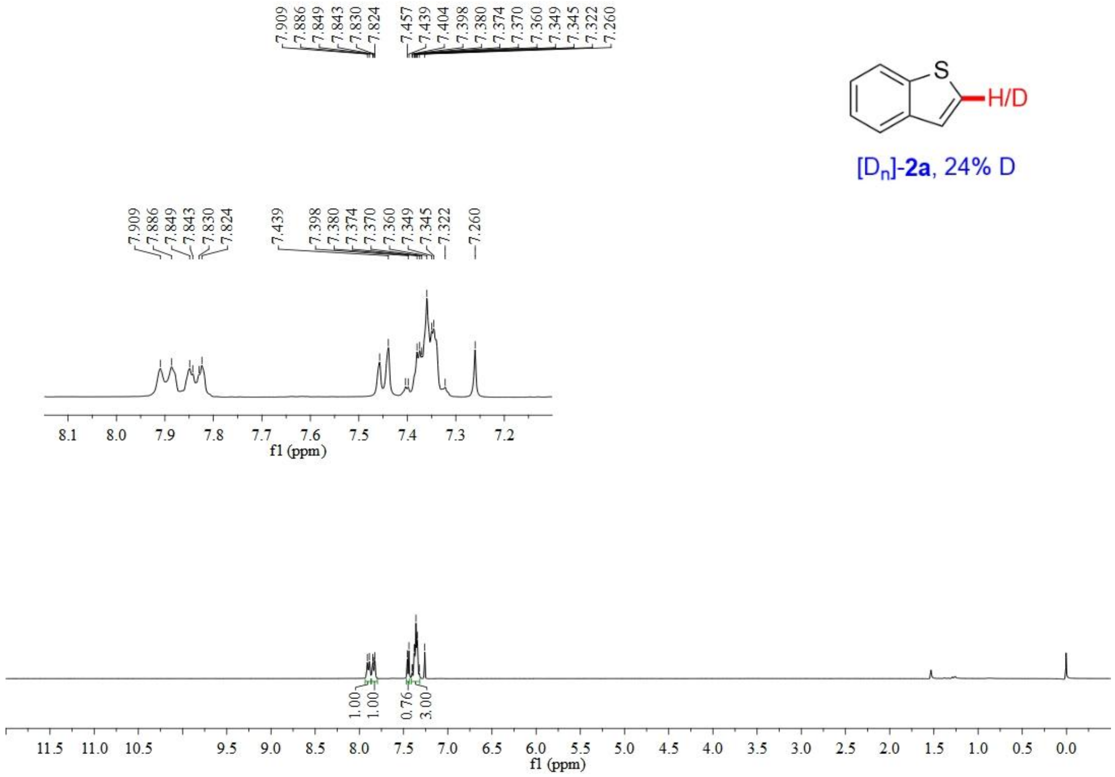

Scheme S7. H/D exchange experiment of the benzo[*b*]thiophene.

A Schlenk tube with a magnetic stir bar was charged with Cp*Rh(MeCN)$_3$[SbF$_6$]$_2$ (5.3 mg, 3 mol%), Ag_2O (162.2 mg, 0.7 mmol), PivOH (40.8 mg, 0.4 mmol), [1,1'-biphenyl]-2-yl(methyl)sulfane **1m** (40.0 mg, 0.2 mmol), benzo[*b*]thiophene **2a** (80.6 mg, 0.6 mmol), D_2O (72.4 μL, 20.0 equiv) and DCE (1.0 mL) under an N_2 atmosphere. The resulting solution was stirred at 120 °C for 4 h. After being cooled to room temperature, the mixture was diluted with 3 mL of CH_2Cl_2. The mixture was filtered through a celite pad and washed with 10-20 mL of CH_2Cl_2. The filtrate was concentrated and the residue was purified by column chromatography on silica gel to provide the desired product. The deuterated ratio was calculated from ^{1}H NMR analysis. The ^{1}H NMR analysis showed that 20% hydrogen at the C2 position of benzo[*b*]thiophene **2a** was deuterated, the ^{1}H NMR analysis showed that 10% hydrogen at the C2' position of [1,1'-biphenyl]-2-yl(methyl)sulfane **1m** was deuterated.

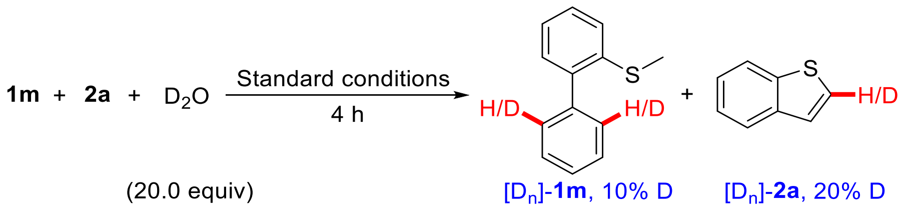

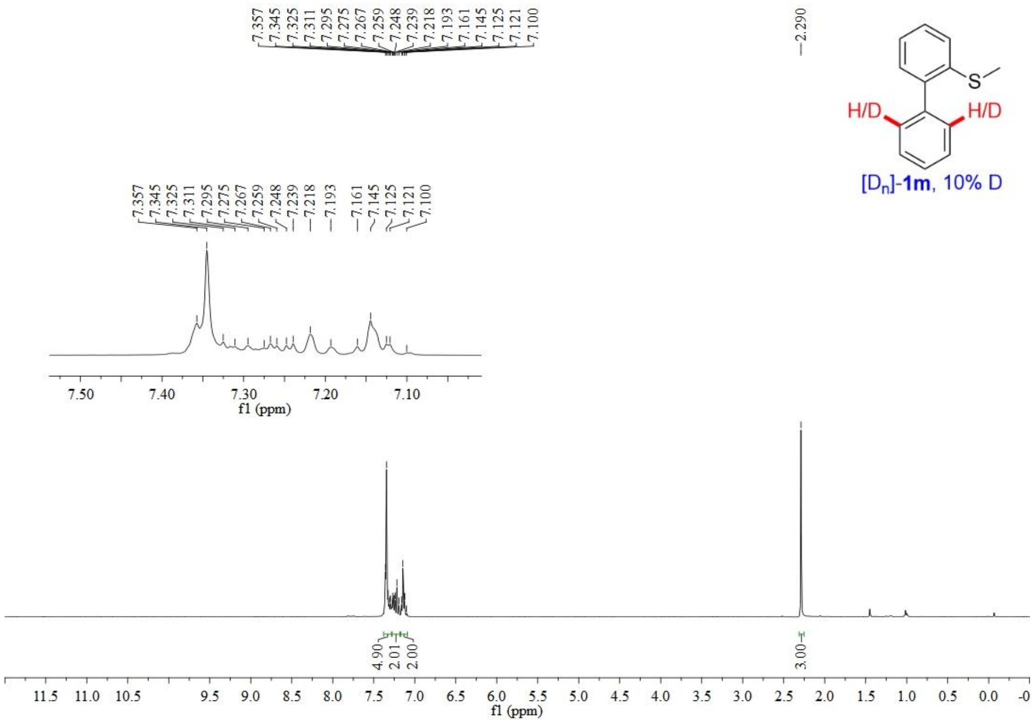

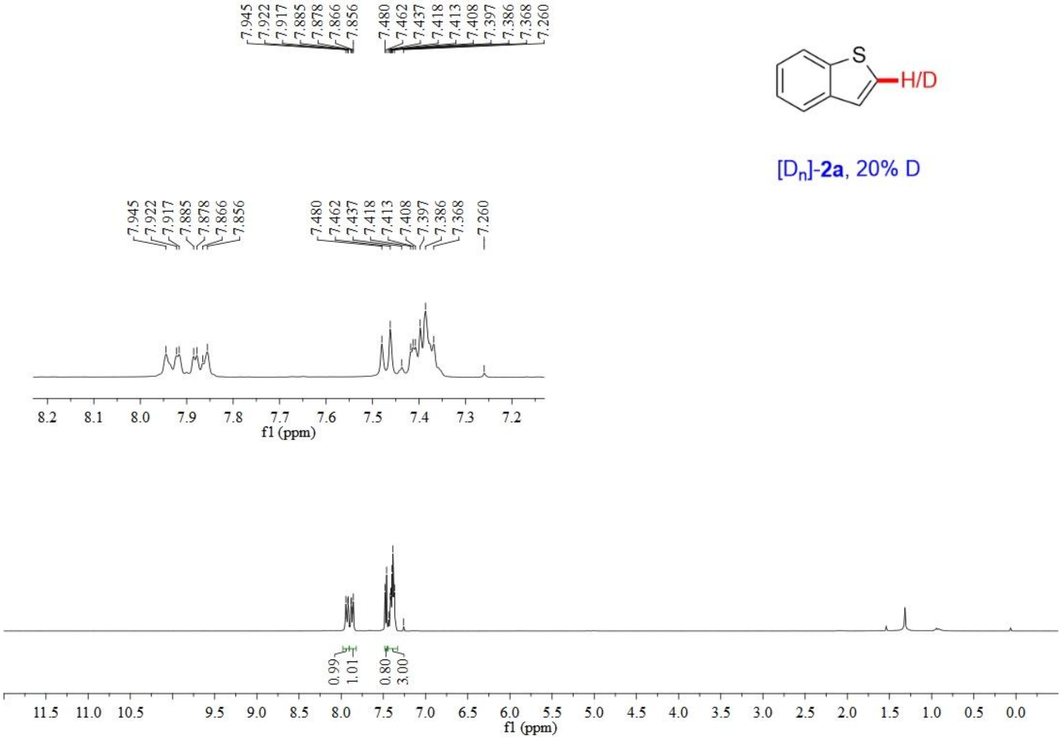

Scheme S8. H/D exchange experiment of the [1,1'-biphenyl]-2-yl(methyl)sulfane and benzo[*b*]thiophene.

(ii) Kinetic isotope experiments.

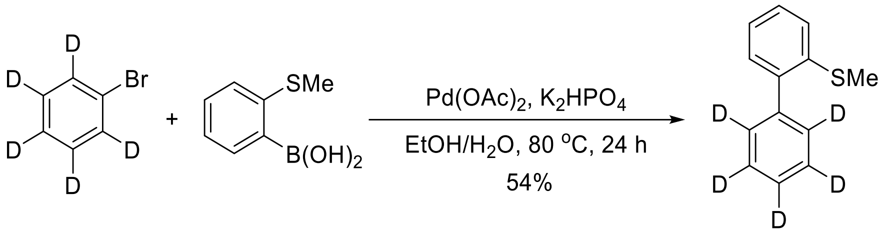
[D$_5$]-1m

Preparation of ([1,1'-biphenyl]-2-yl-2',3',4',5',6'-D$_5$)(methyl)sulfane ([D$_5$]-1m)[6]: Bromobenzene-D$_5$ (2.0 mmol, 1.0 equiv), 2-methylthiophenylboronic acid (4.0 mmol, 2.0 equiv), $Pd(OAc)_2$ (10.0 mol%), K_2HPO_4 (3.0 mmol, 1.5 equiv) was added to a 2-neck round-bottom flask. EtOH (6 mL) and distilled water (6 mL) was added under N_2 atmosphere. The resulting mixture was stirred at 80 °C for 24 h. After cooling to room temperature, then diluted the mixture with EtOAc and water. The aqueous layer was extracted with EtOAc three times. Collected organic layer was dried over anhydrous Na_2SO_4. The solvent was removed and the residue was purified by column chromatography on silica gel, eluting with Petroleum ether/CH_2Cl_2 (20/1, v/v) to the desired product as a light-yellow oil liquid (220.2 mg, 54% yield). ^{1}H NMR (300 MHz, $CDCl_3$): δ = 7.43-7.33 (m, 1H), 7.30-7.28 (m, 1H), 7.23-7.18 (m, 2H), 2.38 (s, 3H) ppm.

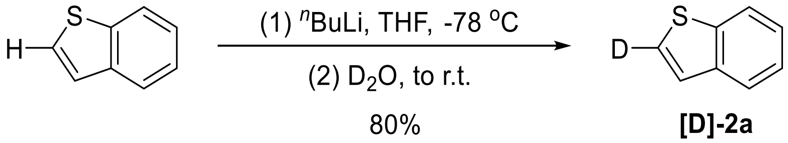
[D]-2a

Preparation of 2-deutero-benzothiophene ([D]-2a)[7]: To a 200 mL two-neck round-bottom flask was added benzo[*b*]thiophene (**2a**, 20.0 mmol, 1.0 equiv) and anhydrous THF (40 mL). The solution was cooled to -78 °C and ^{n}BuLi (2.5 mol/L in THF, 30 mmol, 1.5 equiv) was added dropwise. After stirring for 2 hours at -78 °C, D_2O (9 mL) was added. The mixture was allowed to stir at room temperature overnight, and then saturated aqueous NH_4Cl solution was added. The organic layer was separated, washed with distilled water and aqueous $NaHCO_3$ solution, dried over Na_2SO_4, and concentrated in vacuo, the residue was purified by flash column chromatography on silica gel, eluting with Petroleum ether/EtOAc (20/1, v/v) to afford the desired product

as a white solid (2.15 g, 80% yield). ^{1}H NMR (300 MHz, $CDCl_3$): δ = 7.92-7.90 (m, 1H), 7.86-7.84 (m, 1H), 7.42-7.33 (m, 3H) ppm.

(1) Two separated oven-dried Schlenk tube with a magnetic stir bar was charged with $Cp^*Rh(MeCN)_3[SbF_6]_2$ (5.3 mg, 3 mol%), Ag_2O (162.2 mg, 0.7 mmol), PivOH (40.8 mg, 0.4 mmol), [1,1'-biphenyl]-2-yl(methyl)sulfane **1m** (40.0 mg, 0.2 mmol, 1.0 equiv), benzo[*b*]thiophene **2a** or [D]-**2a** (0.6 mmol, 3.0 equiv) and DCE (1.0 mL) under an N_2 atmosphere. The resulting solution was stirred at 120 °C for specified time (0.5 h, 1.0 h, 1.5 h, 2.0 h). After being cooled to room temperature, the mixture was diluted with 3 mL of CH_2Cl_2. The mixture was filtered through a celite pad and washed with 10-20 mL of CH_2Cl_2. The filtrate was concentrated and the residue was purified by column chromatography on silica gel (Petroleum ether/CH_2Cl_2 = 10/1, v/v) to provide the desired product. The KIE was determined to be k_H/k_D = 0.088/0.092 = 0.96.

Table S6. Kinetic isotope experiments of benzo[*b*]thiophene.

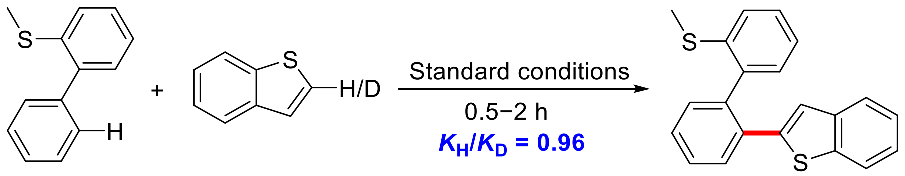

Entry	Time (h)	Yield of **4l**[a]	Yield of **4l**[b]
1	0.5	12	9
2	1	17	17
3	1.5	22	21
4	2	25	23

[a]**2a** (80.6 mg, 0.6 mmol) was used. [b]**[D]-2a** (81.1 mg, 0.6 mmol) was used.

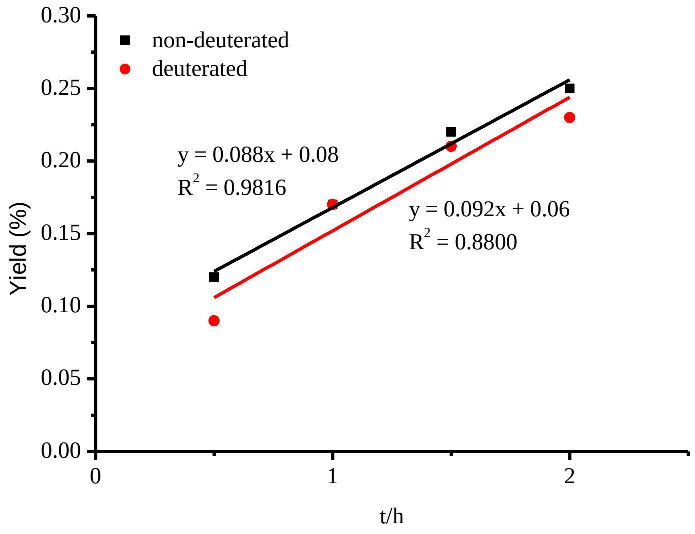

(2) Two separated oven-dried Schlenk tube with a magnetic stir bar was charged with Cp*Rh(MeCN)$_3$[SbF$_6$]$_2$ (5.3 mg, 3 mol %), Ag_2O (162.2 mg, 0.7 mmol), PivOH (40.8 mg, 0.4 mmol), benzo[*b*]thiophene **2a** (80.6 mg, 0.6 mmol), [1,1'-biphenyl]-2-yl(methyl)sulfane **1m** or [D$_5$]-**1m** (0.2 mmol) and DCE (1.0 mL) under an N_2 atmosphere. The resulting solution was stirred at 120 °C for specified time (0.5 h, 1.0 h, 1.5 h, 2.0 h). After being cooled to room temperature, the mixture was diluted with 3 mL of CH_2Cl_2. The mixture was filtered through a celite pad and washed with 10-20 mL of CH_2Cl_2. The filtrate was concentrated and the residue was purified by column chromatography on silica gel (Petroleum ether/CH_2Cl_2 = 10/1, v/v) to provide the desired product. The KIE was determined to be $k_H/k_D = 0.088/0.068 = 1.29$.

Table S7. Kinetic isotope experiments of [1,1'-biphenyl]-2-yl(methyl)sulfane.

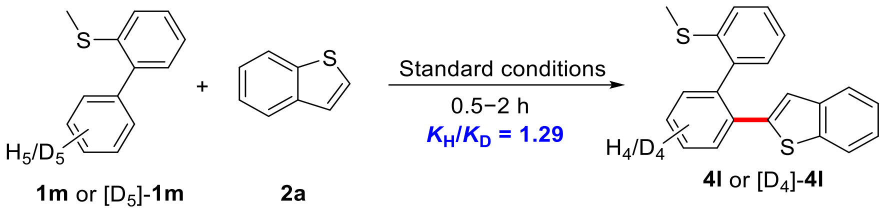

Entry	Time (h)	Yield of **4l**[a]	Yield of [D$_4$]-**4l**[b]
1	0.5	12	7
2	1	17	11
3	1.5	22	15
4	2	25	17

[a]**1m** (40.1 mg, 0.2 mmol) was used. [b][D$_5$]-**1m** (41.1 mg, 0.2 mmol) was used.

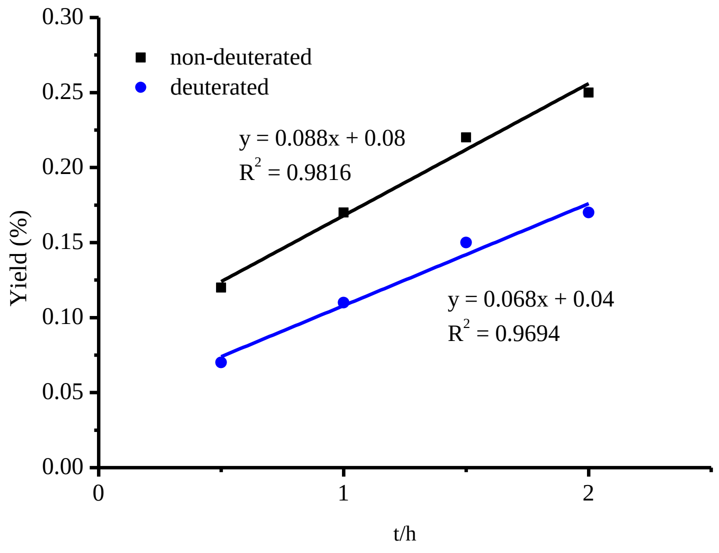

VIII. Synthetic applications

(i) The thioether- and selenoether-substituted biheteroaryl compounds (1.0 mmol, 1.0 equiv) and *m*-CPBA (85%, 1.1 mmol, 1.1 equiv, 223.3 mg) were dissolved in CH_2Cl_2 (10 mL) at 0 ºC. The mixture was stirred at room temperature overnight. Saturated aqueous $NaHCO_3$ solution was added, and the organic layer was separated. The aqueous layer was extracted with CH_2Cl_2. Combined organic extract was dried over Na_2SO_4. After evaporation of the solvents, the mixture was placed in a dried Schlenk tube and DCE (15.0 mL) was added. With continuous N_2 streaming into the tube, TfOH (7.5 mL) was added dropwise. After stirring 24 h at room temperature, distilled water (27 mL) and pyridine (7 mL) were charged, and the resulting mixture was stirred overnight at 120 °C. The mixture was poured into 25 mL of 4 M aqueous HCl and diluted with distilled water and CH_2Cl_2. The organic layer was separated, and the aqueous layer was extracted with CH_2Cl_2. The combined organic extracts were washed with distilled water and brine, dried over Na_2SO_4, and concentrated in vacuo. The residue was purified by column chromatography on silica gel (Petroleum ether/CH_2Cl_2) to provide the desired product.

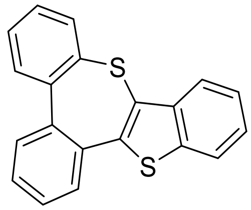

Dibenzo[*b*,*d*]benzo[4,5]thieno[2,3-*f*]thiepine (7a)

Purification by column chromatography on silica gel (Petroleum ether/CH_2Cl_2 = 30/1, v/v) afforded the desired product **7a** as a yellow solid (218.1 mg, 69%). ^{1}H NMR (300 MHz, $CDCl_3$): δ = 8.11 (d, J = 8.1 Hz, 1H), 7.82 (d, J = 7.8 Hz, 1H), 7.75-7.72 (m, 1H), 7.67-7.61 (m, 2H), 7.54-7.51 (m, 2H), 7.50-7.45 (m, 2H), 7.39-7.34 (m, 2H), 7.30 (dd, J_1 = 7.5 Hz, J_2 = 1.8 Hz, 1H) ppm; ^{13}C{^{1}H} NMR (75 MHz, $CDCl_3$): δ = 143.9, 142.6, 141.2, 139.7, 139.4, 139.2, 134.0, 132.5, 132.0, 131.4, 130.5, 128.9, 128.8, 128.4, 128.3, 128.1, 125.1, 124.9, 123.0, 122.6 ppm. HRMS (ESI$^+$): calcd for $C_{20}H_{13}S_2^+$: $[M+H]^+$, 317.0454, found: 317.0459.

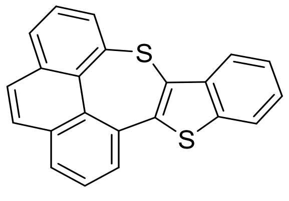

Benzo[4,5]thieno[2,3-*f*]phenanthro[4,5-*bcd*]thiepine (**7b**)

Purification by column chromatography on silica gel (Petroleum ether/CH_2Cl_2 = 30/1, v/v) afforded the desired product **7b** as a yellow solid (115.9 mg, 34%). ^{1}H NMR (300 MHz, $CDCl_3$): δ = 8.19 (d, J = 8.1 Hz, 1H), 8.00-7.97 (m, 2H), 7.92 (dd, J_1 = 7.2 Hz, J_2 = 0.9 Hz, 1H), 7.86-7.75 (m, 4H), 7.73-7.67 (m, 1H), 7.59-7.51 (m, 2H), 7.39 (t, J = 8.1 Hz, 1H) ppm; ^{13}C{^{1}H} NMR (75 MHz, $CDCl_3$): δ = 144.2, 140.9, 139.1, 134.7, 134.6, 133.8, 133.4, 132.6, 132.2, 130.4, 129.1, 128.9, 128.3, 128.0, 127.7, 126.6, 126.2, 125.0, 124.96, 123.1, 122.4 ppm. HRMS (ESI$^+$): calcd for $C_{22}H_{13}S_2^+$: [M+H]$^+$, 341.0454, found: 341.0451.

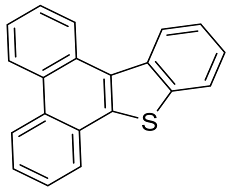

Benzo[*b*]phenanthro[9,10-*d*]thiophene (**8a**)

Purification by column chromatography on silica gel (Petroleum ether/CH_2Cl_2 = 30/1, v/v) afforded the desired product **8a** as a yellow solid (76.7 mg, 27%). ^{1}H NMR (300 MHz, $CDCl_3$): δ = 8.74 (d, J = 8.4 Hz, 1H), 8.55 (d, J = 8.1 Hz, 1H), 8.50 (d, J = 8.1 Hz, 1H), 8.44-8.41 (m, 1H), 7.93-7.90 (m, 1H), 7.79 (d, J = 7.8 Hz, 1H), 7.55-7.50 (m, 1H), 7.46-7.41 (m, 3H), 7.39-7.29 (m, 2H) ppm; ^{13}C{^{1}H} NMR (75 MHz, $CDCl_3$): δ = 139.3, 138.3, 137.6, 129.9, 129.6, 129.4, 127.9, 127.22, 127.19, 127.18, 125.5, 125.1, 125.0, 124.9, 124.7, 123.9, 123.8, 123.3, 123.2 ppm. HRMS (ESI$^+$): calcd for $C_{20}H_{13}S^+$: [M+H]$^+$, 285.0733, found: 285.0730.

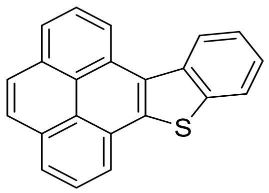

Benzo[*b*]pyreno[4,5-*d*]thiophene (**8b**)

Purification by column chromatography on silica gel (Petroleum ether/CH_2Cl_2 = 30/1, v/v) afforded the desired product **8b** as a yellow solid (138.7 mg, 45%). ^{1}H NMR (300 MHz, $CDCl_3$): δ = 8.10 (d, J = 8.1 Hz, 1H), 8.03 (dd, J_1 = 7.5 Hz, J_2 = 1.5 Hz, 1H), 7.99-7.94 (m, 2H), 7.87-7.65 (m, 5H), 7.51 (t, J = 7.5 Hz, 2H),7.38 (t, J = 7.2 Hz, 1H) ppm; ^{13}C{^{1}H} NMR (75 MHz, $CDCl_3$): δ = 144.0, 141.1, 139.9, 134.6, 134.5, 134.1, 133.6, 133.4, 130.9, 129.5, 128.9, 128.8, 128.3, 128.1, 127.9, 127.6, 126.3, 124.9, 124.8, 123.9, 122.1, 121.0 ppm. HRMS (ESI$^+$): calcd for $C_{22}H_{13}S^+$: [M+H]$^+$, 309.0733, found: 309.0721.

(ii) A 25 mL Schlenk tube with a magnetic stir bar was charged with dibenzo[*b,d*]benzo[4,5]thieno[2,3-*f*]thiepine (**7a**) (0.5 mmol, 158.2 mg), *m*-CPBA (85%) and CH_2Cl_2 (5.0 mL). The resulting mixture was stirred at 0 °C and then at room temperature for 24 h. The filtrate was concentrated under vacuum and the residue was purified by column chromatography on silica gel (Petroleum ether/EtOAc) to provide the desired product.

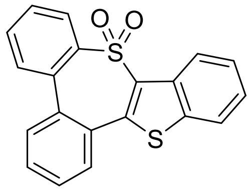

Dibenzo[*b,d*]benzo[4,5]thieno[2,3-*f*]thiepine 9,9-dioxide (9)

Compound **9** was synthesized by **7a** (0.5 mmol, 158.2 mg) and *m*-CPBA (85%, 1.1 mmol, 2.2 equiv, 223.3 mg). Purification by column chromatography on silica gel (Petroleum ether/EtOAc = 5/1, v/v) afforded the desired product **9** as a yellow solid (142.7 mg, 82%). ^{1}H NMR (300 MHz, $CDCl_3$): δ = 8.67-8.64 (m, 1H), 8.15 (d, J = 5.1 Hz, 1H), 7.70-7.65 (m, 3H), 7.53-7.41 (m, 5H), 7.38-7.36 (m, 1H), 7.30-7.28 (m, 1H) ppm; ^{13}C{^{1}H} NMR (75 MHz, $CDCl_3$): δ = 147.9, 143.1, 138.6, 138.0, 136.4, 135.6, 134.5, 132.8, 132.5, 131.1, 131.0, 130.4, 129.8, 129.1, 128.5, 126.1, 126.0, 125.3, 124.3, 122.0 ppm. HRMS (ESI$^+$): calcd for $C_{22}H_{13}S_2O_2^+$: [M+H]$^+$, 349.0352, found: 349.0357.

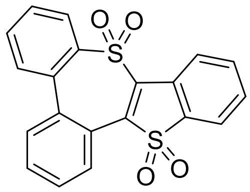

Dibenzo[*b*,*d*]benzo[4,5]thieno[2,3-*f*]thiepine 9,9,14,14-tetraoxide (10)

Compound **10** was synthesized by **7a** (0.5 mmol, 158.2 mg) and *m*-CPBA (85%, 5.0 equiv, 2.5 mmol, 507.6 mg). Purification by column chromatography on silica gel (Petroleum ether/EtOAc = 2/1, v/v) afforded the desired product **10** as a yellow solid (171.2 mg, 90%). ^{1}H NMR (300 MHz, $CDCl_3$): δ = 8.61-8.54 (m, 2H), 7.28 (d, J = 7.8 Hz, 1H), 7.90 (dd, J_1 = 7.5 Hz, J_2 = 1.8 Hz, 1H), 7.79-7.76 (m, 3H),7.74-7.66 (m, 4H), 7.57 (t, J = 7.5 Hz, 1H) ppm; ^{13}C{^{1}H} NMR (75 MHz, $CDCl_3$): δ = 141.7, 141.3, 140.6, 138.6, 137.0, 136.2, 134.6, 134.2, 133.4, 131.7, 131.4, 131.1, 129.4, 129.2, 126.9, 126.8, 126.5, 125.6, 125.5, 122.5 ppm. HRMS (ESI$^+$): calcd for $C_{20}H_{13}S_2O_4^+$: [M+H]$^+$, 380.0177, found: 380.0170.

IX. Photophysical properties

Table S8. Photophysical data of sulfur-embedded polycyclic aromatics in CH_2Cl_2.

Compound	**7a**	**7b**	**8a**	**8b**	**9**	**10**
$\lambda_{abs, max}$ (nm)[a]	306	304	318	354	304	354
$\lambda_{em, max}$ (nm)[b]	360, 384, 500	392, 532	358, 386	388, 412, 432	380	434

[a]Absorption maxima in CH_2Cl_2 at 1×10^{-5} mol/L. [b]Emission maxima in CH_2Cl_2 at 1×10^{-5} mol/L.

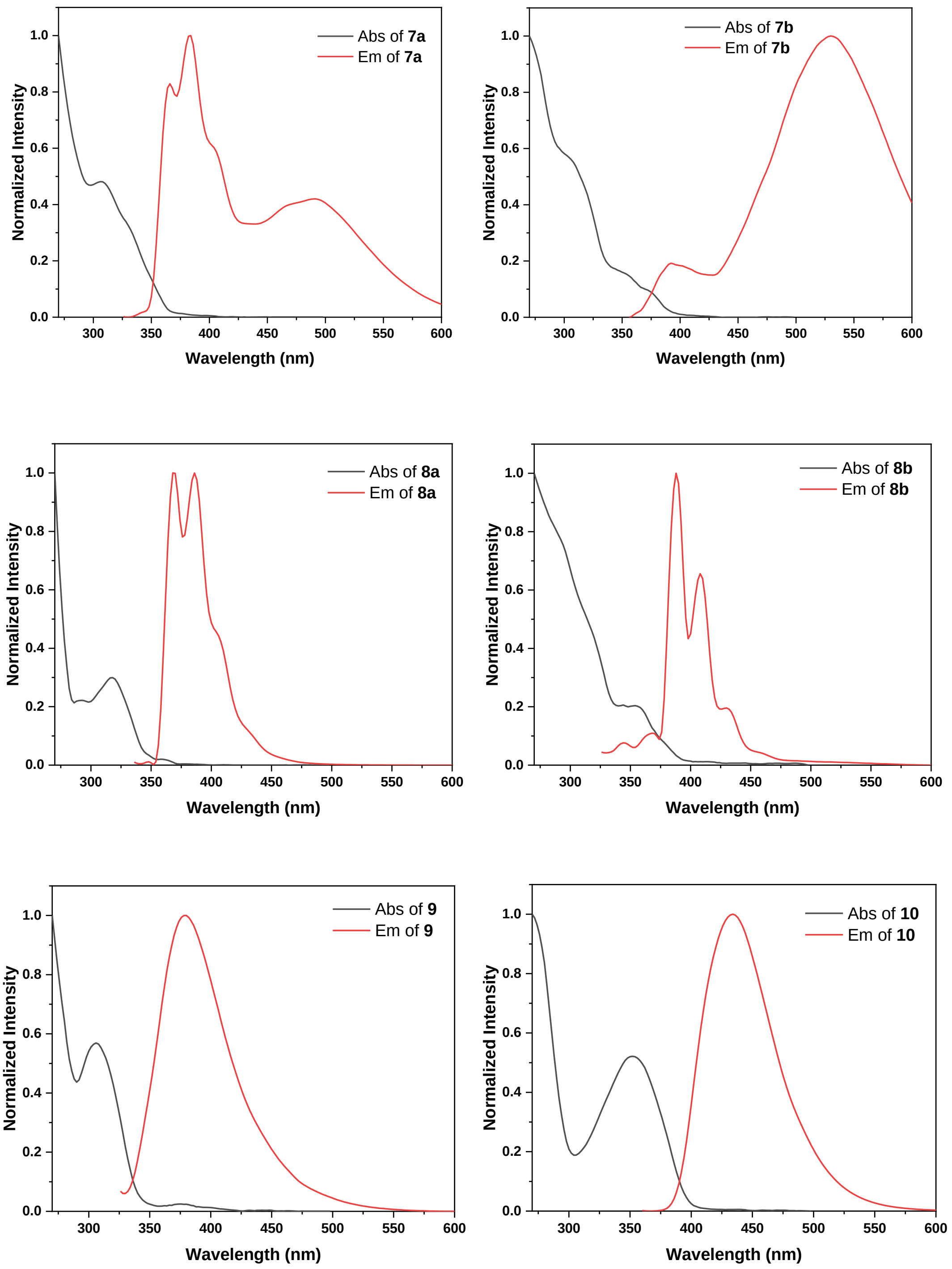

Figure S1. Absorption and fluorescence emission spectra of sulfur-embedded polycyclic aromatics. Absorption maxima in CH_2Cl_2 at 1×10^{-5} mol/L. Emission maxima in CH_2Cl_2 at 1×10^{-5} mol/L.

Table S9. TD-DFT calculation results of 7a.

Excited State	Energy (eV)	Wavelength (nm)	Oscillator strength	Configurations
**1**	3.5975	344.64	0.0431	H-2 -> L 0.16900<br>H -> L 0.66472<br>H -> L+1 0.12448
**2**	4.0092	309.25	0.0158	H-2 -> L -0.17397<br>H -> L+1 0.66763
**3**	4.2390	292.48	0.0606	H-2 -> L 0.37028<br>H-1 -> L 0.54661<br>H -> L+3 -0.11917
**4**	4.2939	288.74	0.2025	H-3 -> L+1 -<br>0.10034<br>H-2 -> L 0.51462<br>H-1 -> L -0.37729<br>H -> L -0.15466<br>H -> L+1 0.12347<br>H -> L+4 -0.10013
**5**	4.3783	283.18	0.0026	H-3 -> L -0.12854<br>H -> L+2 0.66846

Table S10. TD-DFT calculation results of 7b.

Excited State	Energy (eV)	Wavelength (nm)	Oscillator strength	Configurations
**1**	3.3221	373.21	0.0314	H-1 -> L -0.19272<br>H -> L 0.63514<br>H -> L+1 -0.19162
**2**	3.5066	353.58	0.0036	H-1 -> L -0.21761<br>H -> L 0.12888<br>H -> L+1 0.64357
**3**	3.7395	331.55	0.0734	H-4 -> L+1 -0.13388<br>H-2 -> L 0.16594<br>H-2 -> L+1 0.12985<br>H-1 -> L 0.50137<br>H-1 -> L+1 -0.28354<br>H -> L 0.24434<br>H -> L+1 0.17075

**4**	3.9262	315.78	0.0336	H-4 -> L -0.13774<br>H-2 -> L 0.43795<br>H-1 -> L -0.32769<br>H-1 -> L+1 -0.39434
**5**	4.0994	302.44	0.1655	H-4 -> L 0.17726<br>H-2 -> L 0.47255<br>H-1 -> L+1 0.40761<br>H -> L+2 -0.20697

Table S11. TD-DFT calculation results of 8a.

**Excited State**	**Energy (eV)**	**Wavelength (nm)**	**Oscillator strength**	**Configurations**
**1**	3.7257	332.78	0.0214	H-2 -> L -0.10972<br>H-1 -> L -0.29397<br>H-1 -> L+1 -0.14856<br>H -> L -0.36539<br>H -> L+1 0.48145
**2**	3.8201	324.56	0.0593	H-1 -> L -0.11069<br>H-1 -> L+1 0.14869<br>H -> L 0.54223<br>H -> L+1 0.38234
**3**	4.0975	302.59	0.1669	H-2 -> L -0.15131<br>H-1 -> L 0.56158<br>H-1 -> L+1 -0.11090<br>H -> L+1 0.25224<br>H -> L+2 -0.24006
**4**	4.2479	291.87	0.0698	H-2 -> L 0.32797<br>H-2 -> L+1 -0.17639<br>H-1 -> L 0.10287<br>H-1 -> L+1 0.52550<br>H -> L -0.15065<br>H -> L+10.15879
**5**	4.5305	273.66	0.0431	H-2 -> L 0.55754<br>H-2 -> L+1 0.10974<br>H-1 -> L+1 -0.30982<br>H -> L+1 0.12120<br>H -> L+2 -0.12029<br>H -> L+3 0.16980

Table S12. TD-DFT calculation results of 8b.

Excited State	Energy (eV)	Wavelength (nm)	Oscillator strength	Configurations
**1**	3.4407	360.35	0.1666	H-1 -> L -0.21465<br>H-1 -> L+1 0.11666<br>H-> L 0.63464<br>H -> L+1 0.12133
**2**	3.5781	346.51	0.0849	H-2 -> L -0.15942<br>H-2 -> L -0.15942<br>H -> L 0.22709<br>H -> L+1 -0.30412
**3**	3.8407	322.81	0.0892	H-3 -> L -0.10239<br>H-2 -> L 0.40090<br>H-1 -> L 0.34605<br>H -> L+1 0.43584
**4**	4.1271	300.41	0.0140	H-3 -> L -0.43822<br>H-2 -> L -0.24722<br>H -> L+2 0.47969
**5**	4.1826	296.43	0.1678	H-3 -> L 0.15492<br>H-2 -> L 0.40134<br>H-1 -> L+1 0.16593<br>H -> L+1 -0.34317<br>H -> L+2 0.35960

Table S13. TD-DFT calculation results of 9.

Excited State	Energy (eV)	Wavelength (nm)	Oscillator strength	Configurations
**1**	3.9489	313.97	0.0902	H-1 -> L 0.15291<br>H-1 -> L+1 -0.13695<br>H -> L 0.63879<br>H -> L+1 0.16762
**2**	4.0594	305.42	0.0808	H-2 -> L 0.17487<br>H-1 -> L 0.40733<br>H -> L -0.22563<br>H -> L+1 0.48654
**3**	4.1642	297.74	0.1650	H-2 -> L -0.20902<br>H-1 -> L 0.52227

				H -> L+1 -0.38177
**4**	4.3499	285.03	0.0417	H-2 -> L -0.35527<br>H-1 -> L+1 0.54257<br>H -> L+1 0.23237
**5**	4.5551	272.19	0.0928	H-2 -> L 0.48458<br>H-2 -> L+1 -0.17875<br>H-1 -> L+1 0.40882<br>H -> L+1 -0.15806

Table S14. TD-DFT calculation results of 10.

**Excited State**	**Energy (eV)**	**Wavelength (nm)**	**Oscillator strength**	**Configurations**
**1**	3.5002	354.22	0.1658	H -> L 0.69380
**2**	3.7077	334.40	0.0124	H-1 -> L 0.68975<br>H -> L+1 0.11849
**3**	4.1441	299.18	0.0243	H-6 -> L 0.11199<br>H-4 -> L -0.12875<br>H-3 -> L 0.12691<br>H-2 -> L 0.63708<br>H -> L+2 0.13816
**4**	4.2864	289.25	0.0038	H-6 -> L 0.25755<br>H-5 -> L -0.14877<br>H-4 -> L -0.39471<br>H-3 -> L 0.42249<br>H-2 -> L -0.18956<br>H-2 -> L -0.18956
**5**	4.3803	283.05	0.0042	H-4 -> L -0.39592<br>H-3 -> L -0.38922<br>H -> L+1 0.40347

X. Single crystal X-ray structure and crystallographic data

The single crystals of compounds **4l** (2281727), **7a** (2433068), **7b** (2475673), **8a** (2433061), **8b** (2433067), **9** (2433062) and **10** (2433063) were obtained from solvent diffusion method with CH_2Cl_2 and hexane.

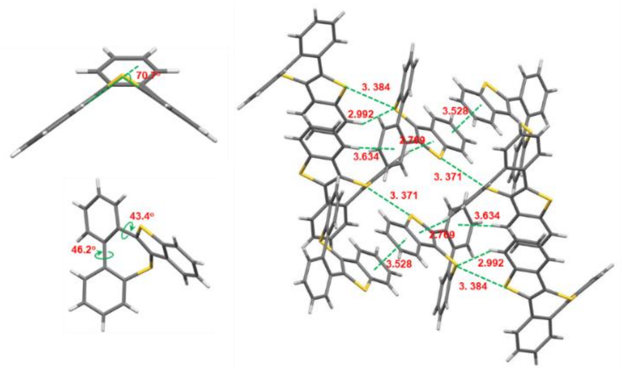

Figure S2. Crystal X-ray structure and packing pattern of **7a**.

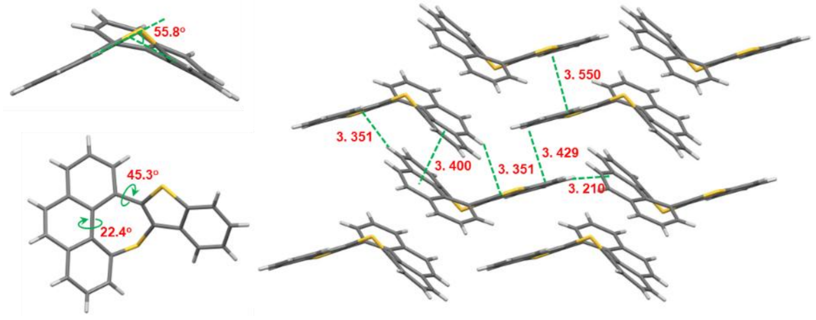

Figure S3. Crystal X-ray structure and packing pattern of **7b**.

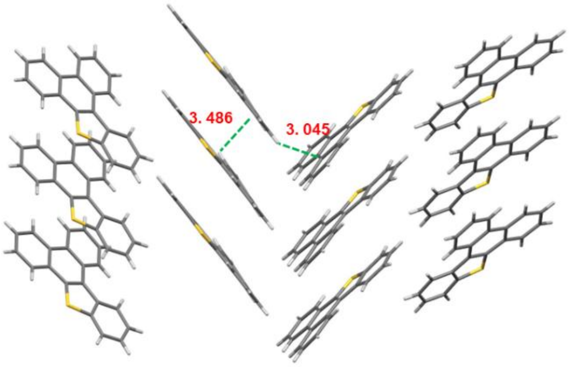

Figure S4. Crystal X-ray structure and packing pattern of **8a**.

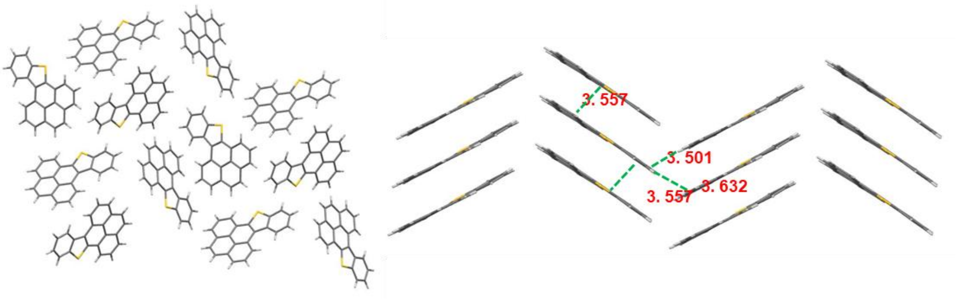

Figure S5. Crystal X-ray structure and packing pattern of **8b**.

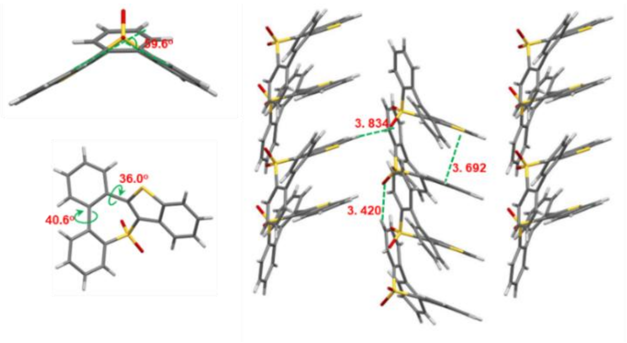

Figure S6. Crystal X-ray structure and packing pattern of **9**.

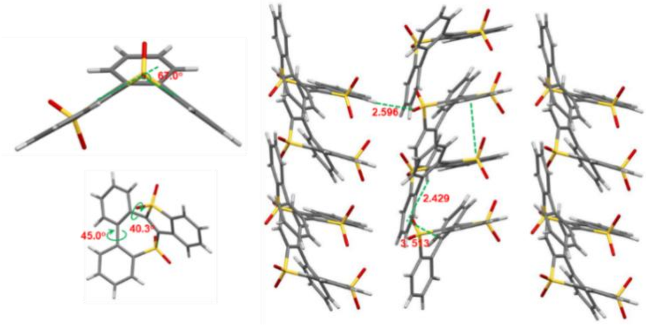

Figure S7. Crystal X-ray structure and packing pattern of **10**.

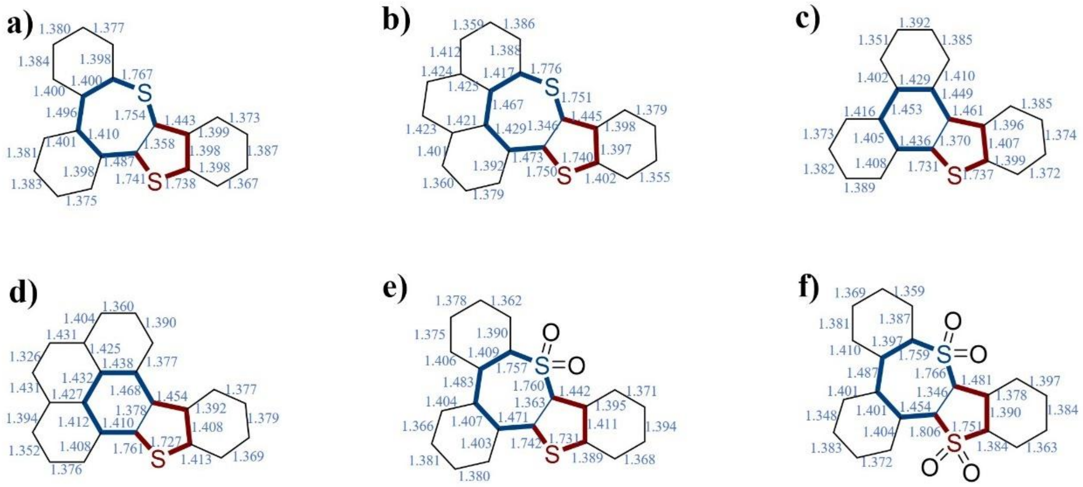

Figure S8. Bond length in angstroms(blue) determined by X-ray crystallography of a) **7a**, b) **7b**, c) **8a**, d) **8b**, e) **9**, and f) **10**.

Table S15. Crystal data and structure refinement for **4l**.

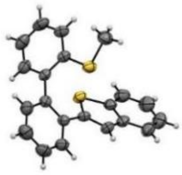

Identification code	**4l**
Empirical formula	$C_{21}H_{16}S_2$
Formula weight	332.46
Temperature/K	293(2)
Crystal system	orthorhombic
Space group	Pbca
a/Å	10.7590(2)
b/Å	15.6784(3)
c/Å	20.0594(4)
α/°	90
β/°	90
γ/°	90
Volume/Å^3	3383.70(11)
Z	8
ρ_{calc}g/cm^3	1.305
μ/mm^{-1}	2.799
F(000)	1392.0
Crystal size/mm^3	0.2 × 0.18 × 0.15
Radiation	Cu Kα (λ = 1.54184)
2Θ range for data collection/°	8.816 to 136.316
Index ranges	-12 ≤ h ≤ 10, -18 ≤ k ≤ 18, -24 ≤ l ≤ 24
Reflections collected	11547
Independent reflections	3071 [R_{int} = 0.0523, R_{sigma} = 0.0356]
Data/restraints/parameters	3071/0/210
Goodness-of-fit on F^2	1.100
Final R indexes [I>=2σ (I)]	R_1 = 0.0570, wR_2 = 0.1651
Final R indexes [all data]	R_1 = 0.0626, wR_2 = 0.1711
Largest diff. peak/hole / e Å^{-3}	0.46/-0.39

Table S16. Crystal data and structure refinement for **7a**.

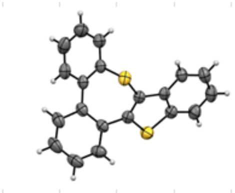

Identification code	**7a**
Empirical formula	$C_{20}H_{12}S_2$
Formula weight	316.42
Temperature/K	293(2)
Crystal system	triclinic
Space group	P-1
a/Å	9.7309(3)
b/Å	11.2753(6)
c/Å	14.8333(3)
α/°	106.545(4)
β/°	90.845(2)
γ/°	103.498(4)
Volume/Å3	1511.24(11)
Z	4
ρcalcg/cm3	1.391
μ/mm-1	3.109
F(000)	656.0
Crystal size/mm3	0.3 × 0.25 × 0.06
Radiation	Cu Kα (λ = 1.54184)
2Θ range for data collection/°	8.444 to 136.466
Index ranges	-11 ≤ h ≤ 11, -13 ≤ k ≤ 13, -17 ≤ l ≤ 17
Reflections collected	9924
Independent reflections	9924 [R_{int} = ?, R_{sigma} = 0.0201]
Data/restraints/parameters	9924/0/398
Goodness-of-fit on F2	1.270
Final R indexes [I>=2σ (I)]	R_1 = 0.0993, wR_2 = 0.2737
Final R indexes [all data]	R_1 = 0.1036, wR_2 = 0.2849
Largest diff. peak/hole / e Å-3	0.96/-0.64

Table S17. Crystal data and structure refinement for **7b**.

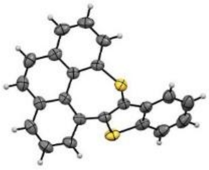

Identification code	**7b**
Empirical formula	$C_{22}H_{12}S_2$
Formula weight	340.44
Temperature/K	297.00
Crystal system	triclinic
Space group	P-1
a/Å	7.9692(3)
b/Å	9.1737(3)
c/Å	11.3045(4)
α/°	97.109(2)
β/°	101.458(2)
γ/°	95.919(2)
Volume/Å3	796.78(5)
Z	1
ρcalcg/cm3	1.419
μ/mm-1	2.993
F(000)	352.0
Crystal size/mm3	0.2 × 0.18 × 0.15
Radiation	CuKα (λ = 1.54184)
2Θ range for data collection/°	8.068 to 133.342
Index ranges	-9 ≤ h ≤ 9, -10 ≤ k ≤ 10, -13 ≤ l ≤ 13
Reflections collected	8332
Independent reflections	2787 [R_{int} = 0.0594, R_{sigma} = 0.0602]
Data/restraints/parameters	2787/0/217
Goodness-of-fit on F2	1.033
Final R indexes [I>=2σ (I)]	R_1 = 0.0505, wR_2 = 0.1403
Final R indexes [all data]	R_1 = 0.0621, wR_2 = 0.1465
Largest diff. peak/hole / e Å-3	0.39/-0.48

Table S18. Crystal data and structure refinement for **8a**.

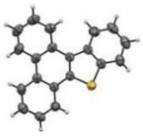

Identification code	**8a**
Empirical formula	$C_{20}H_{12}S$
Formula weight	284.36
Temperature/K	293
Crystal system	orthorhombic
Space group	P212121
a/Å	5.2323(2)
b/Å	8.9668(4)
c/Å	29.1269(10)
α/°	90
β/°	90
γ/°	90
Volume/Å3	1366.55(9)
Z	4
ρcalcg/cm3	1.382
μ/mm-1	1.983
F(000)	592.0
Crystal size/mm3	0.15 × 0.1 × 0.08
Radiation	Cu Kα (λ = 1.54184)
2Θ range for data collection/°	10.322 to 135.898
Index ranges	-6 ≤ h ≤ 4, -10 ≤ k ≤ 10, -35 ≤ l ≤ 35
Reflections collected	4479
Independent reflections	2178 [R_{int} = 0.0411, R_{sigma} = 0.0517]
Data/restraints/parameters	2178/0/190
Goodness-of-fit on F2	1.033
Final R indexes [I>=2σ (I)]	R_1 = 0.0487, wR_2 = 0.1300
Final R indexes [all data]	R_1 = 0.0520, wR_2 = 0.1341
Largest diff. peak/hole / e Å-3	0.33/-0.29
Flack parameter	-0.017(18)

Table S19. Crystal data and structure refinement for **8b**.

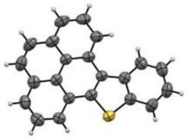

Identification code	**8b**
Empirical formula	$C_{22}H_{12}S$
Formula weight	308.38
Temperature/K	293(2)
Crystal system	orthorhombic
Space group	P212121
a/Å	4.7483(2)
b/Å	15.0591(5)
c/Å	20.1810(7)
α/°	90
β/°	90
γ/°	90
Volume/Å3	1443.04(9)
Z	4
ρcalcg/cm3	1.419
μ/mm-1	1.928
F(000)	640.0
Crystal size/mm3	0.2 × 0.15 × 0.12
Radiation	CuKα (λ = 1.54178)
2Θ range for data collection/°	7.324 to 133.334
Index ranges	-4 ≤ h ≤ 5, -17 ≤ k ≤ 16, -23 ≤ l ≤ 24
Reflections collected	9014
Independent reflections	2454 [R_{int} = 0.0601, R_{sigma} = 0.0485]
Data/restraints/parameters	2454/0/208
Goodness-of-fit on F2	1.019
Final R indexes [I>=2σ (I)]	R_1 = 0.0441, wR_2 = 0.1128
Final R indexes [all data]	R_1 = 0.0525, wR_2 = 0.1173
Largest diff. peak/hole / e Å-3	0.48/-0.20
Flack parameter	0.018(16)

Table S20. Crystal data and structure refinement for **9**.

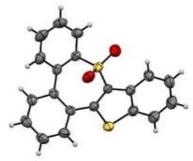

Identification code	**9**
Empirical formula	$C_{20}H_{12}O_2S_2$
Formula weight	348.42
Temperature/K	273.15
Crystal system	orthorhombic
Space group	Pbca
a/Å	7.8957(4)
b/Å	16.3997(7)
c/Å	24.1206(10)
α/°	90
β/°	90
γ/°	90
Volume/Å3	3123.3(2)
Z	8
ρcalcg/cm3	1.482
μ/mm-1	3.164
F(000)	1440.0
Crystal size/mm3	0.2 × 0.15 × 0.12
Radiation	CuKα (λ = 1.54178)
2Θ range for data collection/°	7.33 to 133.174
Index ranges	-9 ≤ h ≤ 9, -19 ≤ k ≤ 18, -28 ≤ l ≤ 27
Reflections collected	19098
Independent reflections	2746 [R_{int} = 0.0582, R_{sigma} = 0.0405]
Data/restraints/parameters	2746/0/217
Goodness-of-fit on F2	1.081
Final R indexes [I>=2σ (I)]	R_1 = 0.0503, wR_2 = 0.1414
Final R indexes [all data]	R_1 = 0.0567, wR_2 = 0.1449
Largest diff. peak/hole / e Å-3	0.40/-0.45

Table S21. Crystal data and structure refinement for **10**.

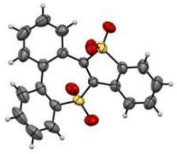

Identification code	**10**
Empirical formula	$C_{20}H_{12}O_4S_2$
Formula weight	380.42
Temperature/K	293
Crystal system	orthorhombic
Space group	Pbca
a/Å	8.02320(10)
b/Å	16.8258(3)
c/Å	25.0537(4)
α/°	90
β/°	90
γ/°	90
Volume/Å3	3382.17(9)
Z	8
ρcalcg/cm3	1.494
μ/mm-1	3.066
F(000)	1568.0
Crystal size/mm3	0.15 × 0.1 × 0.05
Radiation	Cu Kα (λ = 1.54184)
2Θ range for data collection/°	7.056 to 136.646
Index ranges	-6 ≤ h ≤ 9, -20 ≤ k ≤ 17, -30 ≤ l ≤ 29
Reflections collected	12149
Independent reflections	3077 [R_{int} = 0.0439, R_{sigma} = 0.0401]
Data/restraints/parameters	3077/0/235
Goodness-of-fit on F2	1.101
Final R indexes [I>=2σ (I)]	R_1 = 0.0655, wR_2 = 0.1826
Final R indexes [all data]	R_1 = 0.0703, wR_2 = 0.1864
Largest diff. peak/hole / e Å-3	0.96/-0.41

XI. Theoretical calculations

Theoretical calculations were carried out using Gaussian 09 software.[8] The ground-state structures were optimized by density functional theory (DFT) at B3LYP/6-31G* level.[9,10] The electrostatic potentials and FMO distributions were visualized using Gaussview 5.0 software.

(i) Calculated FMO distributions and orbital energy levels

Table S22. FMO distributions of sulfur-embedded polycyclic aromatics.

Compounds	HOMO	LUMO
**7a**	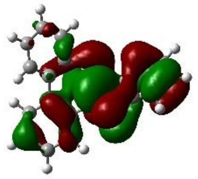	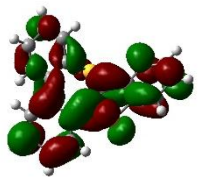
**7b**	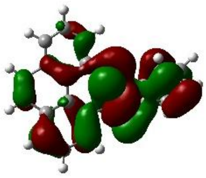	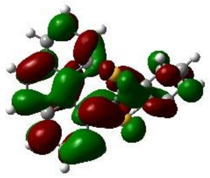
**8a**	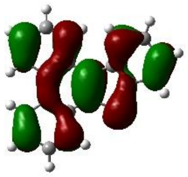	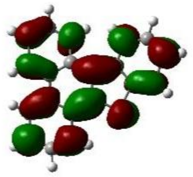
**8b**	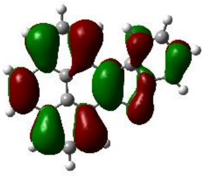	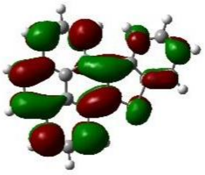

**9**	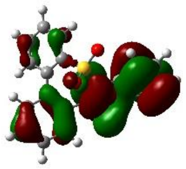	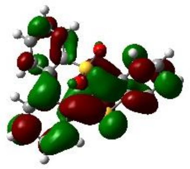
**10**	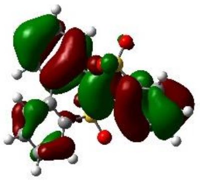	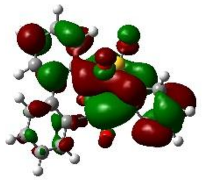

Table S23. Calculated orbital energy levels of sulfur-embedded polycyclic aromatics

Compounds	HOMO (eV)	LUMO (eV)	E_g (eV)
**7a**	-5.63	-1.33	4.30
**7b**	-5.55	-1.56	3.99
**8a**	-5.54	-1.35	4.19
**8b**	-5.33	-1.66	3.67
**9**	-6.15	-1.71	4.44
**10**	-6.64	-2.62	4.02

(ii) The electrostatic potential maps

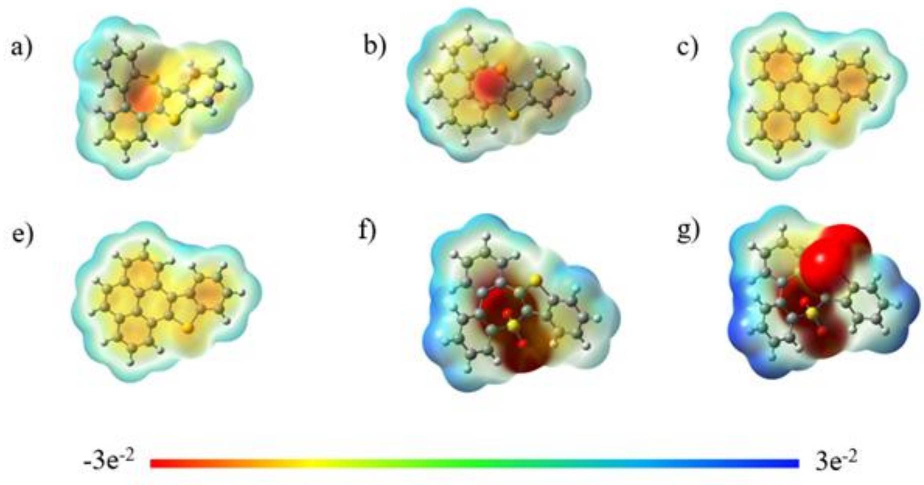

Figure S9. Electrostatic potential maps of (a) **7a**, (b) **7b**, (c) **8a**, (d) **8b**, (e) **9**, (f) **10**.

(iii) Hirshfeld surfaces analysis

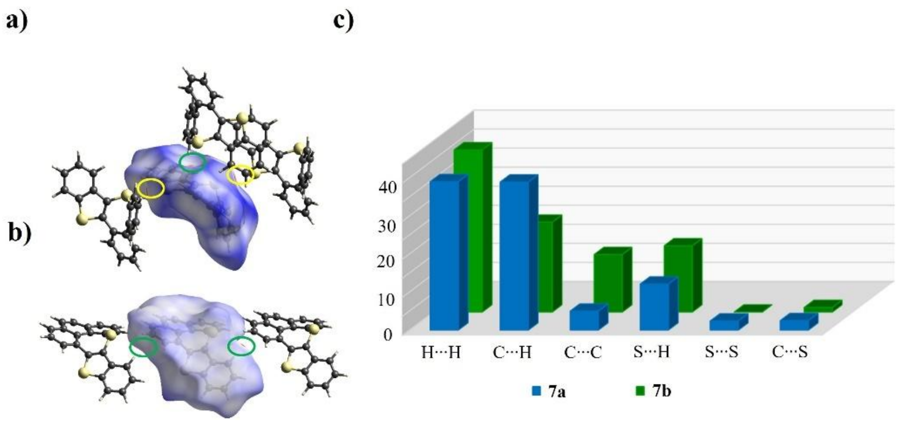

Figure S10. Hirshfeld d_{norm} surfaces of the crystals consisting of (a) **7a**, (b) **7b**; (c) Relative contributions to the Hirshfeld d_{norm} surfaces for the various intermolecular contacts of **7a** and **7b**. The green and yellow circles correspond to the C–H···π interactions and S···S interactions, respectively.

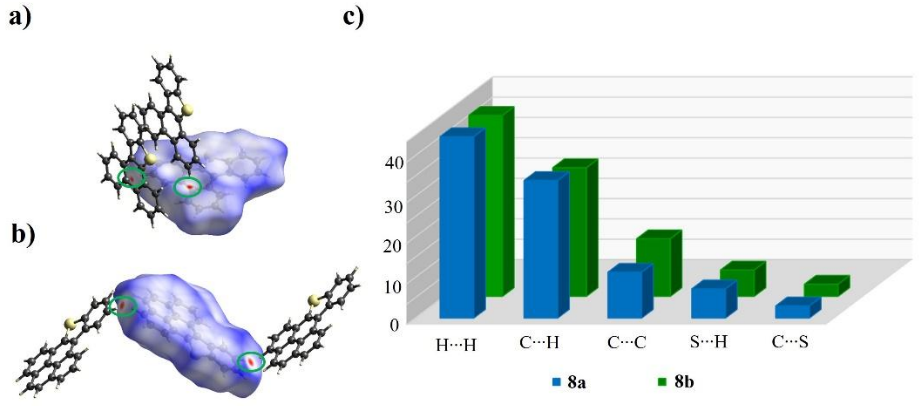

Figure S11. Hirshfeld d_{norm} surfaces of the crystals consisting of (a) **8a**, (b) **8b**; (c) Relative contributions to the Hirshfeld d_{norm} surfaces for the various intermolecular contacts of **8a** and **8b**. The green circles correspond to the C–H···π interactions.

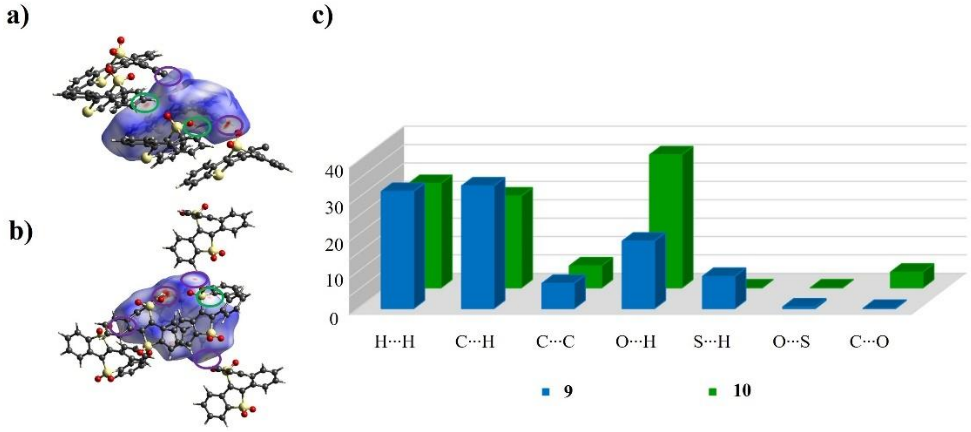

Figure S12. Hirshfeld d_{norm} surfaces of the crystals consisting of (a) **9**, (b) **10**; (c) Relative contributions to the Hirshfeld d_{norm} surfaces for the various intermolecular contacts of **9** and **10**. The green and red circles correspond to the C–H···π and hydrogen bond interactions, respectively.

XII. References

[1] Y. Li, B.-J. Li, W.-H. Wang, W.-P. Huang, X.-S. Zhang, K. Chen, Z.-J. Shi, *Angew. Chem. Int. Ed.*, **2011**, 50, 2115−2119.

[2] G. Liao, T. Zhang, L. Jin, B.-J. Wang, C.-K. Xu, Y. Lan, Y. Zhao, B. Shi, *Angew. Chem. Int. Ed.*, **2022**, *61*, e202115221.

[3] P. L. McGinley, J. T. Koh, *J. Am. Chem. Soc.*, **2007**, *129*, 3822−3823.

[4] N. X. Gu, P. H. Oyala, J. C. Peters, *J. Am. Chem. Soc.*, **2018**, *140*, 6374−6382.

[5] C. Duan, J. Zhang, J. Xiang, X. Yang, X. Gao, *Angew. Chem. Int. Ed.*, **2022**, *61*, e202201494.

[6] X.-S. Zhang, Y.-F. Zhang, K. Chen, Z.-J. Shi, *Org. Chem. Front.*, **2014**, *1*, 1096−1100.

[7] Y. Ran, Y. Yang, H. You, J. You, *ACS Catal.*, **2018**, *8*, 1796−1801.

[8] M. J. Frisch, G. W. Trucks, H. B. Schlegel, G. E. Scuseria, M. A. Robb, J. R. Cheeseman, G. Scalmani, V. Barone, B. Mennucci, G. A. Petersson, H. Nakatsuji, M. Caricato, X. Li, H. P. Hratchian, A. F. Izmaylov, J. Bloino, G. Zheng, J. L. Sonnenberg, M. Hada, M. Ehara, K. Toyota, R. Fukuda, J. Hasegawa, M. Ishida, T. Nakajima, Y. Honda, O. Kitao, H. Nakai, T. Vreven, J. A. Jr. Montgomery, J. E. Peralta, F. Ogliaro, M. Bearpark, J. J. Heyd, E. Brothers, K. N. Kudin, V. N. Staroverov, R. Kobayashi, J. Normand, K. Raghavachari, A. Rendell, J. C. Burant, S. S. Iyengar, J. Tomasi, M. Cossi, N. Rega, N. J. Millam, M. Klene, J. E. Knox, J. B. Cross, V. Bakken, C. Adamo, J. Jaramillo, R. Gomperts, R. E. Stratmann, O. Yazyev, A. J. Austin, R. Cammi, C. Pomelli, J. W. Ochterski, R. L. Martin, K. Morokuma, V. G. Zakrzewski, G. A. Voth, P. Salvador, J. J. Dannenberg, S. Dapprich, A. D. Daniels, Ö. Farkas, J. B. Foresman, J. V. Ortiz, J. Cioslowski, D. J. Fox, Gaussian 09, Revision D.01, Gaussian, Inc., Wallingford CT, 2009.

[9] A. D. Becke, *J. Chem. Phys.*, **1993**, *98*, 5648−5652.

[10] C. Lee, W. Yang, R. G. Parr, *Phys. Rev. B.*, **1988**, *37*, 785−789.

XIII. Copies of ^{1}H and ^{13}C{^{1}H} NMR spectra

^{1}H NMR spectrum of **1r** in $CDCl_3$ (300 MHz)

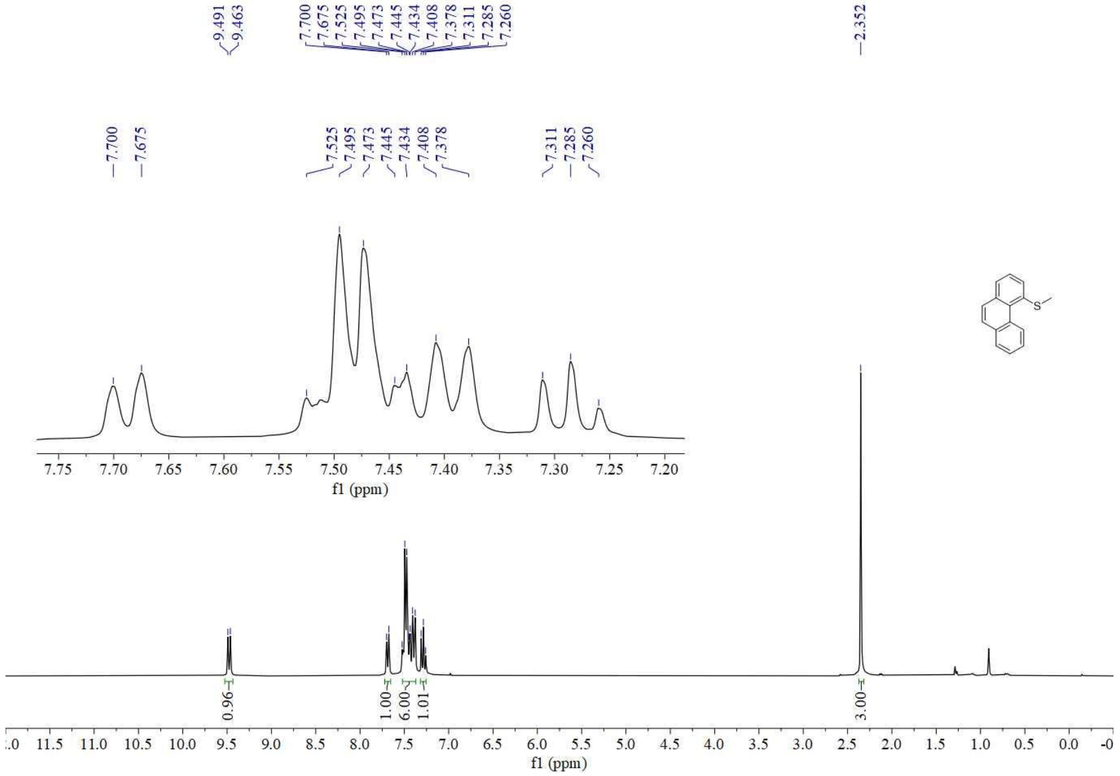

^{13}C{^{1}H} NMR spectrum of **1r** in $CDCl_3$ (75 MHz)

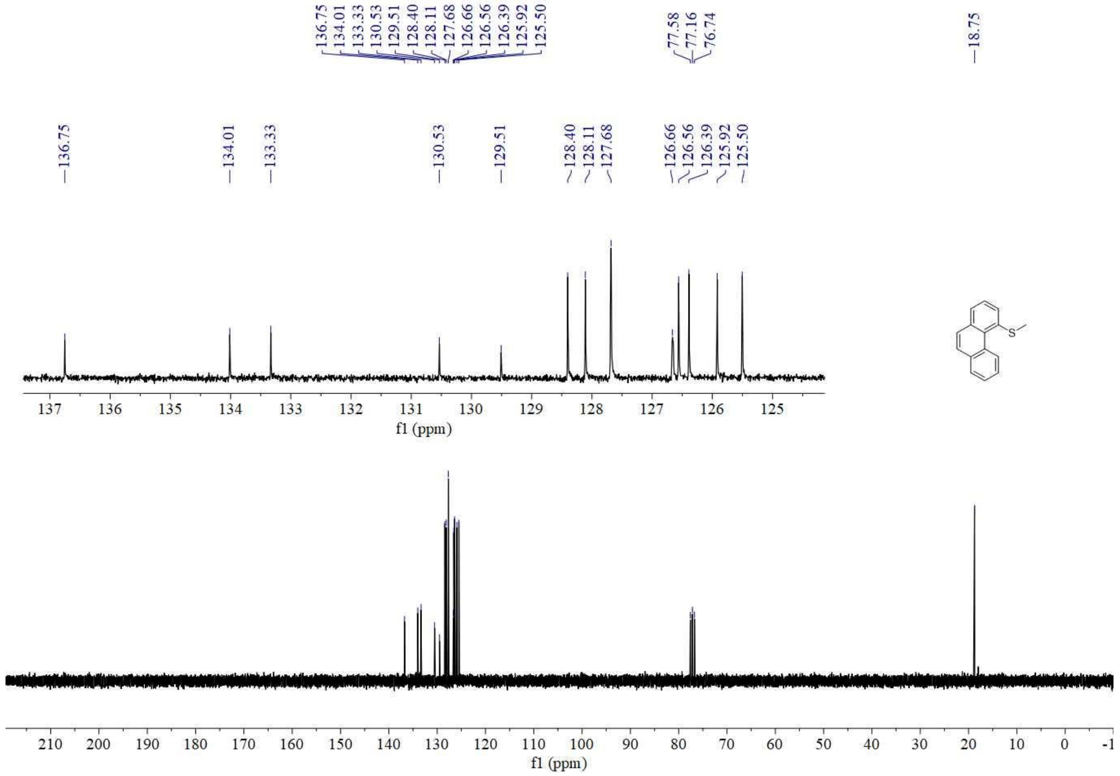

^{1}H NMR spectrum of **5c** in $CDCl_3$ (300 MHz)

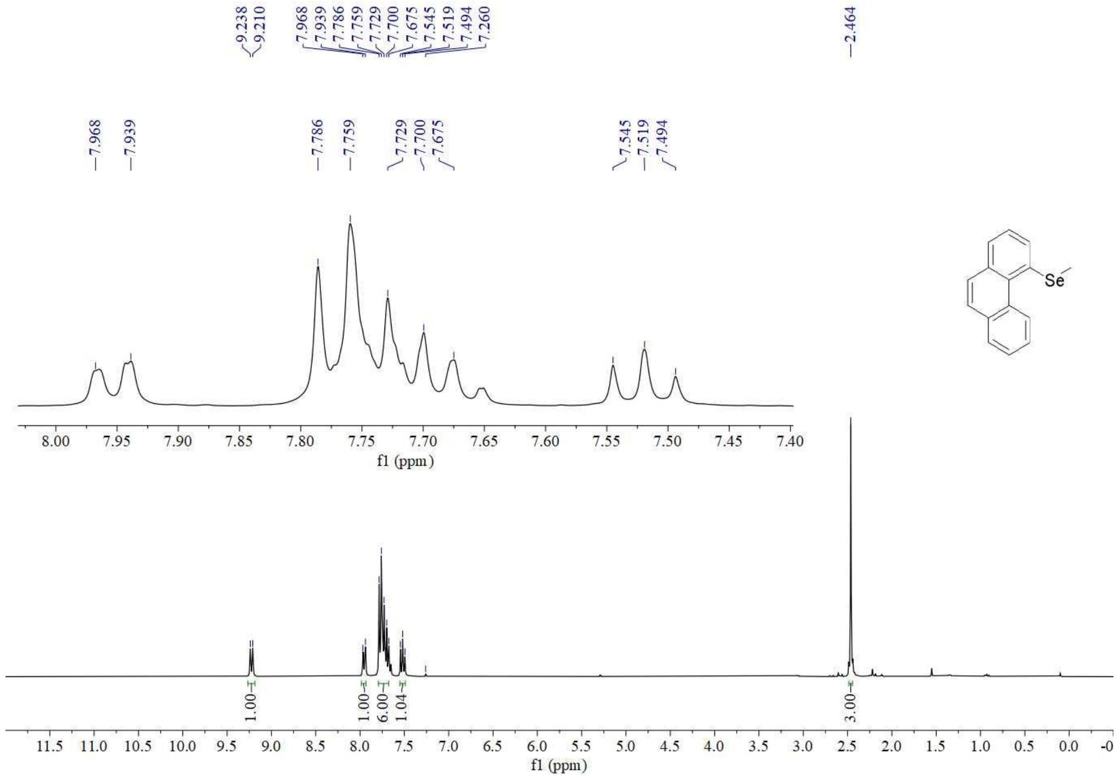

$^{13}C\{^{1}H\}$ NMR spectrum of **5c** in $CDCl_3$ (75 MHz)

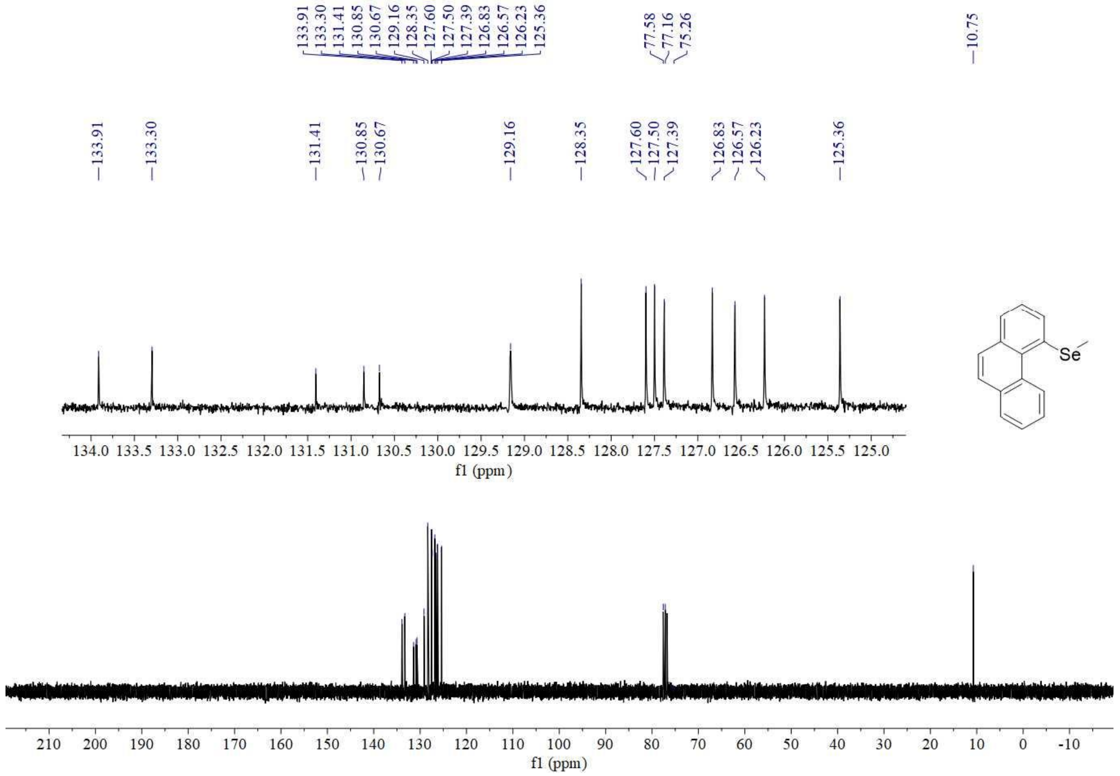

^{1}H NMR spectrum of [D$_5$]-**1m** in $CDCl_3$ (300 MHz)

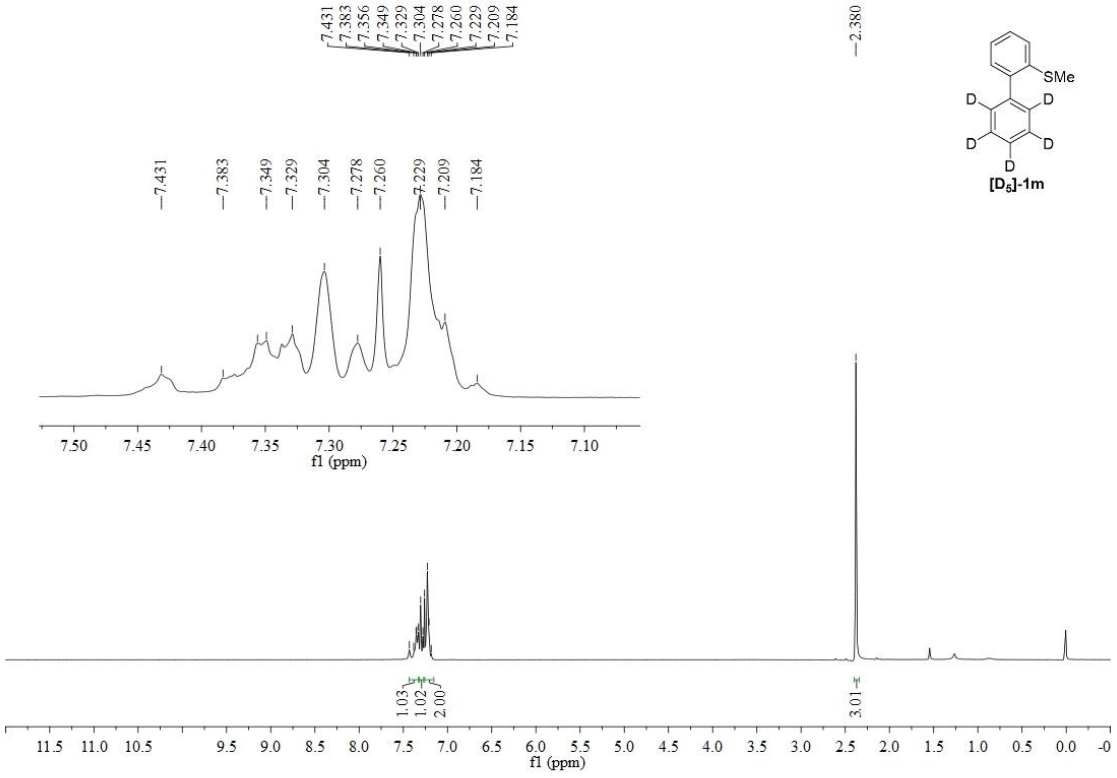

^{1}H NMR spectrum of [D]-**2a** in $CDCl_3$ (300 MHz)

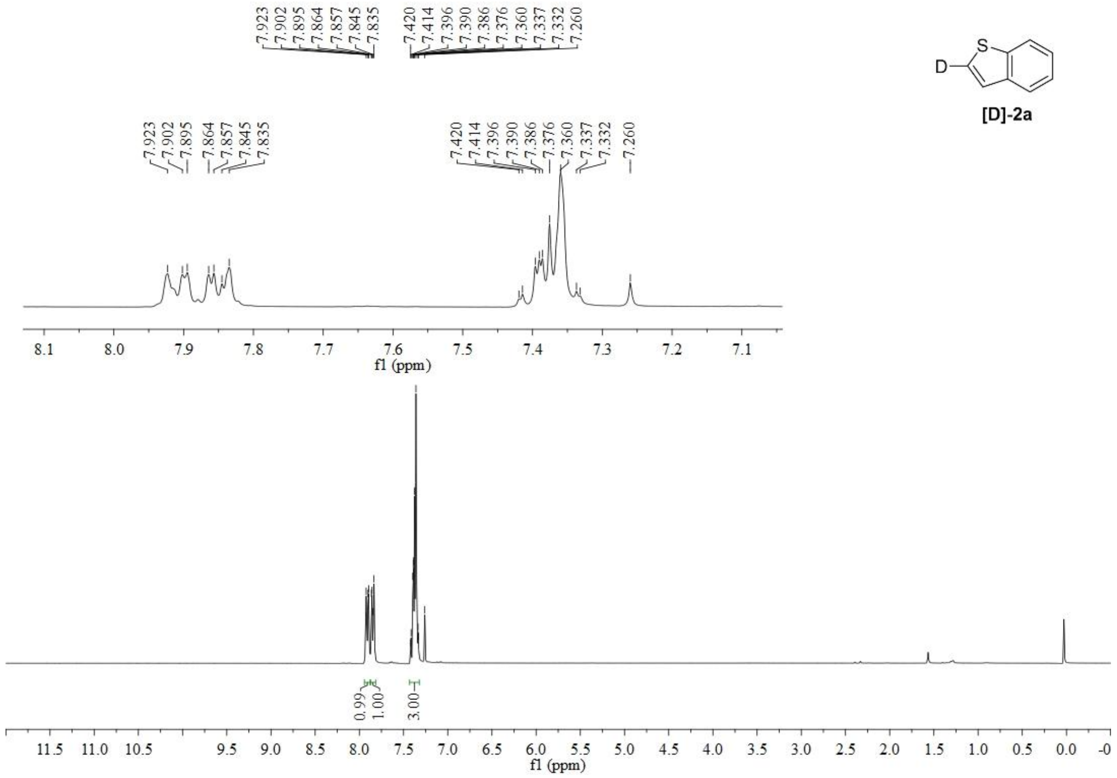

^{1}H NMR spectrum of **3a** in $CDCl_3$ (300 MHz)

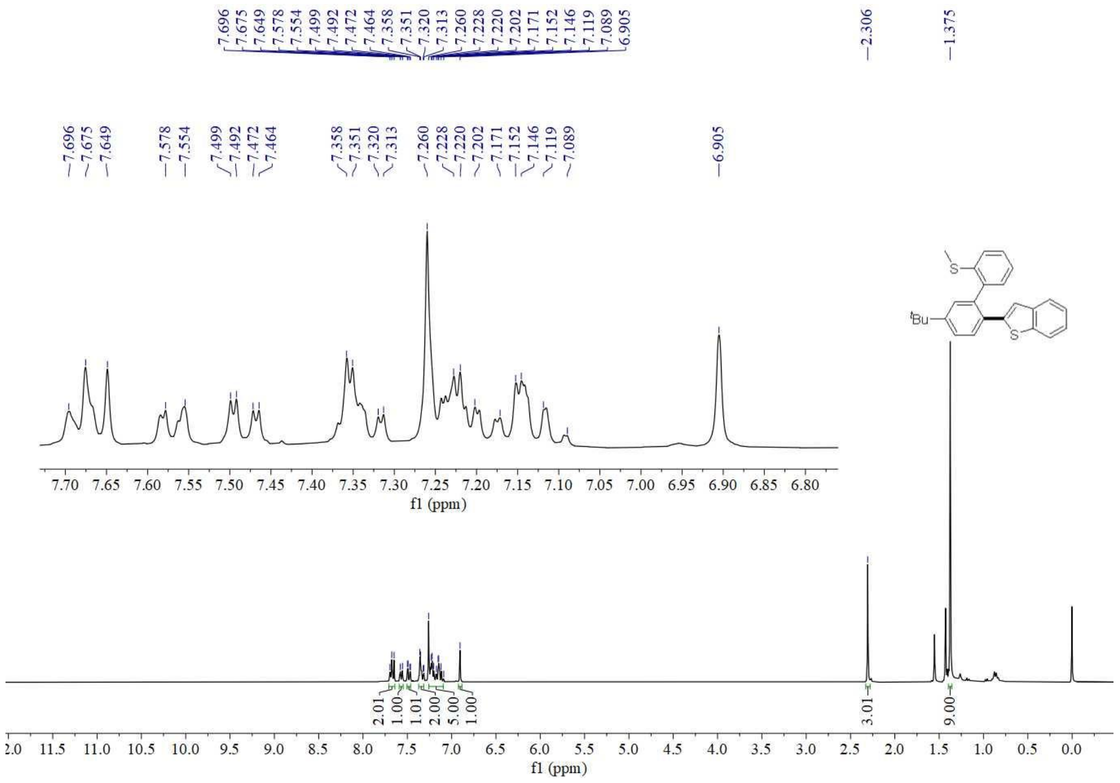

$^{13}C\{^1H\}$ NMR spectrum of **3a** in $CDCl_3$ (75 MHz)

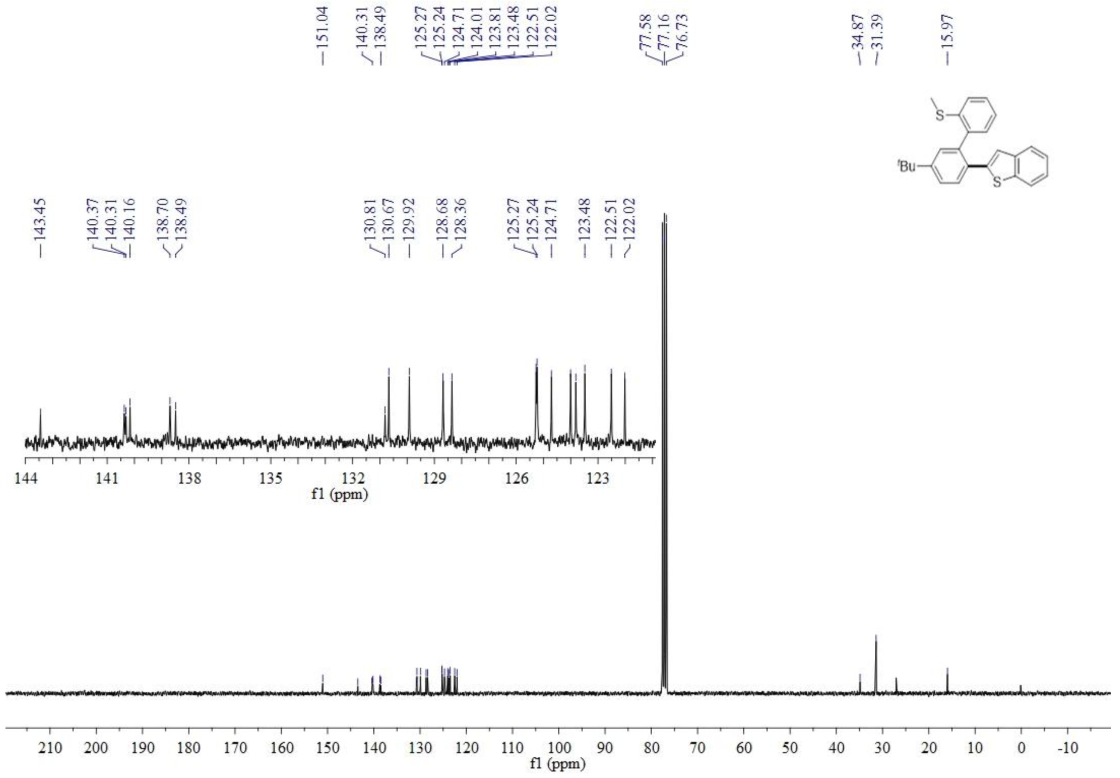

^{1}H NMR spectrum of **3b** in $CDCl_3$ (300 MHz)

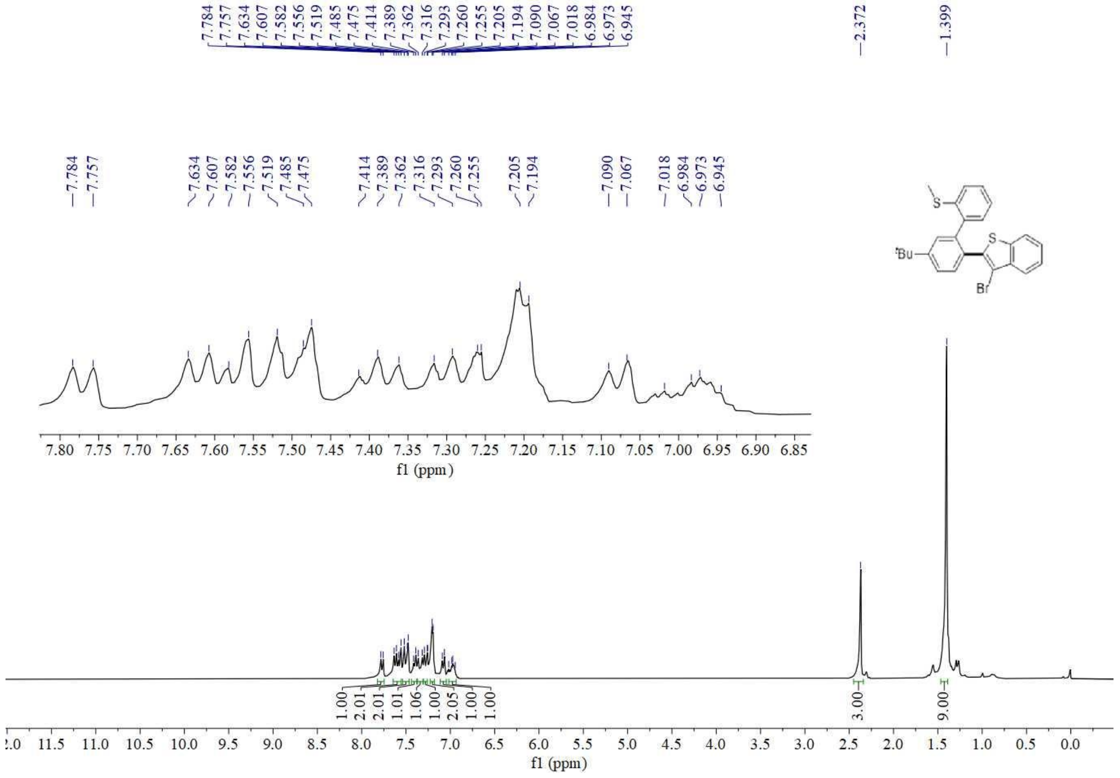

$^{13}C\{^{1}H\}$ NMR spectrum of **3b** in $CDCl_3$ (75 MHz)

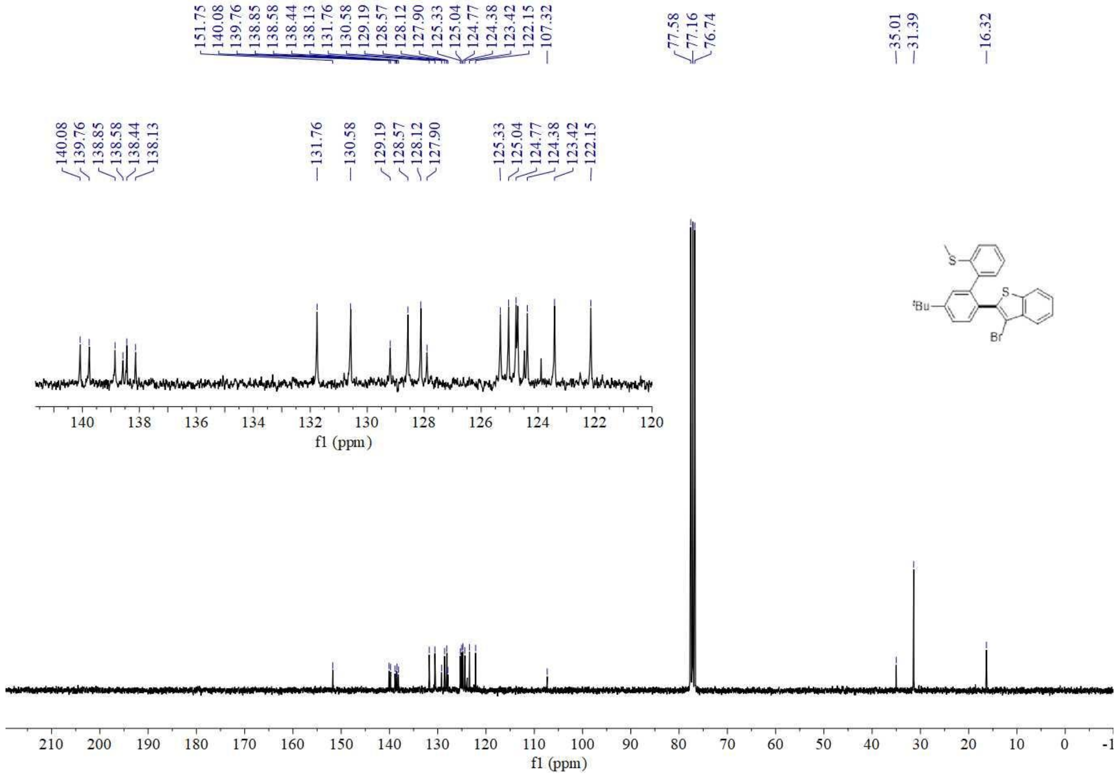

^{1}H NMR spectrum of **3c** in $CDCl_3$ (300 MHz)

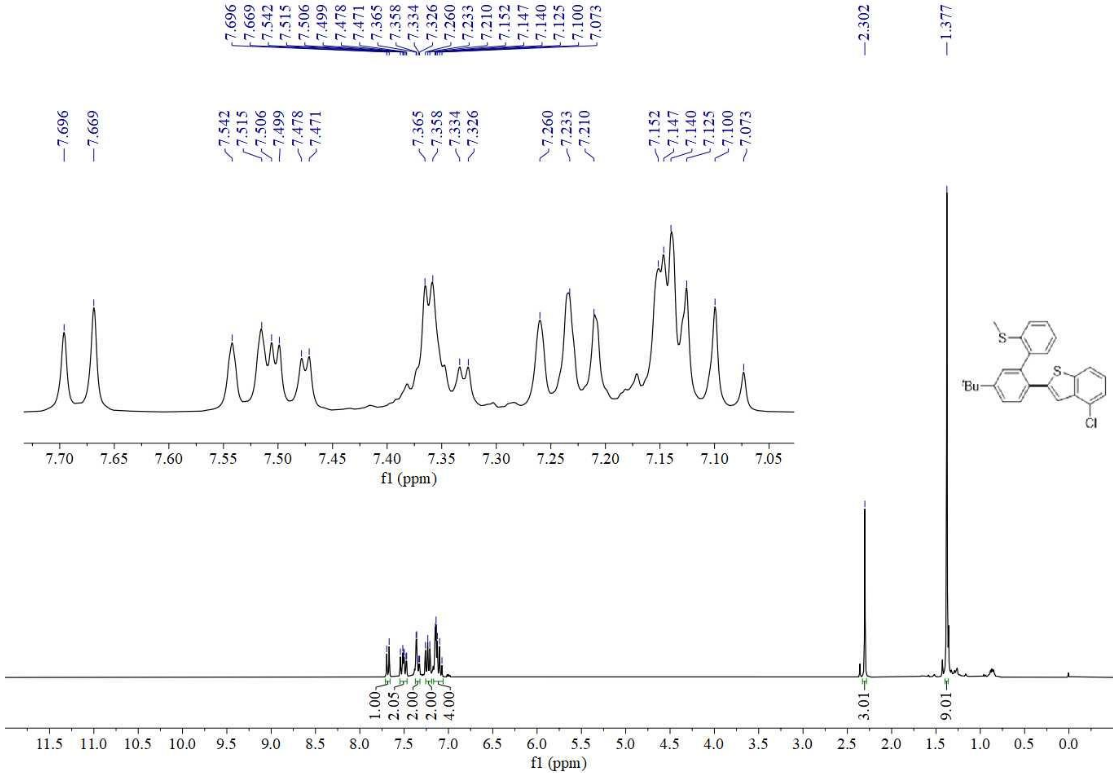

^{13}C{^{1}H} NMR spectrum of **3c** in $CDCl_3$ (75 MHz)

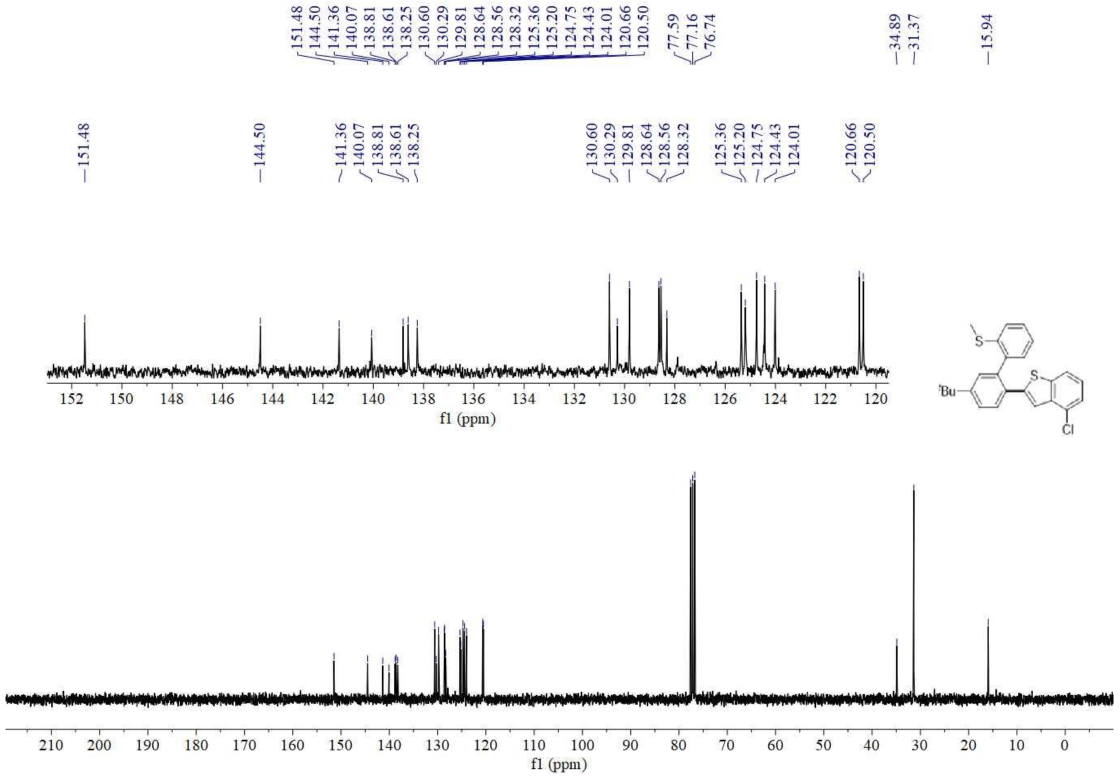

^{1}H NMR spectrum of **3d** in $CDCl_3$ (300 MHz)

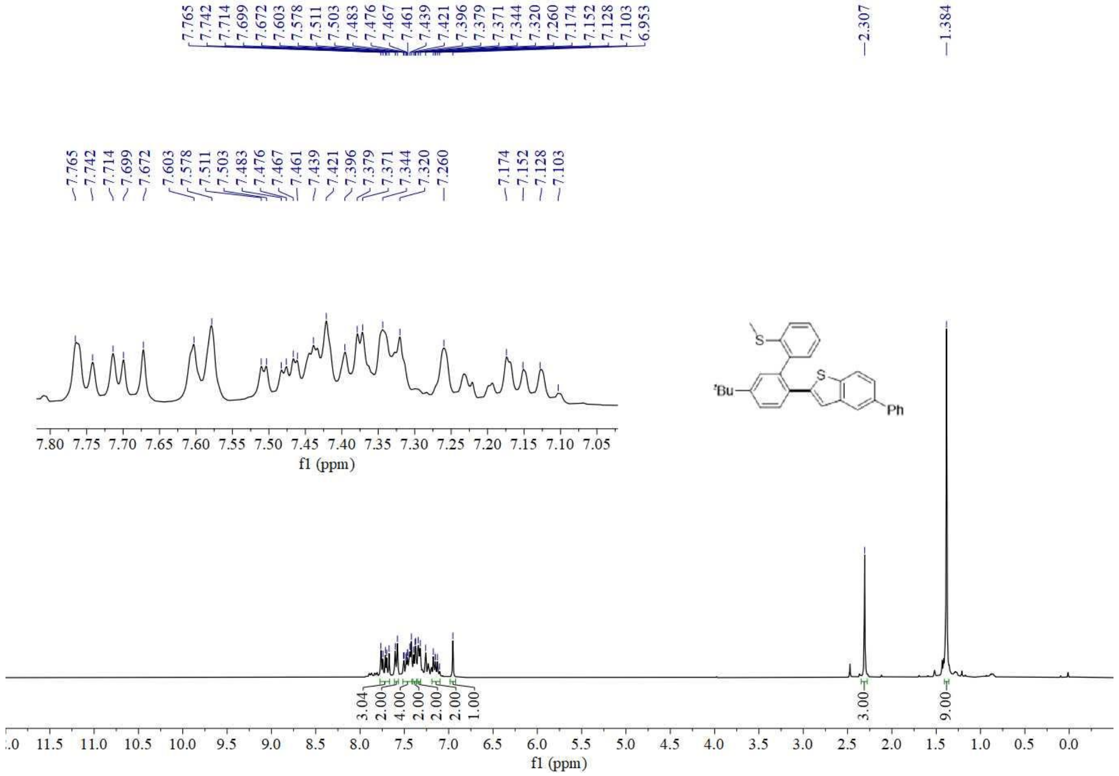

$^{13}C\{^{1}H\}$ NMR spectrum of **3d** in $CDCl_3$ (75 MHz)

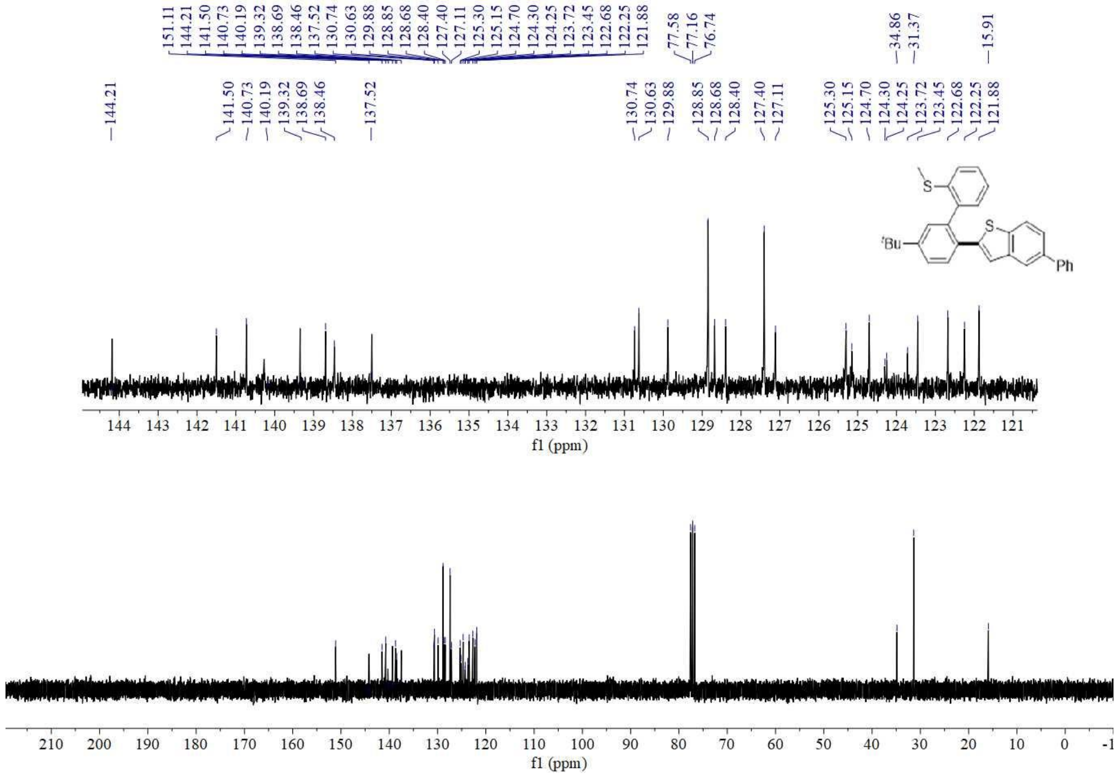

^{1}H NMR spectrum of **3e** in $CDCl_3$ (300 MHz)

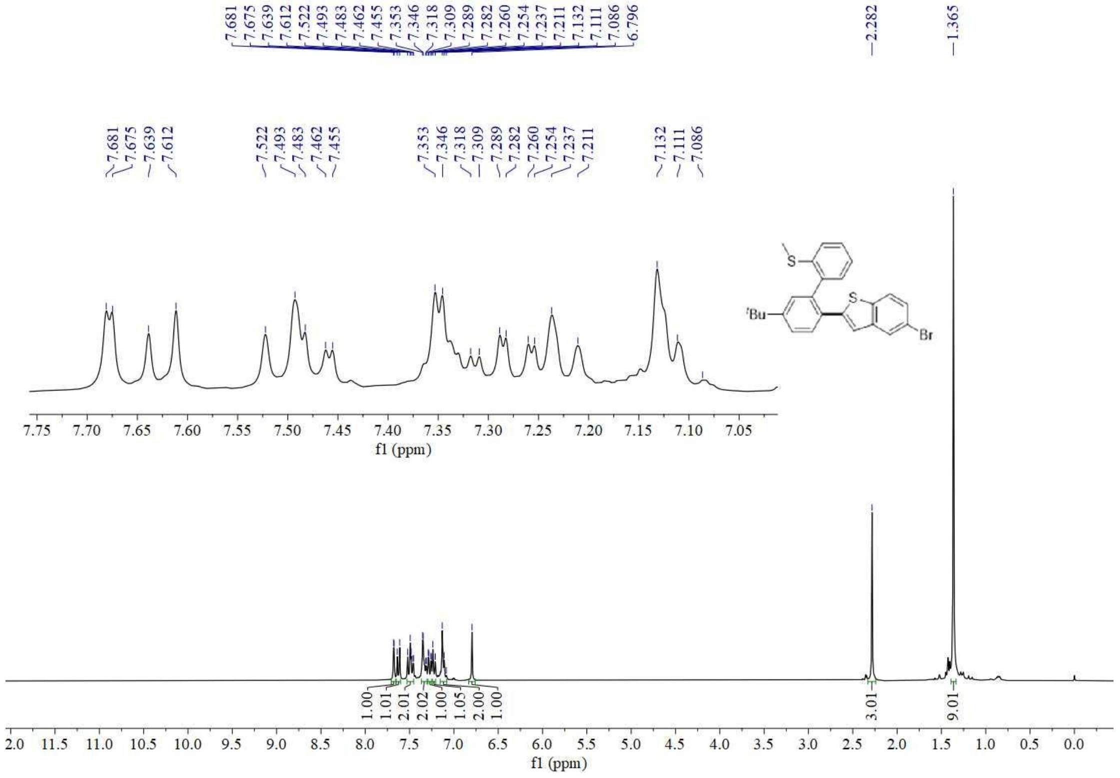

$^{13}C\{^1H\}$ NMR spectrum of **3e** in $CDCl_3$ (75 MHz)

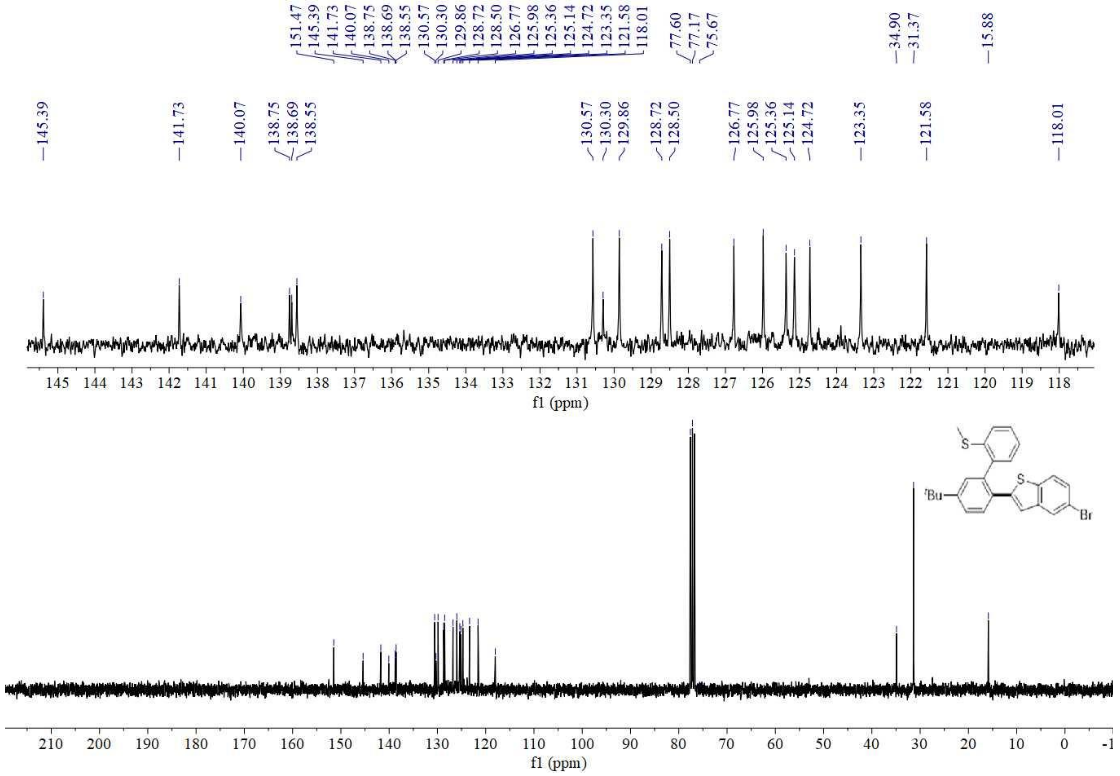

^{1}H NMR spectrum of **3f** in $CDCl_3$ (300 MHz)

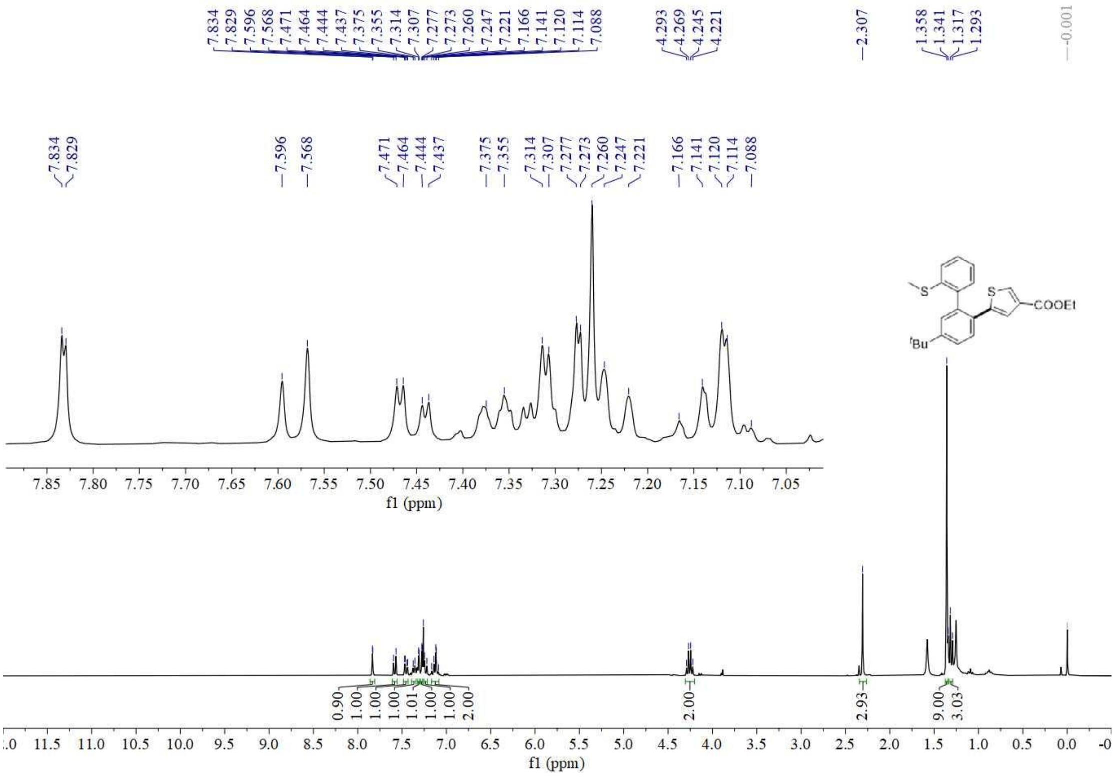

^{13}C{^{1}H} NMR spectrum of **3f** in $CDCl_3$ (75 MHz)

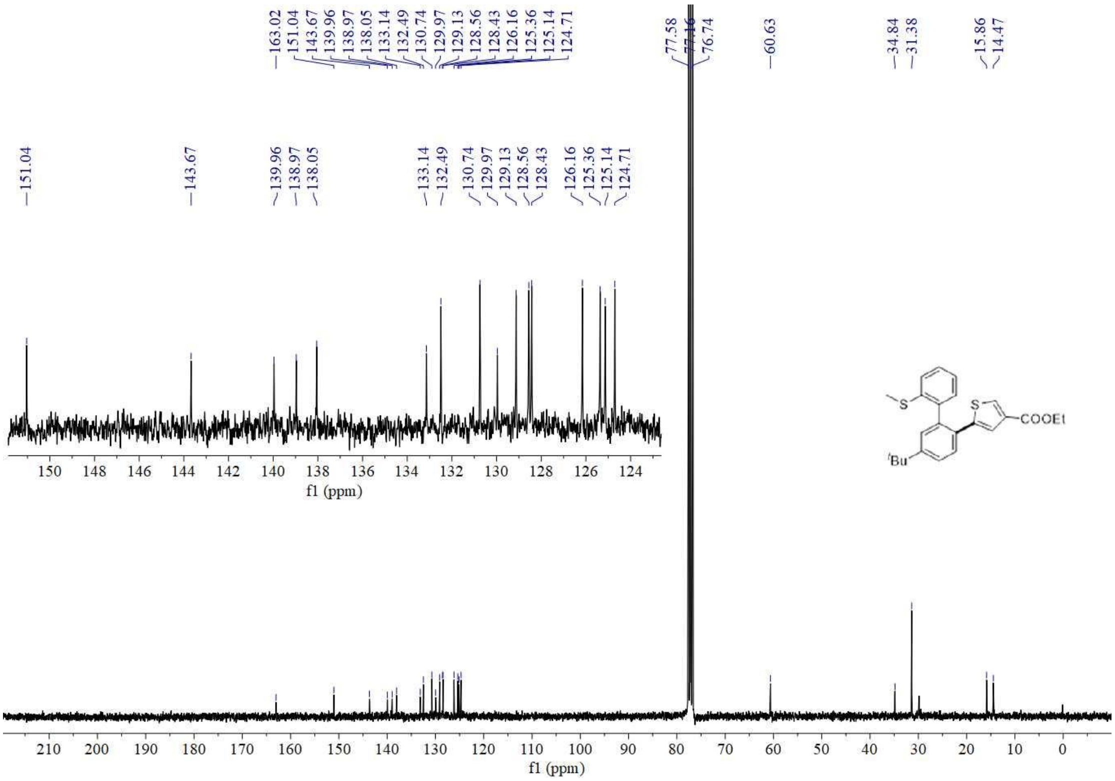

^{1}H NMR spectrum of **3g** in $CDCl_3$ (300 MHz)

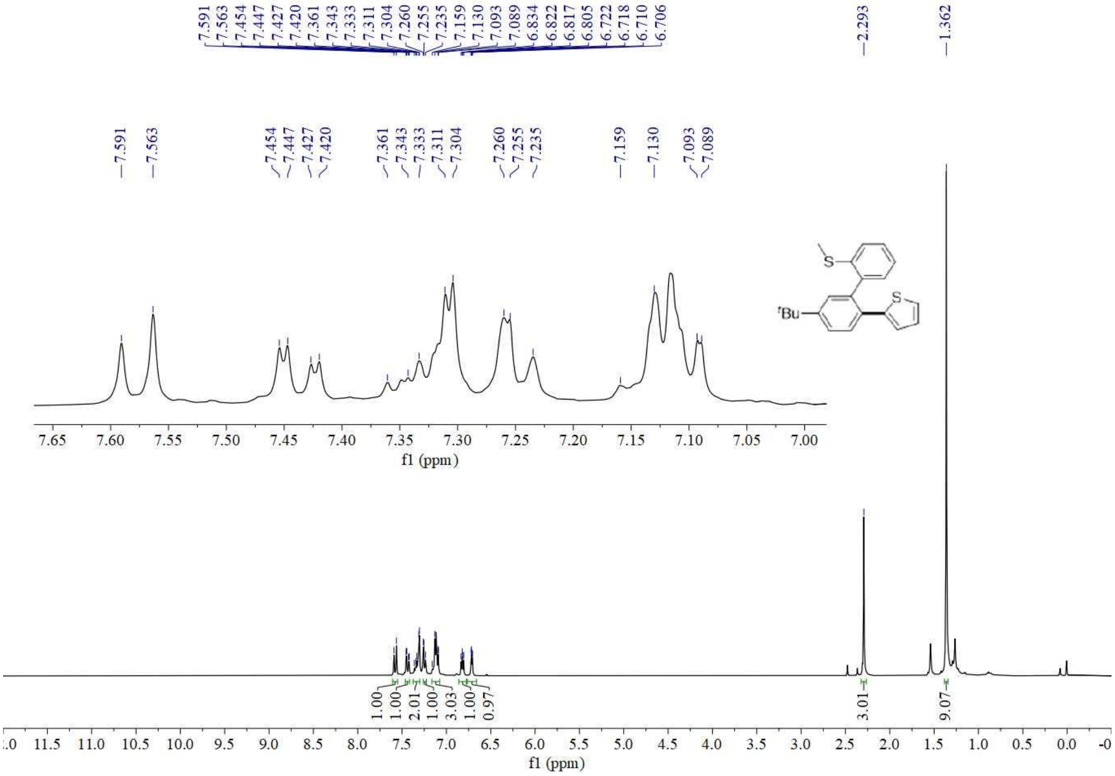

^{13}C{^{1}H} NMR spectrum of **3g** in $CDCl_3$ (75 MHz)

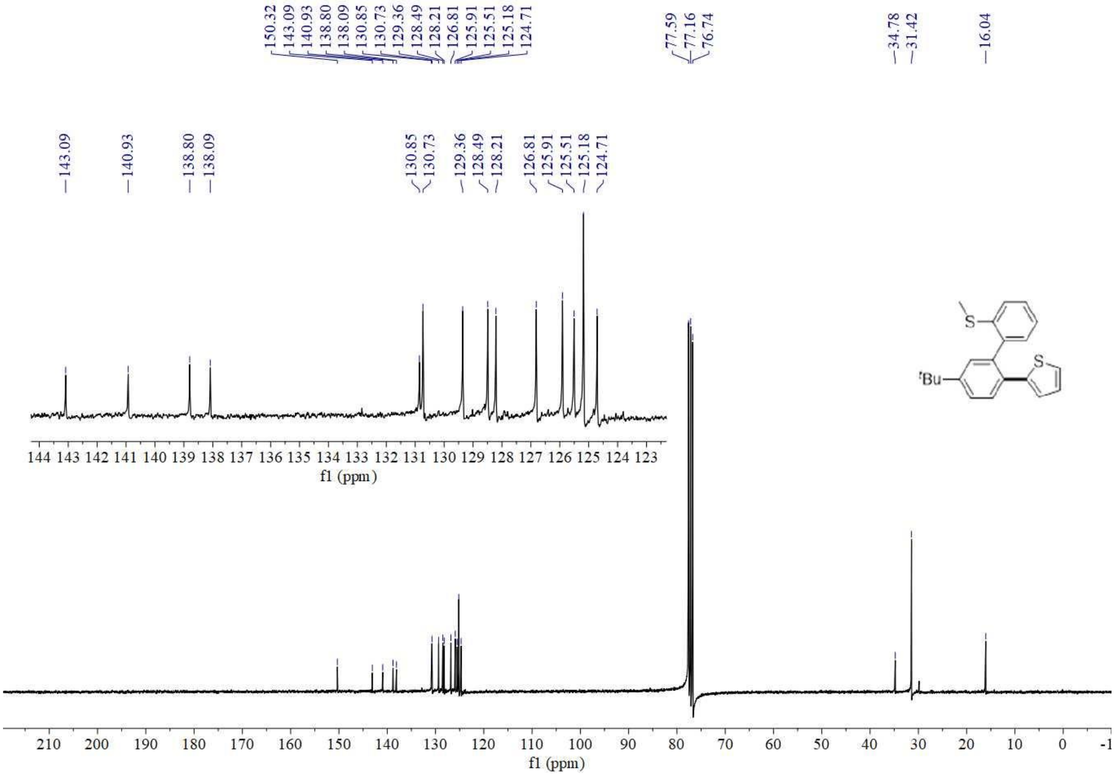

^{1}H NMR spectrum of **3h** in $CDCl_3$ (300 MHz)

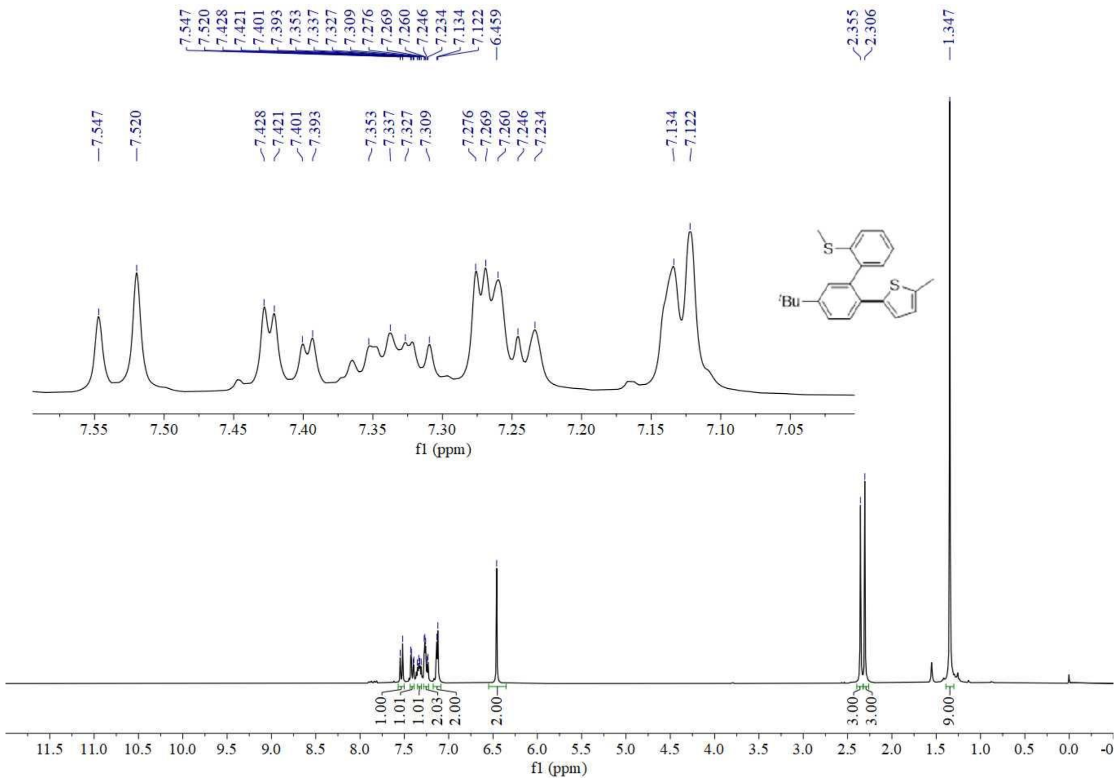

^{13}C{^{1}H} NMR spectrum of **3h** in $CDCl_3$ (75 MHz)

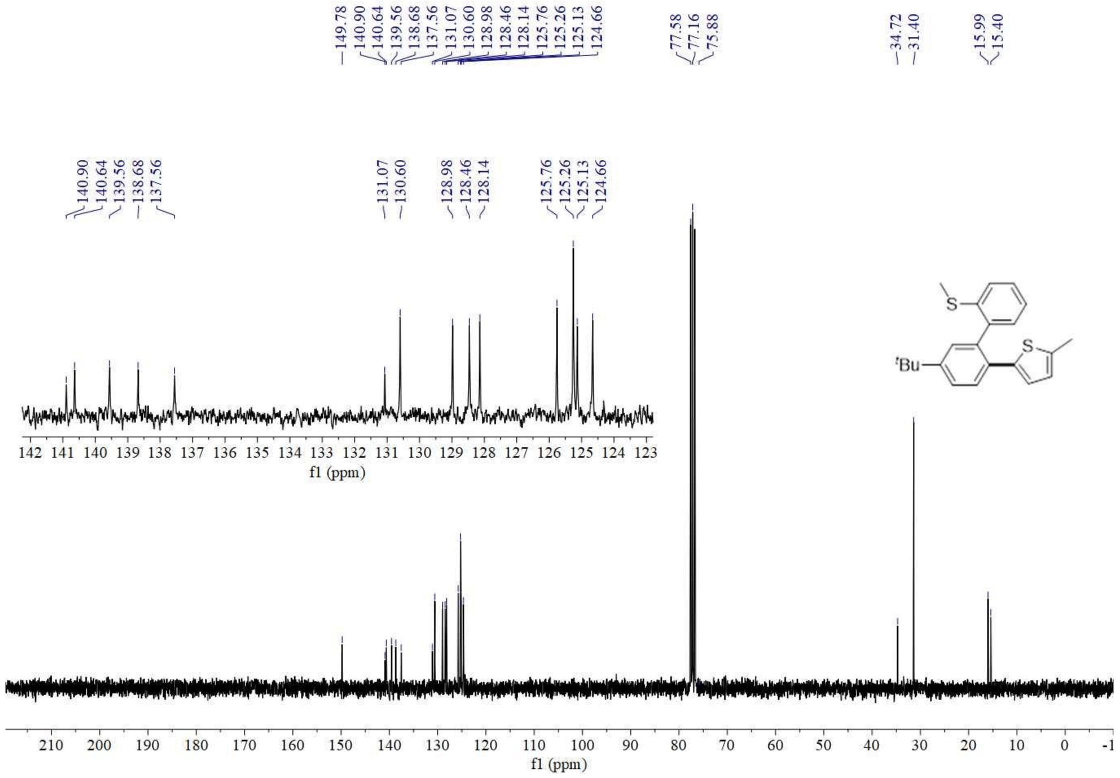

^{1}H NMR spectrum of **3i** in $CDCl_3$ (300 MHz)

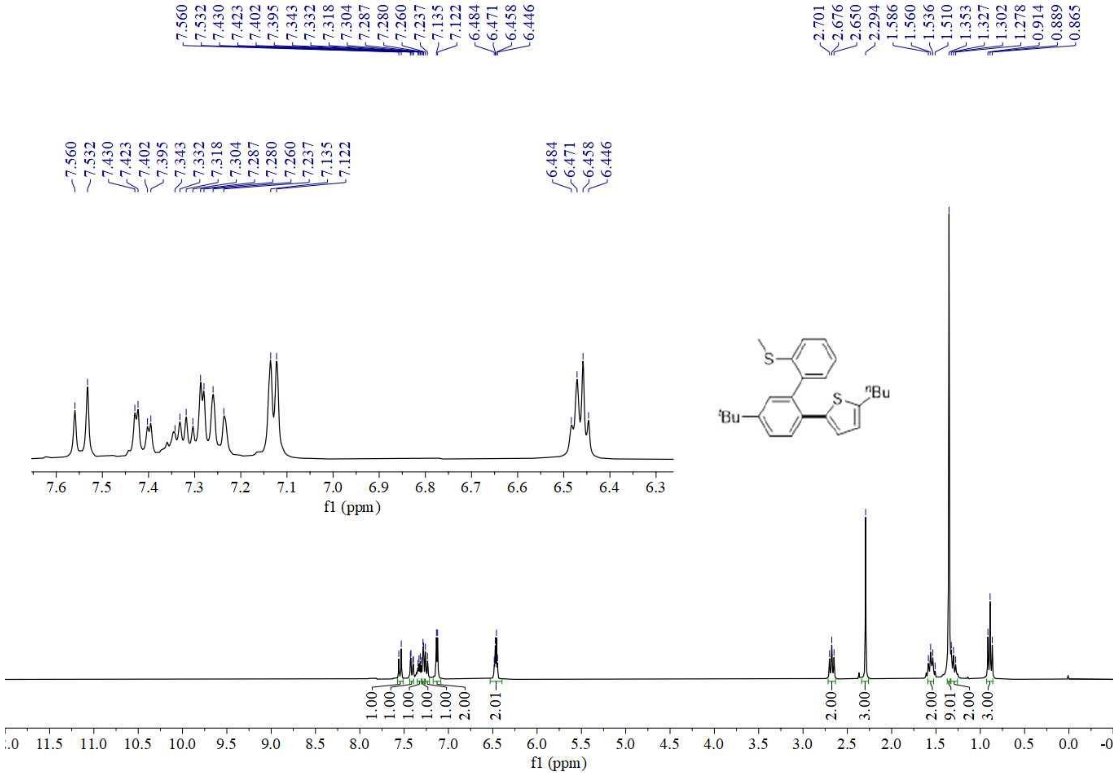

^{13}C{^{1}H} NMR spectrum of **3i** in $CDCl_3$ (75 MHz)

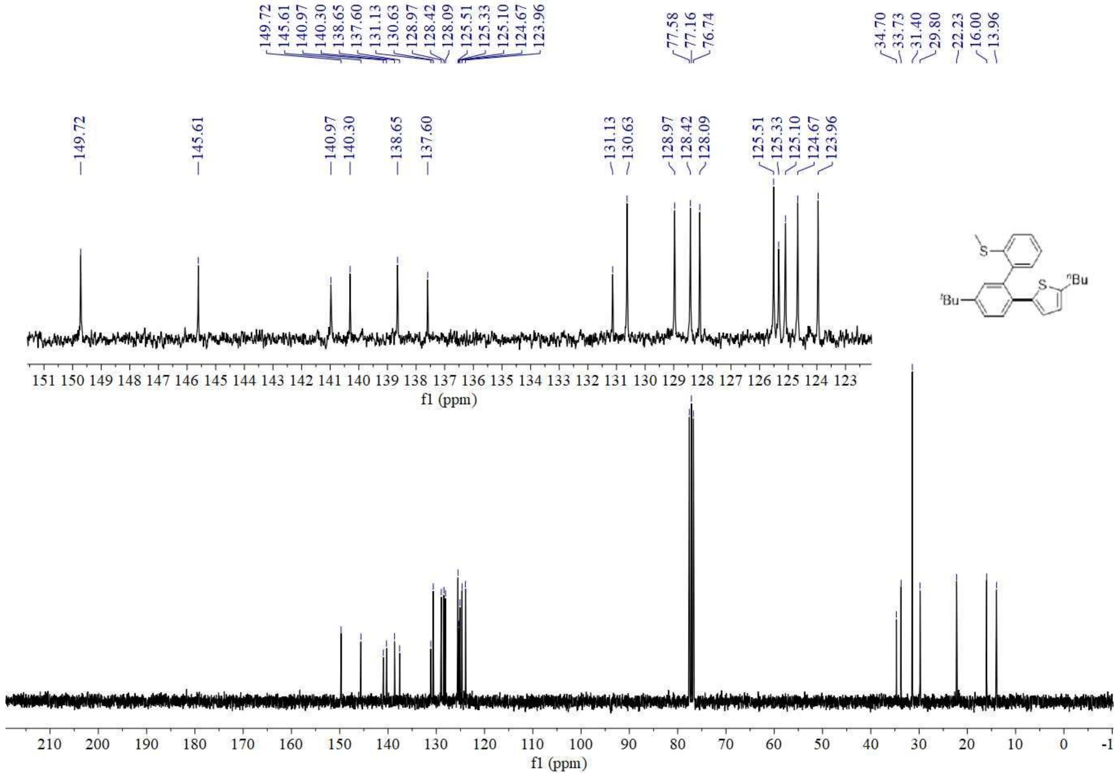

^{1}H NMR spectrum of **3j** in $CDCl_3$ (300 MHz)

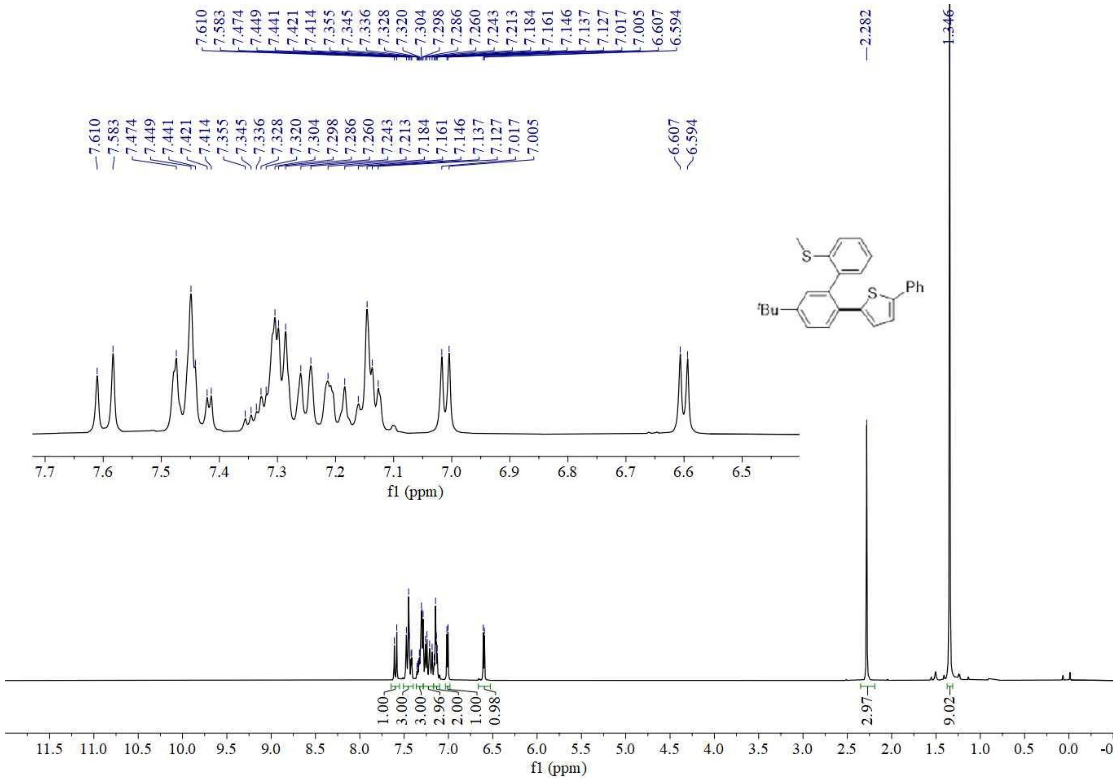

^{13}C{^{1}H} NMR spectrum of **3j** in $CDCl_3$ (75 MHz)

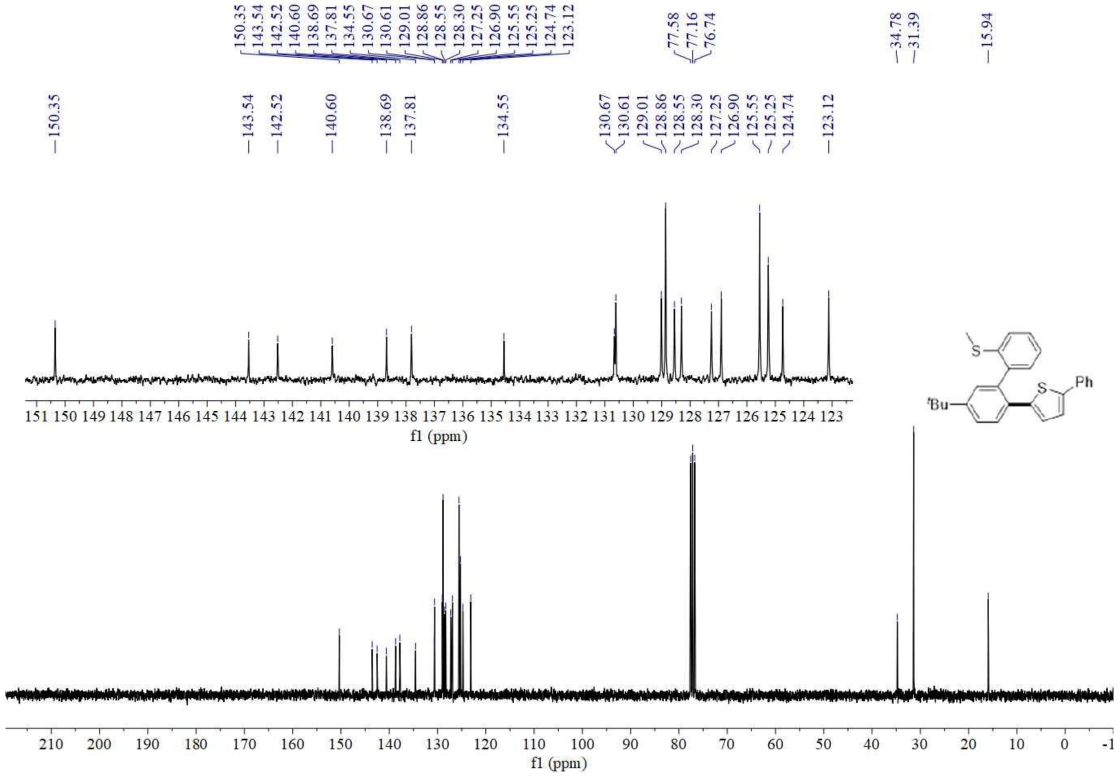

^{1}H NMR spectrum of **3k** in $CDCl_3$ (300 MHz)

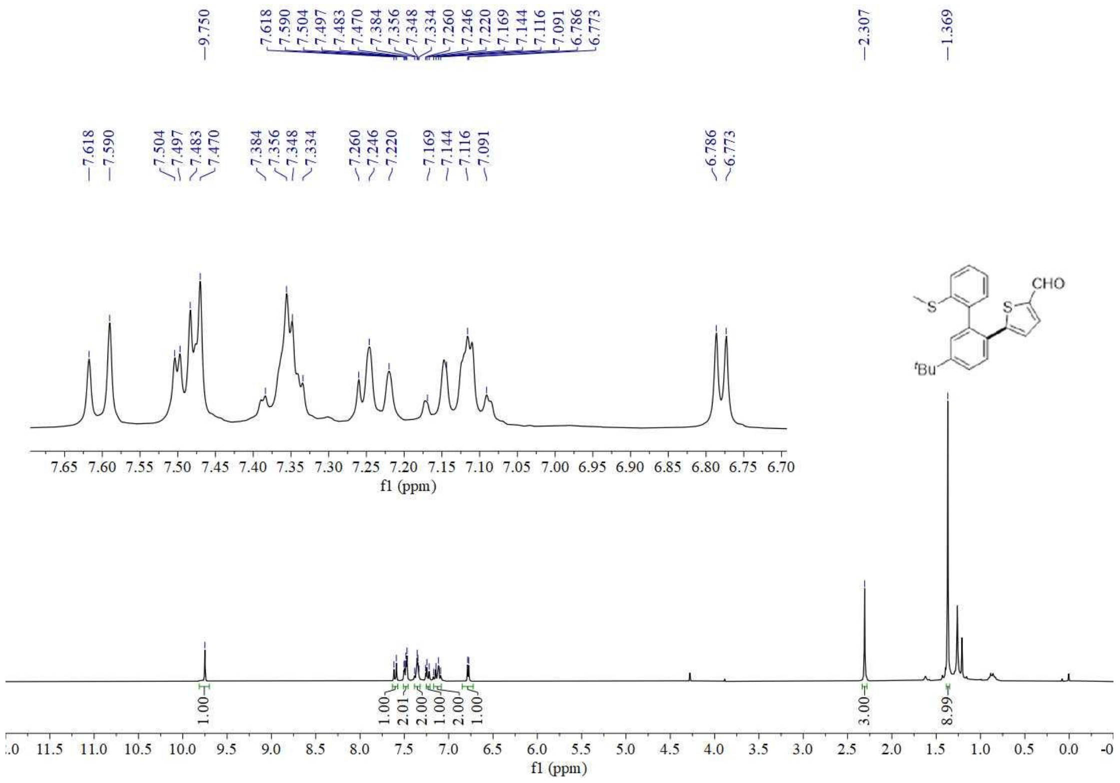

^{13}C{^{1}H} NMR spectrum of **3k** in $CDCl_3$ (75 MHz)

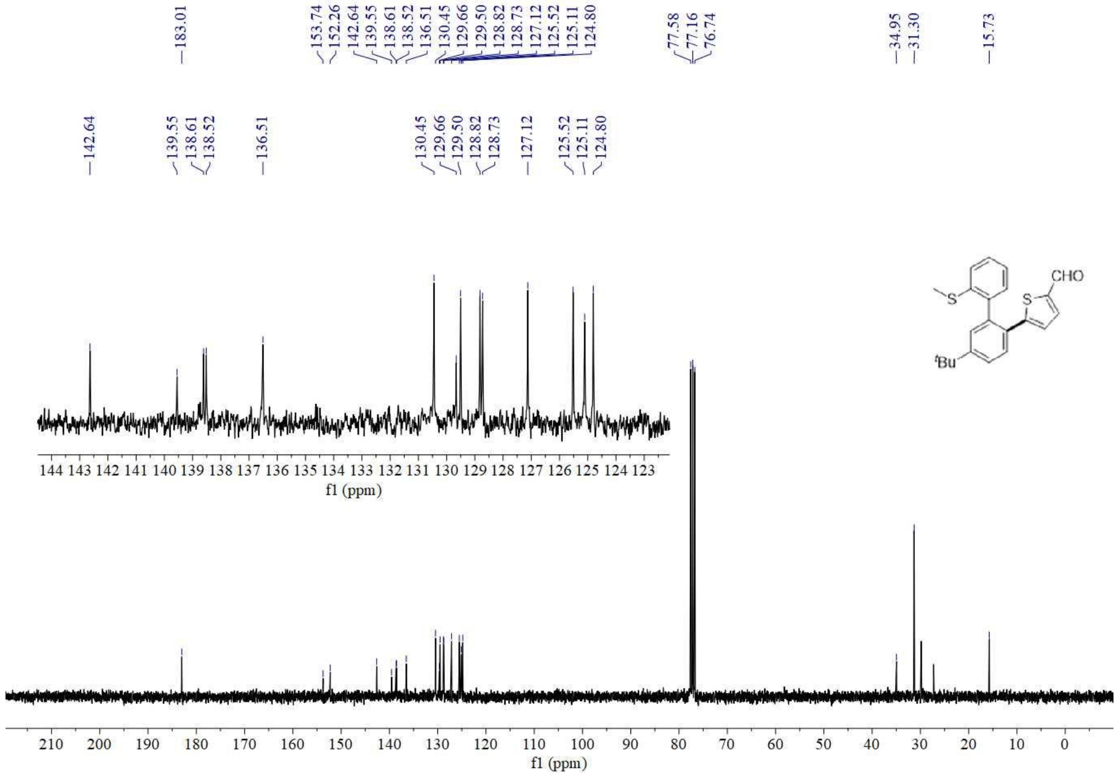

^{1}H NMR spectrum of **3l** in $CDCl_3$ (300 MHz)

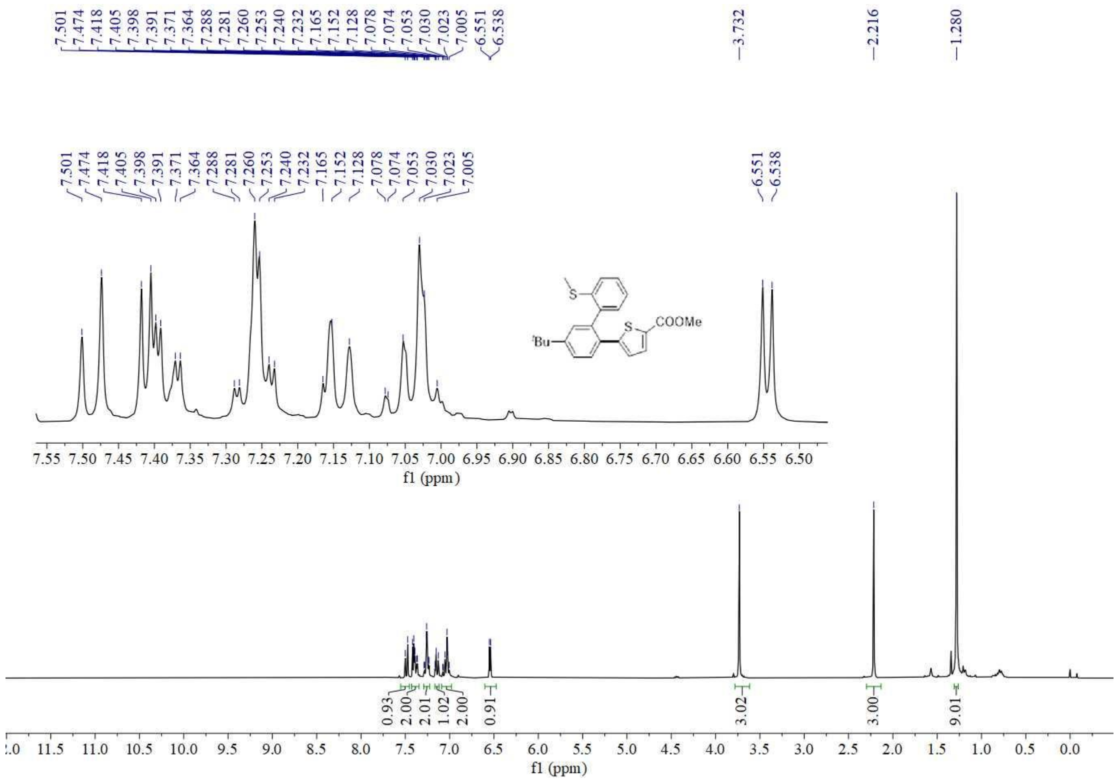

^{13}C{^{1}H} NMR spectrum of **3l** in $CDCl_3$ (75 MHz)

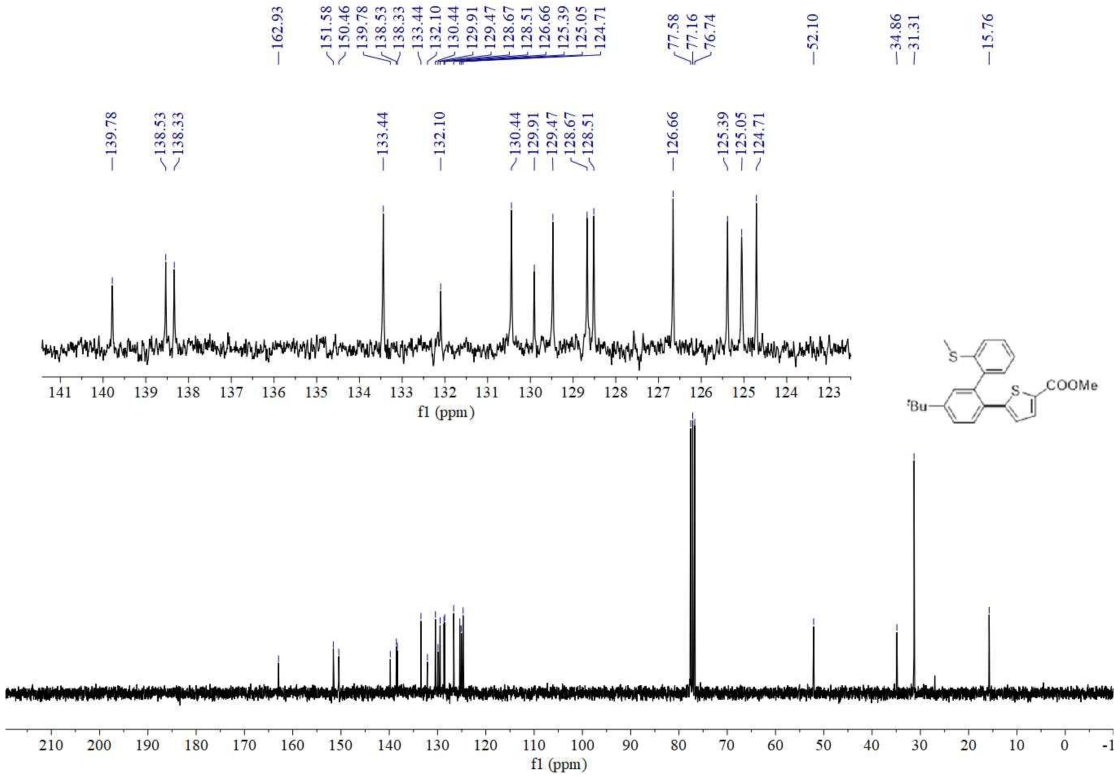

^1H NMR spectrum of **3m** in $CDCl_3$ (300 MHz)

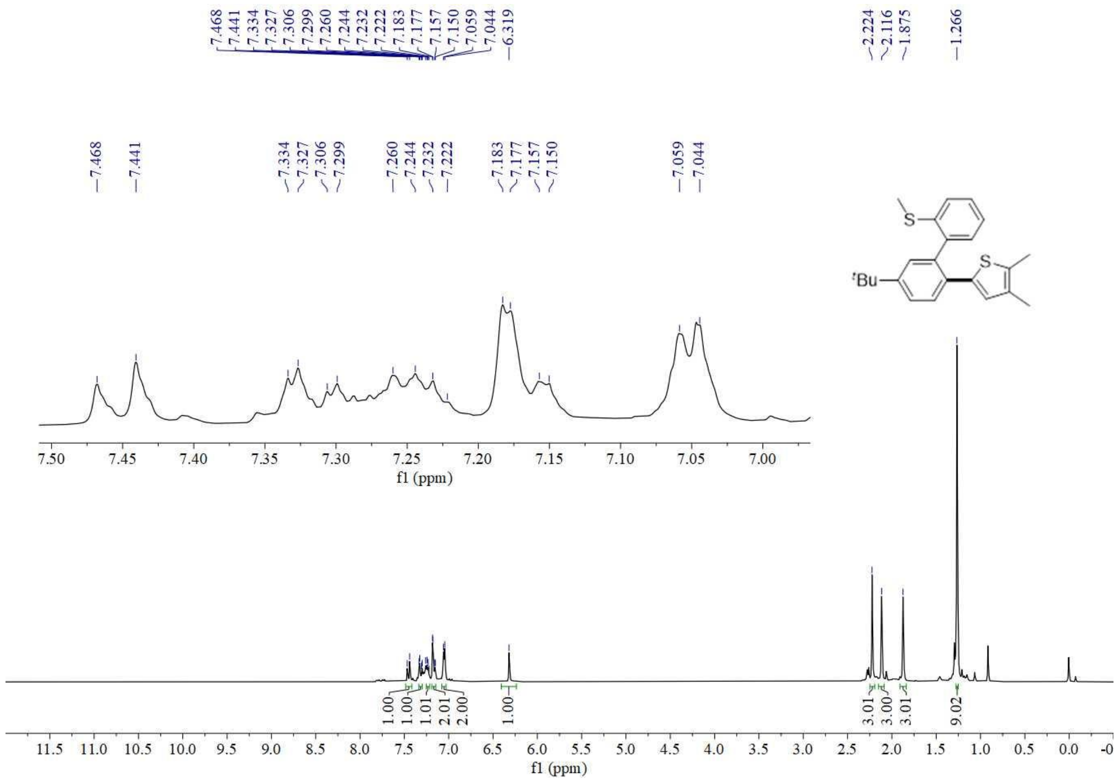

$^{13}C\{^1H\}$ NMR spectrum of **3m** in $CDCl_3$ (75 MHz)

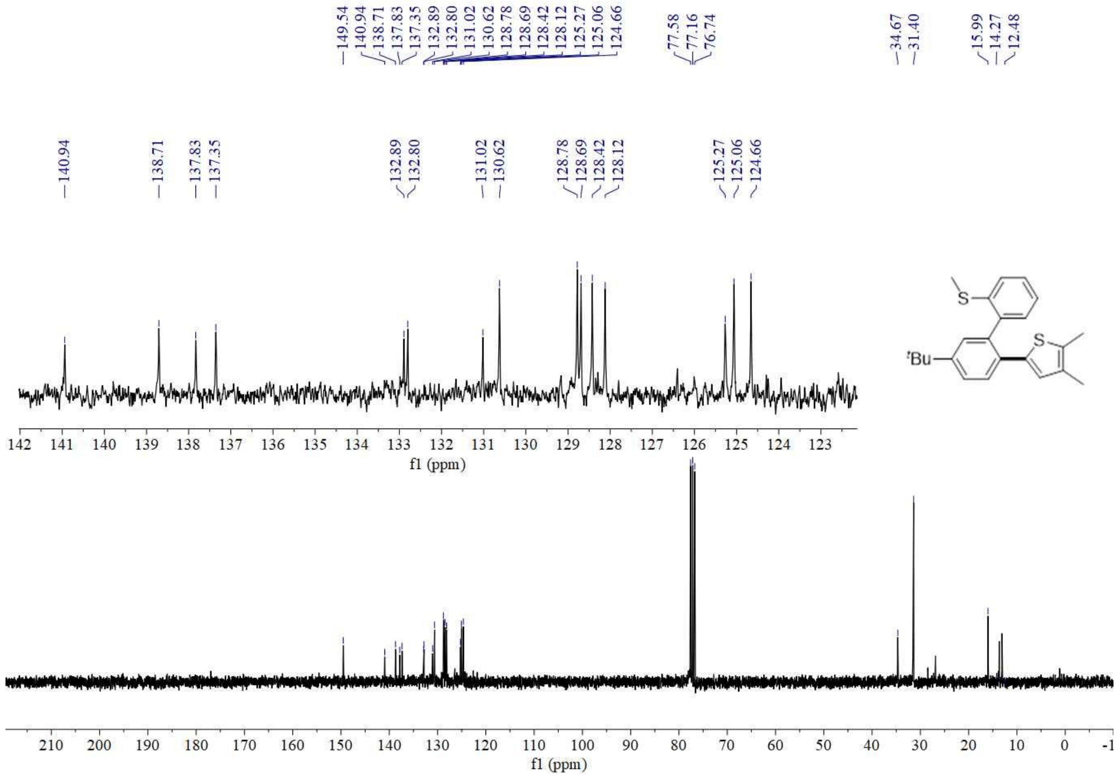

^{1}H NMR spectrum of **3n** in $CDCl_3$ (300 MHz)

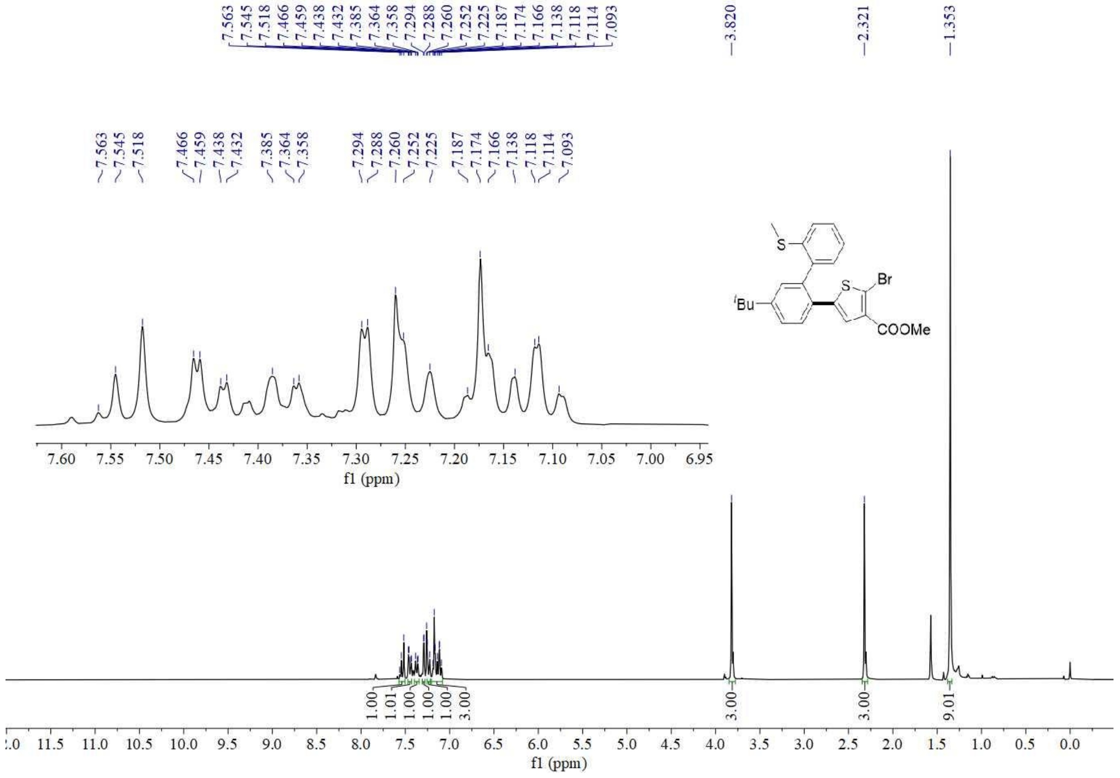

$^{13}C\{^{1}H\}$ NMR spectrum of **3n** in $CDCl_3$ (75 MHz)

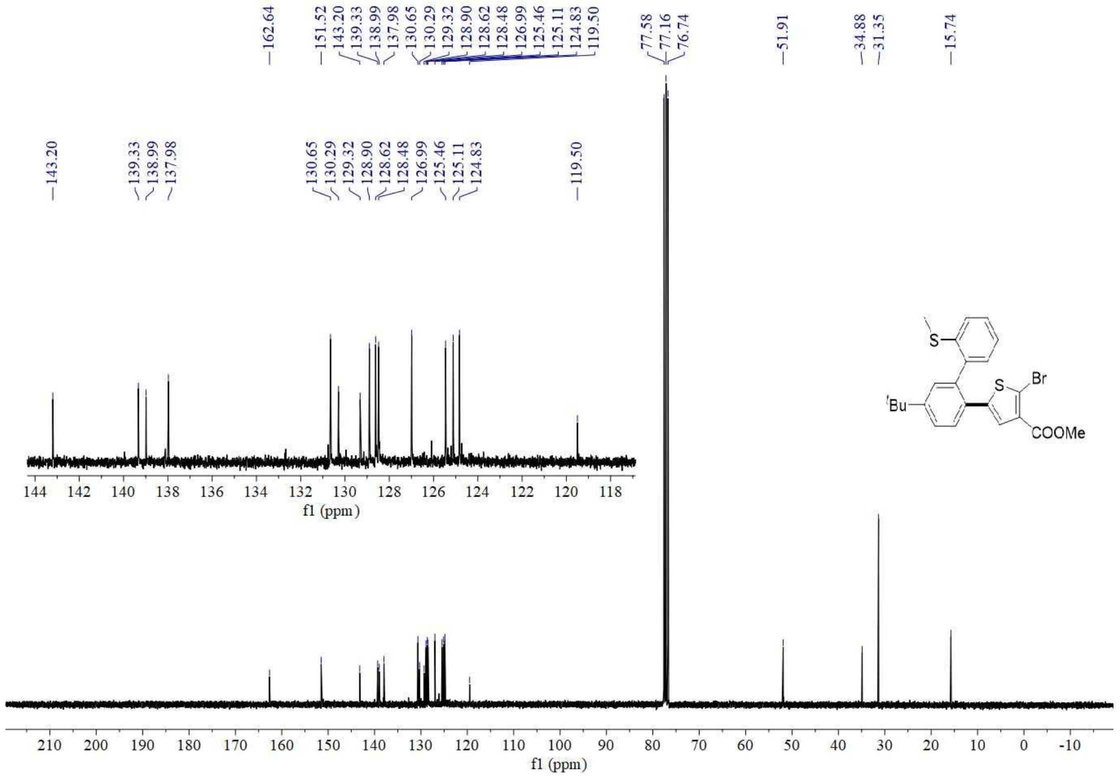

^{1}H NMR spectrum of **3o** in $CDCl_3$ (300 MHz)

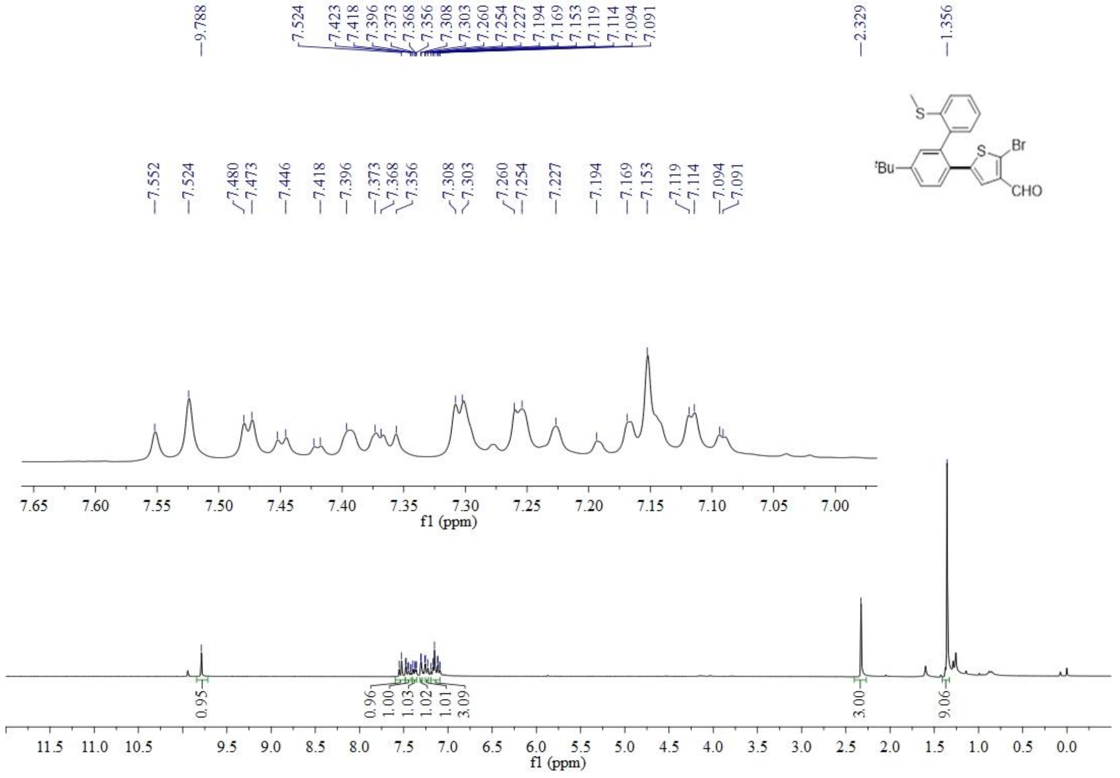

^{13}C{^{1}H} NMR spectrum of **3o** in $CDCl_3$ (75 MHz)

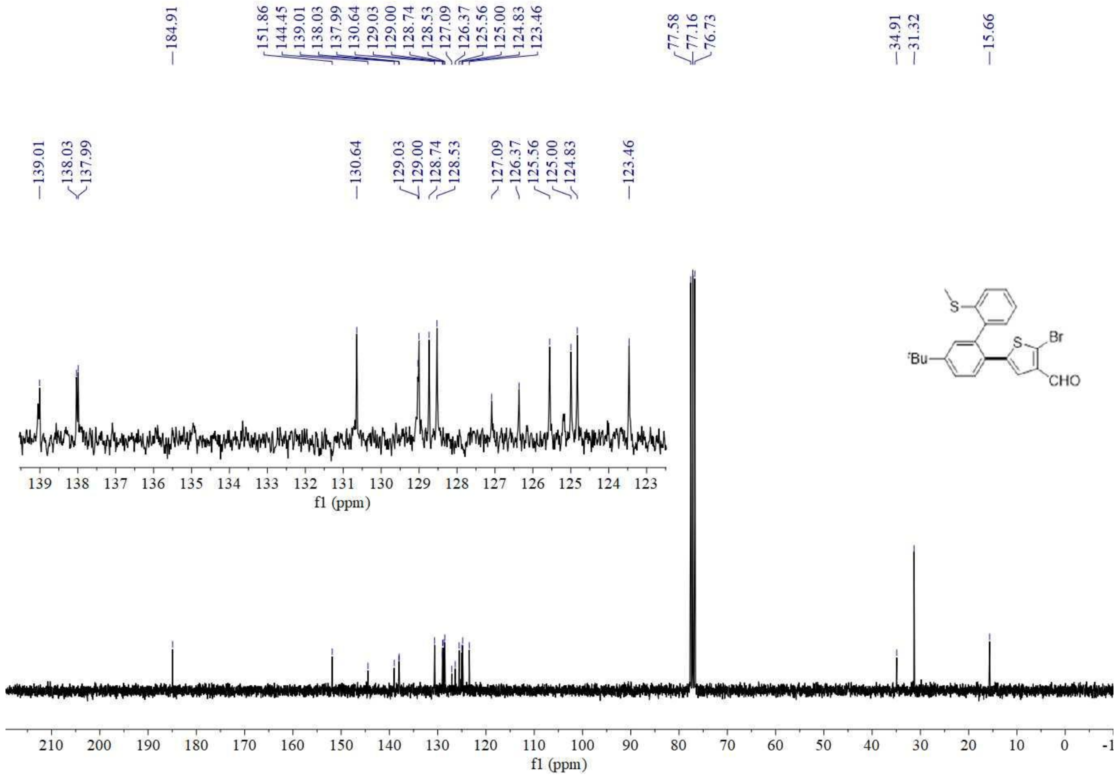

^{1}H NMR spectrum of **3p** in $CDCl_3$ (300 MHz)

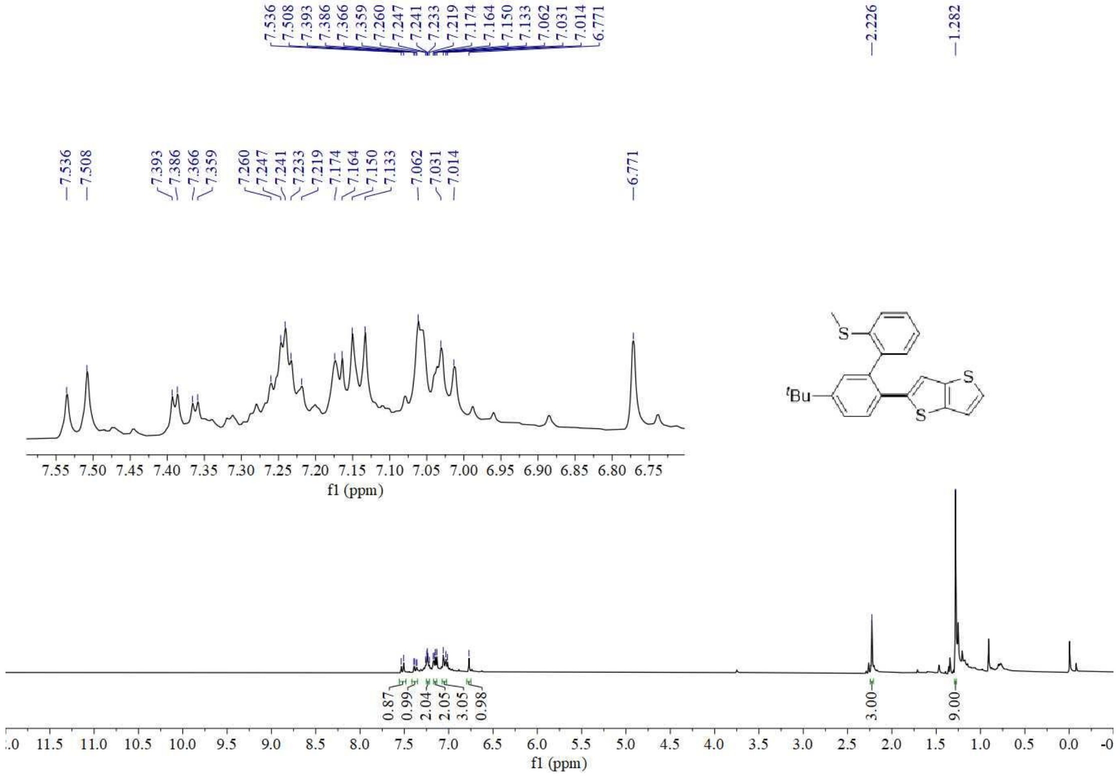

^{13}C{^{1}H} NMR spectrum of **3p** in $CDCl_3$ (75 MHz)

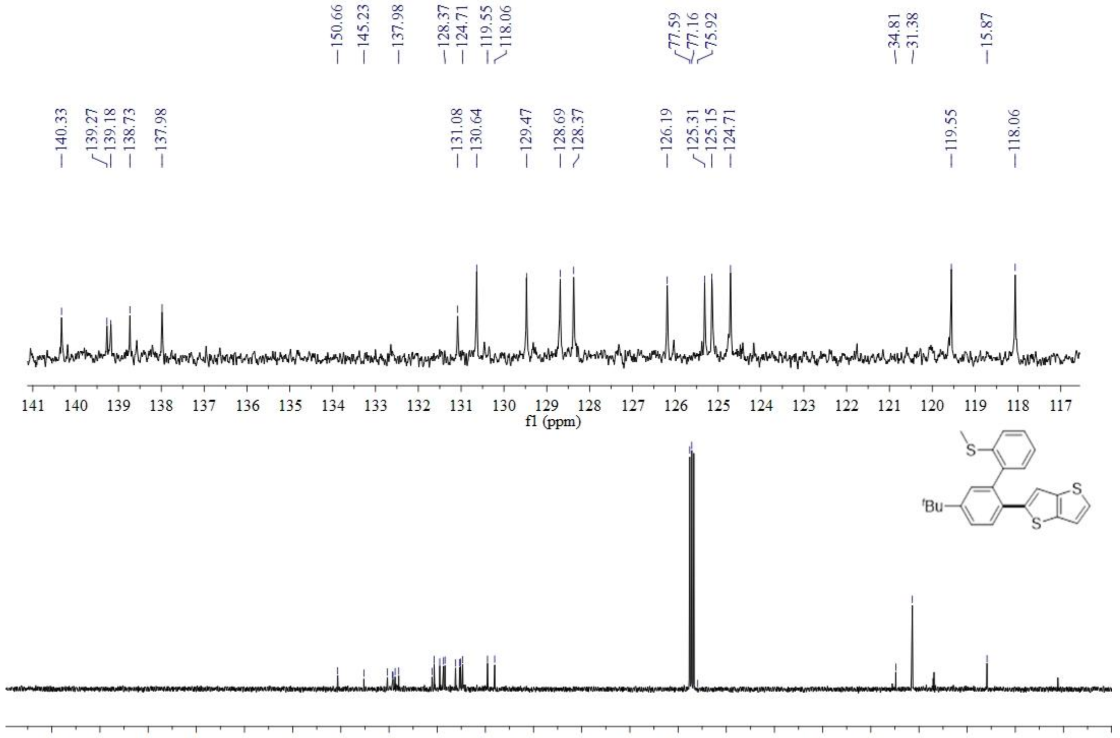

^{1}H NMR spectrum of **3q** in $CDCl_3$ (300 MHz)

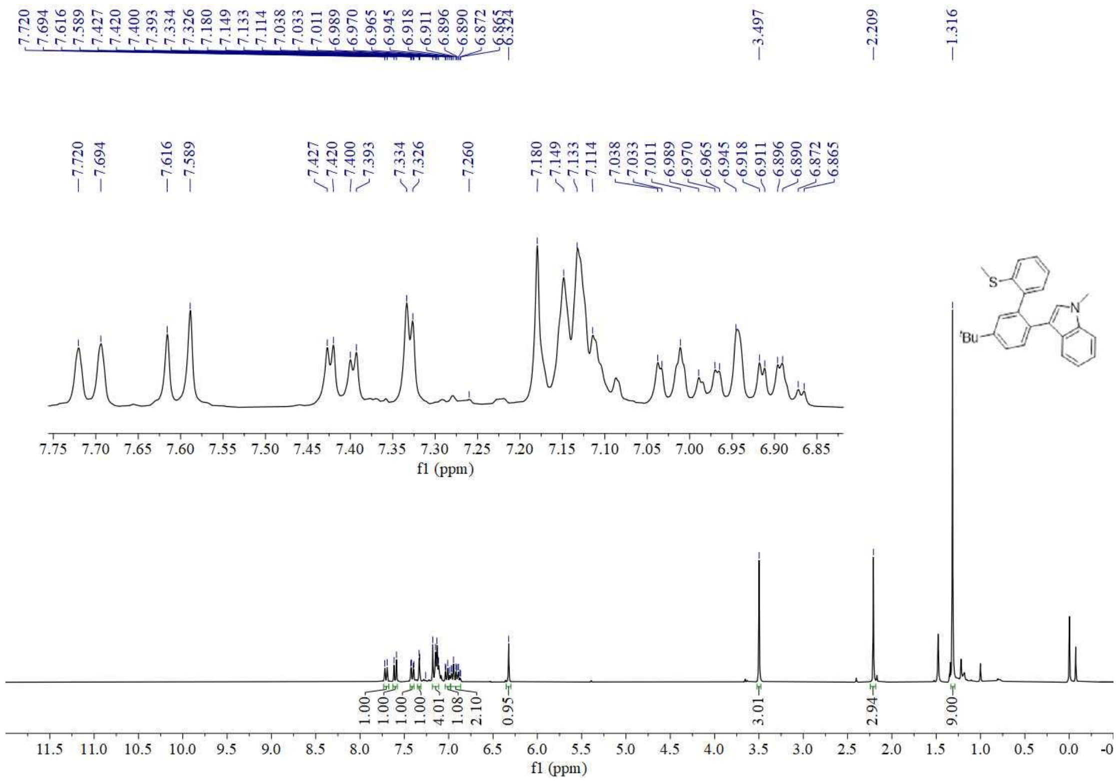

^{13}C{^{1}H} NMR spectrum of **3q** in $CDCl_3$ (75 MHz)

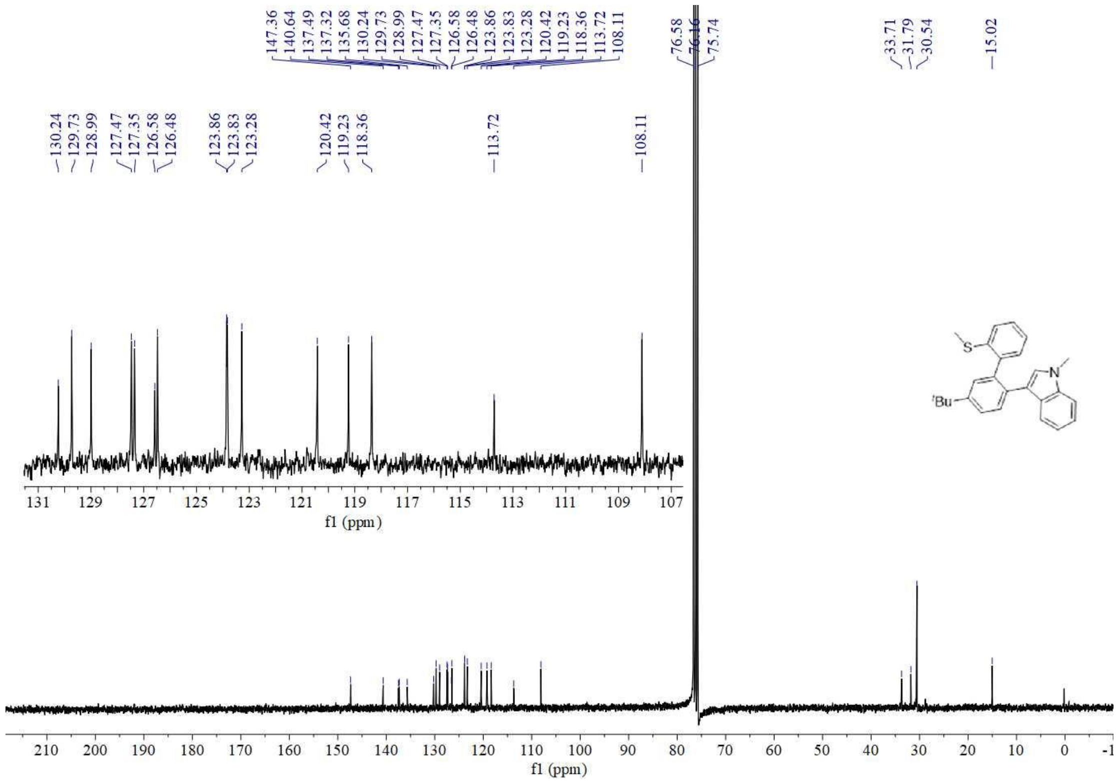

^{1}H NMR spectrum of **4a** in $CDCl_3$ (300 MHz)

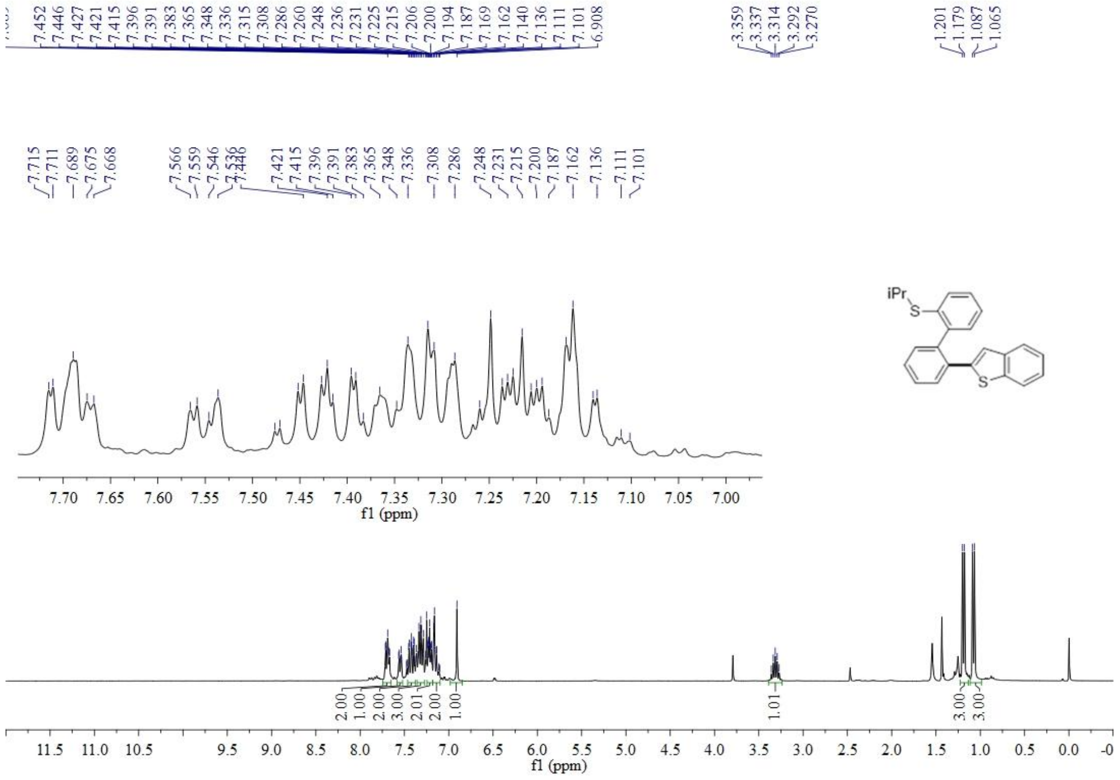

^{13}C{^{1}H} NMR spectrum of **4a** in $CDCl_3$ (75 MHz)

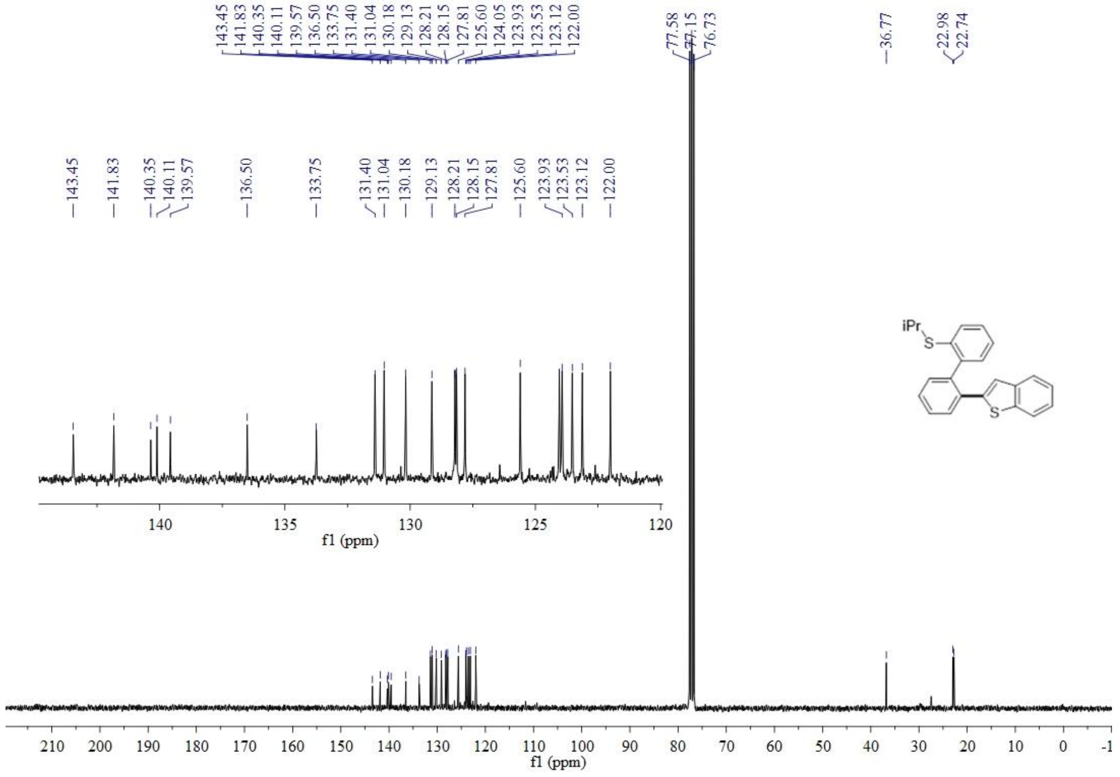

^{1}H NMR spectrum of **4b** in $CDCl_3$ (300 MHz)

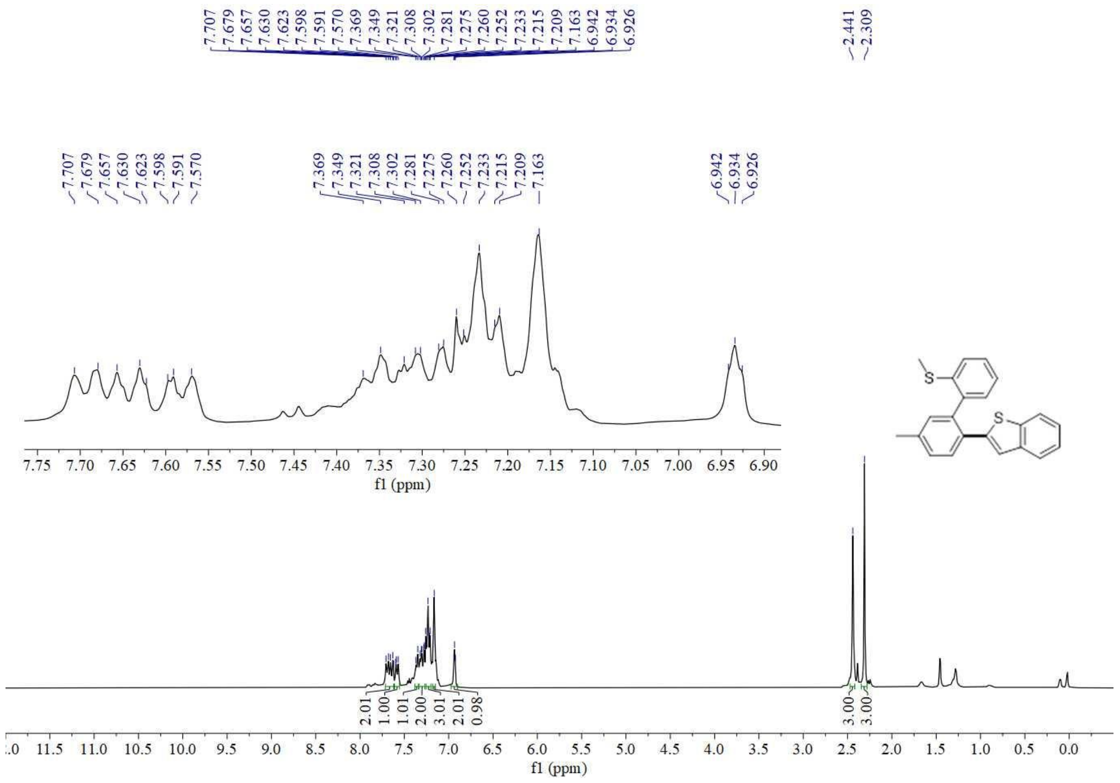

$^{13}C\{^{1}H\}$ NMR spectrum of **4b** in $CDCl_3$ (75 MHz)

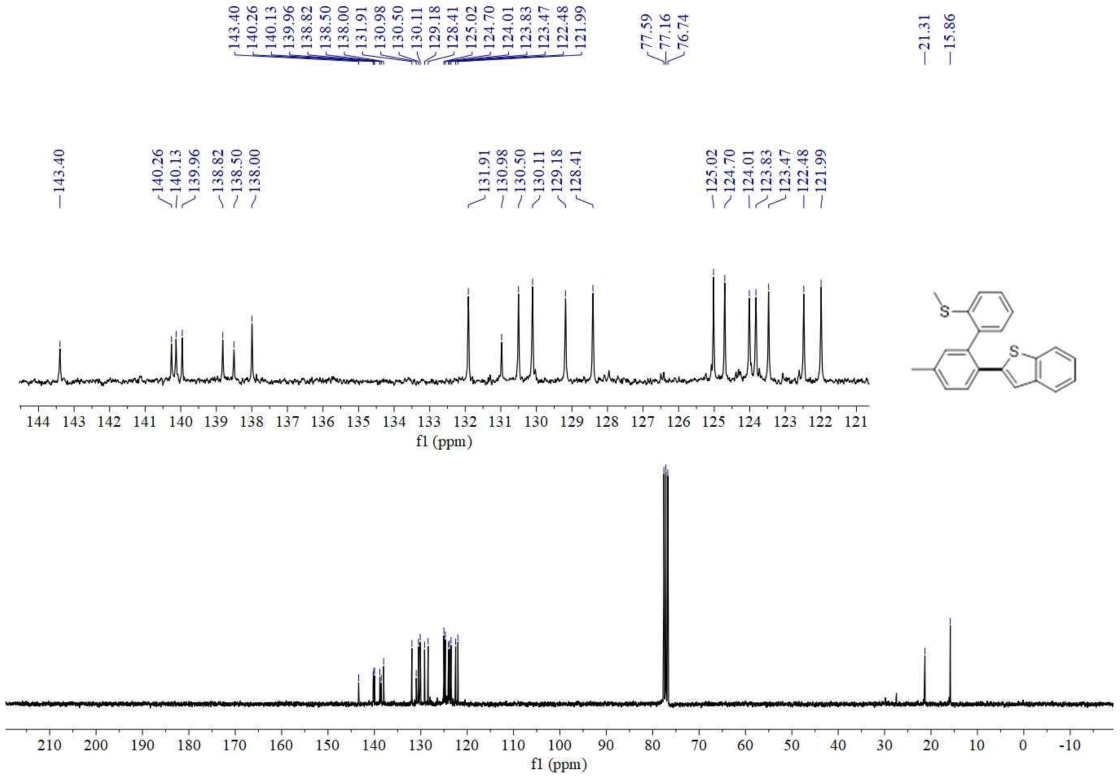

^{1}H NMR spectrum of **4c** in $CDCl_3$ (300 MHz)

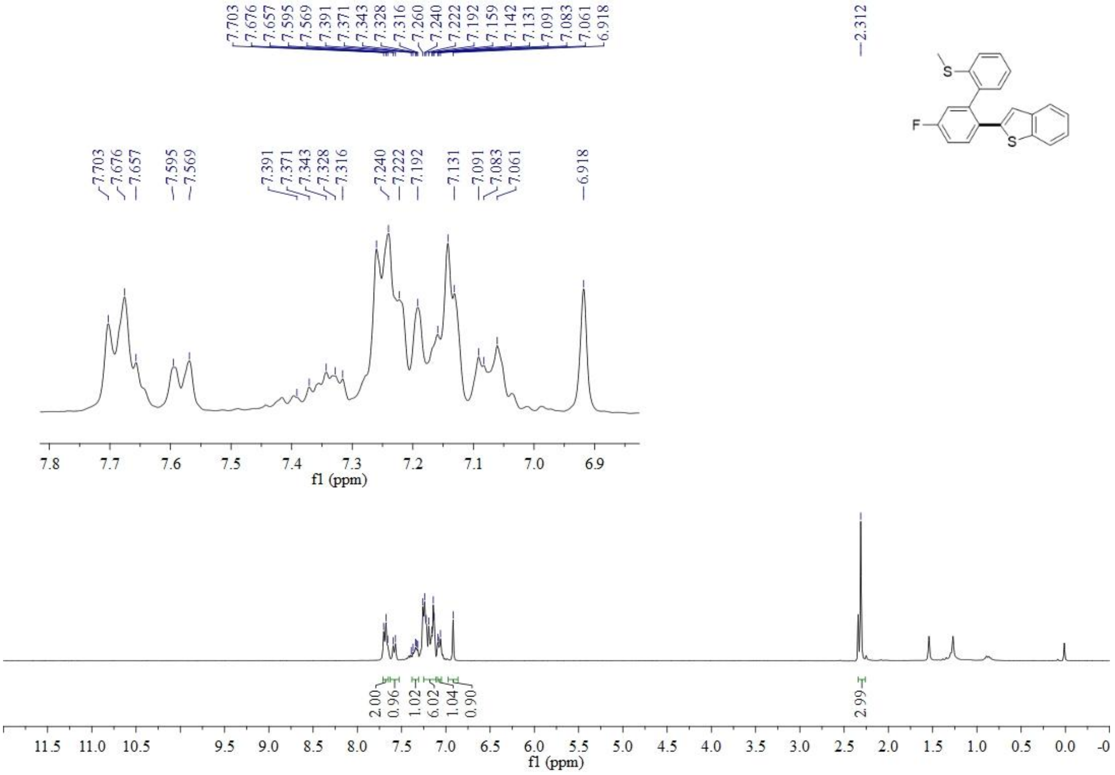

^{13}C{^{1}H} NMR spectrum of **4c** in $CDCl_3$ (75 MHz)

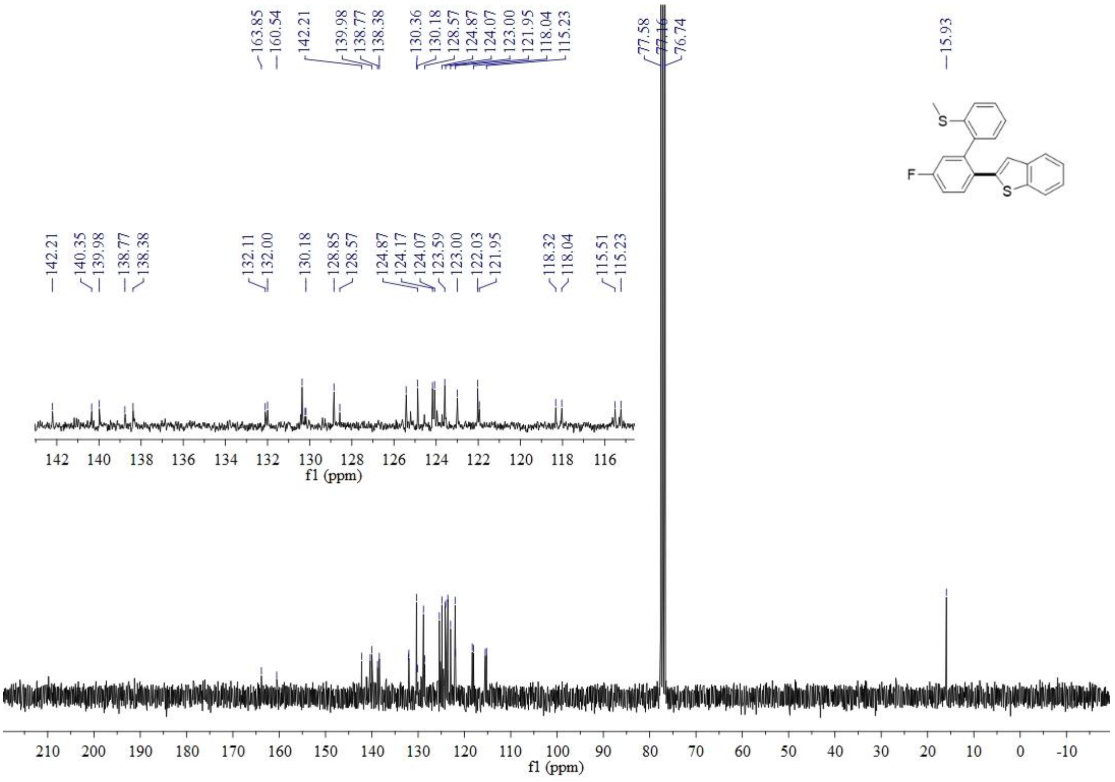

^{1}H NMR spectrum of **4d** in $CDCl_3$ (300 MHz)

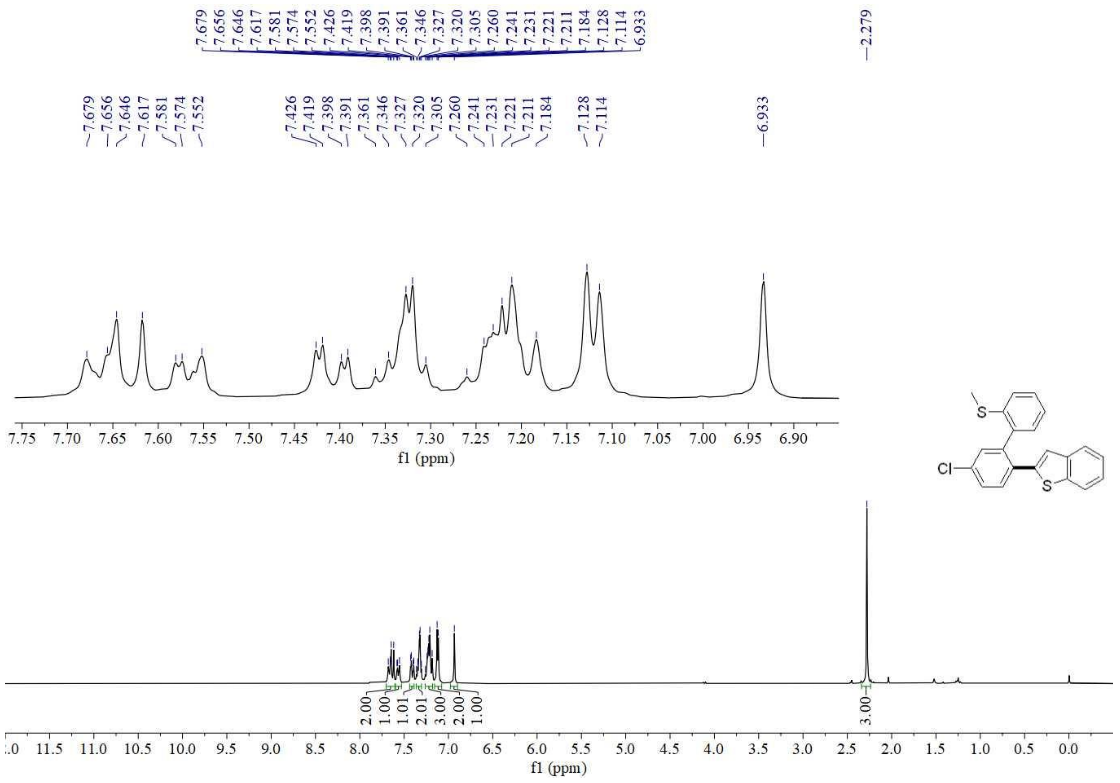

$^{13}C\{^{1}H\}$ NMR spectrum of **4d** in $CDCl_3$ (75 MHz)

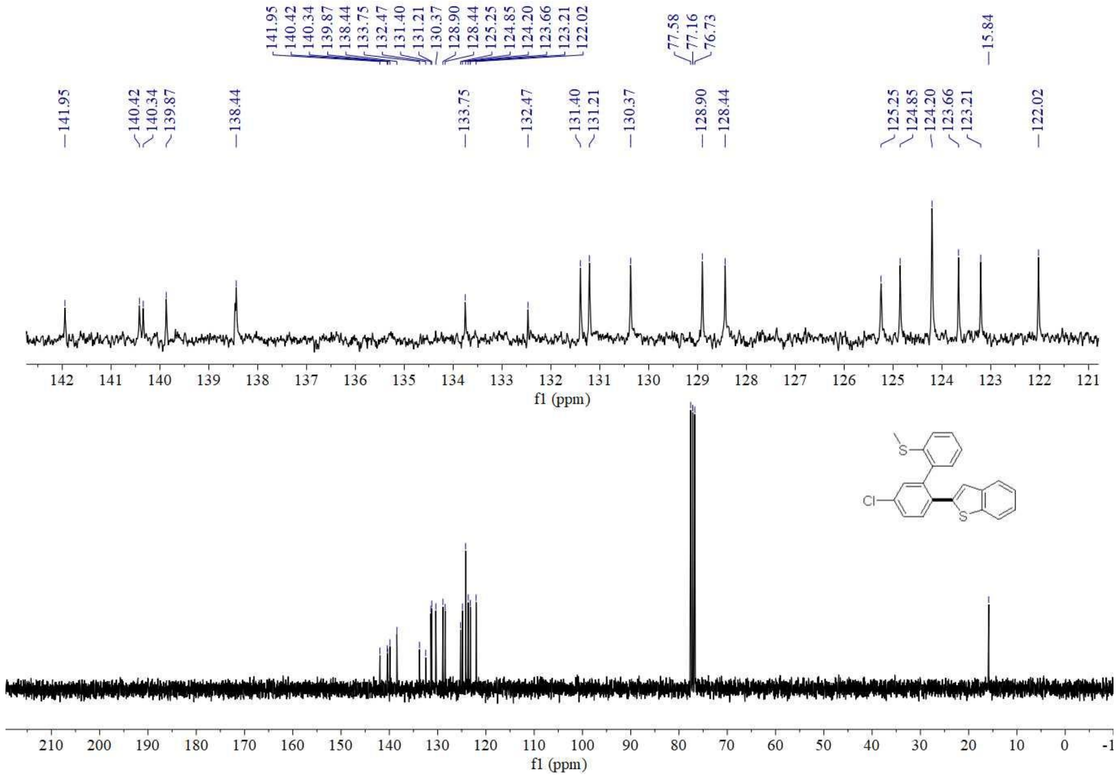

^{1}H NMR spectrum of **4e** in $CDCl_3$ (300 MHz)

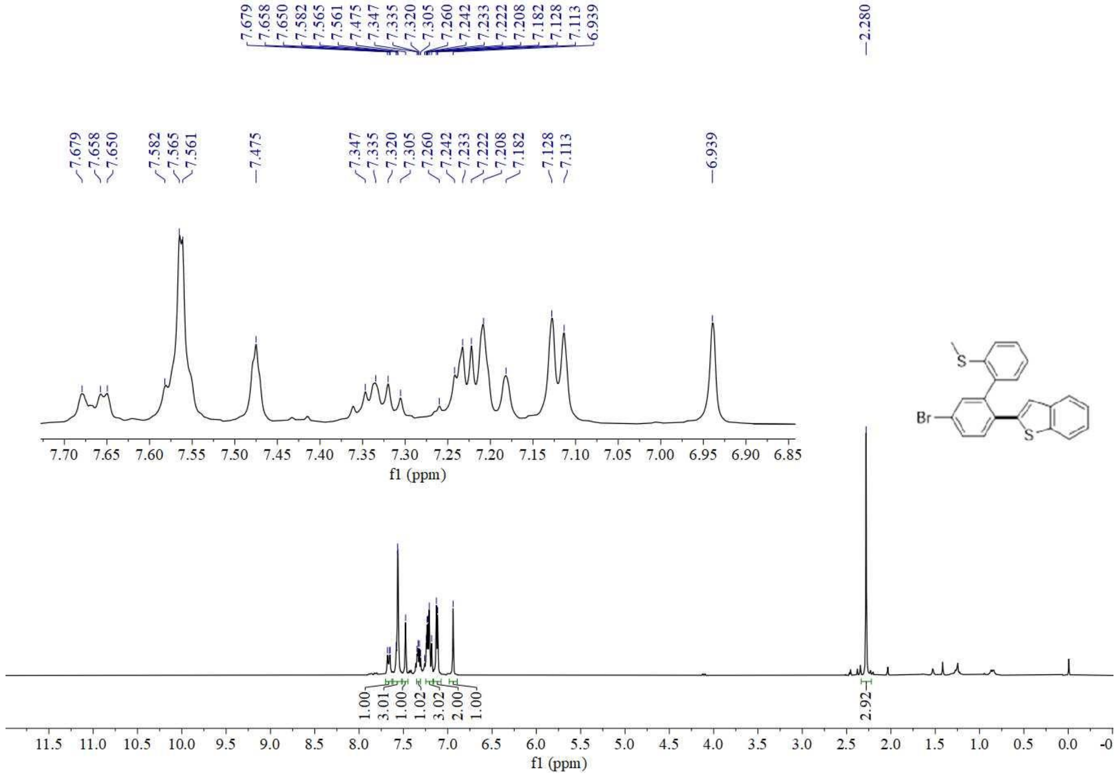

^{13}C{^{1}H} NMR spectrum of **4e** in $CDCl_3$ (75 MHz)

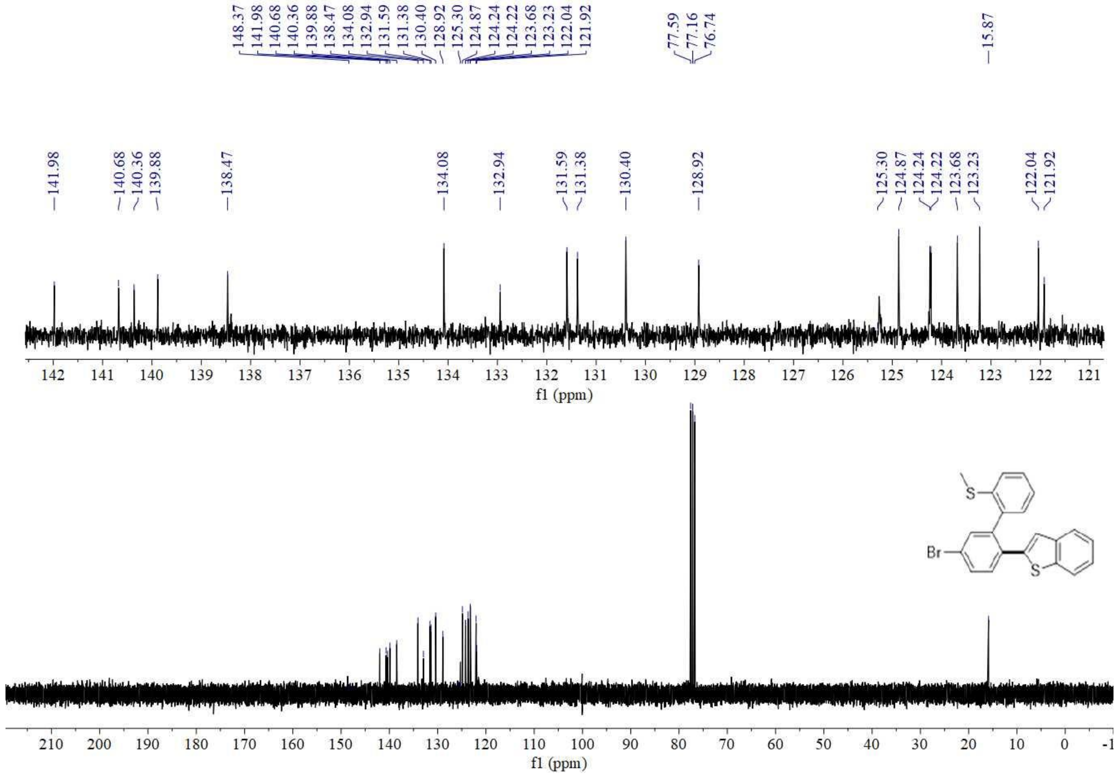

^{1}H NMR spectrum of **4f** in $CDCl_3$ (300 MHz)

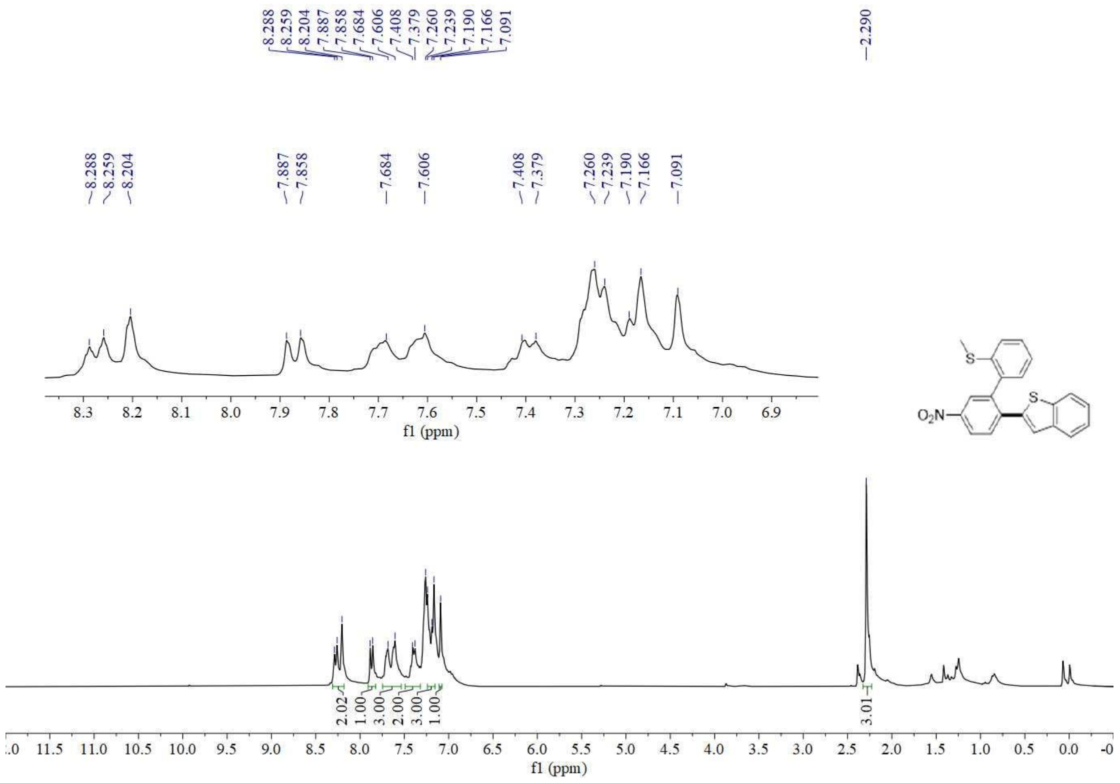

^{13}C{^{1}H} NMR spectrum of **4f** in $CDCl_3$ (75 MHz)

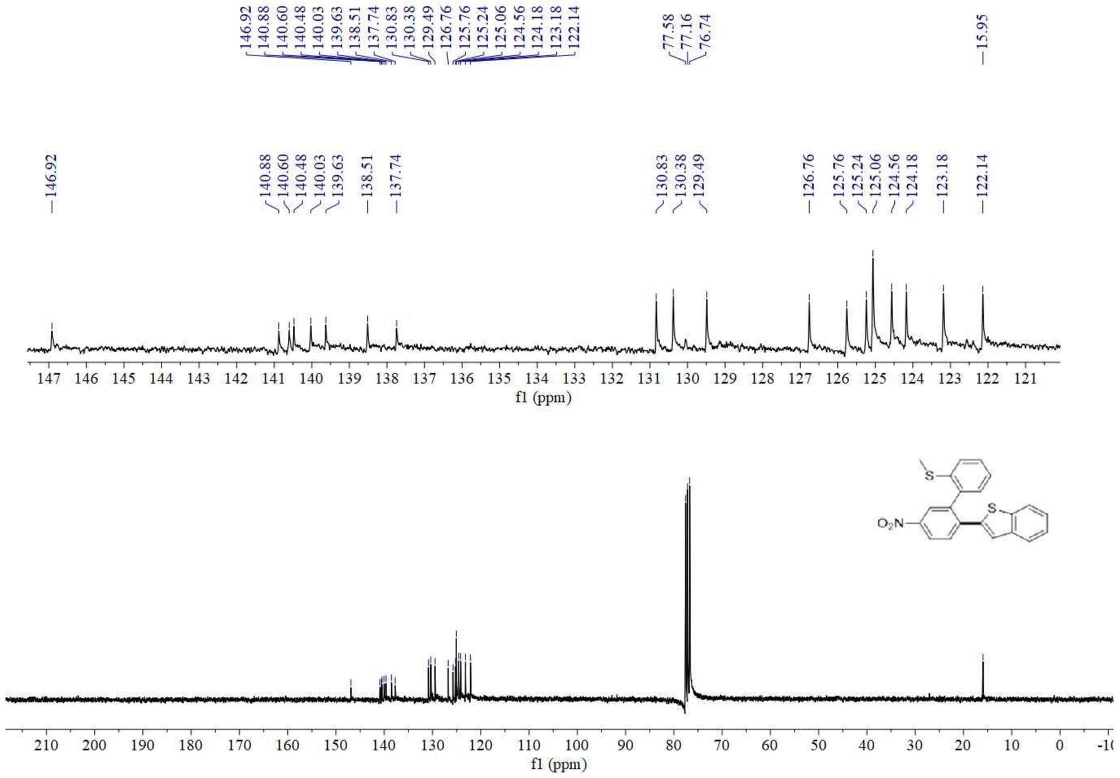

^{1}H NMR spectrum of **4g** in $CDCl_3$ (300 MHz)

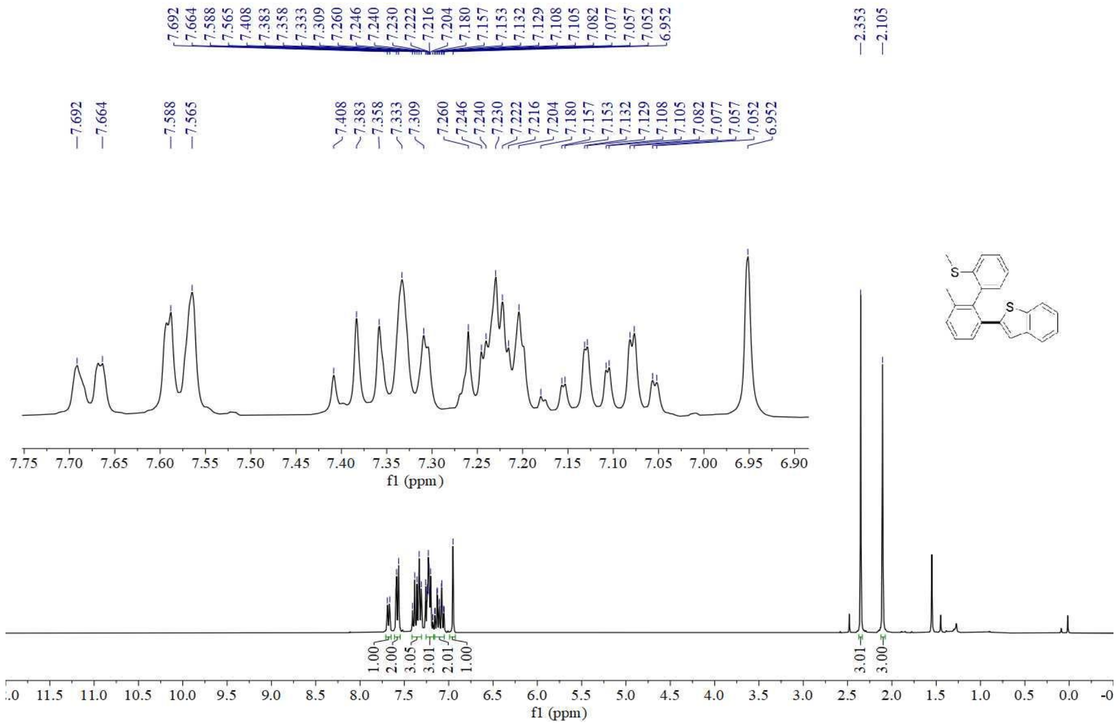

^{13}C{^{1}H} NMR spectrum of **4g** in $CDCl_3$ (75 MHz)

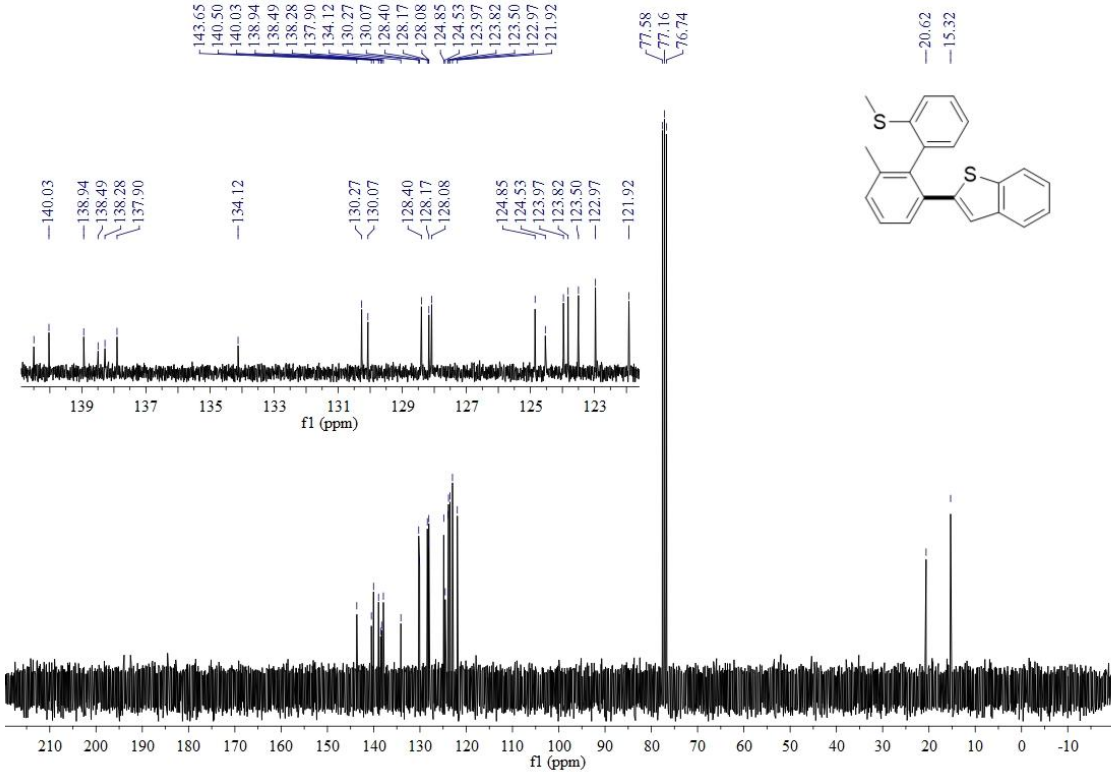

^{1}H NMR spectrum of **4h** in $CDCl_3$ (300 MHz)

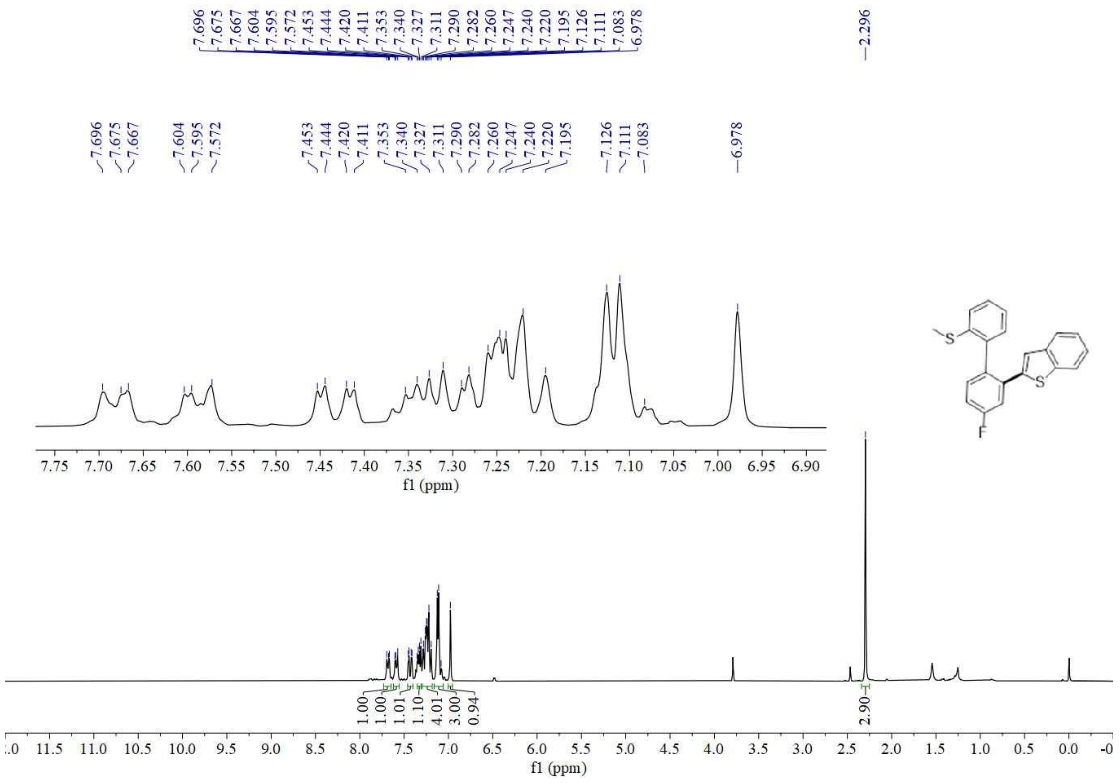

$^{13}C\{^1H\}$ NMR spectrum of **4h** in $CDCl_3$ (75 MHz)

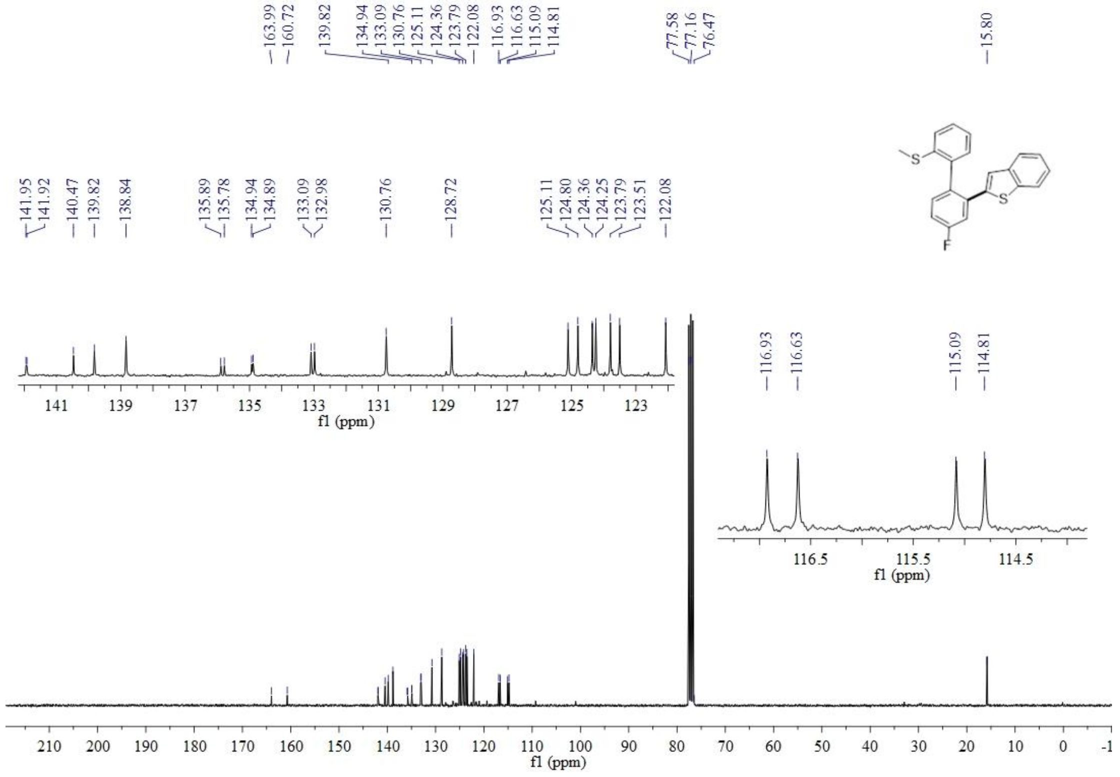

^{1}H NMR spectrum of **4i** in $CDCl_3$ (300 MHz)

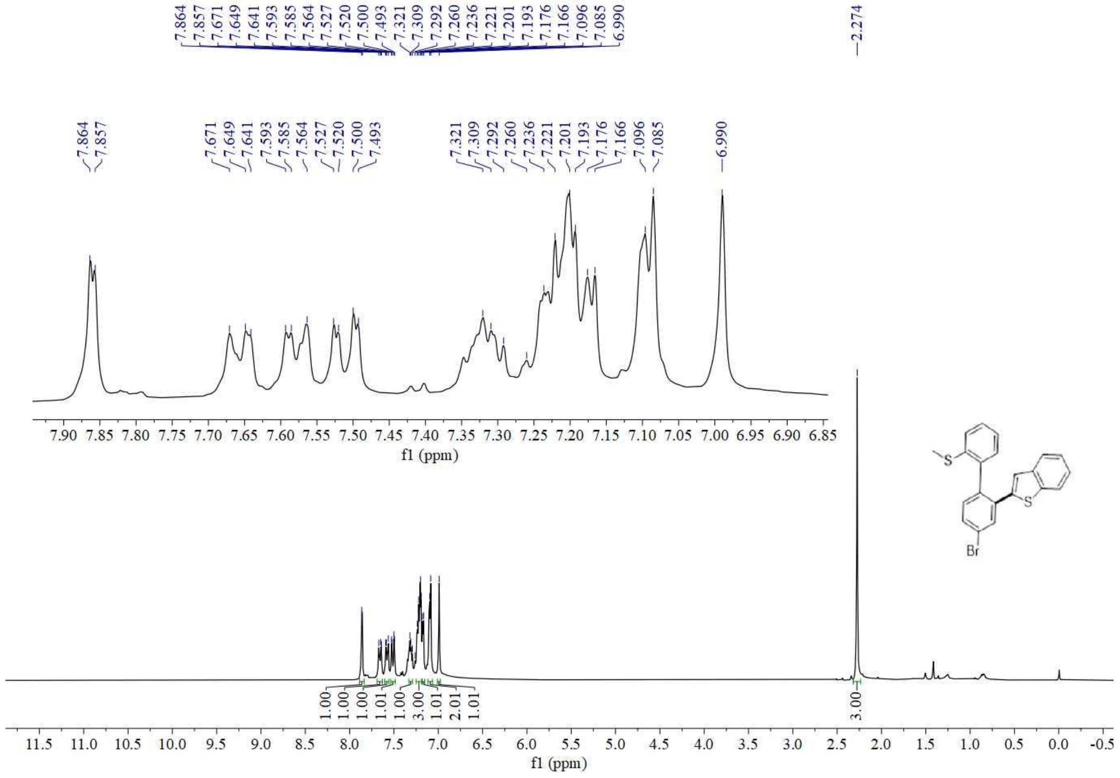

$^{13}C\{^{1}H\}$ NMR spectrum of **4i** in $CDCl_3$ (75 MHz)

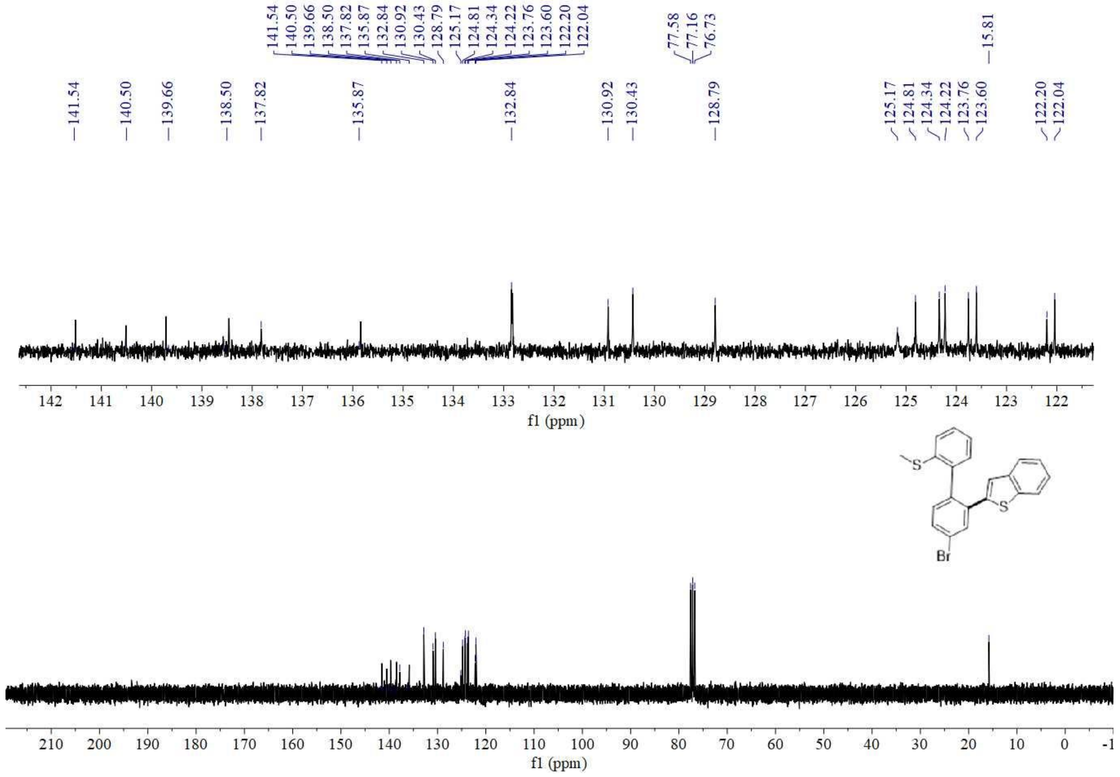

^{1}H NMR spectrum of **4j** in $CDCl_3$ (300 MHz)

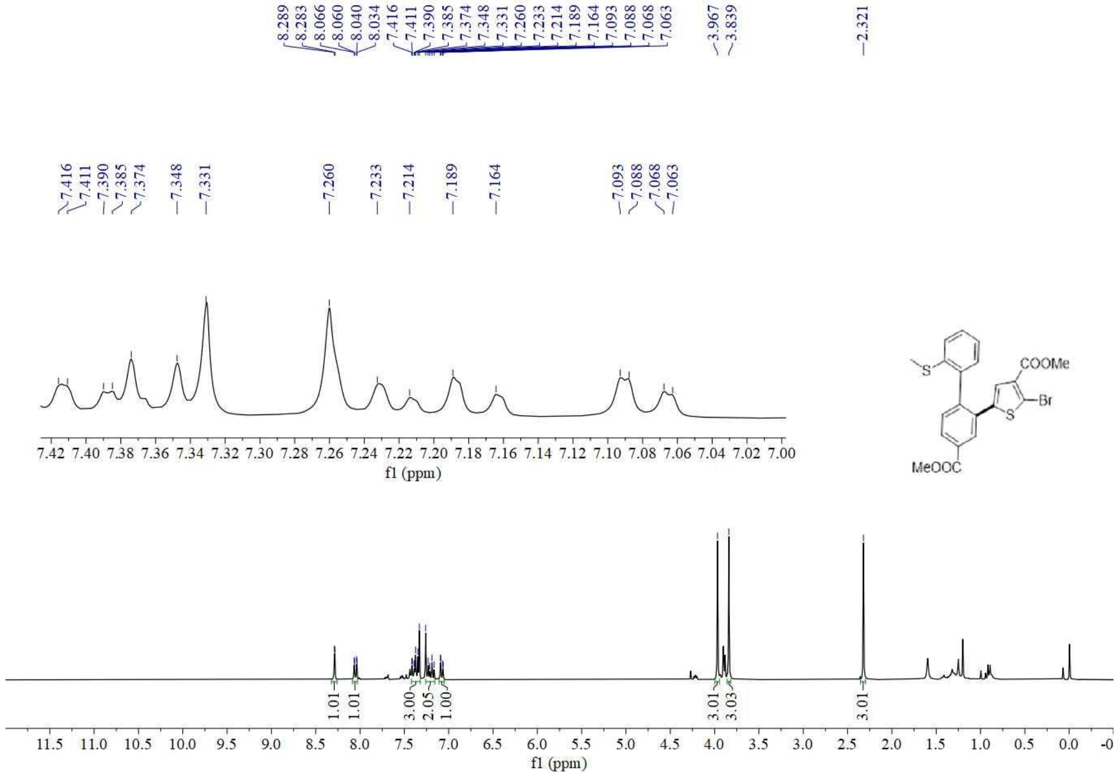

^{13}C{^{1}H} NMR spectrum of **4j** in $CDCl_3$ (75 MHz)

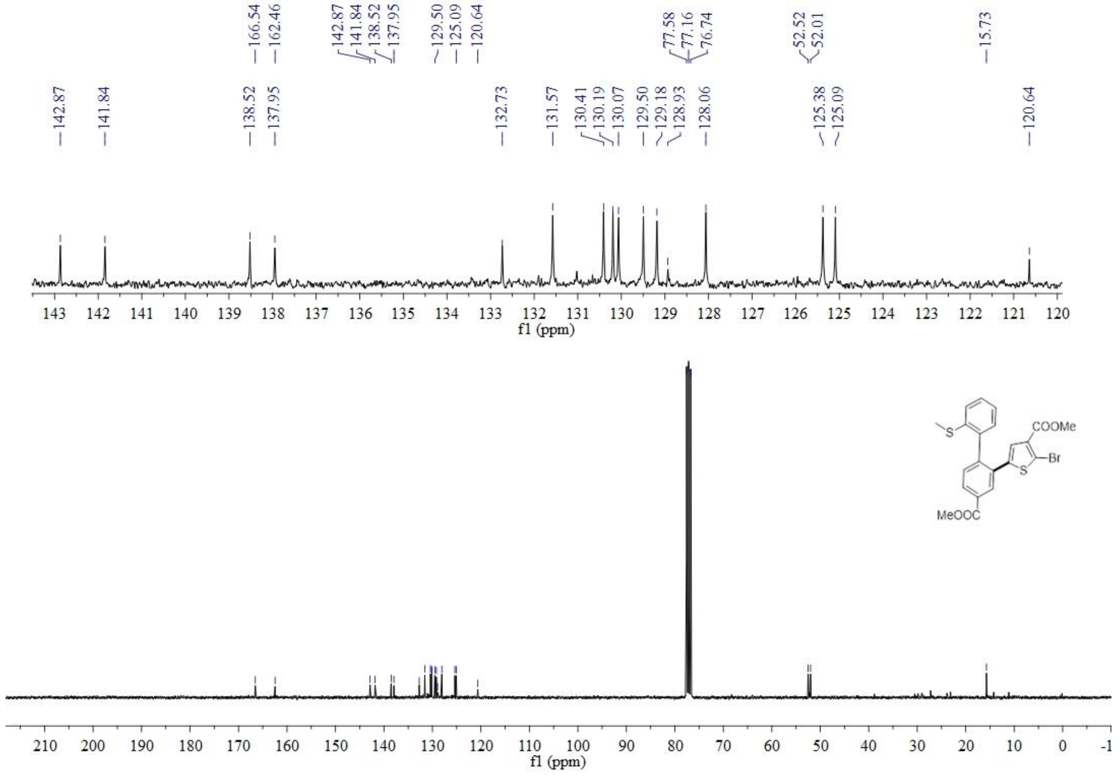

^{1}H NMR spectrum of **4k** in $CDCl_3$ (300 MHz)

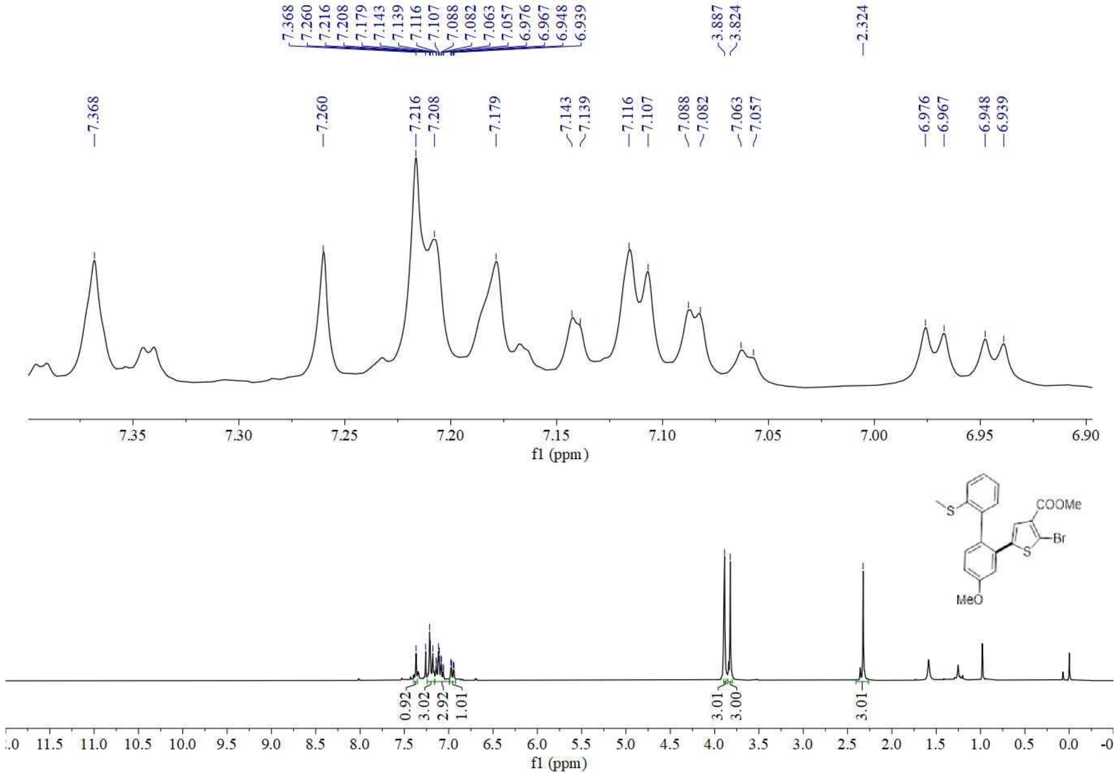

^{13}C{^{1}H} NMR spectrum of **4k** in $CDCl_3$ (75 MHz)

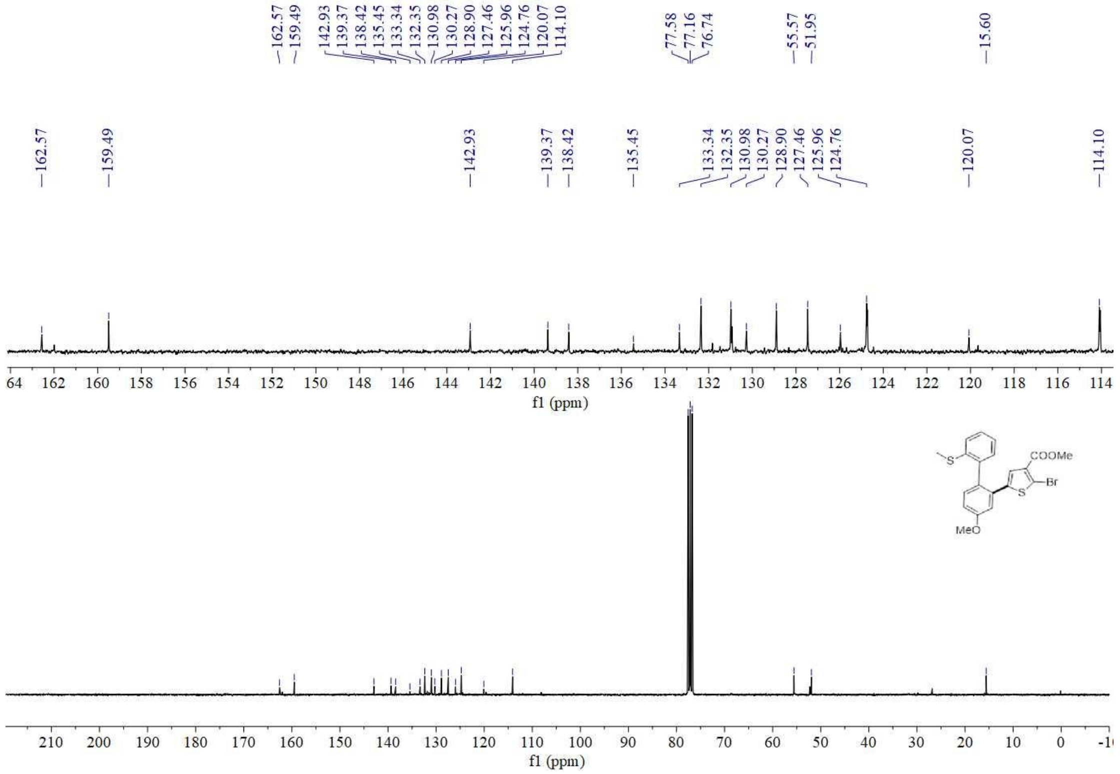

^{1}H NMR spectrum of **4k'** in $CDCl_3$ (300 MHz)

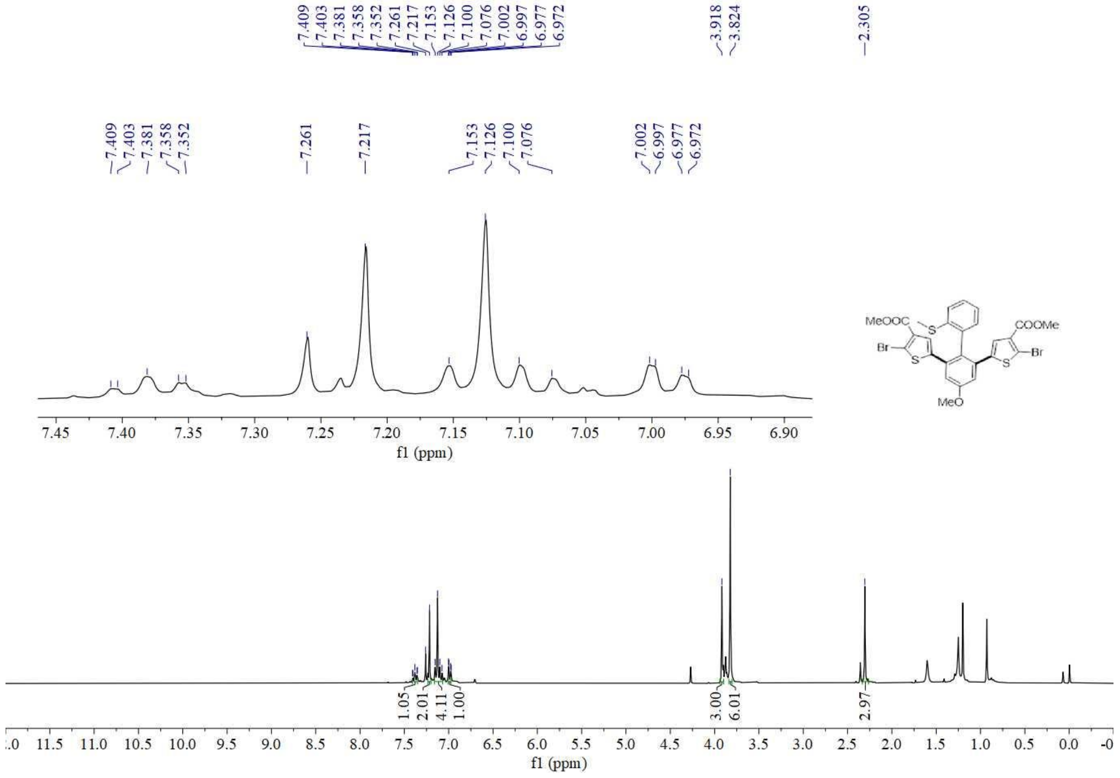

^{13}C{^{1}H} NMR spectrum of **4k'** in $CDCl_3$ (75 MHz)

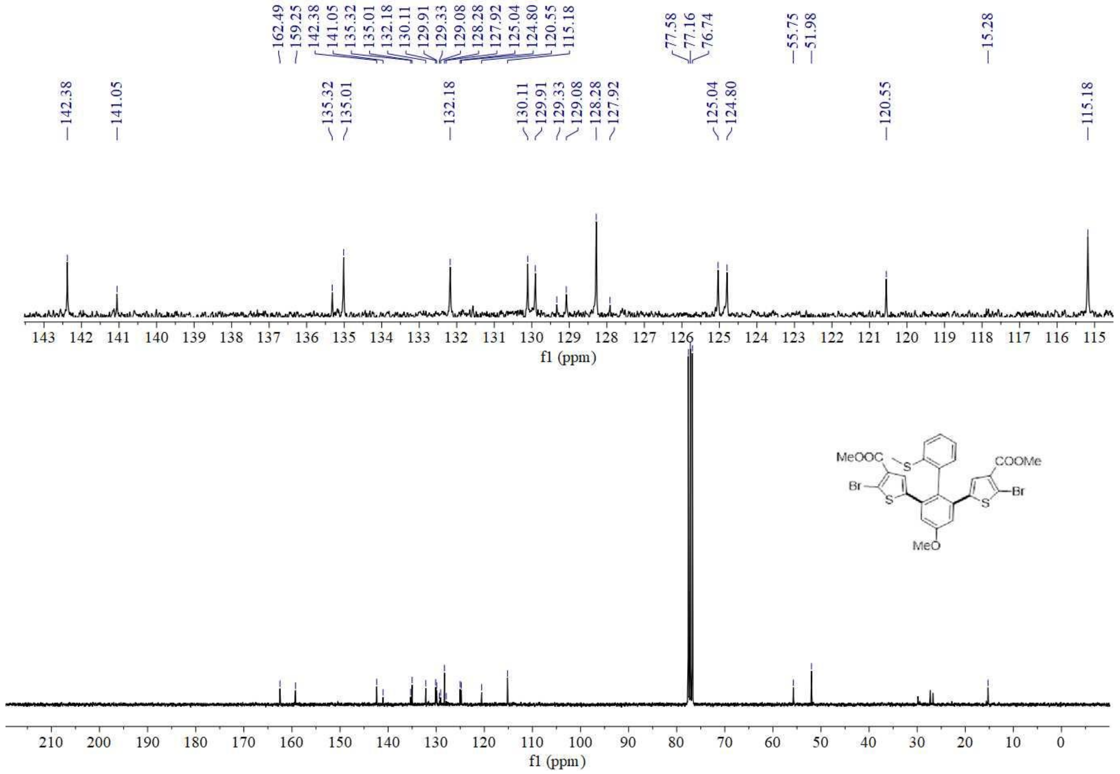

^{1}H NMR spectrum of **4l** in $CDCl_3$ (300 MHz)

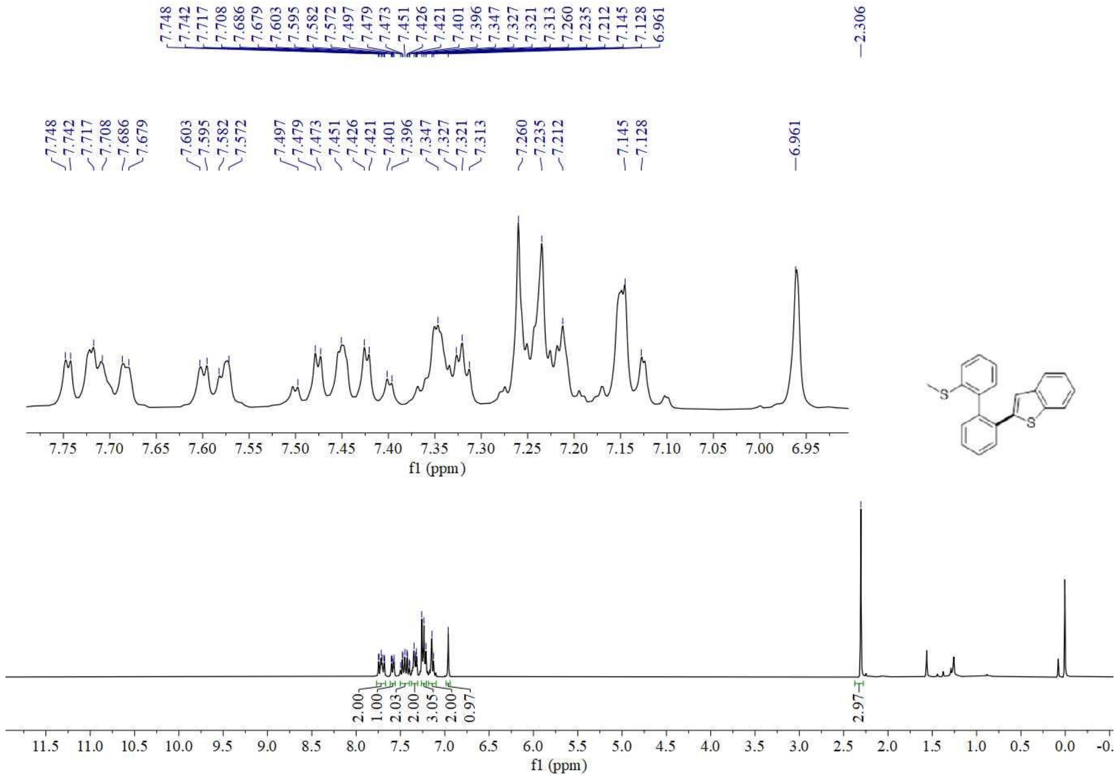

^{13}C{^{1}H} NMR spectrum of **4l** in $CDCl_3$ (75 MHz)

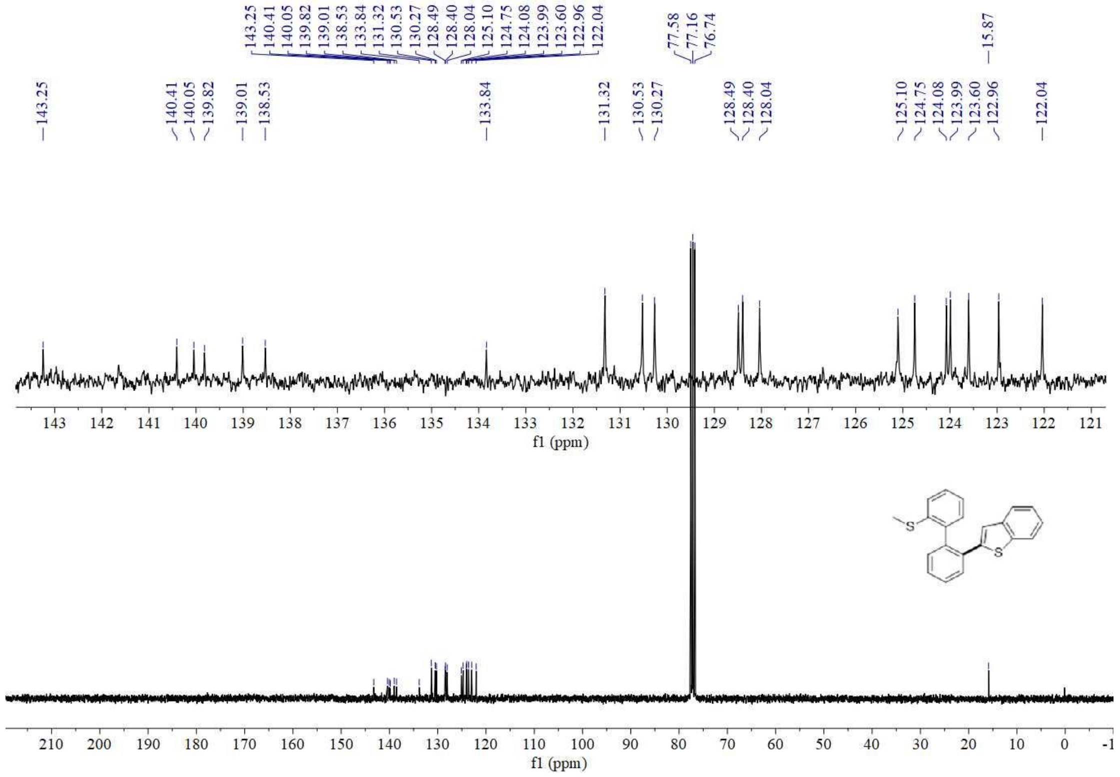

^{1}H NMR spectrum of **4l'** in $CDCl_3$ (300 MHz)

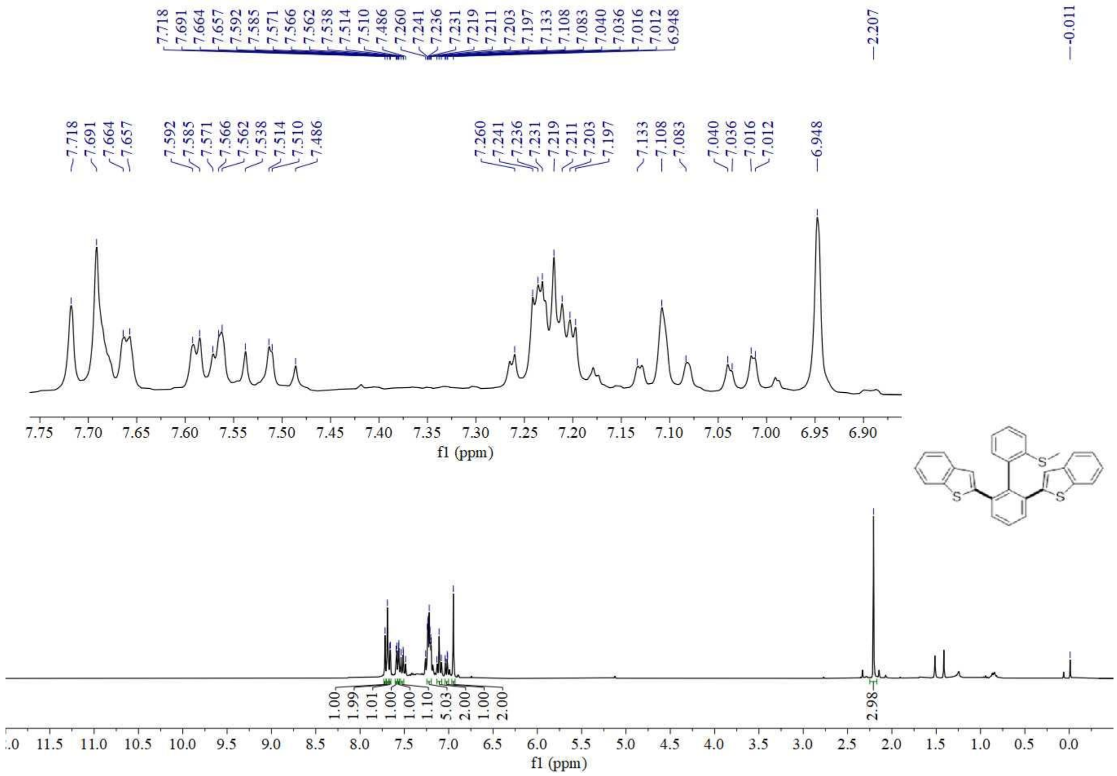

^{13}C{^{1}H} NMR spectrum of **4l'** in $CDCl_3$ (75 MHz)

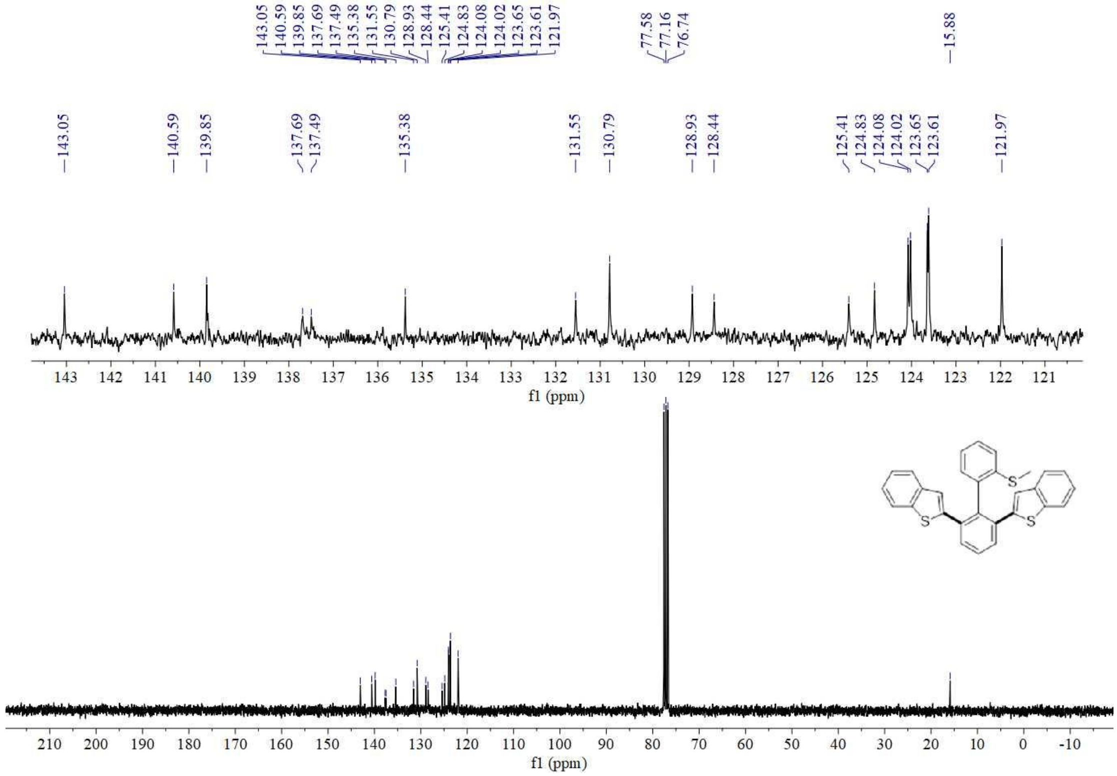

^1H NMR spectrum of **4m** in $CDCl_3$ (300 MHz)

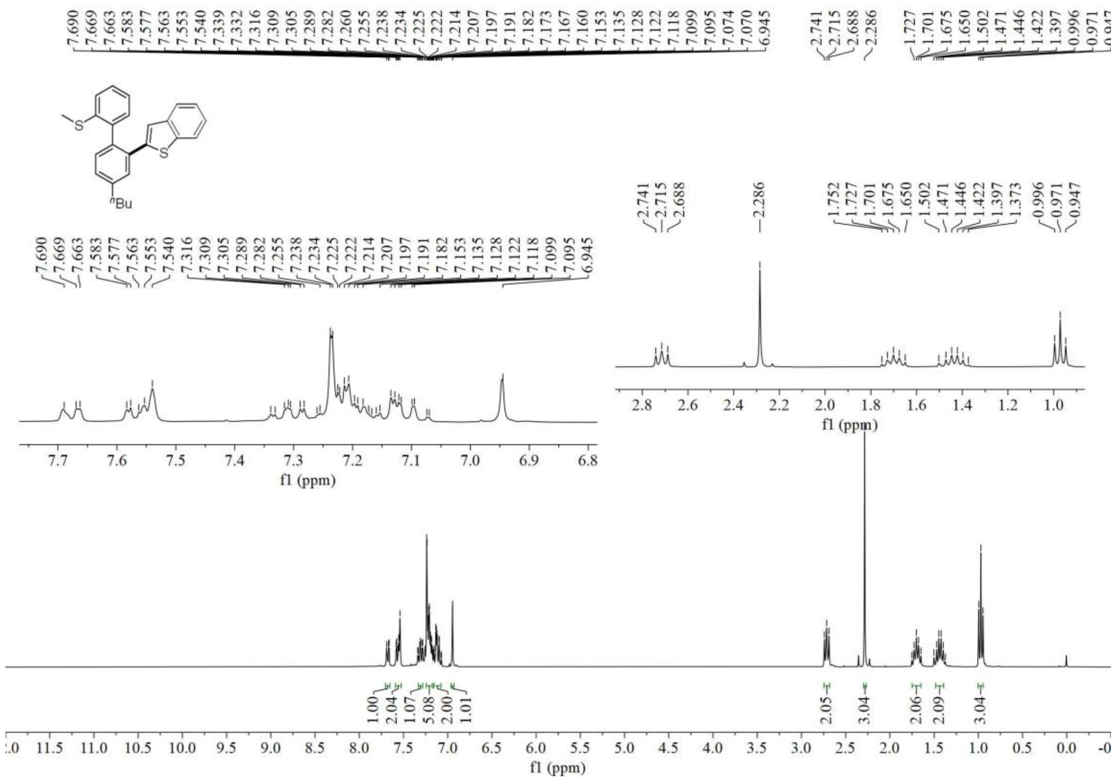

$^{13}C\{^1H\}$ NMR spectrum of **4m** in $CDCl_3$ (75 MHz)

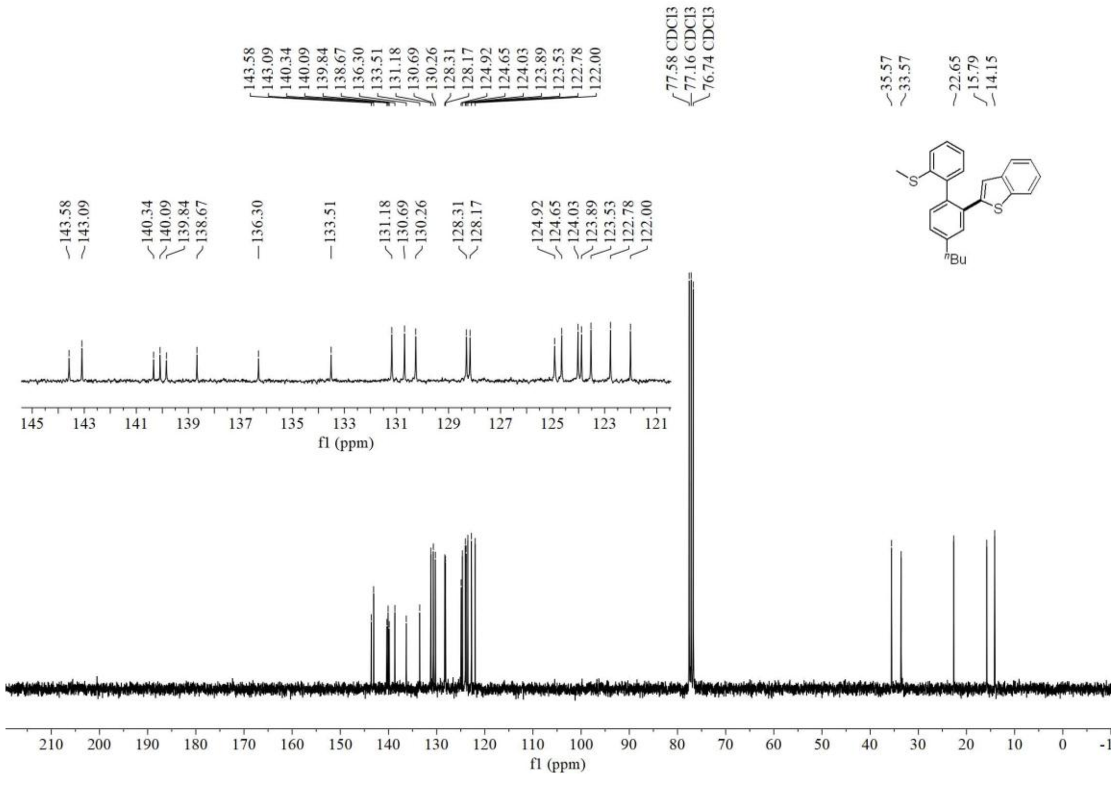

^{1}H NMR spectrum of **4m'** in $CDCl_3$ (300 MHz)

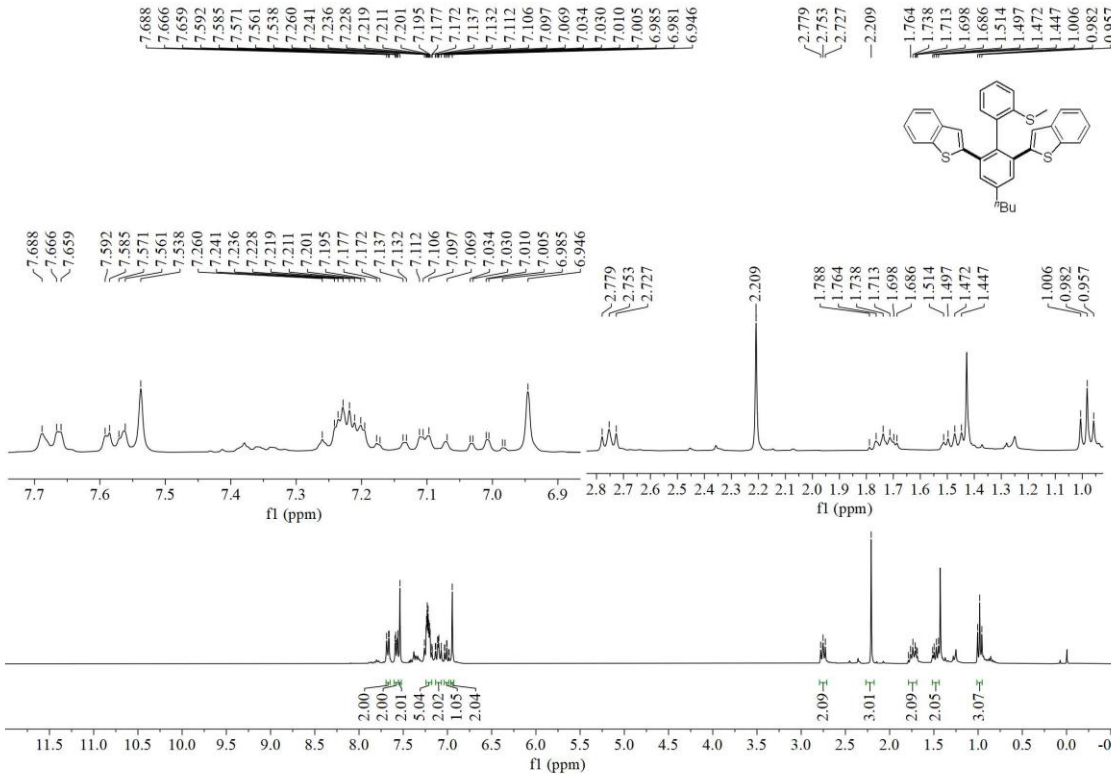

^{13}C{^{1}H} NMR spectrum of **4m'** in $CDCl_3$ (75 MHz)

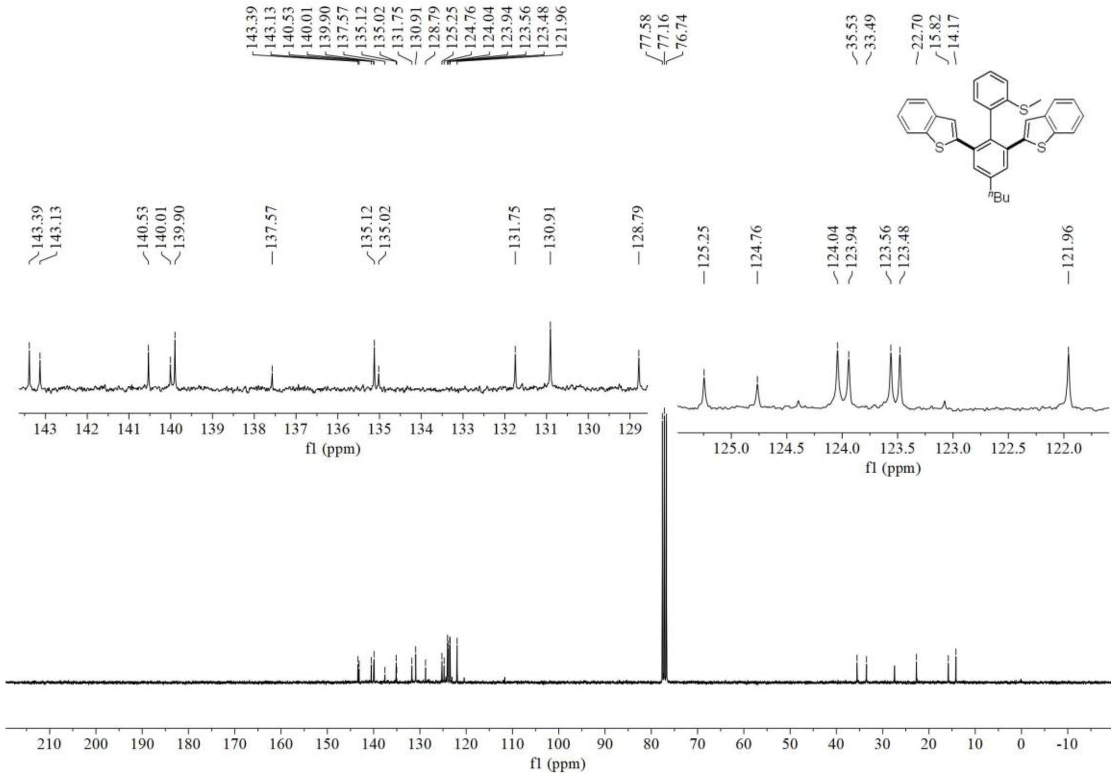

^{1}H NMR spectrum of **4n** in $CDCl_3$ (300 MHz)

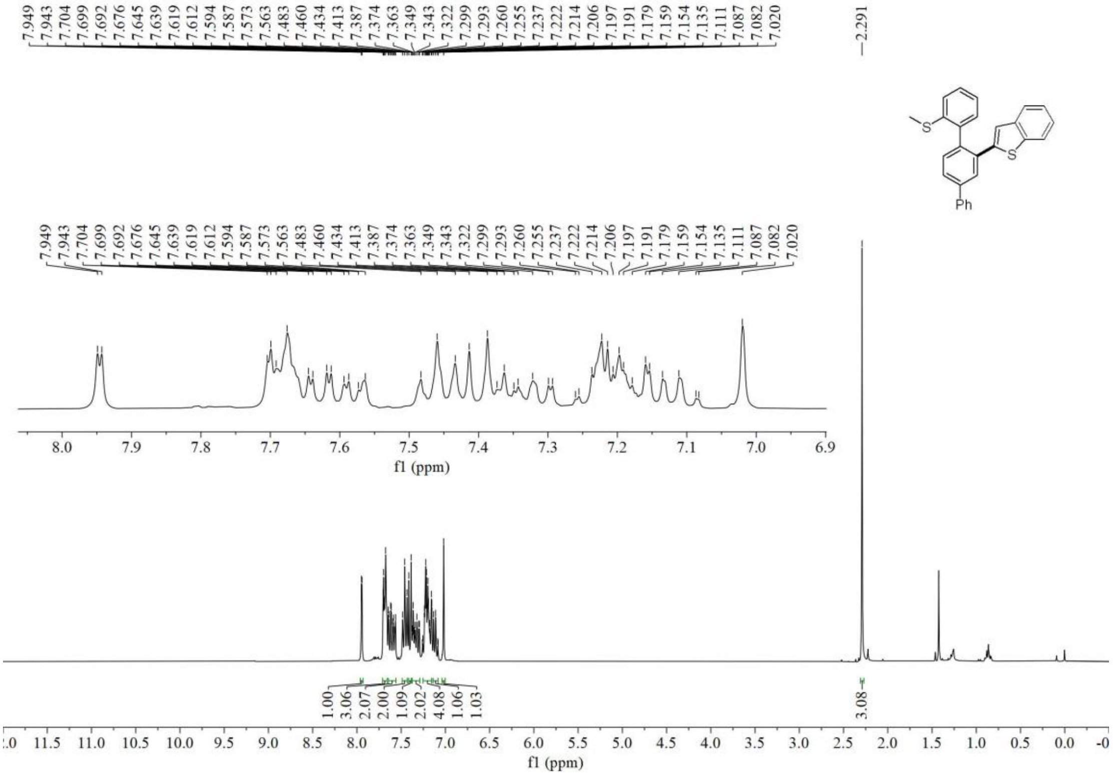

$^{13}C\{^{1}H\}$ NMR spectrum of **4n** in $CDCl_3$ (75 MHz)

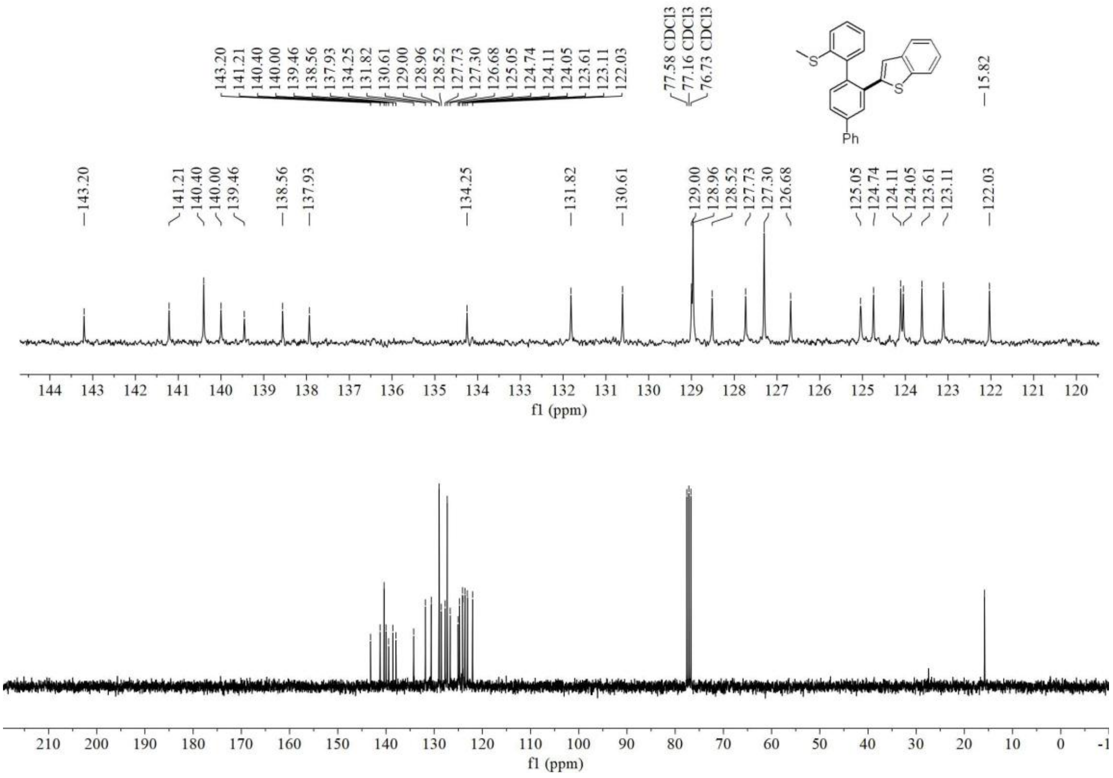

^{1}H NMR spectrum of **4n'** in $CDCl_3$ (300 MHz)

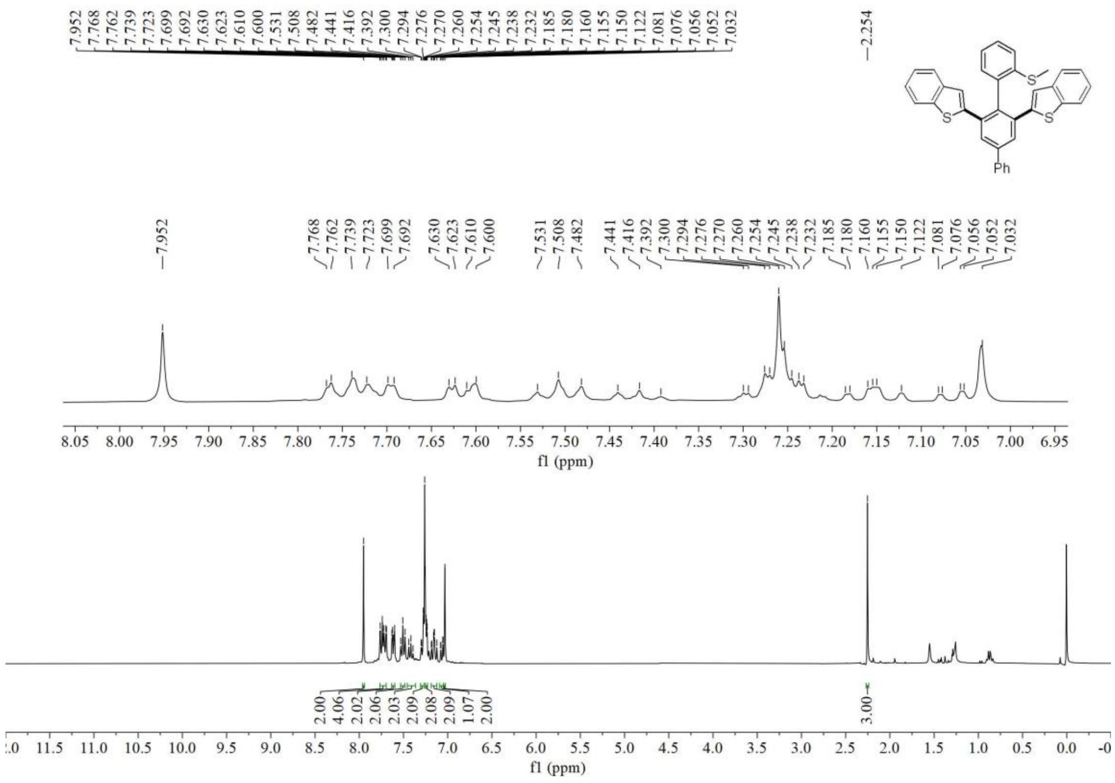

$^{13}C\{^{1}H\}$ NMR spectrum of **4n'** in $CDCl_3$ (75 MHz)

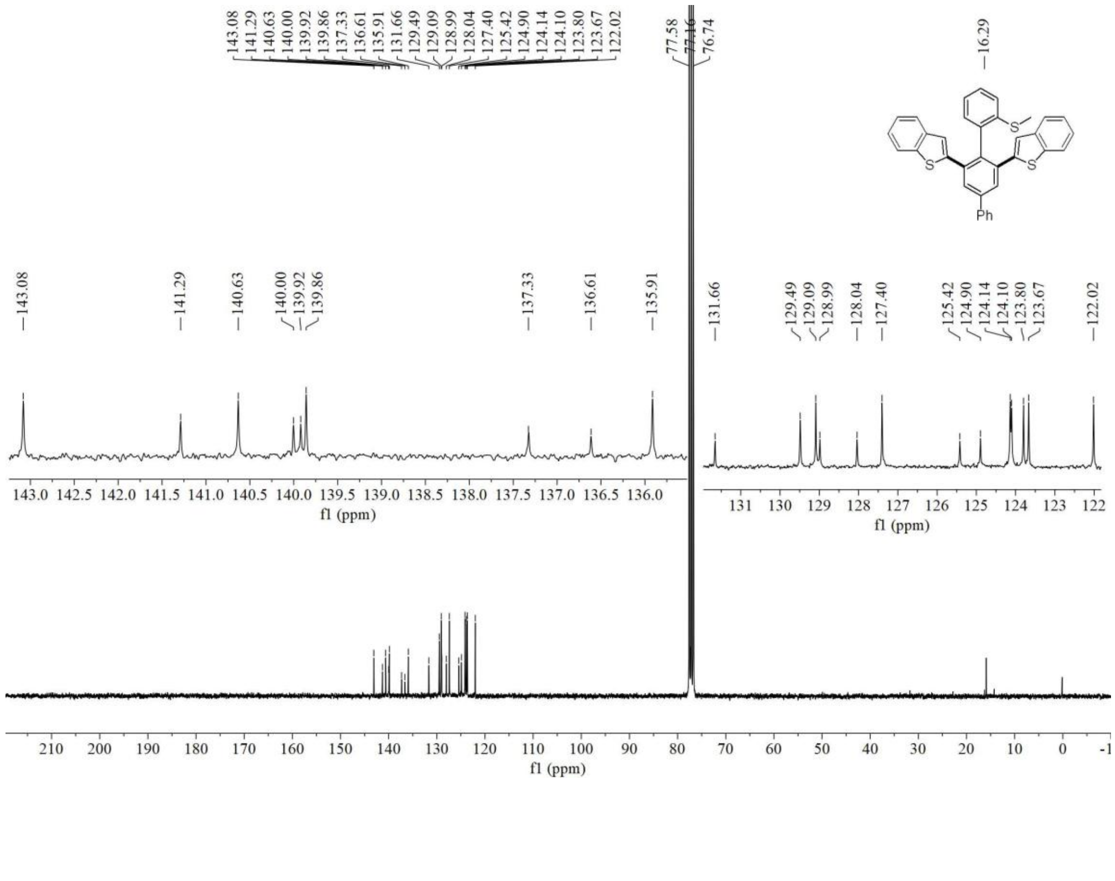

^{1}H NMR spectrum of **4o** in $CDCl_3$ (300 MHz)

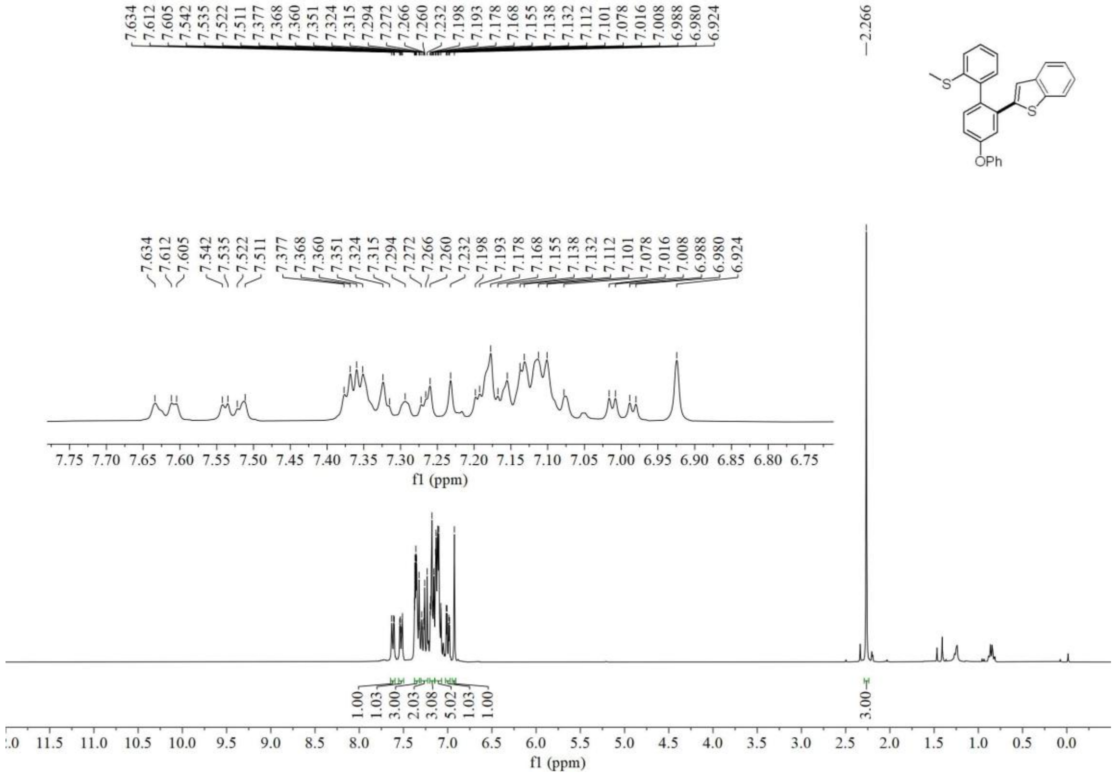

^{13}C{^{1}H} NMR spectrum of **4o** in $CDCl_3$ (75 MHz)

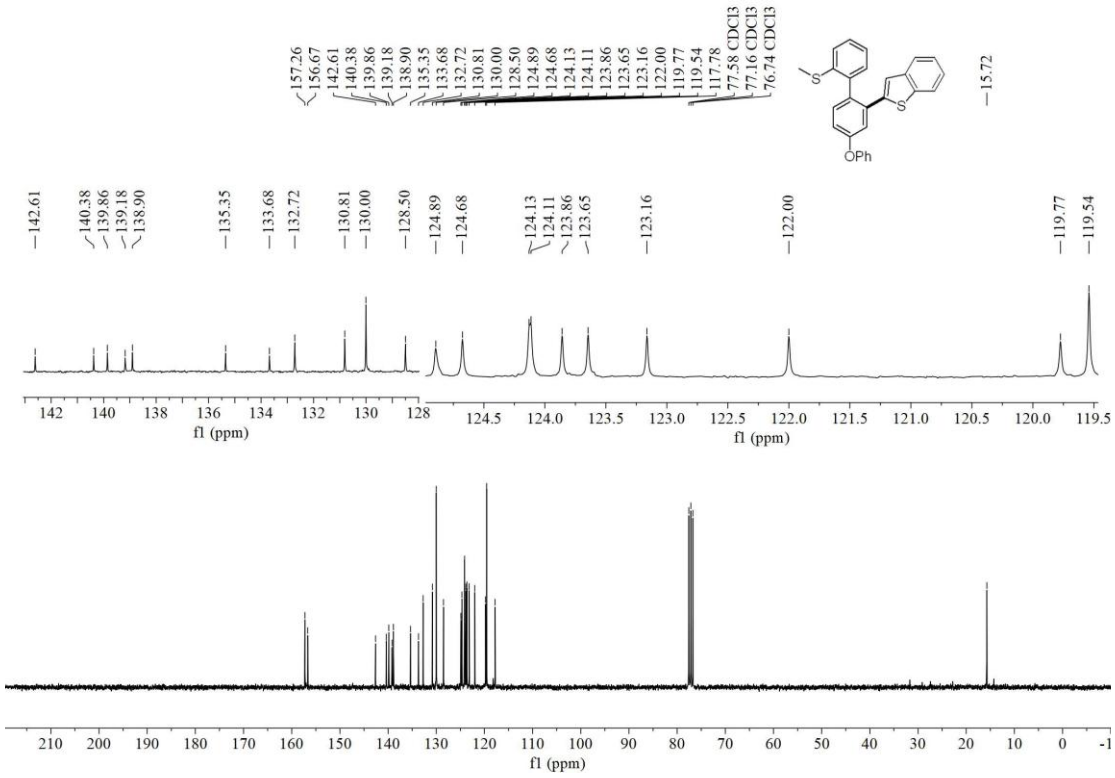

^{1}H NMR spectrum of **4o'** in $CDCl_3$ (300 MHz)

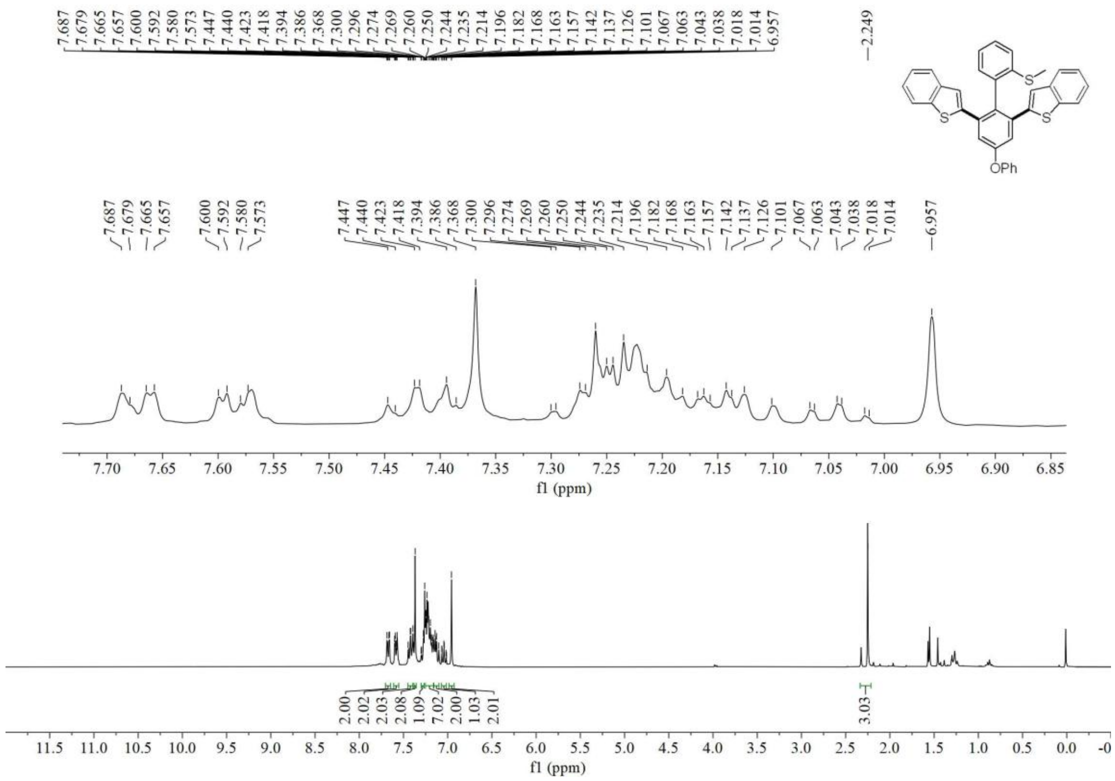

^{13}C{^{1}H} NMR spectrum of **4o'** in $CDCl_3$ (75 MHz)

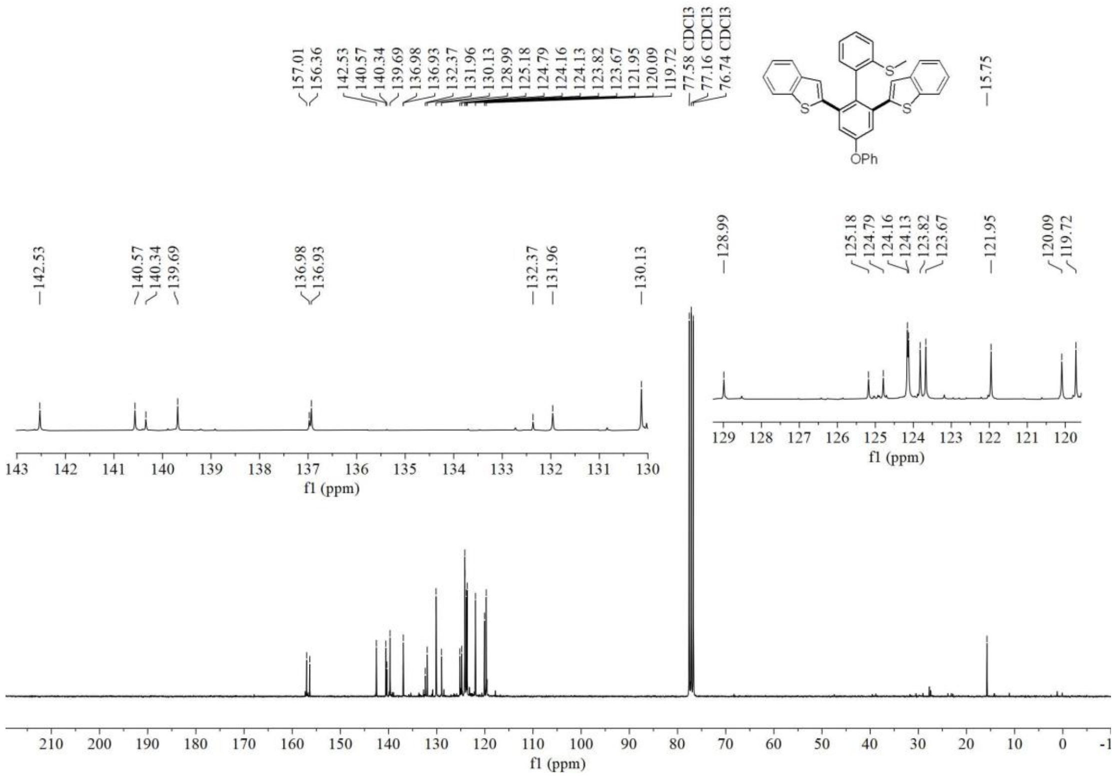

^{1}H NMR spectrum of **4p** in $CDCl_3$ (300 MHz)

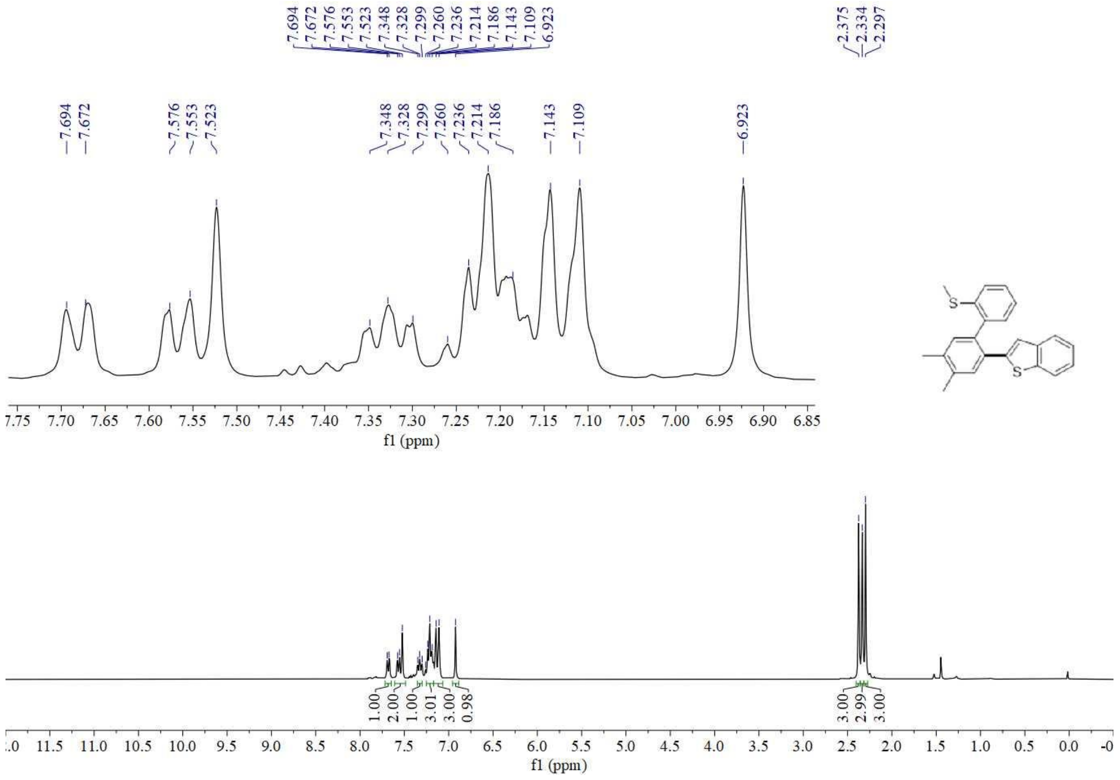

^{13}C{^{1}H} NMR spectrum of **4p** in $CDCl_3$ (75 MHz)

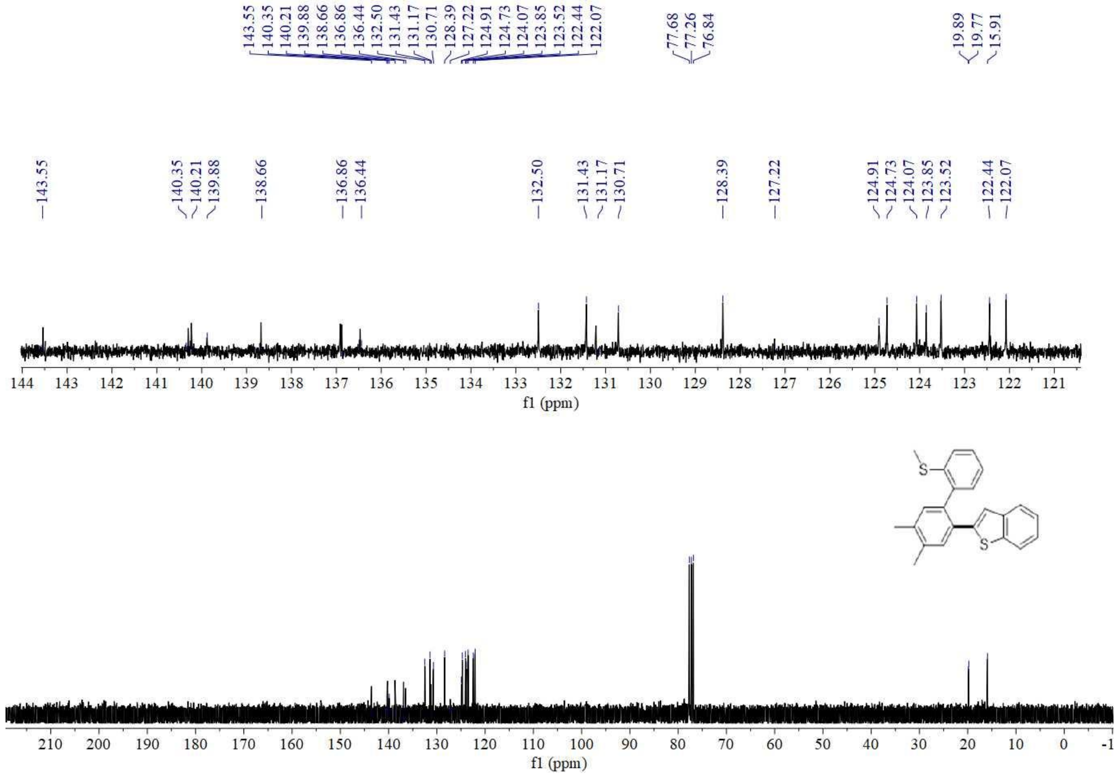

^{1}H NMR spectrum of **4q** in $CDCl_3$ (300 MHz)

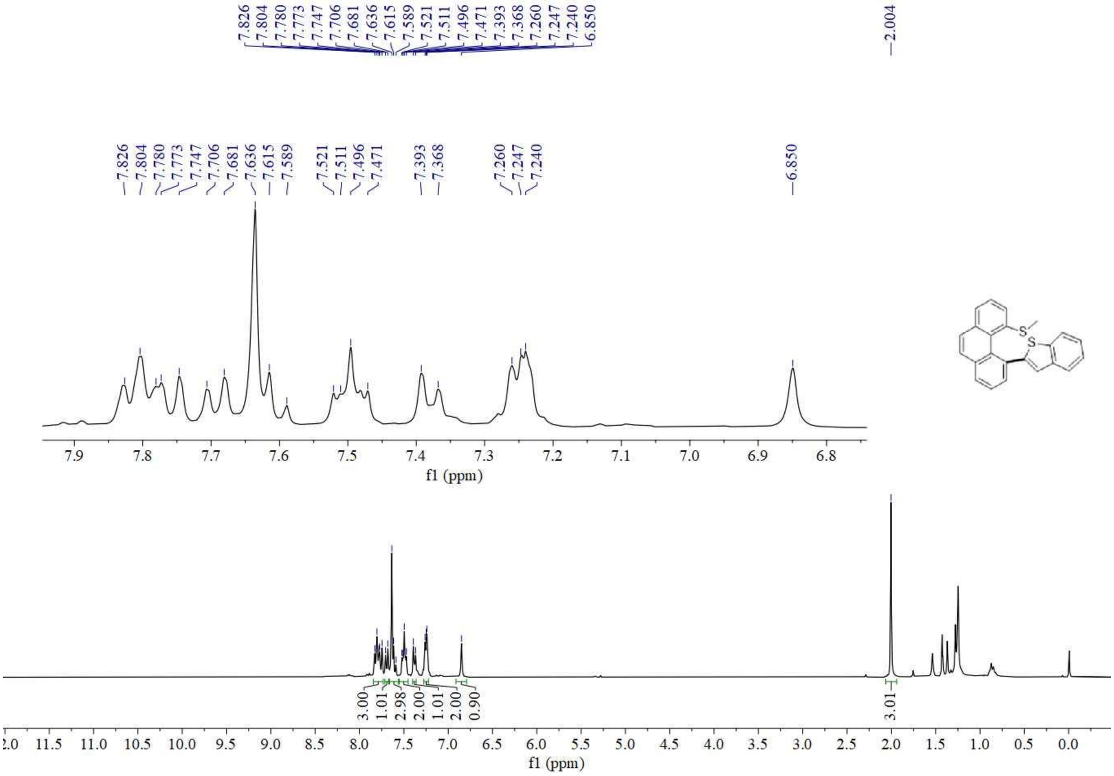

^{13}C{^{1}H} NMR spectrum of **4q** in $CDCl_3$ (75 MHz)

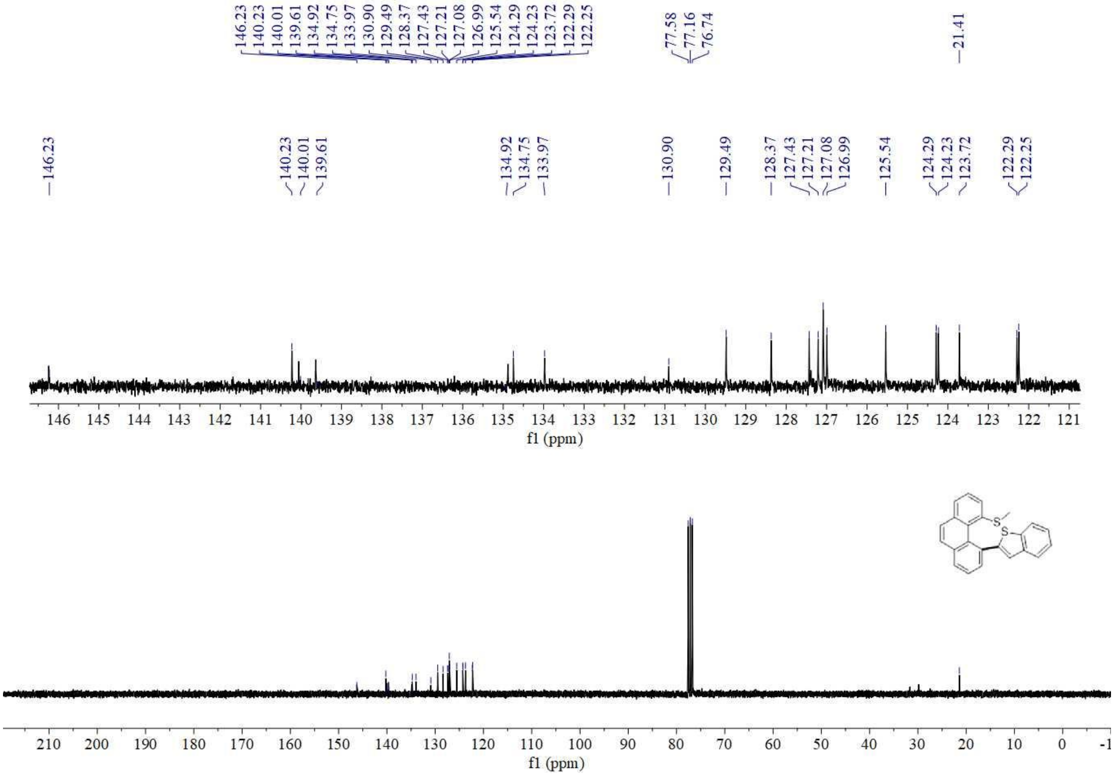

^{1}H NMR spectrum of **4r** in $CDCl_3$ (300 MHz)

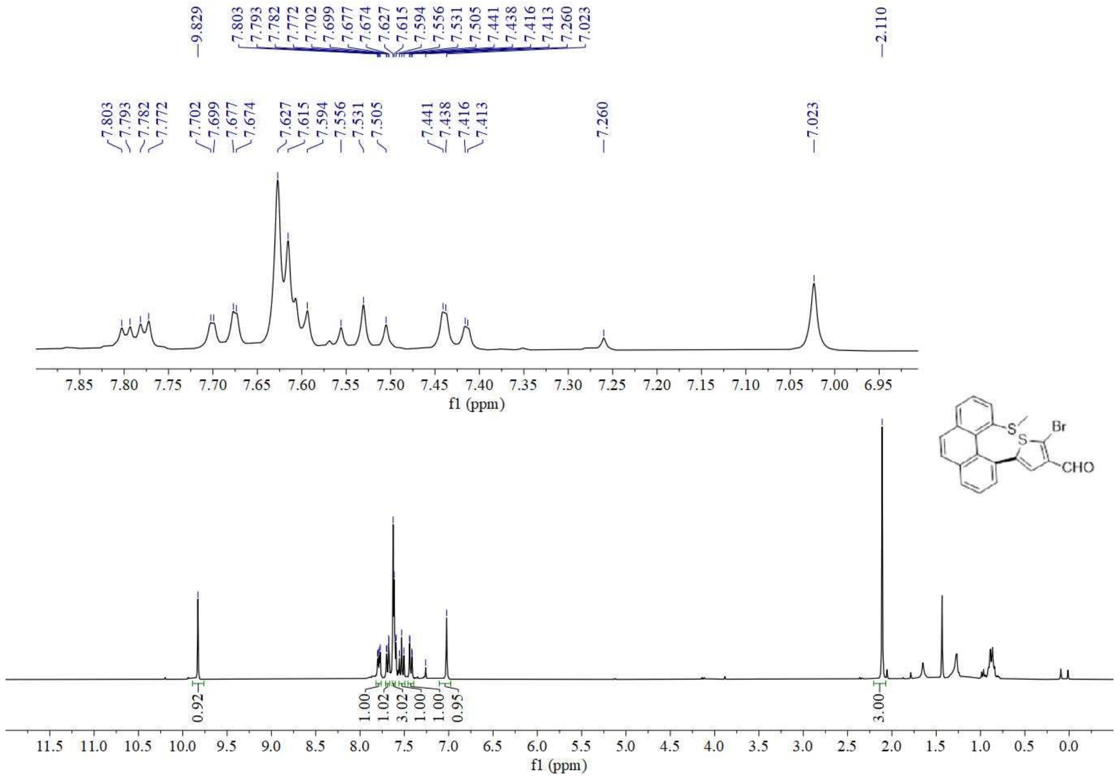

^{13}C{^{1}H} NMR spectrum of **4r** in $CDCl_3$ (75 MHz)

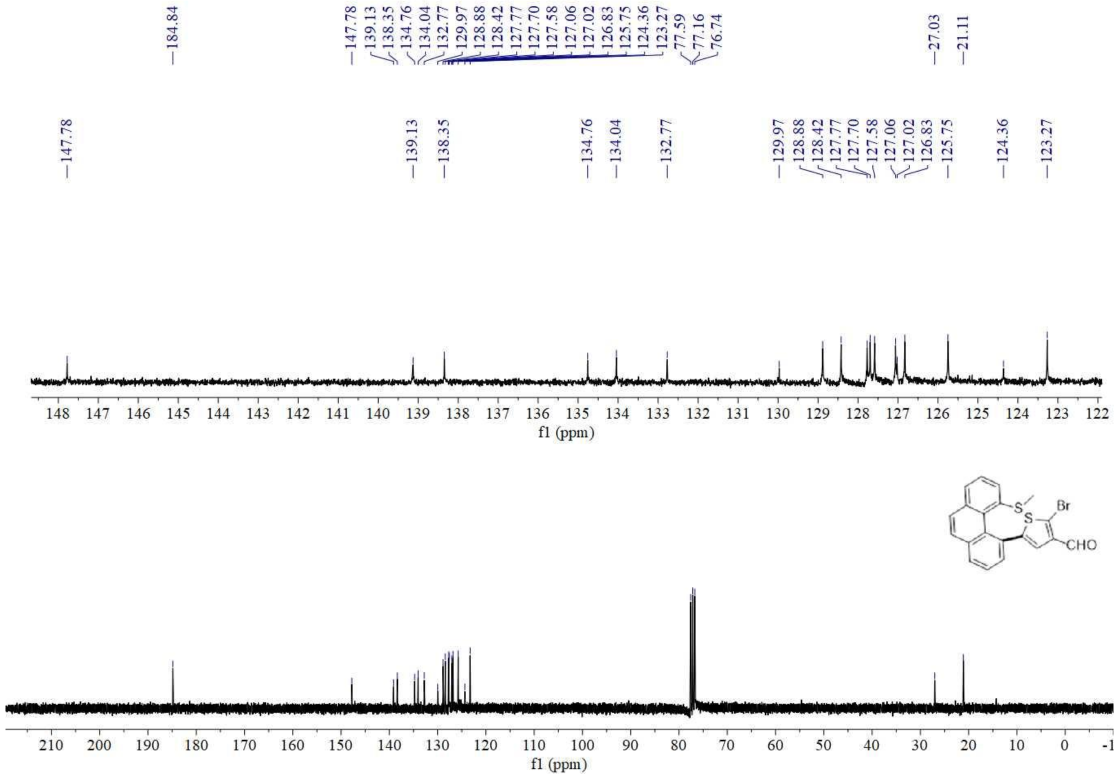

^{1}H NMR spectrum of **4s** in $CDCl_3$ (300 MHz)

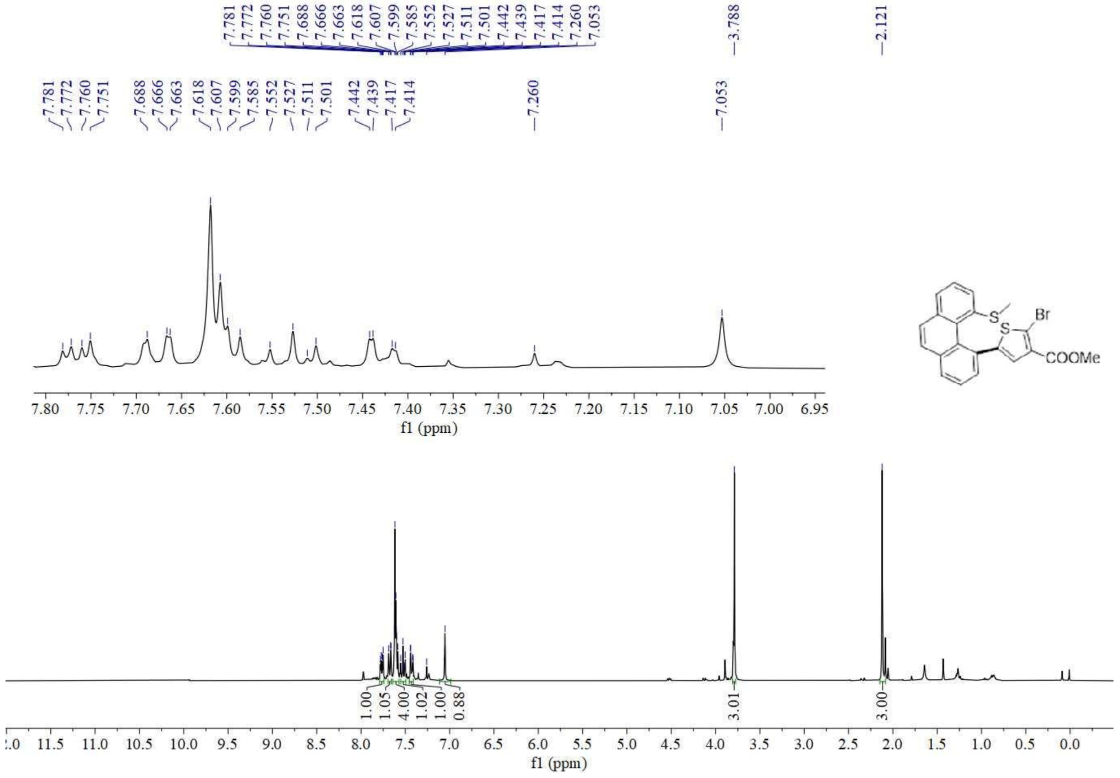

^{13}C{^{1}H} NMR spectrum of **4s** in $CDCl_3$ (75 MHz)

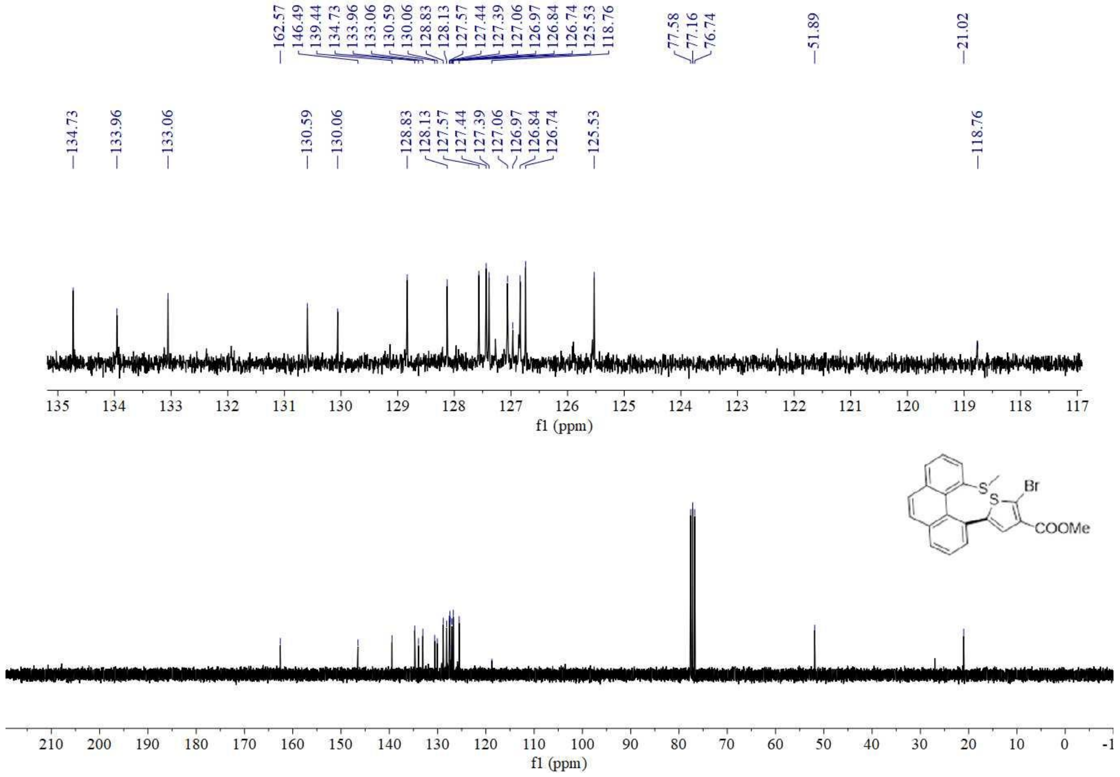

^{1}H NMR spectrum of **6a** in $CDCl_3$ (300 MHz)

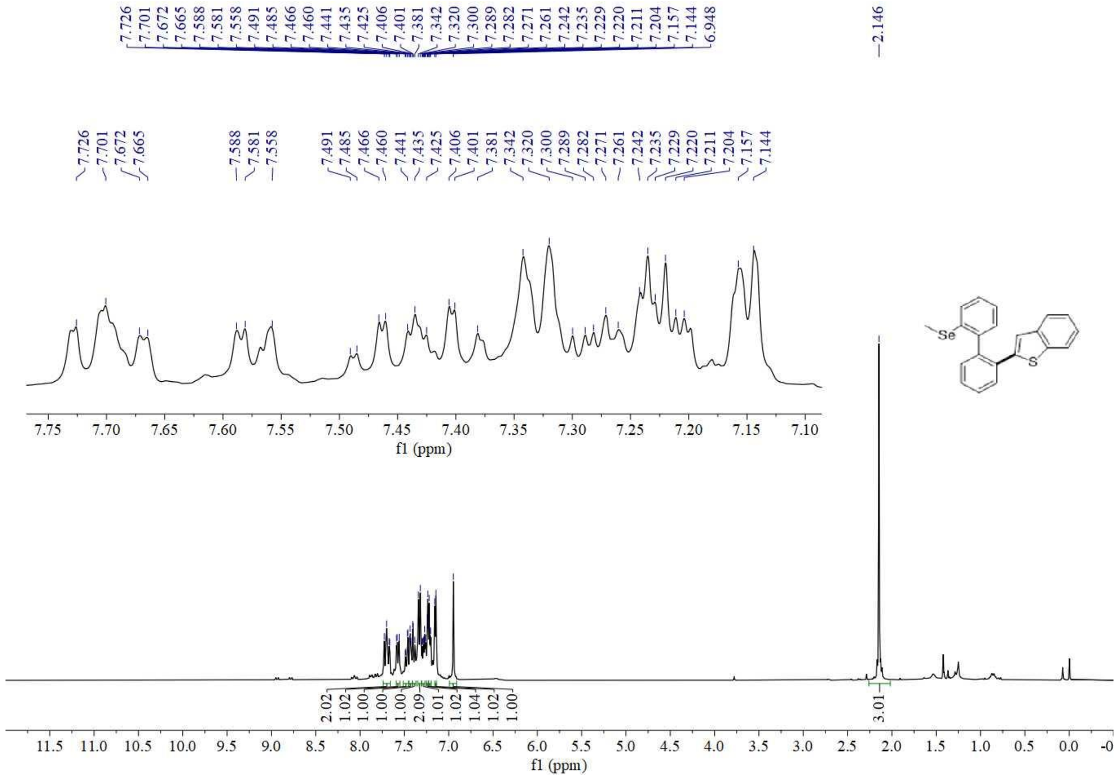

^{13}C{^{1}H} NMR spectrum of **6a** in $CDCl_3$ (75 MHz)

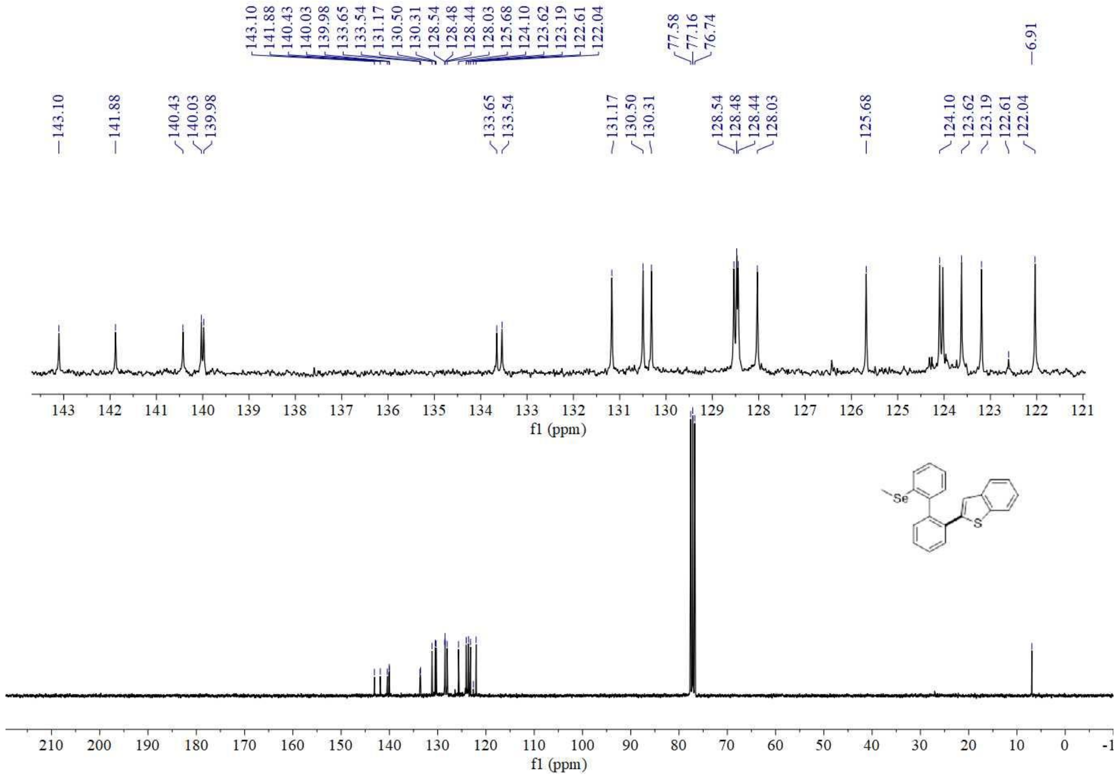

^{1}H NMR spectrum of **6b** in $CDCl_3$ (300 MHz)

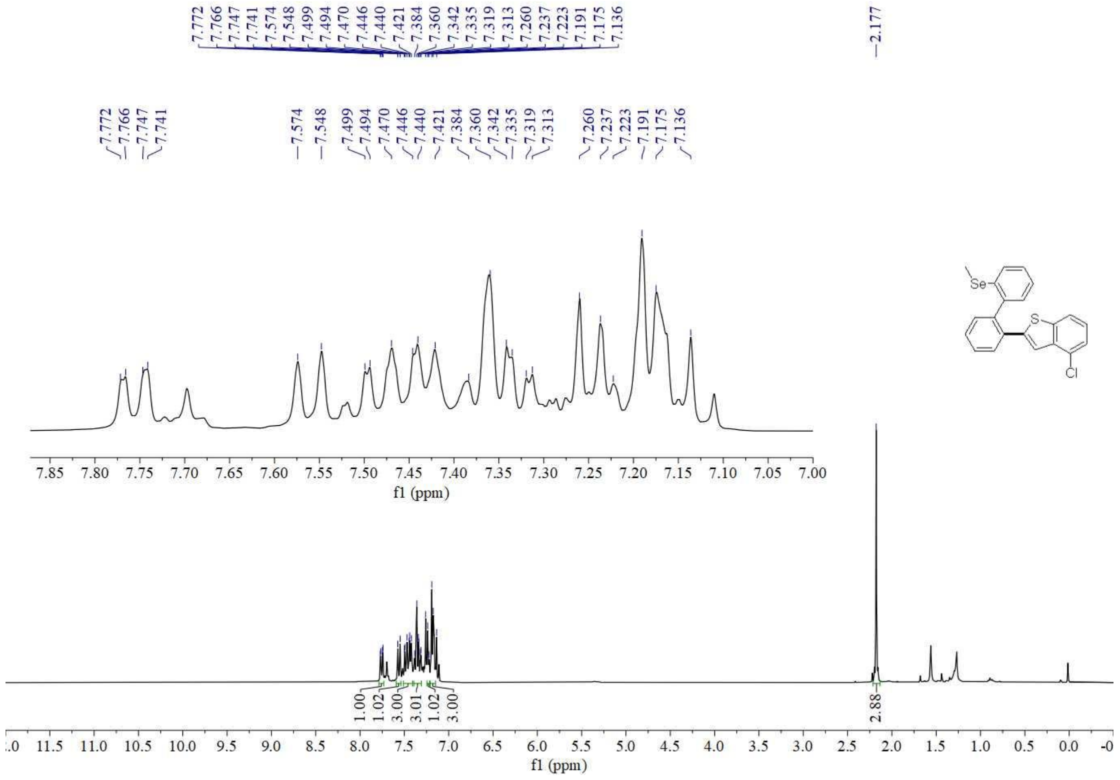

^{13}C{^{1}H} NMR spectrum of **6b** in $CDCl_3$ (75 MHz)

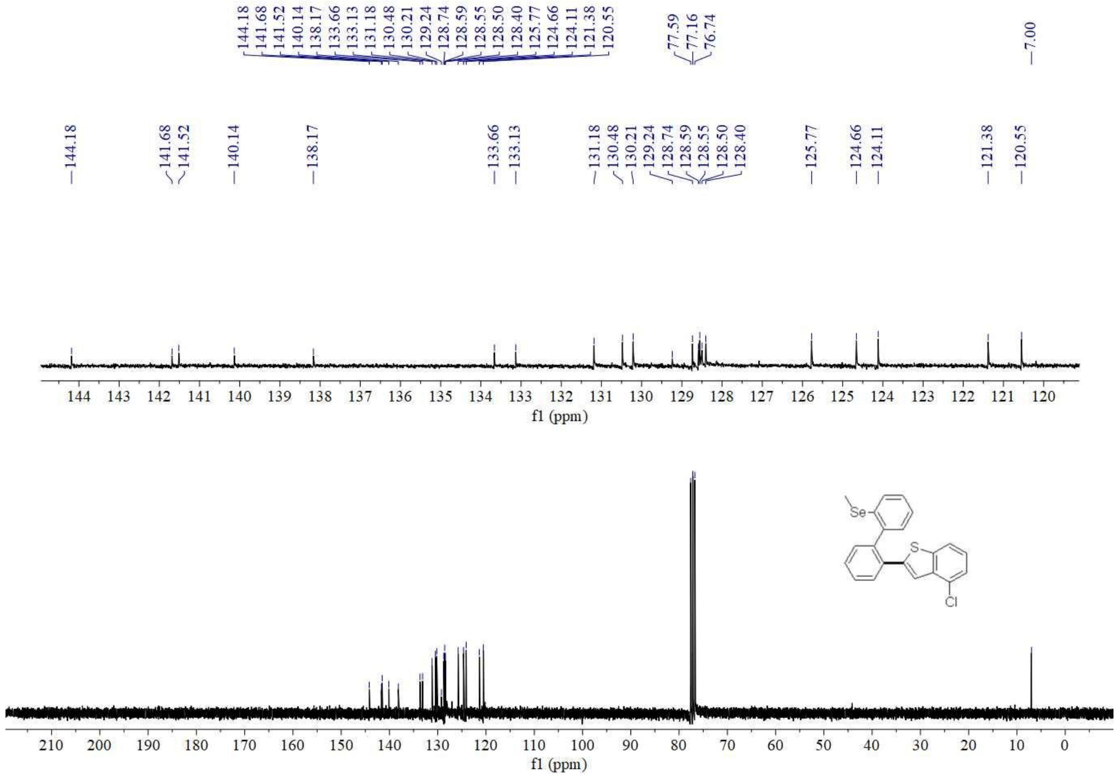

^{1}H NMR spectrum of **6c** in $CDCl_3$ (300 MHz)

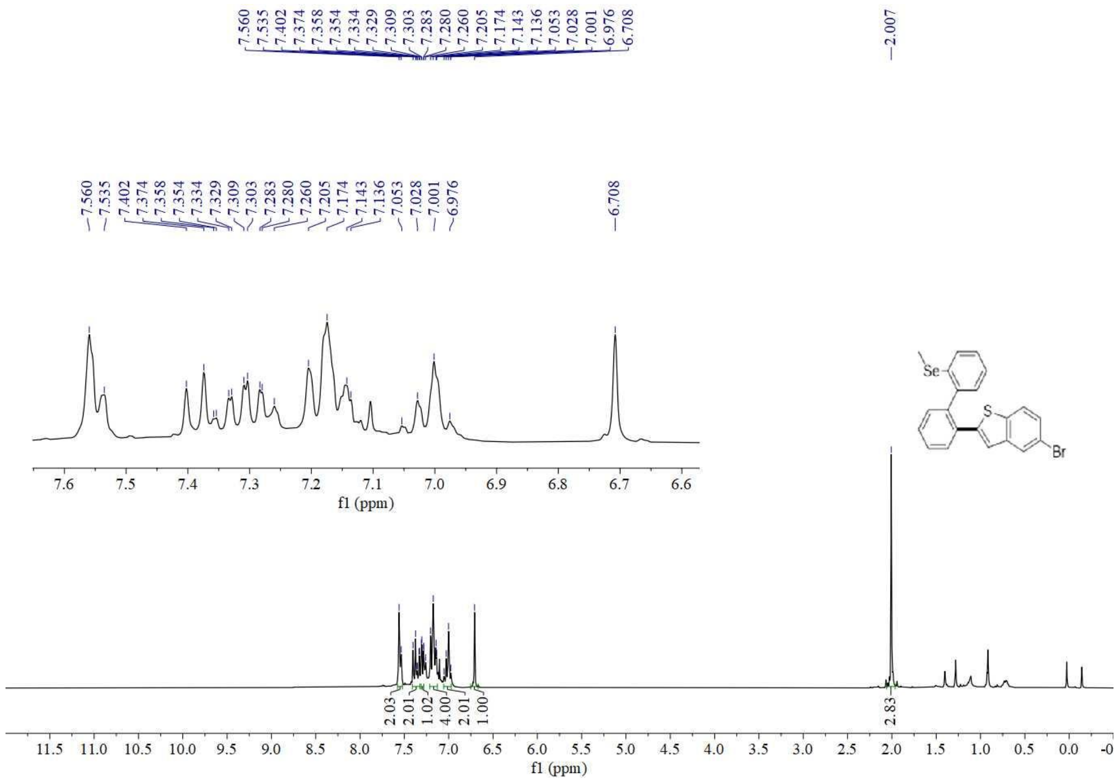

^{13}C{^{1}H} NMR spectrum of **6c** in $CDCl_3$ (75 MHz)

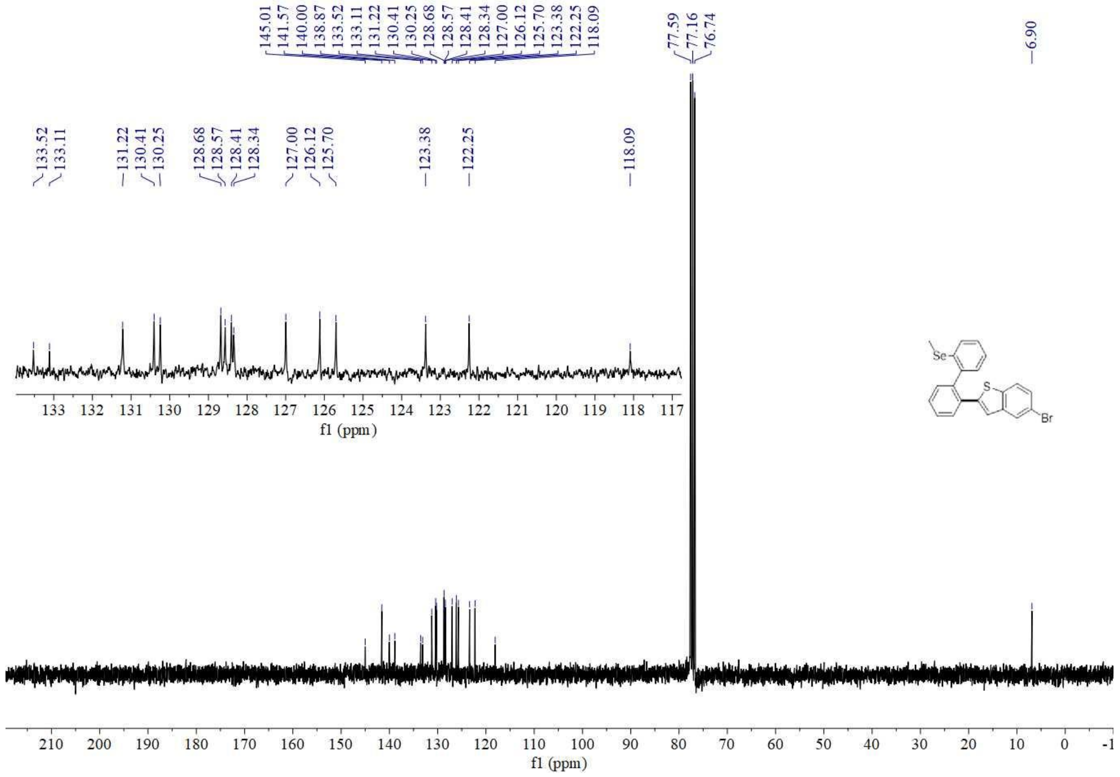

^{1}H NMR spectrum of **6d** in DMSO-d_6 (300 MHz)

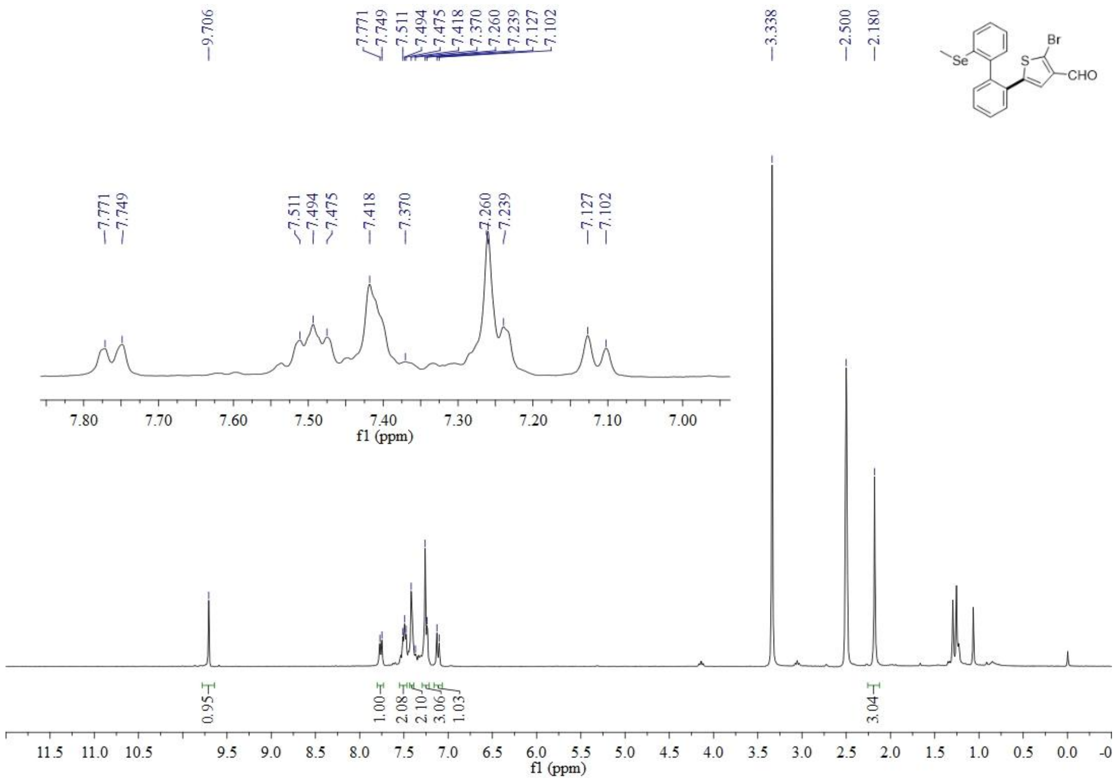

^{13}C{^{1}H} NMR spectrum of **6d** in DMSO-d_6 (75 MHz)

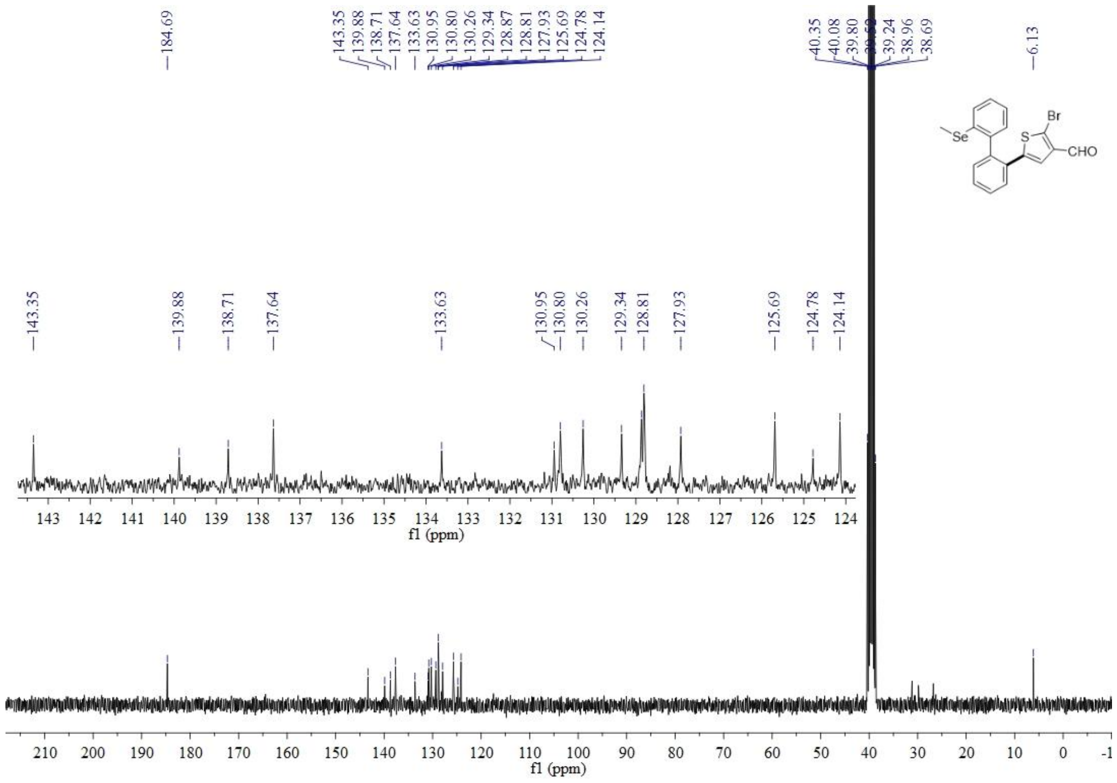

^{1}H NMR spectrum of **6e** in $CDCl_3$ (300 MHz)

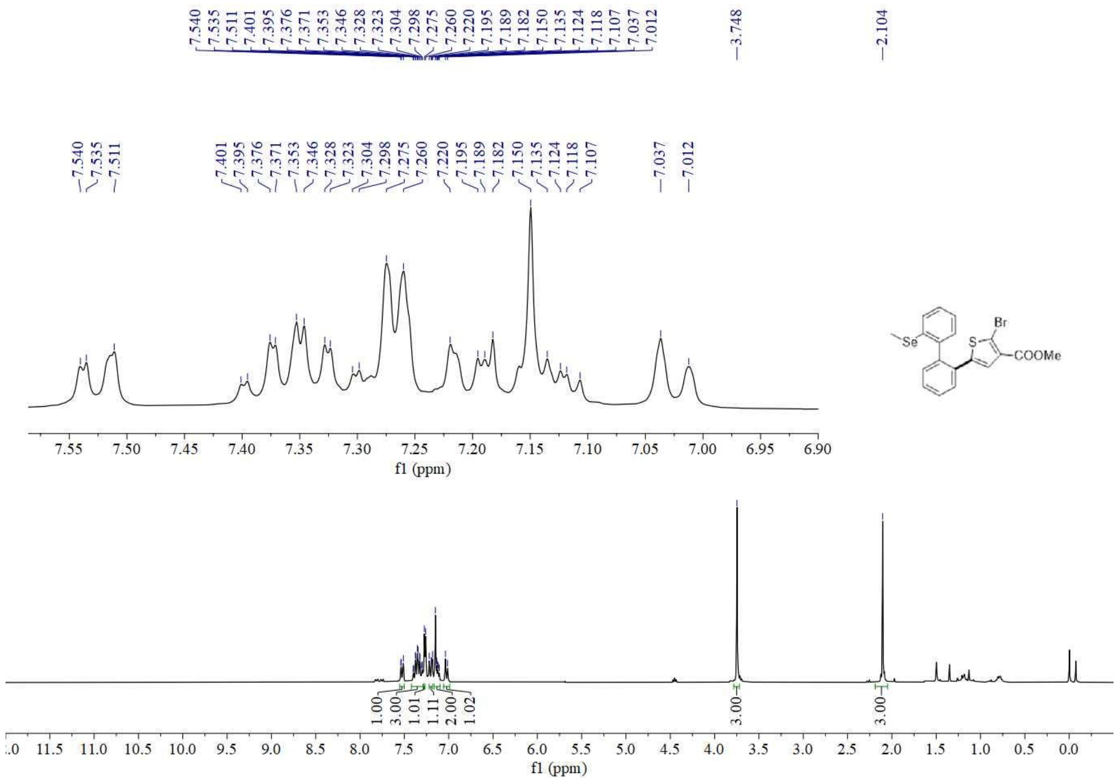

$^{13}C\{^{1}H\}$ NMR spectrum of **6e** in $CDCl_3$ (75 MHz)

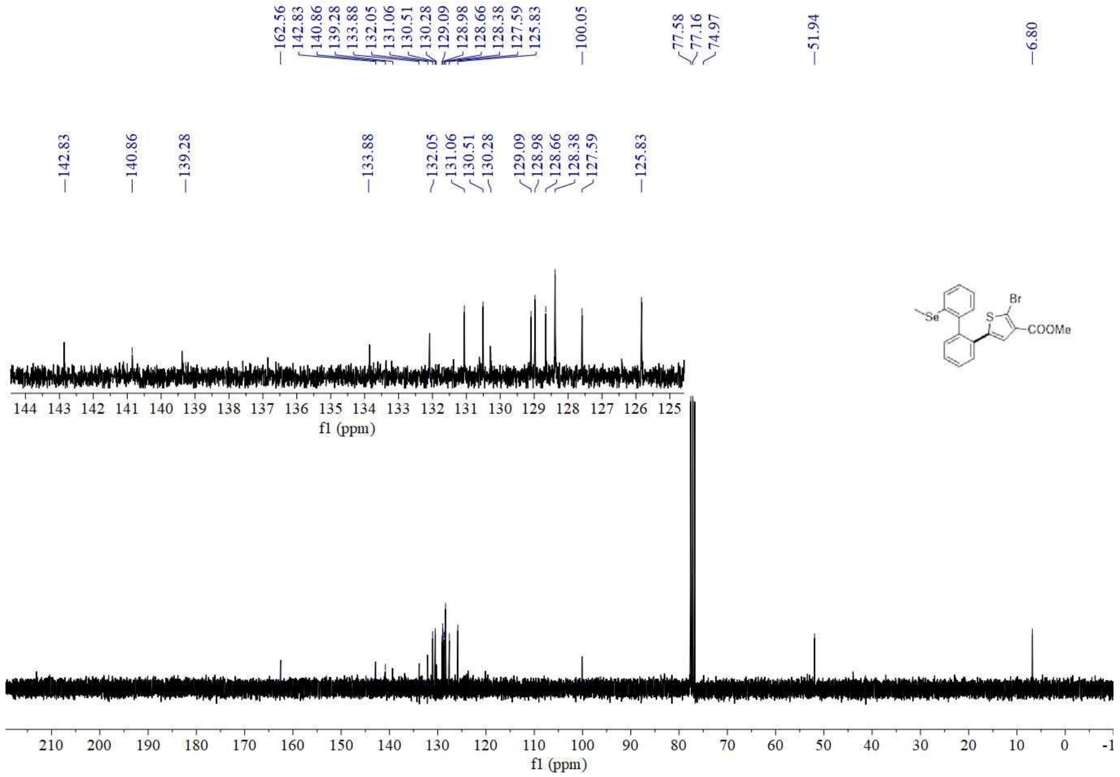

^{1}H NMR spectrum of **6f** in $CDCl_3$ (300 MHz)

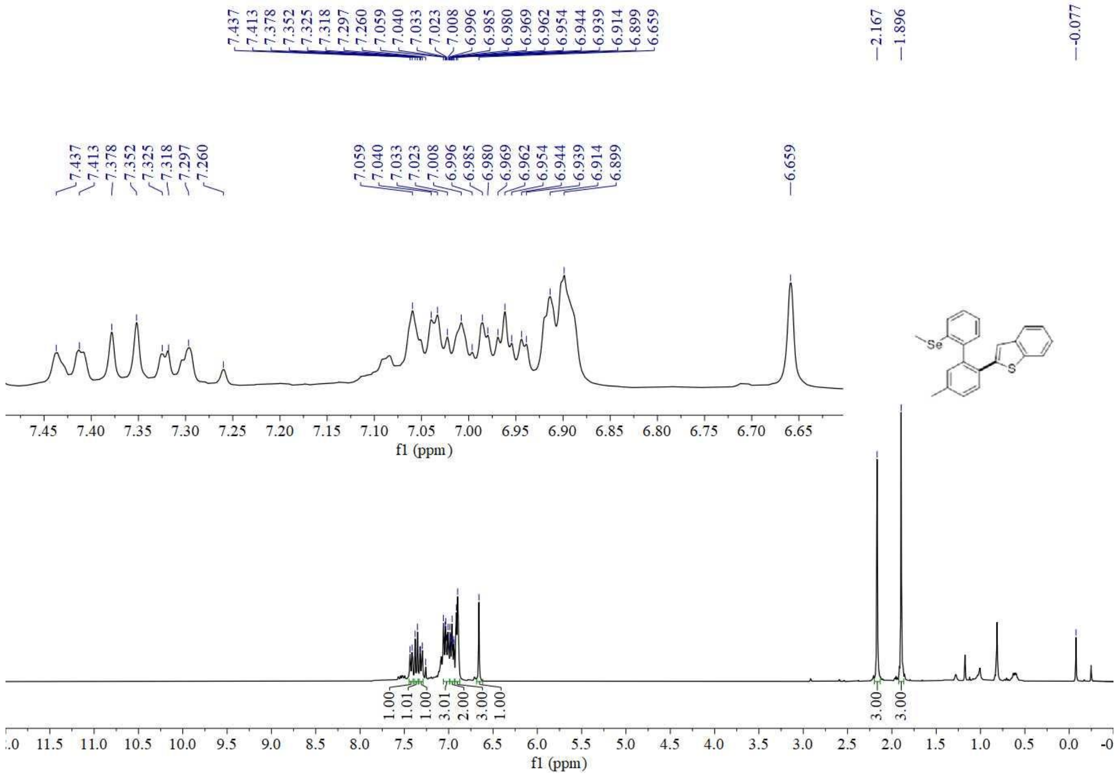

$^{13}C\{^{1}H\}$ NMR spectrum of **6f** in $CDCl_3$ (75 MHz)

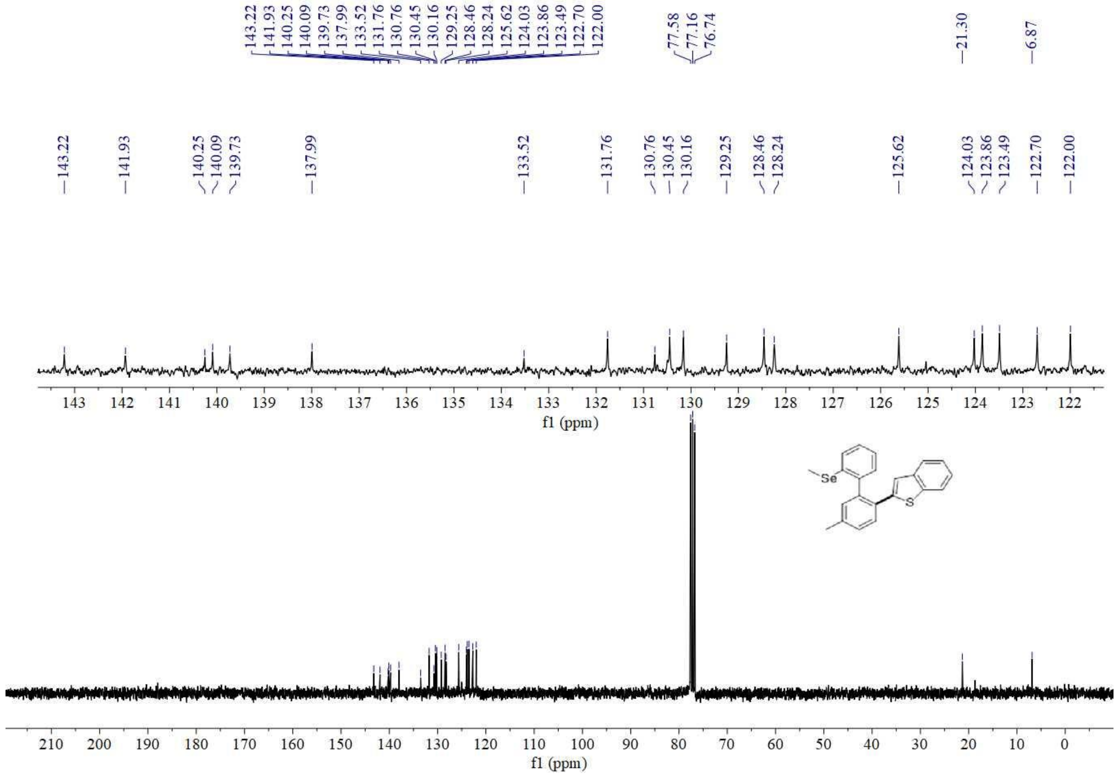

^{1}H NMR spectrum of **6g** in $CDCl_3$ (300 MHz)

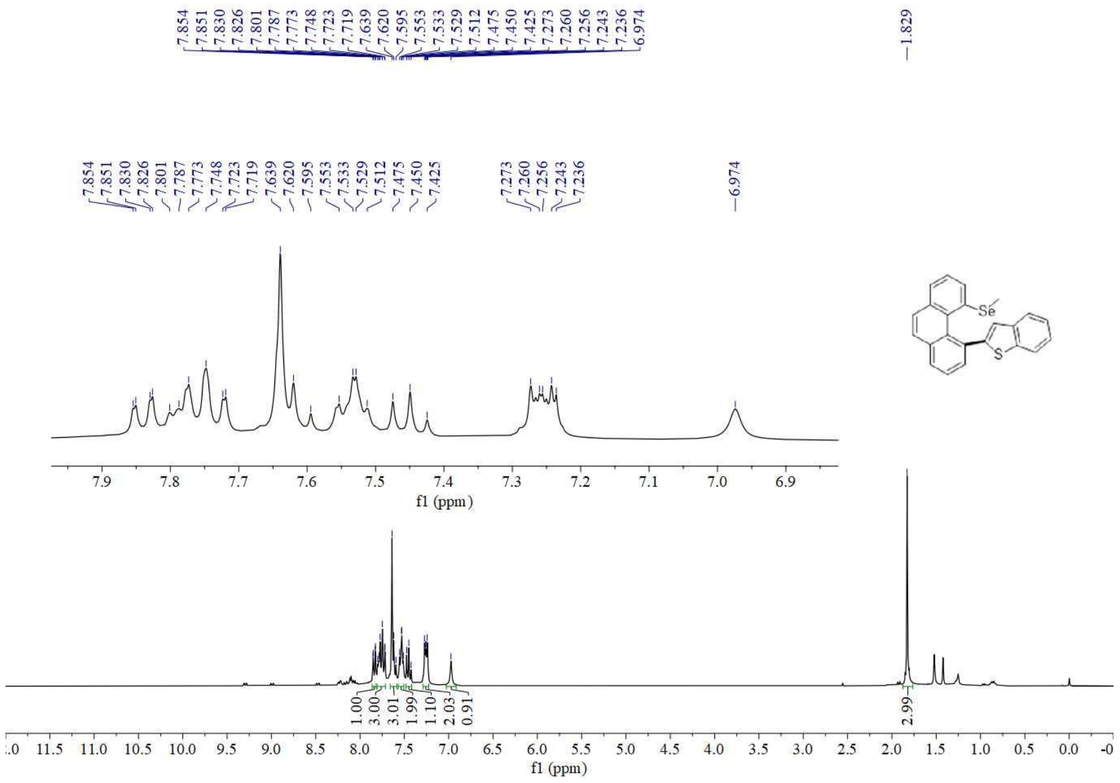

$^{13}C\{^{1}H\}$ NMR spectrum of **6g** in $CDCl_3$ (75 MHz)

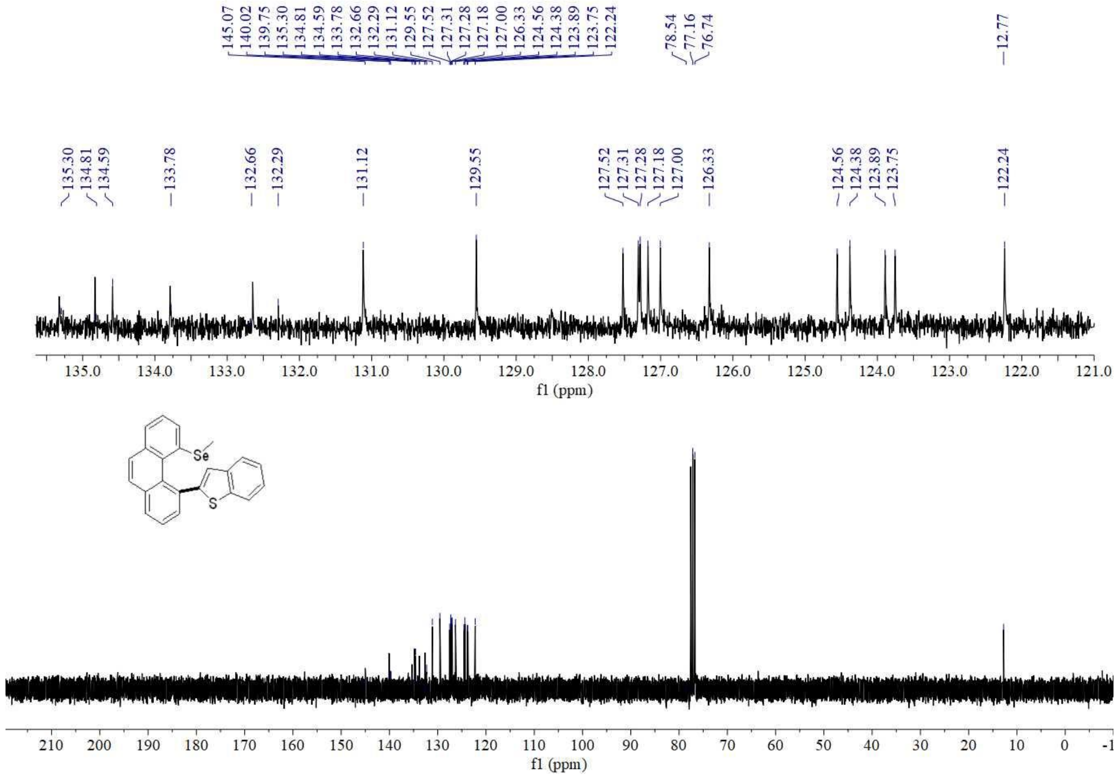

^{1}H NMR spectrum of **7a** in $CDCl_3$ (300 MHz)

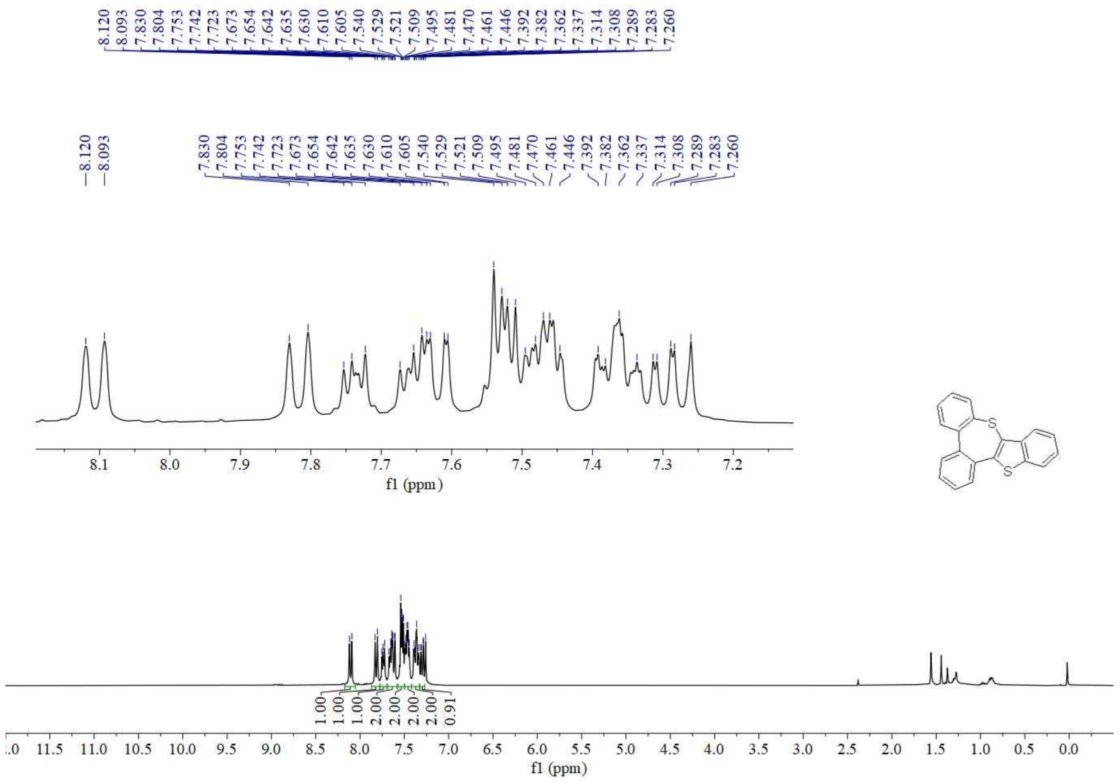

^{13}C{^{1}H} NMR spectrum of **7a** in $CDCl_3$ (75 MHz)

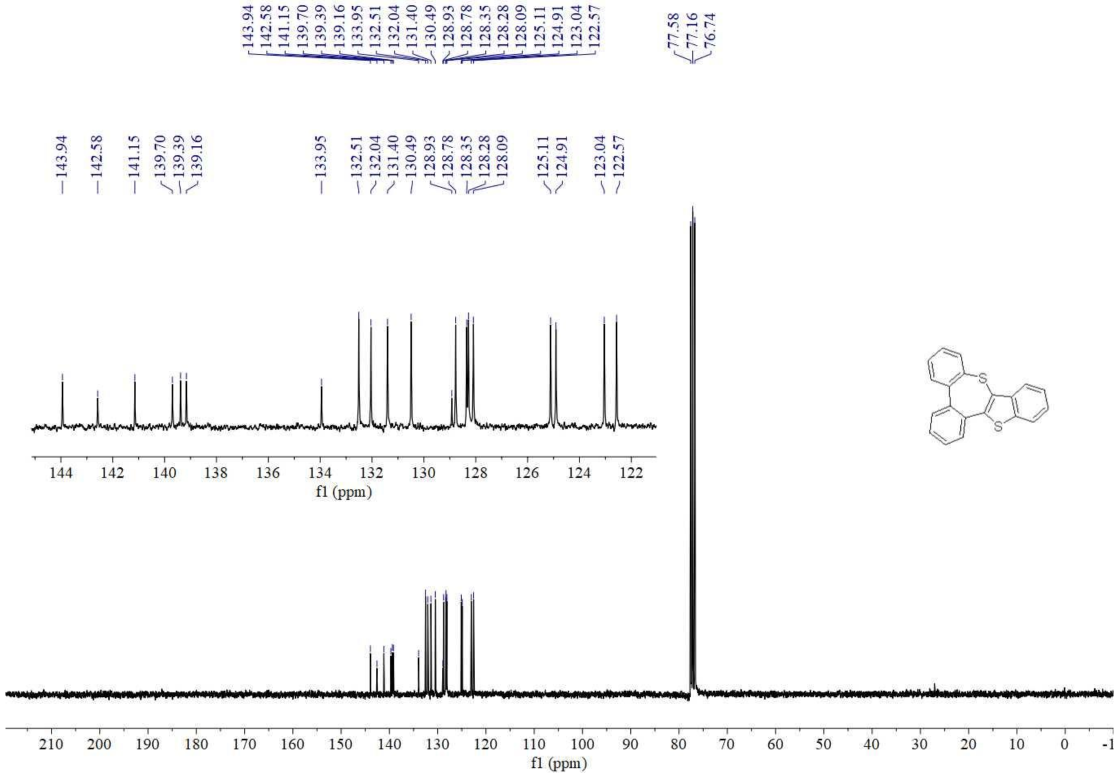

^{1}H NMR spectrum of **7b** in $CDCl_3$ (300 MHz)

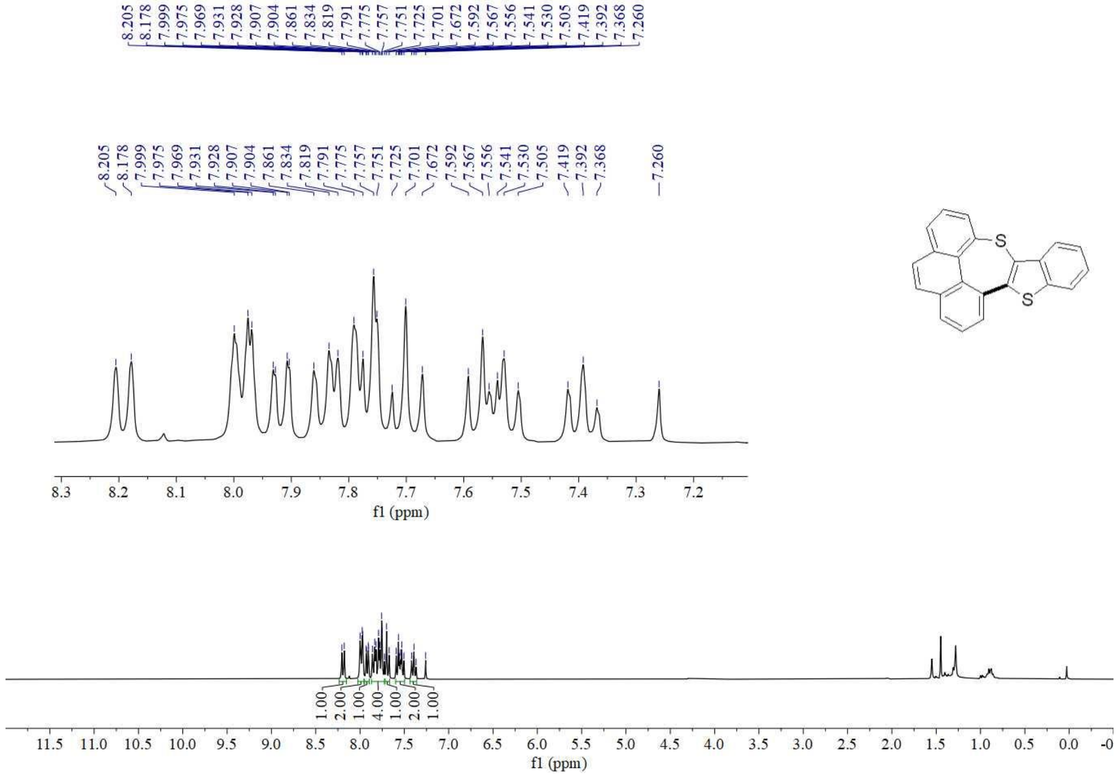

^{13}C{^{1}H} NMR spectrum of **7b** in $CDCl_3$ (75 MHz)

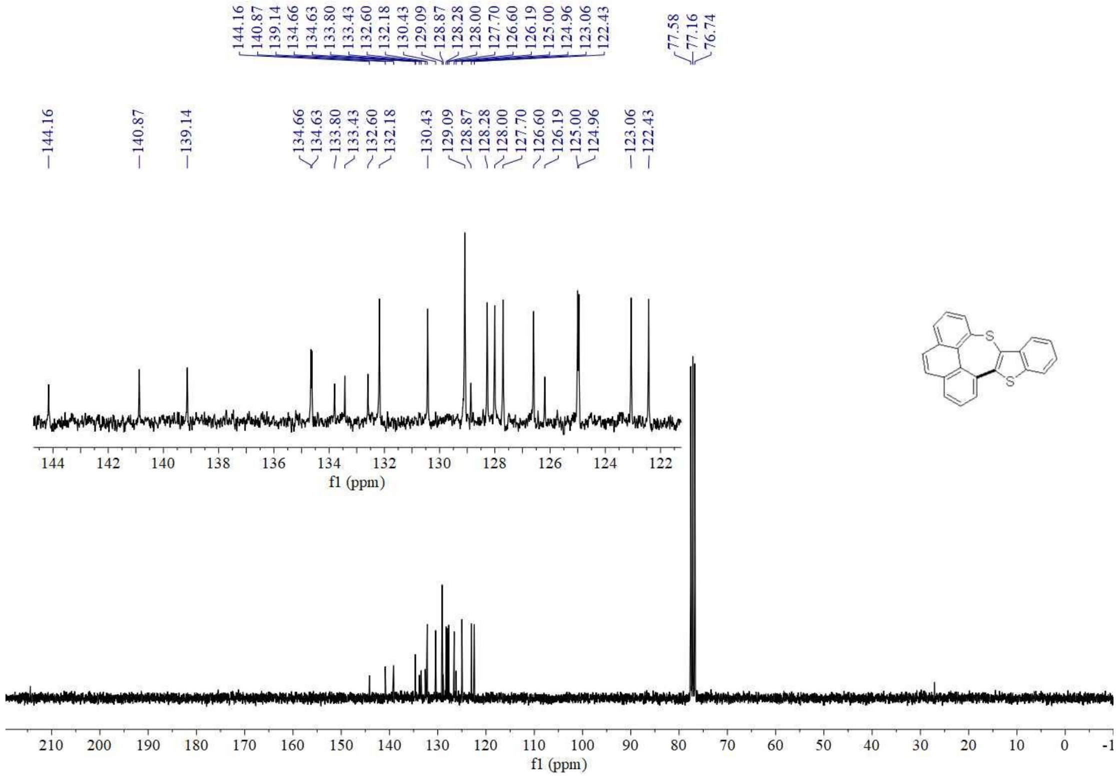

^{1}H NMR spectrum of **8a** in $CDCl_3$ (300 MHz)

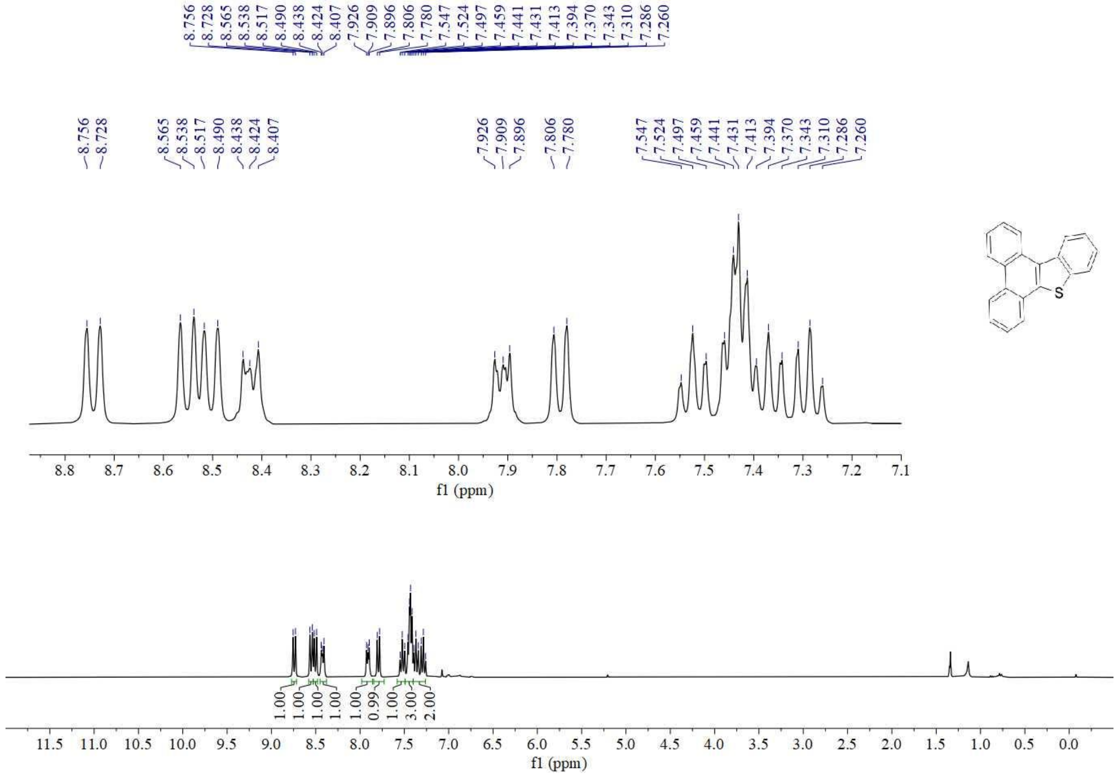

^{13}C{^{1}H} NMR spectrum of **8a** in $CDCl_3$ (75 MHz)

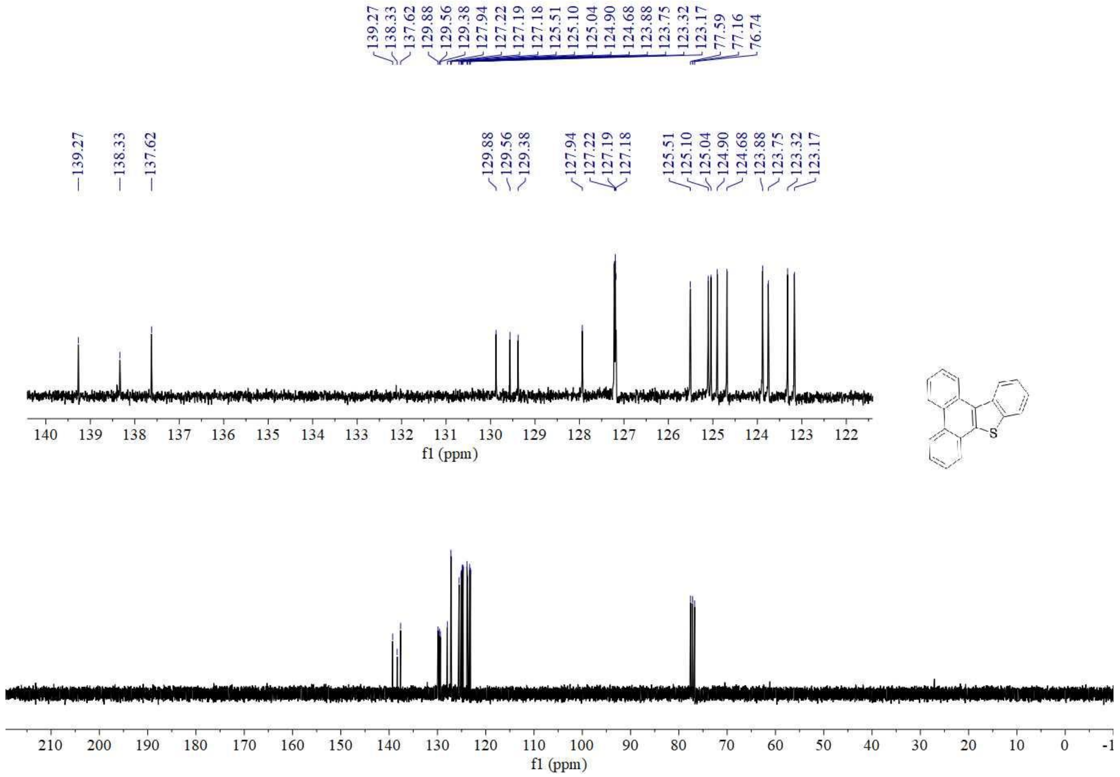

^{1}H NMR spectrum of **8b** in $CDCl_3$ (300 MHz)

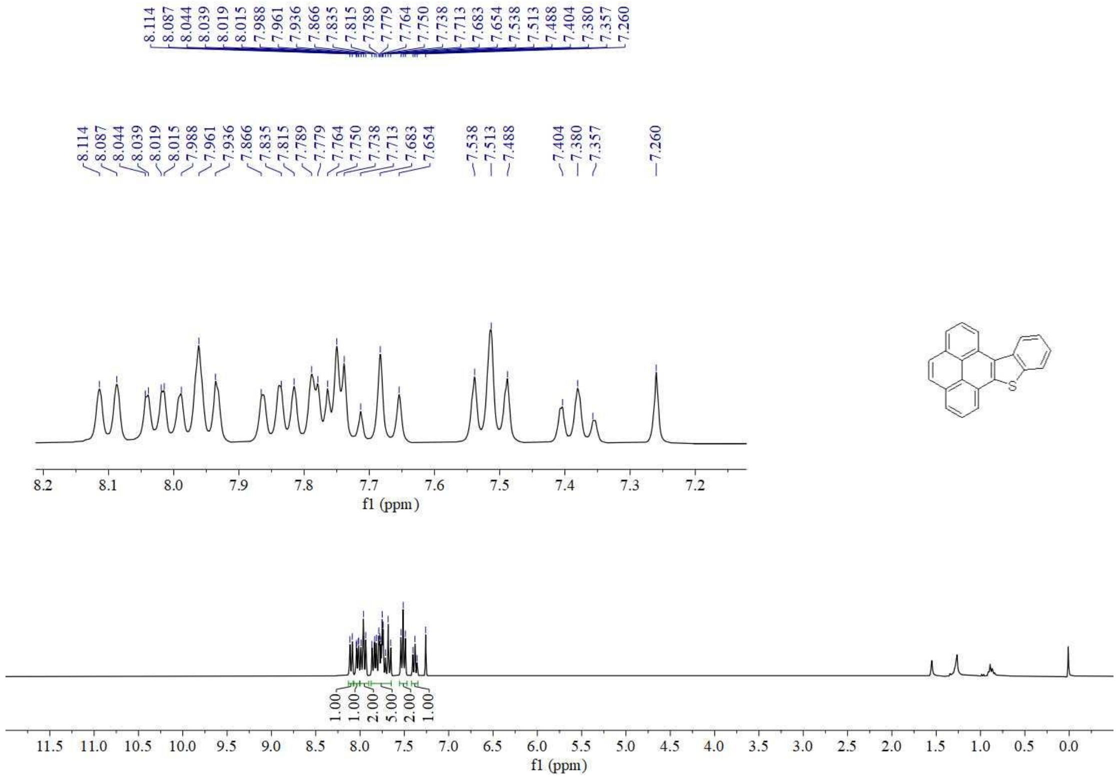

^{13}C{^{1}H} NMR spectrum of **8b** in $CDCl_3$ (75 MHz)

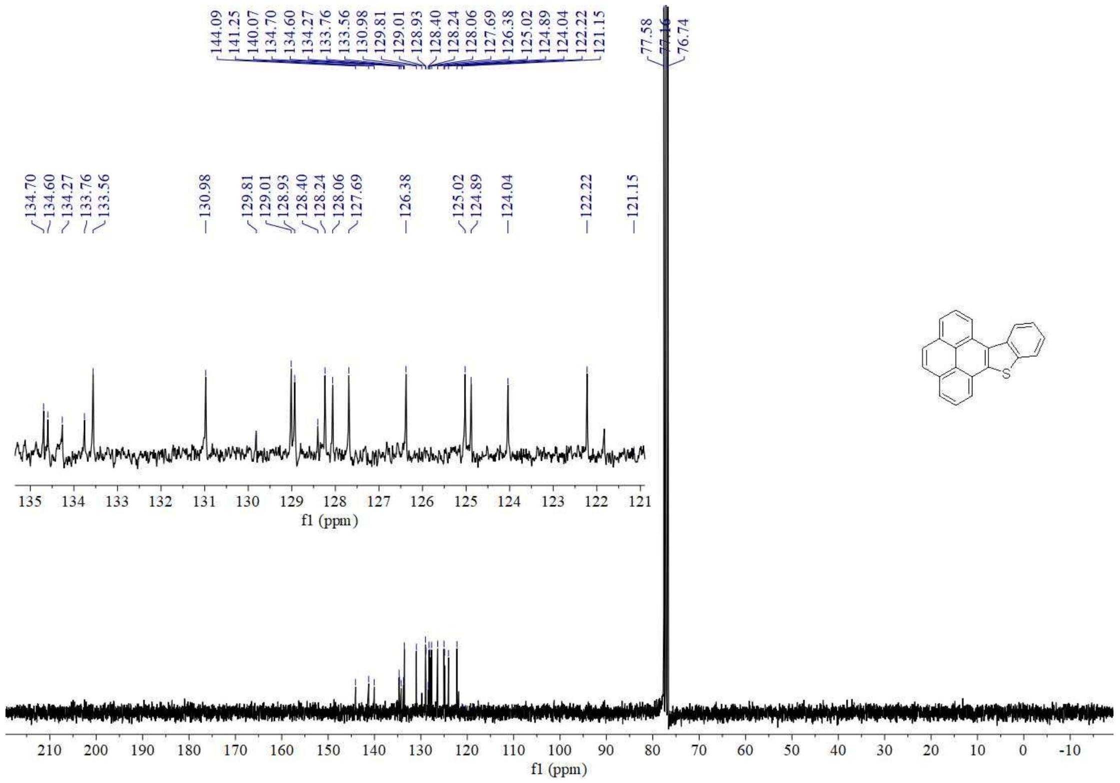

^{1}H NMR spectrum of **9** in $CDCl_3$ (300 MHz)

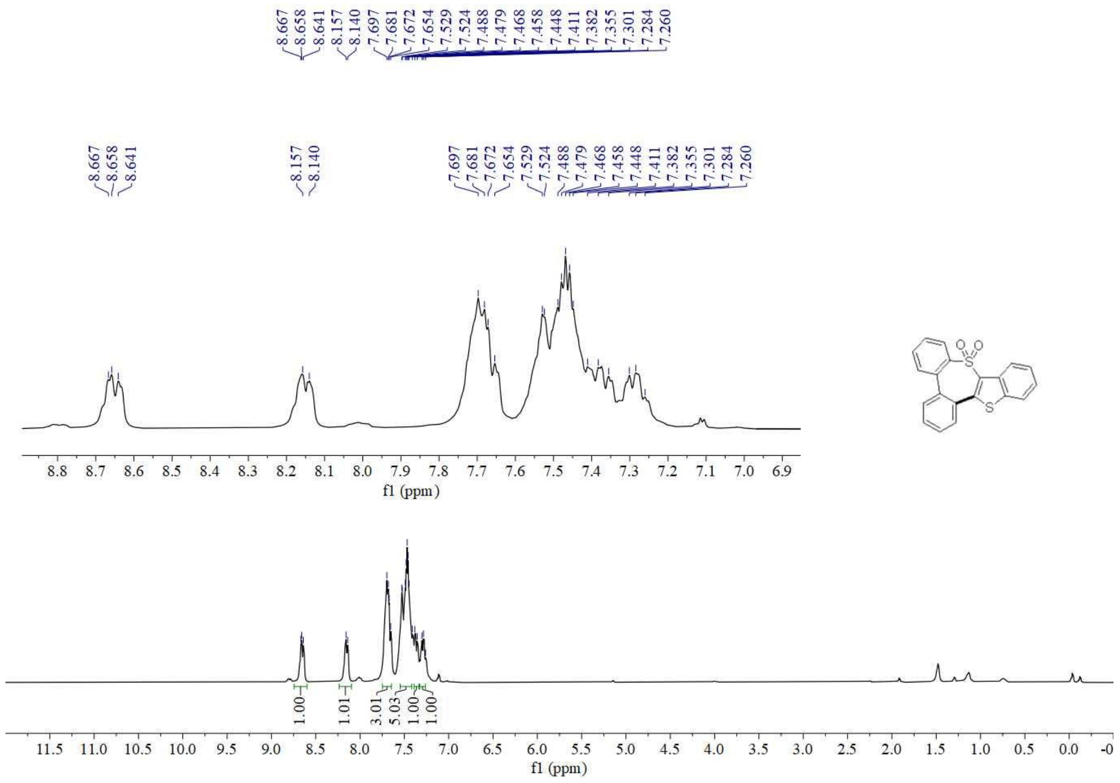

$^{13}C\{^{1}H\}$ NMR spectrum of **9** in $CDCl_3$ (75 MHz)

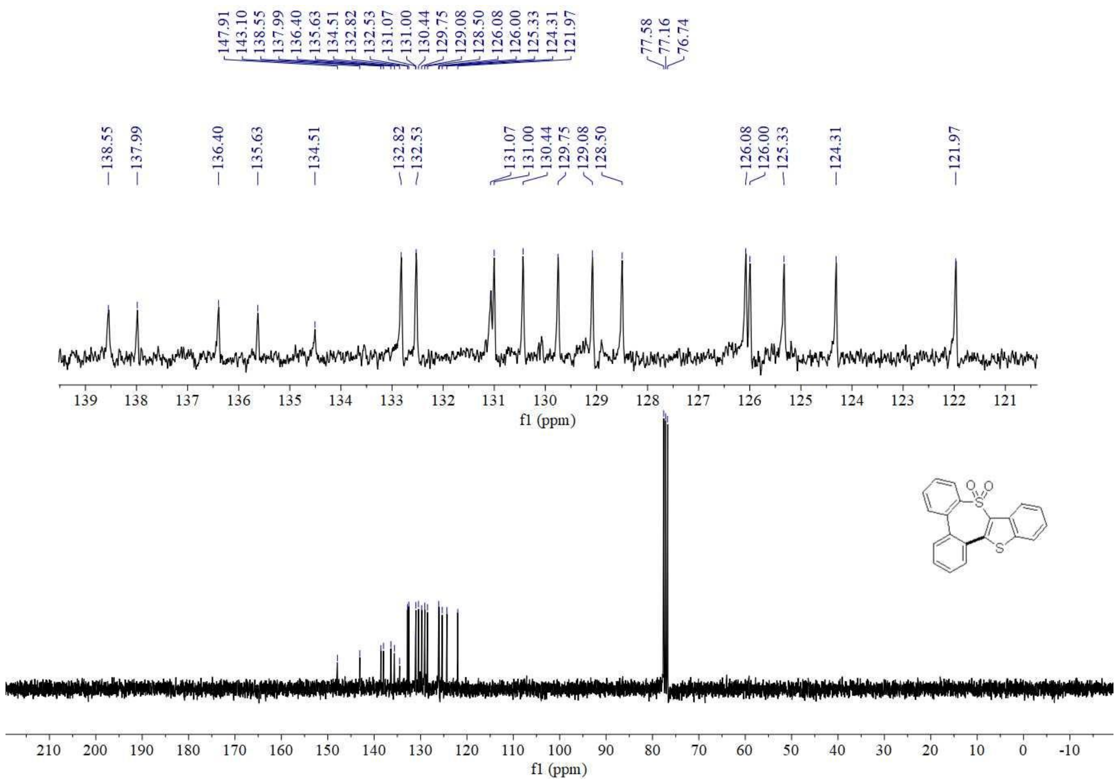

^{1}H NMR spectrum of **10** in $CDCl_3$ (300 MHz)

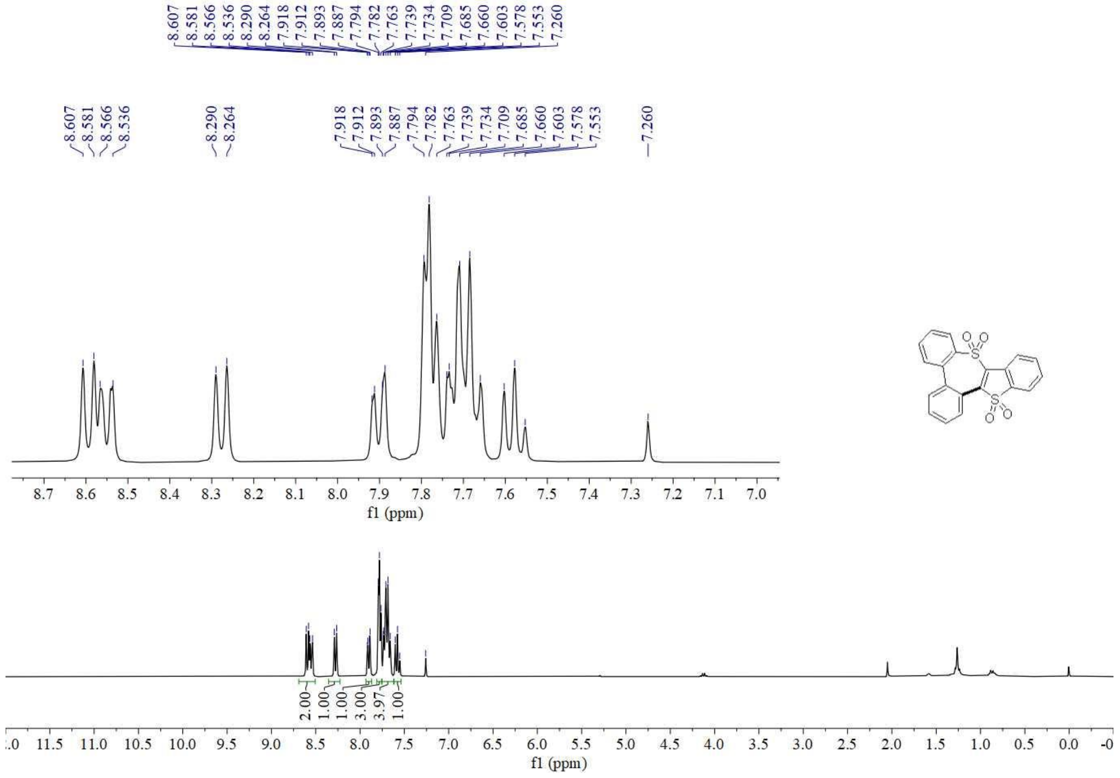

$^{13}C\{^{1}H\}$ NMR spectrum of **10** in $CDCl_3$ (75 MHz)

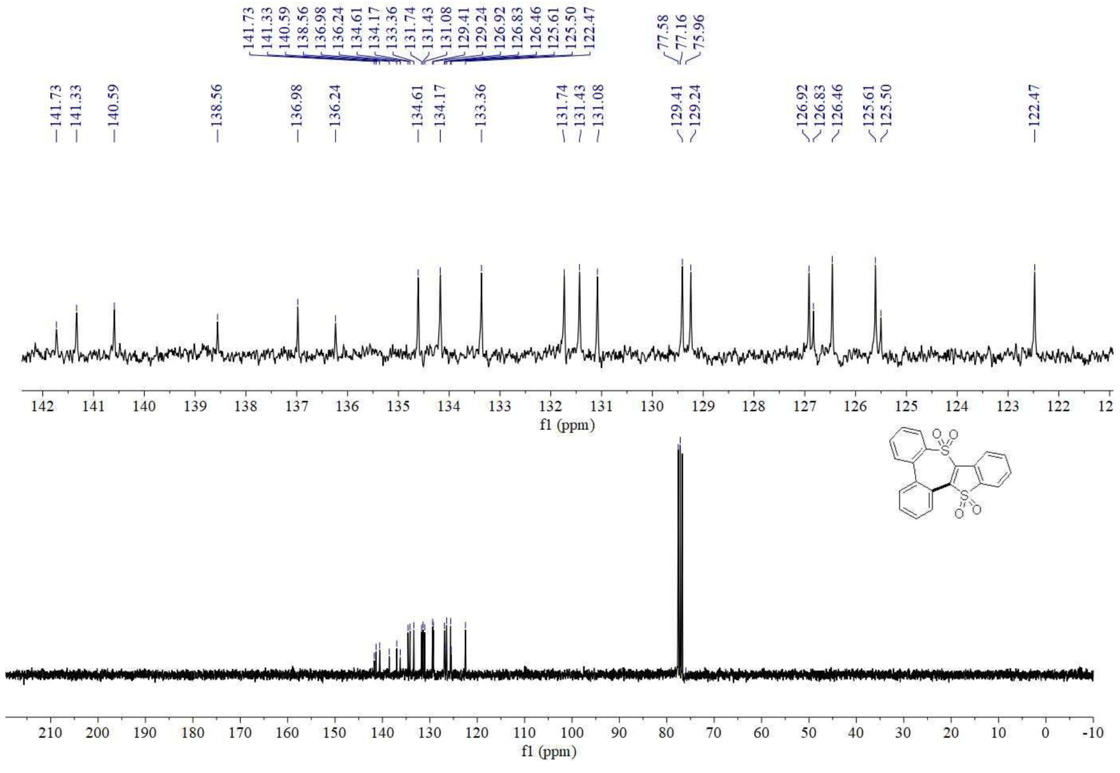

Supplement: SC-017-D5SC08209A-s001 [file SC-017-D5SC08209A-s001.pdf]
